# Deciphering c-MYC-regulated genes in two distinct tissues

**DOI:** 10.1186/1471-2164-12-476

**Published:** 2011-09-30

**Authors:** Samuel C Robson, Lesley Ward, Helen Brown, Heather Turner, Ewan Hunter, Stella Pelengaris, Michael Khan

**Affiliations:** 1 Wellcome Trust/Cancer Research UK Gurdon Institute, Cambridge, CB2 1QN, UK; 2 Molecular Biology Service, University of Warwick, CV4 7AL, UK; 3 Pfizer Global R & D, Kent, CT13 9NJ, UK; 4 Genstruct Inc, One Alewife Center, Cambridge, MA, 02140, USA; 5 Warwick Medical School, University of Warwick, CV4 7AL, UK; 6 Biomedical Research Institute, Department of Biological Sciences, University of Warwick, CV4 7AL, UK

## Abstract

**Background:**

The transcription factor MYC is a critical regulator of diverse cellular processes, including both replication and apoptosis. Differences in MYC-regulated gene expression responsible for such opposing outcomes *in vivo *remain obscure. To address this we have examined time-dependent changes in global gene expression in two transgenic mouse models in which MYC activation, in either skin suprabasal keratinocytes or pancreatic islet β-cells, promotes tissue expansion or involution, respectively.

**Results:**

Consistent with observed phenotypes, expression of cell cycle genes is increased in both models (albeit enriched in β-cells), as are those involved in cell growth and metabolism, while expression of genes involved in cell differentiation is down-regulated. However, in β-cells, which unlike suprabasal keratinocytes undergo prominent apoptosis from 24 hours, there is up-regulation of genes associated with DNA-damage response and intrinsic apoptotic pathways, including *Atr*, *Arf*, *Bax *and *Cycs*. In striking contrast, this is not the case for suprabasal keratinocytes, where pro-apoptotic genes such as *Noxa *are down-regulated and key anti-apoptotic pathways (such as Igf1-Akt) and those promoting angiogenesis are up-regulated. Moreover, dramatic up-regulation of steroid hormone-regulated Kallikrein serine protease family members in suprabasal keratinocytes alone could further enhance local Igf1 actions, such as through proteolysis of Igf1 binding proteins.

**Conclusions:**

Activation of MYC causes cell growth, loss of differentiation and cell cycle entry in both β-cells and suprabasal keratinocytes *in vivo*. Apoptosis, which is confined to β-cells, may involve a combination of a DNA-damage response and downstream activation of pro-apoptotic signalling pathways, including Cdc2a and p19^Arf^/p53, and downstream targets. Conversely, avoidance of apoptosis in suprabasal keratinocytes may result primarily from the activation of key anti-apoptotic signalling pathways, particularly Igf1-Akt, and induction of an angiogenic response, though intrinsic resistance to induction of p19^Arf ^by MYC in suprabasal keratinocytes may contribute.

## Background

The *c-MYC *proto-oncogene encodes a transcription factor, c-MYC (MYC), which regulates the expression of cellular targets involved in a wide range of diverse cellular functions, including cell growth, proliferation, loss of cell-cell contact, loss of differentiation and angiogenesis [1-8]. While the predominant role of physiological MYC in most tissues is to promote G1/S transition in the cell cycle (and thereby proliferation) [1,9,10] and inhibit differentiation [11-13], deregulated MYC (oncogenic) can lead to uncontrolled proliferation and tumour growth [3]. Paradoxically though, MYC is able to act as its own tumour suppressor, as deregulated MYC activity can also promote apoptosis (both *in vitro *[14-16] and *in vivo *[17]) and senescence [18,19]. See [20] for a recent review of the MYC field. Such linkage between seemingly opposing functions - proliferation and apoptosis - is also found in other cell-cycle-associated genes, such as *E2f*, *E1a *and *c-Fos *[21].

The mechanisms by which MYC elicits the vast host of biological responses for which it appears to be responsible are not yet fully understood. Currently, around 1,700 genes have been classified as putative MYC targets [22,23] using methods such as serial analysis of gene expression (SAGE) [24], DNA microarrays [3] and subtractive hybridization [25]. It has been hypothesized that MYC may have the potential to regulate up to 15% of the entire genome [26], leading to it being described as a 'master regulator' of gene expression.

Regulatable transgenic mouse models have allowed controlled activation of a modified MYC-containing chimaeric transcription factor (MYC-ER^TAM^) in distinct cell populations in adult mice, such as the pancreatic islet β-cells [27] and suprabasal keratinocytes (SBK) of skin epidermis [28]. Our previous work has shown that continuous activation of MYC-ER^TAM ^in these diverse tissues exposes the dual potential of MYC to activate pathways involved in cell replication and cell death under differing environmental conditions. In suprabasal epidermis, MYC promotes entry of post-mitotic keratinocytes into the cell cycle, concomitant with loss of differentiation and increased vascularization leading to formation of pre-cancerous papillomas [28]. In contrast, although MYC promotes rapid cell cycle entry of pancreatic β-cells, these cells quickly proceed to undergo apoptosis leading to severe cell depletion and diabetes [27]. This indicates a crucial role for tissue context and the surrounding micro-environment in determining cell fate.

The divergence of MYC-induced phenotypes between these two tissues has enabled us to compare MYC-regulated gene expression patterns over a time course of MYC-ER^TAM ^activation, by employing high-throughput transcriptome analysis using microarrays. Comparison of the transcriptional response between the two tissues identified potential signalling pathways which may promote apoptosis of β-cells and prevent apoptosis in SBK; the DNA-damage response pathway, and the Insulin-like growth factor 1 (Igf1) signalling pathway, respectively. In addition, up-regulation of angiogenesis-related genes and of those encoding members of the steroid hormone-regulated Kallikrein serine protease family was found in SBK but not in β-cells. Kallikreins may increase availability and action of Igf1 through proteolysis of Igf1 binding proteins. Together with angiogenesis, Kallikreins may provide a local tissue-specific regulatory mechanism for determining ultimate MYC function *in vivo*.

## Results and Discussion

### Activation of MYC-ER^TAM ^mediates transcription of genes involved in a wide range of cellular functions

Time courses were set up following activation of MYC in β-cells (pancreas) and SBK (skin) via administration of 4-hydroxytamoxifen (4OHT) for 4 hrs, 8 hrs, 16 hrs and 32 hrs as described (Methods). Vehicle-treated samples acted as direct time point controls for 4OHT-treated samples. Laser capture microdissection (LCM) was utilized to allow isolation of pancreatic islet tissue. Significant gene expression changes for the main experimental conditions (4OHT treatment, tissue type and time point following initial treatment) and their interactions, as well as information on the effects of further covariates such as batch effects and RNA quality, were identified using a custom R package, *Envisage *(available on request).

**Figure 1:**
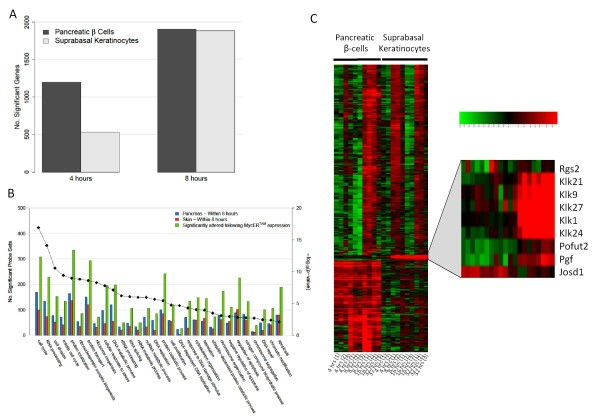
**Transcriptional response to MYC-ER^TAM ^activation**. A) Activation of MYC-ER^TAM ^led to a significant change in expression of a large number of genes within only 4 hours for the pancreas (≥ 2-fold change, p ≤ 0.05). A similar transcriptional response was not detected until 8 hours post-MYC-ER^TAM ^activation for SBK. B) Analysis of 6,633 genes, whose expression was altered within 8 hours upon activation of MYC-ER^TAM ^in either pancreas or skin (green bars), identified enrichment of genes in a wide range of MYC-mediated functions. Analyses of genes whose expression was altered greater than 2-fold within 8 hours for the SBK (red bars) and the pancreatic β-cells (blue bars) showed a similar level of response to MYC-ER^TAM ^activation in both tissues within 8 hours, with a slight increase in the level of response for the pancreas as compared to the skin, in particular for DNA-replication and response to DNA-damage. p-values represent significance of enrichment of GO classes for the 6,633 genes. C) Genes identified as showing significant differential expression due to the joint effects of MYC-ER^TAM ^activation and tissue (with or without an interaction with the time point) were clustered to identify functionally related genes (red, up-regulated; black, no change; green, down-regulated). The blow-up identifies a group of functionally related genes showing increased expression in SBK but not islet β-cells.

Analysis of gene expression for 12,349 curated probe sets (Methods) identified 6,633 unique genes as being significantly altered following activation of MYC with a false discovery rate of 5%. 1,615 genes showed significant effects for the joint effects of 4OHT treatment, time and tissue type; 2,015 genes showed significant effects for the interaction between 4OHT treatment and tissue type; 2,221 genes showed significant effects for the interaction between 4OHT treatment and time; and 1,843 genes showed significant effects for the main effect of 4OHT treatment only. Of the MYC-responsive genes, the expression levels of 1,199 were altered greater than 2-fold (up- or down-regulated) after only 4 hours of 4OHT treatment for the pancreatic β-cells, while only 530 were similarly affected for SBK. However, at 8 hours following initial 4OHT treatment, the expression levels of 1,905 and 1,882 were altered greater than 2-fold for the pancreas and the skin respectively (Figure 1A). This suggests a more prominent initial response to MYC in β-cells compared to SBKs.

Gene ontology (GO) enrichment analysis using the DAVID functional annotation tool [29] identified enrichment of genes involved in myriad cellular functions following activation of MYC. The majority of MYC-responsive genes were involved in metabolic, transcriptional, transportational and signal transduction pathways (Figure 1B; green bars). Genes involved in post-transcriptional modification (e.g. RNA processing, RNA splicing) and post-translational modification (e.g. protein catabolic process, protein folding, protein localization and ubiquitin-dependent catabolic process) were also significantly enriched. Similarly, a significant enrichment of genes relating to ribosome biogenesis was detected, suggestive of MYC's recently elucidated role as a regulator of ribosome biogenesis and protein synthesis [30]. As expected given the role of MYC in proliferation, genes involved in cell cycle progression were amongst the most significantly enriched (p = 1.21 × 10^-17^). Genes involved in apoptosis and DNA-damage checkpoint pathways were also enriched (p = 7.81 × 10^-3 ^and p = 5.07 × 10^-5 ^respectively), along with genes involved in related functions such as cellular response to stress (p = 1.56 × 10^-8^) and cytoskeleton organisation (p = 9.12 × 10^-5^).

Enrichment of GO terms for MYC-responsive genes showing early changes in expression (≥ 2-fold change within the first 8 hours of MYC activation) for the pancreas (Figure 1B; blue bars) and skin (Figure 1B; red bars) identified similar numbers for both tissues, with the exception of genes relating to DNA-damage and DNA replication, where a larger number of genes are detected for the pancreas than for the skin. These results indicate that whilst expression of genes relating to multiple cellular functions is common to both tissues, expression of genes involved in DNA-damage and replication is more specific to the β-cells.

### Expression of putative MYC target genes following MYC-ER^TAM ^activation

The MYC Target Gene Database [31] currently identifies 1,697 genes as putative MYC targets [22]. Of these, 13.4% and 19.2% were found to be both MYC-responsive and show a 2-fold change in expression in the skin and pancreas respectively within 8 hours (Additional file 1, Table S1). The predominant role for these genes was in DNA replication, biosynthesis, metabolism, cell cycle, cell division and other related functions. Cellular functions relating to apoptosis and cell death were also highly enriched, although to a lesser degree than those relating to cellular proliferation. These data suggest that activation of the MYC-ER^TAM ^protein *in vivo *leads to a rapid change in the expression of a large number of putative MYC targets. However, known target genes represent only a small fraction of detected MYC-responsive genes, indicating that the majority of these observed expression changes may be downstream of direct MYC-induced transcription.

To identify the level of overlap between the genes classed as significantly altered in this study and those identified in previous analyses, we utilised the Gene Set Enrichment Analysis (GSEA) program developed by the Broad Institute [32]. This allowed us to identify gene sets in which significant differentially expressed genes are enriched. Gene sets were taken from the Molecular Signatures Database (MSigDB), as well as from additional published datasets. Additional file 1, Table S7 and Additional file 1, Table S8 show the results from GSEA (FDR < 0.01) for the genes showing significant expression at the early time points (4 hours and 8 hours) for the pancreas and skin, respectively. These results indicate a clear enrichment in the pancreas of genes annotated as being related to MYC function from previous publications, including MYC target genes from the MYC Target Gene Database [22], genes indicative of a MYC-induced oncogenic signature from Blid *et al. *[33] and genes up-regulated in the studies of Coller *et al*., Schumacher *et al*., Yu *et al*., and Lee *et al. *[3,34-36]. Similarly, down-regulated genes showed enrichment with down-regulated genes from the study of Yu *et al. *[36]. In comparison, genes up-regulated in the skin are enriched for genes also found to be up-regulated in the studies of Coller *et al*., Yu *et al. *and Zeller *et al. *[3,22,36], while MYC target genes from the MYC Target Gene Database [22], genes indicative of a MYC-induced oncogenic signature from Blid *et al. *[33] were enriched with an FDR value < 0.017. This suggests that the gene expression signature identified in both the pancreas and skin is indicative of changes in expression related to MYC function.

### Cell cycle response following MYC activation

**Figure 2:**
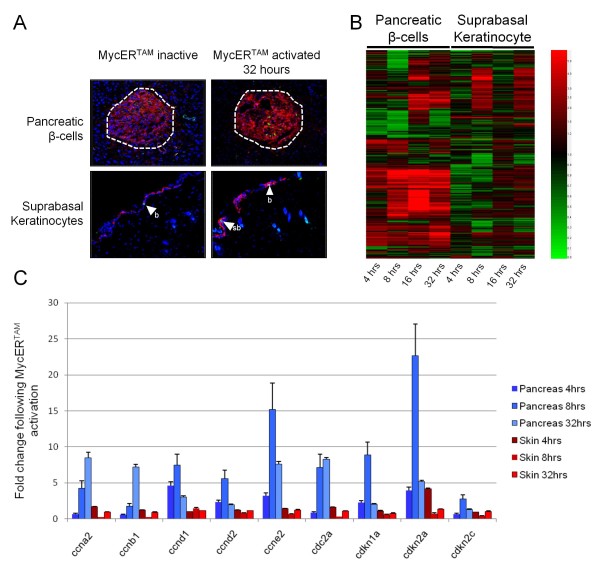
**Cell cycle following activation of MYC-ER^TAM^**. A) Sections from 4OHT-treated MYC-ER^TAM ^transgenic mice were stained with Ki67-specific antibodies (green) and a tissue specific antibody (red; insulin for β-cells, keratin 1 for SBK). DAPI staining (blue) identifies cell nuclei. No Ki67 staining was detected in β-cells or SBK in vehicle-treated samples, although proliferating basal cells in the skin showed clear staining. Following 32 hours of 4OHT, staining of both β-cells and SBK was detected, indicated by concomitant staining of Ki67 and the tissue-specific protein. Images shown are representative sections from different levels through replicate tissue samples. b, basal keratinocyte; sb, suprabasal keratinocyte. B) The change in expression of genes relating to cell cycle following activation of MYC-ER^TAM ^is represented as a heatmap (red, up-regulated; black, no change; green, down-regulated). Significant change in expression is detected for key early G1-S phase transition genes such as *Ccnd1, Ccnd2 *and *Ccne2*, indicating activation of cell cycle progression, particularly for the pancreas. C) qRT-PCR validation for gene expression changes in genes relating to cell cycle progression indicated a clear proliferative response in the pancreatic β-cells following MYC-ER^TAM ^activation. In particular, genes relating to G1/S-phase transition such as *Ccnd1*, *Ccnd2 *and *Ccne2 *showed early changes in expression (8 hours), whilst genes whose products relate to later cell cycle events such as *Ccna2 *and *Ccnb1 *showed increased expression at later time points (32 hours). Few significant changes were detected for these key proliferation markers in SBK. This may be due to the relatively low proportion of keratinocytes that are responsive to Myc-induced proliferation at these early time-points, whilst differentiated keratinocytes of the granular layer are refractory to the proliferative influence of MYC.

One of the key functions of the MYC onco-protein is promotion of cell cycle progression, particularly G1/S phase transition [1,2,37]. Activation of MYC in suprabasal keratinocytes (SBK) and pancreatic β-cells has been previously shown to initiate G1/S transition in target cells [27,28,38], and this was seen through immunohistological staining with anti-Ki67 antibodies (Figure 2A), as well as in the response detected in genes relating to cell cycle progression by gene ontology classification (Figure 1B and Additional file 1 Table S2). A subset of some interesting genes from this list is shown in Table 1.

**Table 1:** Cell cycle and cell growth genes responsive to MYC-ER^TAM ^activation

		Pancreatic β-Cells				Suprabasal Keratinocytes				
		
Gene Symbol	RefSeq	4 Hours	8 Hours	16 Hours	32 Hours	4 Hours	8 Hours	16 Hours	32 Hours	Myc-response p-value
										
**Cell Cycle**										
										
ccna2	X75483	0.96	4.52**	10.49**	3.25**	0.81	0.67	1.39	0.97	2.05E-02
ccna2	X75483	0.55**	1.33*	12.39**	3.47**	0.63**	0.71*	0.99	1.05	1.12E-02
ccnb1	NM_007629	1.02	0.90	2.38**	1.13	0.48**	1.03	1.01	0.58**	9.74E-03
ccnb1	NM_007629	1.06	2.85**	8.15**	3.62**	0.60	0.46*	1.39	0.61	3.73E-02
ccnd1	NM_007631	1.86**	2.09**	0.88	1.45	1.13	1.31	1.79*	1.04	4.55E-02
ccnd1	NM_007631	1.69	2.38	1.41	2.27*	1.05	1.33	1.72	1.26	2.24E-02
ccnd1	NM_007631	3.41**	2.74*	2.03*	2.05	1.35	0.99	1.54	1.81	2.23E-02
ccnd2	NM_009829	1.63*	0.61*	2.28**	2.95**	1.37	1.46	0.94	1.40	2.62E-02
ccnd2	NM_009829	1.82	1.03	1.22	1.90*	0.83	1.33	0.97	1.78	1.48E-02
ccnd2	NM_009829	2.06**	2.04**	1.27	2.30**	0.90	0.84	1.29	0.84	1.21E-02
ccnd2	AK007904	1.99**	0.64*	1.88**	2.62**	1.66*	2.57**	1.00	2.13**	1.28E-03
ccnd2	NM_009829	2.09*	1.00	1.06	2.01*	1.26	0.96	1.12	1.51	2.38E-02
ccnd3	NM_007632	1.26*	0.84	1.54**	1.20	0.81*	2.58**	0.84	3.33**	8.71E-04
ccne1	NM_007633	1.82**	3.37**	7.02**	1.71*	0.94	1.35	1.15	1.82**	2.52E-02
ccne1	BB293079	1.83**	6.68**	1.87**	2.04**	0.85	1.50*	1.16	1.29	1.02E-02
ccne2	AF091432	2.62**	5.86**	5.92**	7.86**	1.74	1.11	1.42	1.39	1.45E-02
cdc2a	NM_007659	0.95	2.91**	11.93**	3.35**	0.51*	1.17	0.89	1.12	4.33E-02
cdc25a	C76119	2.71**	2.23**	2.09**	3.90**	1.11	0.46**	1.07	0.78	1.84E-03
cdc25a	C76119	2.17**	1.73*	2.03**	2.10**	1.02	1.14	1.1	1.21	2.45E-02
cdk4	NM_009870	1.16	0.92	8.41**	1.11	1.32	12.27**	0.74	1.62	2.70E-02
cdk4	NM_009870	1.21	0.74	5.31**	1.5	1.23	11.30**	0.92	1.48	3.31E-02
cdk4	NM_009870	1.20	0.88	5.03**	1.3	1.37	11.17**	0.72	1.37	3.45E-02
cdk7	U11822	0.63*	0.77	3.69**	3.45**	0.82	2.12**	1.09	1.59*	1.37E-02
cks2	NM_025415	0.92	3.03**	10.08**	4.80**	0.51**	0.76	1.27	0.71	4.37E-03
cks2	NM_025415	0.98	2.33**	8.92**	2.34**	0.63*	0.92	0.93	1.32	1.18E-02
pcna	BC010343	1	2.44**	17.91**	4.75**	1.32	3.24**	0.76	3.08**	3.38E-02
pcnt	NM_008787	2.70**	3.79**	5.22**	4.18**	1.14	1.89**	1.10	1.82**	5.43E-06
										
**Cell Cycle Arrest**										
									
cdkn1a	AK007630	1.57**	2.05**	1.71**	1.24	0.96	1.44*	0.99	0.90	5.01E-03
cdkn1b	NM_009875	1.03	0.19**	0.71	0.95	0.23**	0.42*	0.48*	0.89	1.02E-02
cdkn2a	NM_009877	2.17**	1.79**	1.57**	2.61**	1.21	1.07	1.07	0.87	8.43E-03
cdkn2c	BC027026	0.32**	1.18	5.62**	0.59*	0.85	1.28	0.87	1.60*	9.38E-03
gadd45g	AK007410	0.59*	0.41**	1.88**	0.59**	4.07**	5.16**	3.39**	2.78**	5.95E-03
gas6	NM_019521	0.60**	0.73**	1.02	0.90	1.39**	2.61**	1.43**	5.62**	2.31E-02
ing3	BB298005	0.90	1.12	1.15	0.66**	0.79	0.49**	0.77*	1.34*	1.50E-02
										
**Cell Growth and Cytoskeleton Organization**										
							
add1	BF140063	0.99	0.96	1.16	0.96	0.85	2.57**	0.86	3.93**	1.94E-02
add1	BF140063	0.82	0.80	0.62**	0.56**	1.25	2.41**	1.24	0.44**	8.01E-03
cap1	NM_007598	4.88*	0.80	1.16	1.14	1.50	8.59**	0.87	0.17**	4.61E-02
cfl1	NM_007687	1.29	0.51**	1.30	1.89**	1.03	2.28**	1.15	2.14**	4.26E-02
creg1	BC027426	1.06	0.83	2.39**	1.72**	0.82	1.54*	0.69*	1.09	3.57E-02
creg1	BC027426	2.08**	2.12**	1.60**	2.16**	0.69*	0.85	0.98	0.92	3.68E-02
egf	NM_010113	1.05	0.79	0.92	1.38*	1.62**	0.47**	0.91	0.84	1.28E-02
lsp1	NM_019391	0.90	0.78*	0.97	1.10	1.02	3.42**	0.67**	1.90**	3.22E-02
myh4	BG794681	0.99	1.25	0.65	1.06	1.72	2.94*	0.94	1.55	8.16E-03
pdlim3	NM_016798	1.00	1.09	1.07	0.78	0.85	2.73**	1.49*	2.29**	4.52E-02
plec1	BM210485	1.31	0.49*	1.25	3.10**	0.53	1.38	1.04	2.87**	2.42E-02
plec1	BM232239	1.20	0.77	1.35	2.46**	3.23**	4.56**	2.82**	0.59*	2.09E-03
tuba3a	NM_009446	0.97	0.97	1.13	1.00	0.83	3.09**	0.89	3.20**	1.22E-02
tubb2c	BC005547	1.06	1.04	4.72**	1.48*	1.13	2.01**	1.04	1.41*	3.23E-02
tubb6	NM_026473	1.21	0.90	2.95**	1.71*	0.97	2.99**	1.28	3.49**	2.41E-02

Selected MYC-responsive genes relating to cell growth and cell proliferation. 'MYC-response p-value' is the p-value identified for the highest-order interaction of the MYC activation variable and represents the significance of this term within the selected model. Flags represent contrast p-values comparing 4OHT-treated and vehicle-treated samples at specific time points ('*', p ≤ 0.05; '**' p ≤ 0.01). Cells are colour-coded based on a detected fold-change greater than 1.5-fold (red, up-regulated; blue, down-regulated)

Activation of MYC in the β-cells resulted in a change in expression of 213 cell cycle- and proliferation-related genes within 8 hours of MYC activation, with 116 genes up-regulated and 101 genes down-regulated (Additional file 1, Table S2). *Pcna*, a MYC target gene associated with the cell cycle, was expressed in both pancreatic β-cells and SBK throughout most of the time-course, with *Cdc25a *expressed only in the former. In β-cells, cyclin genes *Ccnd1, Ccnd2 *and *Ccne2*, whose products are necessary for G_1_/S-phase transition in the cell cycle, were up-regulated within 4 hours of MYC activation. Cyclin genes *Ccna2, Ccnb1 *and *Ccne1*, whose products are involved in later G_1_/S-phase and G_2_/M-phase cell cycle events, were up-regulated greater than 3-fold subsequently at 8 hours (Table 1 and Figure 2C).

Activation of MYC in the SBK resulted in a less prominent cell cycle response compared to β-cells, with a change in expression of 144 cell cycle- and proliferation-related transcripts within 8 hours of MYC activation - 73 genes up-regulated and 74 genes down-regulated (Additional file 1, Table S2). Of G_1_/S-phase cell cycle genes, *Ccnd2 *and *Ccnd3 *showed a ≥ 2-fold increase in expression at 8 hours. In contrast to β-cells, later cell cycle genes such as *Ccna2 *and *Ccne2 *were either down-regulated or unchanged in SBK. Interestingly, the *Ccnb1 *gene whose product, cyclin B1, is involved predominantly in later cell cycle events (G2/M transition), was down-regulated 2-fold in SBK early in the time-course. Cyclin B1 interacts with Cdc2a (Cdk1) to form the mitotic initiation complex, which was also down-regulated by ~2-fold at 4 hrs in epidermis. This contrasts with β-cells, in which both *Ccnb1 *and *Cdc2a *showed significant up-regulation. Although speculation here, these findings may suggest SBK are able to enter the G1/S phase of the cell cycle, which is a known function of MYC, but not progress through the G2/M phase. Interestingly, it was previously shown that over-expression of MYC causes a P53-dependent G2 arrest in normal fibroblasts [39]. Such cells may then be enforced by MYC to reinitiate DNA replication, resulting in aneuploidy.

Of these cell cycle-related genes, *Ccna2 *[40] and *Ccnd1 *[41,42] have been previously designated as putative MYC targets through high-throughput screening, and *Ccnb1 *[43] and *Ccnd2 *[44-46] have been previously confirmed as direct transcriptional targets of MYC through the use of chromatin Immunoprecipitation (ChIP) analysis. The cyclin D2-related kinase *Cdk4*, also a previously characterized direct MYC target [47], showed increased expression after 4 hours of MYC activation in the pancreas, with a 6-fold increase detected subsequently at 16 hours. *Cdk4 *was also found to be highly up-regulated at 8 hours in the skin, with a fold change of almost 12 (Table 1). No significant change was detected for the cyclin E-associated CDK gene *Cdk2 *in either the skin or the pancreas, However *Cdk7*, which has a role in both activating cyclin complexes and regulating transcription, was up-regulated at 8 hours in the skin.

Down-regulation of another known MYC target gene, the cyclin dependent kinase (CDK) inhibitor *Cdkn1b *(p27^Kip1^), which inhibits G_1_/S-phase transition by association with the cyclin E-Cdk2 complex [48], was detected for both the skin and the pancreas. Also, the expression of *Cks2*, a MYC target gene whose product is involved in degradation of p27^Kip1^, increased from 8 hours following MYC activation in the pancreas. Interestingly, the *Cdc2a *gene, whose product Cdk1 is essential for mammalian cell division [49,50], was also found to be highly up-regulated in β-cells (Table 1). Cdk1 has been found to substitute for other CDKs to drive cell cycle progression [51], and is particularly associated with Cdk4 in G_1_/S-phase progression [reviewed in 52]. This may indicate a significant role for Cdk1 in the promotion of cell cycle progression following MYC activation in β-cells. Alternatively, it has been shown that premature activation of Cdk1 can lead to mitotic catastrophe in G_2_/M-phase and apoptosis (upstream of p53-induced mitochondrial outer membrane permeabilization) in neurons [53]. Given that this CDK was also detected at later time points, this may indicate a possible role for Cdk1 in the MYC-induced apoptosis pathways. In addition to this, the CDK inhibitor *Cdkn2c *(p18^Ink4c^), which inhibits G_1_/S-phase transition via interactions with Cdk4 [54] and Cdk6 [55], was down-regulated early in the pancreas. However, by 16 hours expression levels had risen dramatically, which may be indicative of cell cycle arrest prior to apoptosis. In addition, the CDK inhibitor *Cdkn1a *(p21^Cip1^) - a downstream target of the tumour suppressor p53 - was up-regulated at 8 hours (Table 1).

Growth arrest specific gene 6 (*Gas6*) was up-regulated in SBK (Table 1), and has been found to have different roles dependant on tissue location, including promotion of cell cycle progression in fibroblasts and Schwann cells [56,57], and has been shown to play a role in cellular survival by inducing signalling through the PI3K/Akt pathway leading to phosphorylation of the forkhead box protein Foxo1 in endothelial cells [58] and in oligodendrocytes of the central nervous system [59]. Interestingly, the growth arrest and DNA-damage-inducible 45 gamma gene, *Gadd45g*, a proposed MYC target whose product is involved in growth arrest at the G_2_/M DNA-damage checkpoint [60], showed increased expression at 4 hours in SBK, and remained 3-fold up-regulated throughout the time course, whilst down-regulation at 8 hours was detected in β-cells. This suggests potential activation of pathways to limit unchecked proliferation in the keratinocytes (Table 1).

Genes relating to increased cellular mass, cytoskeleton organization and DNA replication were also detected for SBK (Additional file 1, Table S3), including the membrane skeletal proteins Adducin 1 (*Add1*) and *Pdlim3*, the actin-modulating protein Cofilin 1 (*Cfl1*), members of the kinesin family of microtubule motor proteins, members of the myosin superfamily of actin binding motor proteins (e.g. *Myh4*, *Myoc*, *Myo1f*), and members of the tubulin family of microtubule proteins (*Tuba3a*, *Tubb2c*, *Tubb6*). Plectin 1 (*Plec1*), one of the main components of the cytoskeleton, showed an increase in expression of roughly 3- to 4-fold throughout the early stages of the time course (Table 1). This increased activity of microtubule formation and actin formation for both the pancreas and skin is indicative of increased cellular turnover in both tissues.

### Apoptotic response following MYC activation

**Figure 3:**
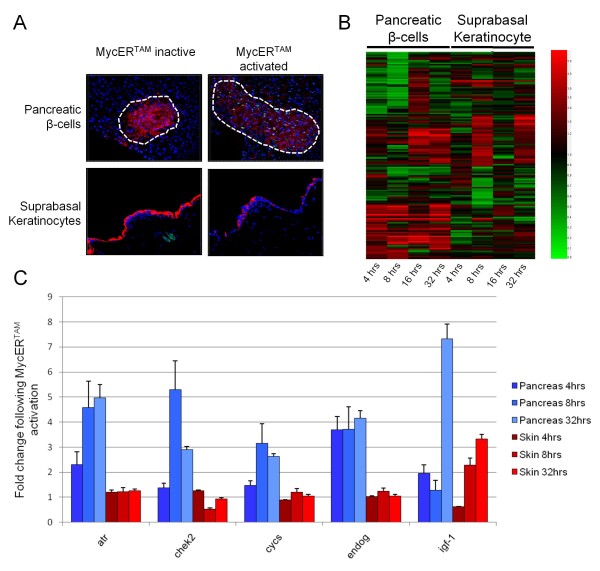
**Apoptosis following activation of MYC-ER^TAM^**. A) Sections from 4OHT-treated MYC-ER^TAM ^transgenic mice were stained with Caspase 3-specific antibodies (green) and a tissue specific antibody (red; insulin for β-cells, keratin 1 for SBK). DAPI staining (blue) identifies cell nuclei. No Caspase 3 staining was detected in β-cells or SBK in vehicle-treated (VT) samples. Following 32 hours of 4OHT, Caspase 3 was detected in β-cells, but no Caspase 3 activity was detected in treated SBK indicating that apoptosis is specific to the β-cell model. Images shown are representative sections from different levels through replicate tissue samples. b, basal keratinocyte; sb, suprabasal keratinocyte. B) The change in expression of genes relating to apoptosis and cell survival following activation of MYC-ER^TAM ^is represented as a heatmap (red, up-regulated; black, no change; green, down-regulated). C) qRT-PCR validation for gene expression changes in genes relating to apoptosis and cell survival confirms disparate changes in gene expression between skin and pancreas. Genes relating to DNA-damage in particular, such as *Atr *and *Chk2*, show a clear increase in expression for pancreatic β-cells, which confirms changes seen in microarray data, as did apoptosis genes *Cycs *and *Endog*. As with the microarray data, up-regulation of the *Igf1 *gene was detected from 8 hours for the skin following MYC-ER^TAM ^activation, with an increase in expression also detected for the pancreas at the later 32 hour time point.

The ultimate phenotypic response to activation of MYC in pancreatic β-cells is apoptosis [27]. Immunohistological staining for Caspase 3, an early marker for initiation of apoptosis pathways, indicated an apoptotic response to MYC activation in the β-cells but not in the SBK (Figure 3A). In contrast, MYC-activated SBK that have begun a process of terminal differentiation, re-enter the cell cycle but are protected from conventional apoptosis [33,38]. These cells will ultimately be shed and removed from the surrounding micro-environment thus ridding the host of potentially harmful pre-cancerous cells [61]. Our array data confirm a large transcriptional response detected in genes relating to apoptosis and survival by gene ontology classification in both tissues (Figure 3B and Additional file 1 Table S4). A subset of important genes from this list is shown in Table 2.

**Table 2:** Apoptosis and cell survival genes responsive to MYC-ER^TAM ^activation

		Pancreatic β-Cells				Suprabasal Keratinocytes				
		
Gene Symbol	RefSeq	4 Hours	8 Hours	16 Hours	32 Hours	4 Hours	8 Hours	16 Hours	32 Hours	Myc-response p-value
										
**Apoptosis**										
										
bax	BC018228	1.09	1.65**	2.49**	1.72**	1.37*	1.70**	1.87**	1.99**	4.61E-02
birc4	BF134200	0.52*	0.64	1.76	1.77*	0.71	0.72	0.77	1.10	3.12E-02
birc4	BF134200	0.69*	0.58*	0.78	0.81	0.53**	0.31**	1.14	0.75	5.29E-03
birc5	BC004702	0.57*	1.99**	16.53**	1.90**	0.61**	1.13	1.06	1.25	8.88E-03
cdc2a	NM_007659	0.95	2.91**	11.93**	3.35**	0.51*	1.17	0.89	1.12	4.33E-02
cdkn2a	NM_009877	2.17**	1.79**	1.57**	2.61**	1.21	1.07	1.07	0.87	8.43E-03
cflar	BE284491	1.03	0.97	1.00	0.94	1.04	1.00	1.28**	2.34**	4.59E-02
cflar	BE284491	0.82*	0.87	0.54**	0.43**	1.00	1.13	1.03	1.09	2.22E-02
cflar	AK020765	0.78*	0.75*	1.18	0.51**	1.05	1.16	1.01	0.73*	4.40E-02
cycs	NM_007808	0.96	0.87	2.54**	2.38**	0.99	1.62**	0.96	0.91	2.84E-02
cycs	NM_007808	1.17*	1.98**	1.55**	2.05**	0.83*	1.00	1.17	1.05	1.64E-03
d2wsu81e	AV104666	2.85**	2.94**	1.50**	1.85**	0.74*	1.01	0.95	1.24	1.19E-05
endog	NM_007931	2.49**	2.90**	2.40**	1.96**	0.75*	1.46**	1.20	1.74**	2.48E-02
fas	BG976607	6.55**	6.15**	3.85**	11.34**	0.82	1.22	1.61	1.35	3.79E-02
fas	BG976607	3.61**	7.00**	1.13	4.43**	1.33	0.63	1.35	0.46**	6.32E-03
fas	BG976607	5.08**	12.85**	5.07**	10.92**	0.72	5.14**	0.74	0.48*	1.12E-03
fas	BG976607	2.43**	1.78**	2.28**	2.50**	0.83	1.33	1.09	0.39**	2.21E-03
ing3	BB298005	0.9	1.12	1.15	0.66**	0.79	0.49**	0.77*	1.34*	1.50E-02
pmaip1	NM_021451	1.15	1.13	1.02	0.85	1.33*	0.45**	0.77*	0.87	6.77E-03
tnfrsf12a	NM_013749	0.59**	0.85	2.53**	0.72**	1.11	3.15**	1.38**	1.17	4.16E-02
tnfrsf12a	NM_013749	0.86	0.87	2.29**	0.85	1.18	2.77**	1.39**	1.11	4.89E-03
tnfrsf4	NM_011659	1.18	0.60*	1.46*	1.64**	1.27	2.12**	1.76**	1.03	5.78E-03
										
**Cell Survival**										
										
akt1	NM_009652	1.01	0.97	1.71**	2.31**	0.82	3.23**	0.95	2.29**	4.43E-02
akt2	NM_007434	1.00	1.05	0.98	1.13	1.27	2.21**	1.02	2.26**	7.85E-03
akt2	BC026151	0.78	0.89	2.31**	2.69**	0.99	1.74**	1.01	1.70**	3.97E-02
igf1	NM_010512	2.08*	0.63	0.74	1.20	1.17	2.33**	1.47	2.00*	4.75E-02
igf1	BG075165	0.97	1.11	1.25	2.11**	1.03	1.09	2.34**	3.54**	4.66E-02
igf1	AF440694	1.05	1.00	1.59	3.71**	1.56*	2.24**	1.24	3.08**	4.10E-02
										
**DNA-damage**										
										
atm	NM_007499	0.84	0.43**	1.12	1.08	0.90	0.92	1.46	0.60*	1.62E-04
atr	AF236887	2.25**	3.79**	2.71**	2.76**	1.17	1.17	1.44*	1.53**	3.46E-02
chek1	BB298208	1.10	3.70**	2.35**	1.92**	0.91	1.03	1.31	1.23	4.05E-03
chek1	C85740	1.94*	4.38**	4.71**	4.17**	0.90	0.48*	1.28	1.03	1.62E-02
chek1	NM_007691	1.37	1.79*	3.16**	1.73**	0.74	0.88	1.38	1.33	5.83E-03
chek2	NM_016681	1.24	2.07**	2.65**	2.29**	0.82	0.63*	1.13	1.34	1.98E-02
gadd45g	AK007410	0.59*	0.41**	1.88**	0.59**	4.07**	5.16**	3.39**	2.78**	5.95E-03
h2afx	NM_010436	1.17	2.17**	4.56**	1.40**	0.67**	1.22*	0.97	1.01	3.43E-03
hus1	NM_008316	0.85	1.97**	2.04**	1.62**	0.97	0.96	1.24	1.23	1.46E-02
hus1	AF076845	1.37	1.05	2.12**	1.29	1.02	1.06	1.07	1.24	1.85E-02
rad1	NM_011232	2.12**	2.27**	2.13*	1.58	0.67	3.18**	0.79	1.94*	3.77E-03
rad51	NM_011234	1.02	6.19**	23.30**	2.40**	0.82	1.12	1.90**	1.46	1.80E-02

Selected MYC-responsive genes relating to apoptosis and cell survival. 'MYC-response p-value' is the p-value identified for the highest-order interaction of the MYC activation variable and represents the significance of this term within the selected model. Flags represent contrast p-values comparing 4OHT-treated and vehicle-treated samples at specific time points ('*', p ≤ 0.05; '**' p ≤ 0.01). Cells are colour-coded based on a detected fold-change greater than 1.5-fold (red, up-regulated; blue, down-regulated).

Activation of MYC in pancreatic β-cells identified a significant change in expression for 92 genes relating to cell death and apoptosis. Of these, 42 genes showed an increase and 50 genes showed a decrease in expression (Additional file 1, Table S4). Early activation of key regulators of apoptosis featured prominently in these data. Activation of MYC in SBK resulted in significant changes in expression for 66 genes relating to apoptosis and cell survival, including 37 genes showing an increase and 29 genes showing a decrease in expression (Additional file 1, Table S4).

The tumour suppressor *Cdkn2a*, which encodes for the CDK inhibitor p16^Ink4a ^and the alternative reading frame tumour suppressor p19^Arf ^(p14^Arf ^in humans), was up-regulated 2-fold at 4 hours and remained at an elevated level throughout the time course for β-cells, but remained unchanged in SBK (Table 2 and Figure 3C). Previous studies have identified a possible role for the p19^Arf^/p53/Mdm2 tumour suppressor pathway in MYC-mediated apoptosis [62,63]. Importantly, data from the Evan lab has elegantly shown that high levels of MYC-ER^TAM ^activation present in this transgenic model, led to expression of p19^Arf ^concomitant with apoptosis [64]. In contrast, low levels of MYC-ER^TAM ^activation did not induce apoptosis or p19^Arf ^but rather β-cell proliferation. However, Finch *et al. *previously showed that loss of p19^Arf ^in β-cells of the MYC-ER^TAM ^transgenic model resulted in mainly increased proliferation, not suppression of apoptosis [65]. Thus the role of p19^Arf ^in defending against aberrant oncogenic MYC-induced hyper-proliferation may be related to cell cycle arrest, and not directly to apoptosis pathways in β-cells.

The anti-apoptotic function of Bclx_L _seen in Rip7-Bclx_L_/pIns-MYC-ER^TAM ^(RM) double transgenic mice indicates that MYC-induced apoptosis is related to the Bax/Bak-mediated intrinsic mitochondrial pathway [27,66]. Activation of this intrinsic apoptotic pathway was evident at the transcriptional level in β-cells by continued 2-fold increased expression of *Bax *and the somatic Cytochrome c gene (*Cycs*) from 16 hours onwards after MYC activation, and up-regulation of the mitochondrial respiratory gene for Endonuclease G, *Endog*, after only 4 hours of MYC activation. Both *Bax *[67,68] and *Cycs *[42,69] have been previously shown to be putative direct MYC targets due to the presence of non-canonical E-box MYC-Max binding sites, and association of MYC with the *Bax *promoter has been previously demonstrated through ChIP [68].

Gene expression profiling of pancreatic β-cells identified a strong and rapid induction of the DNA-damage response pathway (Table 2). A large increase in expression was detected after 8 hours of MYC activation for *Rad51 *and *H2afx*, previously identified MYC targets whose protein products are involved in homologous recombination and repair of DNA [70,71]. Also, significant up-regulation of *Hus1 *and *Rad1 *- whose products form the 9-1-1 DNA-damage sensing machinery with Rad9 - indicated that oncogenic stress through deregulation of MYC resulted in the induction of DNA double strand breaks. The gene for the DNA-damage mediator Atr was also found to be up-regulated by 2-fold throughout the time course from 4 hours, and the associated checkpoint kinases *Chk1 *and *Chk2 *were up-regulated 2-fold from 8 hours (Table 2 and Figure 3C). The gene for the double-strand break-related DNA-damage mediator Atm showed 2-fold down-regulation at 8 hours, although it has been shown that Atm plays a significant role in MYC-induced apoptosis in lymphomagenesis in mice [72]. The genes *Cdkn2a *(p16/p19^Arf^) [2,62] and *Atr *[73] are previously categorized MYC target genes. The checkpoint kinase genes *Chk1 *and *Chk2 *have not been previously classified as MYC target genes, indicating that the observed changes in expression may occur downstream of Atr. Interestingly, as mentioned previously, expression of *Cdkn2c *(p18^Ink4c^), which inhibits Cdk4/6, was significantly up-regulated (6-fold) in β-cells at 16 hrs. p18^Ink4c ^interacts directly with Atm/Atr leading to an increase in p53 protein, and promotion of growth arrest and/or cell death, suggesting a link between p18 ^Ink4c ^and the DNA-damage response pathways [74]. Comparatively few DNA-damage related genes showed significant changes in expression for SBK, although we cannot exclude the possibility that DNA-damage proteins may be regulated by MYC through non-transcriptional means (e.g. protein phosphorylation).

Of particular interest was the survival factor gene *Igf1*, whose product has been shown to inhibit MYC-induced apoptosis *in vitro *by blocking Cytochrome c release from the mitochondria through the Akt1 tumour suppressor pathway [75]. *Igf1 *was up-regulated throughout much of the time-course for SBK, and this was confirmed using qRT-PCR (Figure 3C). Consistent with this finding, *Akt1 *was up-regulated 3-fold at 8 hours and 2-fold at 32 hours. A similar pattern was observed for *Akt2*, a second member of the Akt protein kinase family. This strongly supports the view that activation of the Igf-Akt pathway may contribute to suppression of MYC-related apoptosis. Interestingly, *Igf1*, *Akt1 *and *Akt2 *were up-regulated at later time points in β-cells. It is therefore possible that the Igf-Akt pathway is activated in both tissues, but that the β-cells may be able to bypass these signals.

The pro-apoptotic Bcl2 family member *Pmaip1 *(Noxa) and the gene encoding inhibitor of growth protein p47^Ing3 ^(*Ing3*) both showed a decrease in expression of 2-fold at 8 hours in SBK, but exhibited no change in β-cells (Table 2). Over-expression of p47^Ing3 ^results in cell cycle arrest and apoptosis in cancer cell lines [76], and reduced expression and loss of heterozygosity of the p47^Ing3 ^locus have been found to be associated with human head and neck squamous carcinomas [77]. Also seen were several members of the TNF superfamily of apoptosis-inducing receptors. *Tnfrsf12a*, which has been found to be involved in inducing both apoptosis and angiogenesis [78,79], showed an increase in expression in the skin of 3-fold at 8 hours. *Tnfrsf4*, whose product has been implicated in promoting survival through induction of Bcl2 and Bcl_XL _expression in CD4 T cells [80], similarly showed an increase in expression of 2-fold at 8 hours.

### A pro-angiogenic response in suprabasal epidermis

The placental growth factor gene, *Pgf¸ *showed a marked increase from 8 hours throughout the time course for the skin (Table 3). In contrast, a 2-fold down-regulation was detected for the pancreas throughout much of the time-course. Pgf is a member of the vascular endothelial growth factor (VEGF) family, and has been shown to result in increased numbers, branching and size of dermal blood vessels following over-expression in basal keratinocytes of adult mice [81]. This may indicate a role in the development of neovasculature seen following MYC activation in the SBK [28]. *Vegfa*, a further member of the VEGF family important in angiogenesis, was down-regulated in the pancreas but showed up-regulation in the skin at 8 hours. In contrast, *Vegfc*, important in the growth of lymphatic vessels, showed down-regulation in the skin at early time points, but was up-regulated almost 4-fold in the pancreas at the later 32 hour time-point. *Vegfb *showed a 2-fold increase from 16 hours in pancreas compared to no change in the skin. These results indicate a transcriptional response upon MYC activation for genes relating to neovascular growth.

**Table 3:** Differentiation, angiogenesis, and cellular adhesion genes responsive to MYC-ER^TAM ^activation

		Pancreatic β-Cells				Suprabasal Keratinocytes				
		
Gene Symbol	RefSeq	4 Hours	8 Hours	16 Hours	32 Hours	4 Hours	8 Hours	16 Hours	32 Hours	Myc-response p-value
										
**Differentiation**										
										
adamts1	D67076	0.56	0.72	2.12*	1.10	1.42	1.33	3.29**	3.42**	2.89E-02
creg1	BC027426	2.08**	2.12**	1.60**	2.16**	0.69*	0.85	0.98	0.92	3.68E-02
csta	C89521	1.12	1.13	1.04	0.88	0.49**	1.04	0.34**	0.52**	1.28E-02
eg666231	C81193	1.26*	1.85**	2.40**	1.98**	0.82*	2.76**	1.19	2.29**	1.94E-02
gck	L38990	0.59*	0.14**	2.46**	0.81	1.14	1.41	1.27	1.29	2.31E-03
ins1	NM_008386	0.18*	0.42	9.94**	10.18**	1.55	1.14	1.46	1.26	1.80E-03
ins2	NM_008387	0.17*	0.36	8.70**	12.66**	0.57	1.36	1.00	0.77	1.03E-03
ivl	AV009441	0.99	1.24	1.06	1.03	0.35**	0.59**	0.22**	0.82	4.32E-02
krt1	NM_008473	1.49*	0.99	1.17	1.02	0.48**	4.02**	0.70*	0.86	4.05E-02
krt14	BC011074	1.26	1.43*	1.16	0.73*	0.89	2.02**	0.96	0.98	1.93E-02
krt14	BC011074	1.12	1.00	1.16	0.98	0.91	2.17**	0.89	1.04	2.87E-02
mnx1	NM_019944	0.57*	0.40**	0.88	1.40	0.88	1.03	1.06	0.96	1.07E-02
nkx2-2	NM_010919	0.65	0.66*	1.55	0.43**	0.91	0.76	0.97	0.65*	2.22E-02
nkx6-1	AF357883	0.53*	0.32**	2.29**	1.13	0.91	0.94	1.09	0.98	4.29E-02
odc1	S64539	1.72**	2.29**	1.83**	1.82**	1.11	1.62**	0.99	1.81**	2.43E-02
pcnt	NM_008787	2.70**	3.79**	5.22**	4.18**	1.14	1.89**	1.1	1.82**	1.37E-02
pcsk2	BB357975	2.24**	0.56*	0.69*	1.08	1.24	1.12	1.04	0.83	1.33E-02
pcsk2	AI839700	0.98	0.48**	0.71**	0.93	1.03	1.08	0.89	0.89	3.31E-02
pcsk2	NM_008792	0.42**	0.39**	1.74**	2.59**	0.94	1.01	1.10	0.96	4.15E-02
pdx1	AK020261	0.74*	0.20**	0.71*	0.47**	1.96**	1.11	1.30	0.95	3.47E-02
pdx1	AK020261	0.60**	0.37**	0.70*	0.62**	1.11	1.08	1.20	0.92	4.14E-02
plec1	BM210485	1.31	0.49*	1.25	3.10**	0.53	1.38	1.04	2.87**	2.42E-02
plec1	BM232239	1.20	0.77	1.35	2.46**	3.23**	4.56**	2.82**	0.59*	2.09E-03
slc2a2	NM_031197	0.84*	0.43**	0.94	0.76**	0.74**	1.00	1.19*	0.91	2.91E-02
sprr1b	NM_009265	1.14	1.13	1.10	0.76	0.24**	0.33**	0.67	0.27**	1.27E-02
tgm2	BC016492	1.21	0.74	8.27**	2.63**	0.83	1.89*	2.02**	4.55**	1.54E-03
tgm2	AW321975	1.15	1.47*	3.90**	3.73**	1.09	0.79	1.89**	1.03	1.54E-02
tgm2	BB550124	1.37*	1.36*	3.44**	2.75**	1.26	0.70*	1.91**	1.04	1.58E-02
tgm2	BB041811	1.31*	1.52**	3.05**	3.22**	1.01	0.65**	2.30**	1.22	2.44E-02
										
**Cell Adhesion**										
										
itga7	NM_008398	0.85	0.86	1.40*	2.86**	0.50*	1.67*	0.76	2.48**	2.14E-02
itga9	NM_133721	1.16	1.81*	1.14	1.15	0.82	1.45	1.12	2.11**	3.56E-02
itgb2	NM_008404	1.27	1.00	2.18**	3.99**	1.27	2.81**	0.65	4.16**	8.45E-03
itgb3	AV352983	0.83	0.88	1.30*	0.76*	1.12	1.54**	1.94**	2.33**	4.47E-03
itgb6	NM_021359	0.96	0.97	0.95	1.04	1.11	2.22**	1.33**	1.55**	3.45E-02
kl	BQ175355	0.81	0.35**	0.92	0.86	1.04	1.09	0.87	0.89	2.13E-02
										
**Immune Response**										
									
h2-aa	AV086906	3.97*	1.76	0.65	4.15*	0.27*	15.37**	0.48	0.92	6.05E-03
h2-d1	M34962	2.81	2.55*	1.40	5.07**	0.28*	39.62**	0.53	1.31	2.31E-03
h2-l	M86502	2.32	1.79	1.83	6.04*	0.24	65.12**	0.8	1.04	5.14E-03
h2-l	M69068	2.71	1.98	1.72	6.72**	0.32	48.04**	0.64	0.91	4.38E-02
il1r1	NM_008362	0.53**	0.41**	1.56*	0.62*	0.57**	1.29	1.02	1.33	5.95E-03
il6ra	X53802	0.35**	0.45*	0.87	0.42**	1.40	0.77	1.06	0.70	4.70E-03
										
**Angiogenesis**										
										
cst7	NM_009977	0.92	0.90	1.22*	1.10	1.04	1.99**	0.95	1.45**	1.79E-02
pgf	NM_008827	0.50**	0.39**	1.03	0.43**	1.03	2.29**	2.24**	6.64**	2.52E-02
vegfa	NM_009505	0.83*	0.52**	0.99	0.87	1.19	2.00**	1.16	0.73*	4.27E-02
vegfa	U50279	1.16	0.32**	1.12	0.65	0.86	1.79*	1.16	1.02	4.07E-02
vegfb	U48800	1.34	1.21	2.29**	2.08**	0.57*	1.72*	0.88	1.33	2.16E-02
vegfc	BB089170	1.18	0.69**	0.60**	1.72**	0.76*	0.40**	0.81	0.77*	2.21E-02
vegfc	AW228853	1.25	0.70	0.80	3.87**	0.38**	0.51	0.79	1.82	3.87E-02
										
**Extracellular Matrix**										
									
klk1	BC010754	0.74	1.44	1.12	1.74	1.95	11.58**	20.38**	244.79**	1.13E-04
klk1	BC010754	1.31	1.36	1.01	1.93	2.72*	16.74**	13.93**	255.71**	4.94E-05
klk21	AB039276	0.63**	0.87	1.24	1.12	1.11	3.36**	4.16**	17.78**	4.06E-07
klk24	NM_010643	0.60*	0.81	1.24	1.15	1.64*	4.99**	12.91**	18.13**	3.33E-06
klk27	NM_020268	0.78	1.03	1.15	1.1	1.55	12.63**	45.42**	128.74**	5.94E-08
klk9	M17962	0.86	1.16	1.18	0.99	1.67	5.97**	9.03**	45.34**	1.11E-04
pgf	NM_008827	0.50**	0.39**	1.03	0.43**	1.03	2.29**	2.24**	6.64**	3.14E-02
thbs2	NM_011581	0.98	0.95	1.2	1.00	1.14	1.44**	1.90**	3.92**	2.56E-07
thbs2	BB233297	0.87	1.17	0.83	1.21	2.05**	2.86**	4.37**	3.14**	1.17E-03
thbs2	NM_011581	0.99	0.94	0.99	1.05	2.23**	2.60**	5.86**	3.40**	4.14E-04

Selected MYC-responsive genes relating to differentiation, angiogenesis and cellular adhesion. 'MYC-response p-value' is the p-value identified for the highest-order interaction of the MYC activation variable and represents the significance of this term within the selected model. Flags represent contrast p-values comparing 4OHT-treated and vehicle-treated samples at specific time points ('*', p ≤ 0.05; '**' p ≤ 0.01). Cells are colour-coded based on a detected fold-change greater than 1.5-fold (red, up-regulated; blue, down-regulated).

### Activation of MYC leads to loss of differentiation

Activation of MYC is often associated with loss of differentiation of cells and has been found to block terminal differentiation in a variety of cell types [11,82,83]. Activation of MYC in the pancreatic β-cells resulted in down-regulation of *Ins1 *and *Ins2 *by > 5-fold at 4 hours, indicating acute loss of Insulin production within a short time period following MYC-deregulation. However, the expression levels of both subsequently increased dramatically showing almost 10-fold up-regulation from 16 hours (Table 3). Since the islet area used for RNA extraction was roughly identical for each sample, this indicates acute increase in the levels of Insulin production within the β-cells in response to continuous MYC activation, and not in response to an increase in β-cell mass. Although perhaps a paradox at first glance, this response may be the result of a positive feedback loop due to increased Insulin release into the bloodstream immediately following MYC activation as we have previously shown [23]. Observation of transcript levels of both mRNAs at a later time point (72 hours; data not shown) indicated that this period of high Insulin production is limited, as gene expression subsequently returned to lower levels indicative of loss of β-cell differentiation. This indicates a short window within the first two days where a balance is struck between an increased rate of Insulin production and the simultaneous loss of cells due to MYC-driven apoptosis.

Members of the homeodomain transcription factor family, Pdx1, Pax4, Hb9, Nkx2.2 and Nkx6.1, are essential in pancreatic development [84]. Probe-sets for the pancreatic and duodenal homeobox gene *Pdx1 *(or *Ipf1*), whose product activates transcription of the Insulin gene as well as a number of genes involved in glucose-sensing [85], showed a significant loss (> 3-fold) in expression at 8 hours following MYC activation (as well as potentially significant loss in expression < 2-fold at all other time points), which correlated with the early reduction seen in Insulin production (Table 3). The transcription factor gene *Nkx6.1*, whose product is essential for β-cell differentiation [86], also showed significant down-regulation in the early stages of MYC activation, although expression of this gene was shown to increase during later stages. Further Pdx1-regulated genes *Slc2a2 *(Glut2; previously classed as a putative MYC target gene) and *Gck *(Glucokinase), both part of the glucose-sensing machinery and involved in membrane transport and phosphorylation of glucose respectively, also followed similar expression profiles (Table 3). Gu *et al. *identified a group of 217 mature islet-specific genes [87], 63 of which showed a significant response following MYC activation in our analysis (Additional file 1, Table S6). The majority of these showed down-regulation at early time points. These data indicate a loss in β-cell differentiation and carbohydrate metabolism function following activation of MYC.

Activation of MYC in the SBK resulted in significant changes in expression of many genes relating to differentiation (Additional file 1, Table S5). In particular, it was clear that the primary result of MYC activation on these genes was down-regulation, with 199 differentiation-related genes showing a loss of expression compared to only 112 showing up-regulation. In addition to these general differentiation markers, activation of MYC led to down-regulation of several key keratinocyte differentiation genes (Table 3). Most notable was a significant 3-fold decrease in expression for the Involucrin gene, *Ivl*, after only 4 hours that was maintained throughout much of the time course. Involucrin is a key factor in the progression of differentiation of keratinocytes which works together with its substrate transglutaminase to cross-link with membrane proteins and provide support to the cell [88].

Cornifin (*Sprr1b*), a precursor to the epidermal cornified envelope, is a further keratinocyte differentiation markers that has been shown to affect the number of distinct layers of differentiated keratinocytes [89,90]. As with *Ivl*, *Sprr1b *showed consistently marked down-regulation throughout the time course (Table 3). Similarly, Cystatin A (*Csta*), a cysteine protease inhibitor that is found expressed in keratinocytes as the precursor of the cornified cell envelope [91,92], showed > 2-fold down-regulation throughout much of the time course. Up-regulation of α and β integrin genes such as *Itga7*, *Itga9*, *Itgb2*, *Itgb3 *and *Itgb6*, particularly at later time points, suggests altered adhesion of SBK with surrounding cells and the extracellular matrix following MYC activation (Table 3). Also, expression changes were detected for several Keratin genes, including up-regulation of the suprabasal-specific *Krt1 *and the basal-specific *Krt14 *at 8 hours (Table 3), which encode fibrous structural proteins in keratinocytes.

Previous findings from the Watt group in which MYC is targeted to basal keratinocytes has, in contrast, shown that activation of MYC promotes an increase in the number of proliferating keratinocytes concomitant with promotion of terminal differentiation of epidermal stem cells [89]. In the microarray experiment of Frye *et al. *[90], gene expression signatures were compared between whole skin sections from 4OHT-treated K14-MYC-ER^TAM ^mice and 4OHT-treated WT mice to identify cellular networks involved in the promotion of terminal differentiation of epidermal stem cells at the expense of hair lineages [90]. Activation of MYC for 4 days was sufficient to cause hyperproliferation of the interfollicular epidermis, with increased expression of genes relating to both proliferation (e.g. *Ki67*, *Krt17*, *Krt6*, *Cdc2*, *Cdc20*) and interfollicular epidermis differentiation (Filaggrin and Cornifin/Sprp1). In contrast, expression of genes relating to early G1/S-phase cell cycle progression are more prominent when MYC is activated in SBK, whilst keratinocyte-specific differentiation genes such as *Sprp1 *generally show down-regulation. This suggests that activation of MYC suppresses differentiation in keratinocytes already undergoing terminal differentiation (i.e. SBK) to allow proliferation.

In a more recent study, low, intermediate, or high levels of MYC activity were induced in basal keratinocytes, and showed that MYC drives proliferation at all levels, but at high levels MYC promotes keratinocyte differentiation [93]. Promotion of differentiation may therefore act as a fail-safe mechanism against neoplastic conversion of epidermal stem cells.

### The transcriptome fingerprint of MYC activation is varied in distinct tissues: pancreatic islets and skin

Clustering of genes whose expression changed significantly due to the joint effects of MYC activation and the tissue type identified genes whose expression profiles were correlated across the time courses for the two tissues, indicating possible co-regulation and functional similarity. Genes relating to DNA replication, DNA-damage checkpoint and cell cycle showed tight co-regulation, and showed significantly increased expression in the pancreatic β-cells. The expression profiles of mini-chromosome maintenance (MCM) deficient genes *Mcm2*, *Mcm5*, *Mcm6 *and *Mcm7*, whose products make up part of the MCM complex involved in DNA unwinding [94], the *Cdt1 *gene, whose product is involved in association of the MCM complex with chromatin, and various helicase related genes were found to be closely related, indicated co-expression of genes relating primarily to DNA replication. Close correlation with these genes was also seen in the pancreas for DNA-damage checkpoint related genes *Atr *and *Chk1*. These genes showed consistently high levels of expression in the β-cells, indicating a key role for DNA-damage response and repair in MYC-induced apoptosis. Conversely, no significant change was detected for these genes in the SBK.

In SBK, close correlation was detected for genes involved in proteolysis, particularly members of the Kallikrein family of serine proteases. Expression of Kallikrein genes (*Klk1*, *Klk9*, *Klk21*, *Klk24 *and *Klk27*) was found to increase significantly within 8 hours following activation of MYC, and continued to increase dramatically throughout the time course (Table 3 and Figure 1C). Klk1 and Klk9 have previously been found to be expressed throughout the epidermis of normal human skin [95], and play a role in degradation of the extracellular matrix (ECM) and loss of squamous cells during differentiation. Deregulated expression of Kallikreins has also been implicated in many cancer types [96-101]. The mouse Kallikreins Klk21, Klk24 and Klk27 have also been shown to be functionally active within the testes, both in degradation of the extra-cellular matrix and initiation of survival through degradation of Igf1bp3 [102-104]. Kallikrein proteins have been associated with angiogenesis. Growth of new vasculature is facilitated by regulating the tissue micro-environment, particularly through degradation of the extra-cellular matrix often via activation of matrix metalloproteinases (MMPs). This indicates a functional relationship between Kallikreins and the vascularisation-related gene, placental growth factor *Pgf *(a member of the vascular endothelial growth factor family of angiogenesis-related genes) and the Thrombospondin 2 gene *Thbs2 *(an anti-angiogenic factor which has been linked to extra-cellular matrix remodelling through modulation of matrix metalloproteinases [105]) which showed close correlation with these genes. Given the prominent angiogenic phenotype that develops in these pre-cancerous skin lesions following MYC activation [33], it is reasonable to speculate here that Kallikreins and Pgf may be involved.

## Conclusions

Deregulation or over-expression of the MYC onco-protein is a frequent feature of human cancers, which attests to the pleiotropic role that ectopic MYC plays in cellular function. However, oncogenic MYC can also trigger activation of intrinsic tumour suppressor programs such as p19^Arf^/p53, which serve to limit propagation of such harmful cells by inducing growth arrest or apoptosis. While much is known about the mechanisms of MYC functions, the pathways responsible for deciding the ultimate fate of the cell between 'life' (uncontrolled proliferation) and 'death' (i.e. apoptosis) *in vivo *are not yet clear. The decision for a cell to become apoptotic depends on the complex interactions of many pro- and anti-apoptotic factors. Different tissues may exhibit varying levels of these factors ultimately determining the fate of a cell. However, tissue-specific environmental characteristics can also affect the interaction between these factors, having a decisive effect on cell fate. The MYC-ER^TAM ^transgenic mouse model allows controlled over-expression of MYC in distinct adult tissues, SBK (epidermis) and pancreatic β-cells, enabling tracking of early changes downstream of aberrant MYC activity. In this study, high-throughput transcriptional profiling was used to identify transcriptional events that may provide clues to explain the disparity in the phenotypic response to MYC activation in SBK (proliferation and survival) and pancreatic β-cells (proliferation and apoptosis).

Whilst expression of genes relating to multiple cellular functions (eg. metabolic, transcriptional, transportational, ribosome biogenesis) is common to both tissues, expression of genes involved in DNA-damage and replication is enriched to β-cells. Consistent with early increased expression of Ki67 in β-cells [27], key G_1_/S-phase genes (e.g. those encoding Cyclin D2, Cyclin E and Cdk4) showed early changes in expression, whilst G_2_/M-phase genes (e.g. those encoding Cyclin A and Cyclin B) showed changes at later time points. Down-regulation of the cyclin-dependent kinase inhibitor (CDKI) *Cdkn1b *(p27^Kip1^) gene in β-cells was evident as previously shown in other cell types [48]. The CDKI *Cdkn2c *(p18^Ink4c^), which inhibits Cdk4 and Cdk6, was also down-regulated, consistent with G_1_/S phase transition. The short time period over which these changes were seen supports the idea that MYC-induced cell cycle progression occurs through direct activation of key cell cycle genes such as *Ccnd1, Ccnd2 *and *Ccne2*.

In SBK, although there were changes in cell cycle related genes, the response was much less prominent in comparison with β-cells. Gene expression changes included up-regulation of *Krt6a*, *Pcna, Ccnd3, Cdk4*, and the key G1/S phase cyclin gene *Ccnd2*, accompanied by significant down-regulation of the CDKI gene *Cdkn1b *(p27^Kip1^) throughout the time course. However, further cell cycle related genes (e.g. *Ccna1*, *Ccne2 *and *Cdk2*) did not appear to be measurably affected by MYC activation in SBKs. It is possible that SBK may at some point struggle to progress through the G_2_/M phase, which may be indicated by down-regulation of *Ccnb1 *and *Cdc2a*, whose products are essential in later cell cycle stages. This contrasts with pancreatic β-cells, in which we found both *Ccnb1 *and *Cdc2a *significantly up-regulated. The less pronounced cell cycle response in skin may be due to the relatively low proportion of keratinocytes that are responsive to Myc-induced proliferation at these early time-points. It has previously been shown that there is only a narrow window when very early suprabasal cells that have migrated out of the basal layer, are capable of responding to Myc-induced cell cycle entry [28,105]. The more differentiated keratinocytes of the granular layer are refractory to the proliferative influence of MYC.

Gene expression profiling of the pancreatic β-cells identified the DNA-damage checkpoint pathway as a likely route by which MYC mediates apoptosis in this system, leading to downstream activation of p53 and Bax-mediated release of Cytochrome c from the mitochondria. In addition, close correlation was seen in the pancreas for DNA-damage checkpoint related genes *Atr *and *Chk1*, and members of the MCM complex, *Mcm2, Mcm5 *and *Mcm7*. The change in expression for these genes following MYC activation was consistently high in the β-cells, suggesting a key role for DNA-damage response and repair in MYC-induced apoptosis. Conversely, no significant change was detected for these genes in the SBK. Recent evidence strongly suggests that deregulated MYC induces rapid accumulation of DNA-damage, which is the primary cause of activation of the Atm/Atr-dependant checkpoint [106,107]. The study of Dominguez-Sola *et al. *(2007), in particular, suggests that the pleiotropic role of MYC is due not only to transcriptional regulation of downstream genes, but also due to direct interactions with the DNA. This study also showed that over-expression of MYC results in DNA-damage and checkpoint activation. Consequently, the activation of DNA-damage response (DDR) pathways results in ultimate destruction of the offending cell.

Whilst direct control of these genes by MYC is not discernible from these data, it is clear that MYC deregulation induces a transcriptional response representative of cells undergoing DNA repair, which is a likely explanation for activation of the intrinsic apoptotic pathway. These results fit with the hypothesis that deregulated MYC leads to oncogenic stress and DNA-damage, although whether this is direct or indirect remains to be seen.

With regard to events downstream of the DDR, we found an increase in expression of genes associated with activation of mitochondrial outer membrane permeabilisation in pancreatic β-cells. These included the p53-target *Bax*, and the pro-apoptotic mitochondrial factors *Cycs *and *Endog*, which may indicate replenishment of proteins lost from the mitochondria during apoptotic signalling. In addition, increased expression of the tumour suppressor *Cdkn2a *(p19^Arf^) was also detected, which may have been involved in stabilization of the p53 tumour suppressor by inhibition of Mdm2, or in promoting cell cycle arrest prior to apoptosis together with up-regulation of the p53-target gene *Cdkn1a *(p21^Cip1^). In contrast, SBK showed no change in these genes, but instead a decrease in expression of the pro-apoptotic Bcl2 family member *Pmaip1 *(Noxa) and the inhibitor of growth gene *Ing3 *(p47^Ing3^). Previous work has linked over-expression of p47^Ing3 ^with apoptosis [76], and reduced expression with human head and neck squamous carcinomas [77]. SBK may be protected from apoptosis *in vivo *by the Igf1-Akt survival pathway. Of particular interest was the early induction of *Igf1*, *Akt1 *and *Akt2 *in the SBK, and the tight correlation seen between the three. Expression of these three genes is also seen at later time points in the pancreas, indicating that the Igf1-Akt pathway may be activated in both tissues but is somehow bypassed in the β-cells.

One key difference between the two systems appears to be the presence of members of the Kallikrein serine protease family, which were dramatically up-regulated throughout the time-course for SBK. Kallikrein proteins have been linked to many forms of cancer, and of particular note is their role in the Igf1-Akt survival pathway. Klk1, Klk21, Klk24 and Klk27 have been linked to Igf1-regulated tumour survival through degradation of the Igf binding protein Igfbp3 in humans. This prevents Igfbp3 from antagonizing Igf1-Igf1r interactions, allowing Igf1 to bind to its receptor and initiate survival via the Akt pathway [102-104,108]. Interestingly, the highest expression in a similar study from Frye *et al. *using a basal keratinocyte model for MYC activation [90] was for the brain and skin protease gene *Bssp1*, also known as Kallikrein 6 (*Klk6*). This gene remained low in WT treated mice, but was significantly increased following MYC expression (129-fold up-regulated). In both models, MYC activation drives vastly increased Kallikrein expression, so it is possible that these proteins play similar roles in cell survival in both systems. Increased expression of Kallikrein genes in SBK following MYC activation may therefore create an environment that favours survival over apoptosis.

In addition to the increased cell proliferation in both tissues, our data indicate a loss of differentiation in both pancreatic β-cells and SBK in response to activation of MYC. In pancreatic β-cells, we found down-regulation of genes that are essential in pancreatic development, such as *Pdx1 *and *Nkx6.1*, as well as genes involved in glucose-sensing such as *Slc2a2 *(Glut2) and *Gck*, both putative MYC-targets. In SBK, many significant changes were detected for genes relating to keratinocyte differentiation that generally pointed to a loss or delay in differentiation - an observation that was previously noted in this mouse model [28]. Interestingly, activation of MYC in the basal layer of the epidermis actually promotes keratinocyte differentiation [93], a mechanism which is thought to prevent epidermal stem cells from becoming cancerous. One possible explanation for the contrasting behaviour in suprabasal epidermis, is that SBKs are post-mitotic and have already entered a terminal differentiation process. Subsequent activation of MYC in early SBK may promote loss of differentiation to enable cell cycle entry. Since MYC-activated SBKs are already migrating upwards towards the skin surface, they are less likely to pose a cancer risk to the host given that they will ultimately be sloughed off as previously shown [28].

We have previously shown that MYC activation in SBK results in a prominent angiogenic phenotype [28]. From our microarray data, potential candidates that may promote such a response include *Pgf*, a member of the VEGF family, which was highly up-regulated in skin but in fact down-regulated in pancreas. We also found *Vegfa *up-regulated in skin but not in pancreas. Interestingly *Vegfc*, which is important in growth of lymphatic vessels, was down-regulated in skin but up-regulated in pancreas. However, given that prominent angiogenesis is not detected in the skin until 3-4 days following MYC activation [28], it is possible that the short time course considered here is too early to identify a transcriptional response in all relevant genes relating to vascularisation. Kallikrein proteins have also been implicated in facilitating angiogenesis via degradation of the cellular matrix [109] and our data showed co-regulation of *Pgf *and Kallikrein genes. These data suggest that the local tissue microenvironment in SBK that promote angiogenic growth may be linked to survival pathways that protect the cells against apoptosis.

In summary, activation of MYC results in cell growth, loss of differentiation and cell cycle entry in both β-cells and SBK *in vivo*. Apoptosis, which is confined to β-cells, involves a combination of a DNA-damage response and downstream activation of pro-apoptotic signalling pathways, including Cdc2a and p19^Arf^/p53, and downstream targets. Conversely, avoidance of apoptosis in SBK may result primarily from the activation of key anti-apoptotic signalling pathways, particularly those involved in the Igf1-Akt pathway, and induction of an angiogenic response (*Pgf*, *Vegfa *and *Klks*). A contributory role for intrinsic resistance to the induction of DNA-damage and the p19^Arf ^tumour suppressor pathway by MYC in SBK is also possible. A possible mechanism whereby tissue-specific environmental factors may influence cell fate following MYC deregulation has also been proposed, hypothesising that the decision to live or die may relate to the local tissue-specific microenvironment. However, this remains speculation as the approach taken here gives an insight into only one aspect of the changes occurring within the cell in response to MYC deregulation. Much remains to be learnt from analysis of protein-level changes, post-translational modifications, or epigenetic modifications of DNA. This study has identified several new lines of investigation for future analysis into the dual roles of MYC in apoptosis and survival. It is hoped that such studies will prove fruitful and provide further insight into the complex role of this enigmatic protein.

## Methods

### Tissue Sample Preparation

Transgenic mice expressing switchable pIns-MYC-ER^TAM ^in pancreatic β-cells or inv-MYC-ER^TAM ^in SBK have been previously characterized and described [27,28]. 8-12 week old male mice were treated with 4OHT or vehicle for 4, 8, 16 or 32 hours. Triplicate samples were collected for each time point for each of the four conditions; pIns-MYC-ER^TAM ^4OHT treated/MYC-ER^TAM ^active ("Panc T"), pIns-MYC-ER^TAM ^vehicle treated/MYC inactive ("Panc U"), Inv-MYC-ER^TAM ^4OHT treated/MYC active ("Skin T"), Inv-MYC-ER^TAM ^vehicle treated/MYC inactive ("Skin U"). All mice were housed and treated in accordance with protocols and regulations sanctioned by the Home Office under the Animals Act of 1986.

### RNA Isolation and Microarray Hybridization

A modified LCM protocol was devised to preserve RNA integrity. Fresh frozen pancreas sections were cut to a thickness of 15 μm, bound to a MMI MembraneSlide (Molecular Machines and Industries, Rockledge, FL) and fixed in ice-cold 100% ethanol for 2 mins. Sections were stained briefly (10 secs) with a 1% Toluidine Blue dye in 100%. Stained sections were dehydrated in 2 changes of 100% ethanol and 2 changes of xylene for 1 minute each, airdried for 2 minutes and finally left in a vacuum dessicator for 5 minutes before transportation to the laser capture platform. The SL μCut LCM system (Molecular Machines and Industries, Rockledge, FL) was used to isolate islets of Langerhans from surrounding exocrine tissue. RNA was collected using the RNA Microkit protocol (Qiagen, Valencia, CA). The laser capture procedure was repeated on freshly cut pancreas sections to collect a total area of islet cells equal to roughly 1.5 × 10^6 ^μm^2 ^for each sample. Due to the thinness of murine suprabasal epidermis, isolation of sufficient good quality RNA for microarray hybridization from LCM of SBK proved difficult, a problem also noted by Agar et al. [110]. RNA was instead collected from 5 fresh frozen skin sections (20 μm) collected across several levels of the tissue. A modified version of the Affymetrix GeneChip 2-cycle target labelling *in vitro *transcription (IVT) protocol was used, incorporating double volumes of polyA controls and reagents in the first round cRNA synthesis stage to increase the yield. 10 μg double-amplified biotin-labelled cRNA were hybridized to Affymetrix MOE430 2.0 GeneChips (Affymetrix, Santa Clara, CA) as described in the Affymetrix GeneChip Expression Analysis Technical Manual.

### Data Analysis

Data were normalized across all samples at the probe-level using GC-RMA [111]. Analysis of gene expression data was performed using the Bioconductor microarray analysis packages in R [112] and GeneSpring GX 7.3.1 (Agilent, Santa Clara, CA). 4OHT-treated samples were normalized to their respective vehicle-treated counterparts to ensure that normalized values corresponded to the fold-change in gene expression due to activation of MYC-ER^TAM^. Removal of control and non-responsive probes identified 12,349 probe sets. The custom R package *Envisage *was used to identify probe-sets showing significant differential expression after MYC-ER^TAM ^activation, across time, and between pancreas and skin tissue. p-values were calculated for each model term and their interactions, and were corrected for multiple testing [113]. Only the highest-order interaction p-value was considered for genes where the selected model contains multiple terms relating to the MYC-ER^TAM ^activation state. Contrast p-values were calculated for each condition by applying an unpaired t-test comparing 4OHT-treated samples with vehicle-treated samples within each of the 8 groups (4 hrs, 8 hrs, 16 hrs and 32 hrs for skin or pancreas). Significant early-changing (≥ 2-fold within 8 hours) probe sets were compared with known gene-ontologies using the DAVID functional annotation tool [29]. Quality threshold (QT) partitional clustering [114] was used to identify genes showing similar expression profiles, using the Pearson cross-correlation coefficient with a minimum correlation of 0.9 and a minimum cluster size of 14.

### Quantitative Real-Time reverse transcriptase PCR

TaqMan qRT-PCR was performed on original total RNA samples for genes of interest. 20 ng total RNA was reverse transcribed to cDNA using a high-capacity cDNA reverse transcription kit (Applied Biosystems, Foster City, CA). cDNA transcripts were pre-amplified prior to the qRT-PCR reaction in a multiplexed reaction using TaqMan preAmp mastermix (Applied Biosystems, Foster City, CA), with pooled TaqMan qRT-PCR assays (Applied Biosystems, Foster City, CA) at a concentration of 0.2X in 1X TE buffer. qRT-PCR was performed using an ABI Prism 7000 scanner (Applied Biosystems, Foster City, CA), with an 18s rRNA endogenous control probe (Applied Biosystems, Foster City, CA). qRT-PCR was performed for skin and pancreas 4OHT- and vehicle-treated RNA samples for early time-points 4 hrs and 8 hrs, and for the later 32 hrs time-point. As with microarray analysis, quantitative measures of gene expression upon MYC-activation were calculated by comparing vehicle- and 4OHT-treated samples directly for each condition.

### Immunohistochemical Staining

Frozen sections were cut to 10 μm and fixed with 4% paraformaldehyde (PFA) at room temperature (RT) for 10 mins, washed in PBS for 5 mins, and incubated at RT in a humidifying temperature for 30 mins in 10% bovine serum albumin (BSA). Pancreas sections were double stained for Ki67 and insulin, or caspase 3 and insulin. Sections were incubated at 4°C overnight in primary antibodies diluted in 1% BSA: Insulin, 1:100 (guinea pig; DAKO, Denmark); Ki67, 1:200, (rabbit; Novocastra, UK); Caspase 3, 1:200 (rabbit; Cell Signalling Technology Inc., MA). Sections were washed twice in phosphate-buffered saline (PBS) with 0.1% tween (PBSt) for 5 mins each and incubated for 30 mins at RT in a humidifying chamber with secondary antibodies FITC (Vektor Co., Germany) or ALEXA633 (Invitrogen, Carlsbad, CA) diluted in 1% BSA (1:200). Skin tissue sections were sequentially stained for Keratin 1 and Ki67, or Keratin 1 and Caspase 3. Sections were incubated for 1 hour in Ki67 or Caspase 3 primary antibodies diluted in 1% BSA (1:200). Sections were washed twice with PBSt for 5 mins each and incubated for 30 mins at RT in a humidifying chamber with FITC anti-rabbit secondary antibodies diluted in 1% BSA (1:200). Finally, samples were washed in two changes of PBSt for 5 mins each. This cycle was repeated using Keratin 1 primary antibodies (rabbit; BabCo, CA) diluted in 1% BSA (1:100) and ALEXA633 anti-rabbit secondary antibodies diluted in 1% BSA (1:200). Slides were mounted in Vectashield (Vector Labs, Burlingame, CA) mounting medium containing 4',6-diamidino-2-phenylindole (DAPI) and viewed using a Leica Sp2 confocal microscope (Leica, Wetzlar, Germany).

## List of abbreviations

4OHT: 4-Hydroxytamoxifen; 2a-cRNA: Double-amplified biotin-labelled complementary RNA; ANOVA: Analysis of Variance; BSA: Bovine Serum Albumin; CDK: Cyclin-Dependent Kinase; CDKI: Cyclin-Dependent Kinase Inhibitor; ChIP: Chromatin Immunoprecipitation; cRNA: Complementary RNA; DAPI: 4',6-diamidino-2-phenylindole; DNA: Deoxyribonucleic Acid; DPX: Distyrene/Plasticizer/Xylene; ECM: Extra Cellular Matrix; FDR: False Discovery Rate; FWER: Family Wise Error Rate; GCOS: Genechip Operating System; GC-RMA: Genechip Robust Multi-array Average; GO: Gene Ontology; IVT: *In-Vitro *Transcription; LCM: Laser Capture Microdissection; MCM: Minichromosome Maintenance; MMP: Matrix Metalloproteinase; OCT: Optimal Cutting Temperature; PBS: Phosphate Buffered Saline; PFA: Paraformaldehyde; QC: Quality Control; qRT-PCR: Quantitative Real-Time Reverse Transcriptase Polymerase Chain Reaction; RIN: RNA Integrity Number; RM: RIP7-Bcl_XL_/pIns-MYC-ER^TAM^; RNA: Ribonucleic Acide; RT: Room Temperature; SAGE: Serial Analysis of Gene Expression; SBK: Skin Basal Keratinocytes; VEGF: Vascular Endothelial Growth Factor

## Competing interests

At the time this work was carried out, EH was Senior Scientist in Bioinformatics (EMEA) for Agilent Technologies, the developers of the GeneSpring GX analysis suite. The remaining authors declare that they have no competing interests.

## Authors' contributions

SR conducted the major part of this study, including designing the experiment, preparing samples, developing the *Envisage *tools, running all statistical analyses, performing qRT-PCR and immunohistochemical staining, and drafting the manuscript. LW performed all microarray hybridisations. HB assisted with experiment design, microarray hybridisation and manuscript preparation. HT and EH assisted with development of *Envisage *and other statistical analyses. SP and MK conceived of the study, coordinated the study design, and helped to finalise the manuscript. All authors read and approved the final manuscript.

### Data Access

The microarray data described in this experiment are available from the ArrayExpress repository (http://www.ebi.ac.uk/arrayexpress/) with accession number E-MEXP-2952. *Envisage *code is available upon request.

## Supplementary Material

Additional file 1 **Supplementary gene expression tables and gene set enrichment analysis results**. Click here for file
