# Supplementary material for: Deciphering c-MYC-regulated genes in two distinct tissues
Source: BMC Genomics. 2011 Sep 30;12:476. doi: 10.1186/1471-2164-12-476 (PMC3206520; doi:10.1186/1471-2164-12-476)
Supplement: Additional file 1 — Supplementary gene expression tables and gene set enrichment analysis results. [file 1471-2164-12-476-S1.DOC]

**Table S1: MYC target genes showing significant change in expression within 8 hours of MYC-ER^TAM^ activation.**

Genes present in the MYC Target Gene database showing a significant change in expression within 8 hours following activation of MYC-ER^TAM^. ‘MYC-response p-value’ is the p-value identified for the highest-order interaction of the MYC activation variable and represents the significance of this term within the selected model. Flags represent contrast p-values comparing 4OHT-treated and vehicle-treated samples at specific time points (‘*’, p ≤ 0.05; ‘**’ p ≤ 0.01). Cells are colour-coded based on a detected fold-change greater than 1.5-fold (red, up-regulated; blue, down-regulated).

|  |  | **Pancreatic β-cells** | | | |  | **Suprabasal Keratinocytes** | | | | **Myc-response p-value** |
| --- | --- | --- | --- | --- | --- | --- | --- | --- | --- | --- | --- |
| **Gene Symbol** | **RefSeq** | **4 hrs** | **8 hrs** | **16 hrs** | **32 hrs** |  | **4 hrs** | **8 hrs** | **16 hrs** | **32 hrs** |
| acpp | AI324033 | 0.95 | 1.08 | 1.17 | 0.96 |  | 0.40** | 0.87 | 0.50** | 0.94 | 3.56E-02 |
| acpp | BB008092 | 1.02 | 0.97 | 1.16 | 1 |  | 1.11 | 0.44** | 0.97 | 0.55** | 3.91E-03 |
| acsl1 | BI413218 | 1.21 | 1.01 | 0.8 | 1.17 |  | 0.69 | 2.50** | 1.03 | 1.27 | 1.37E-02 |
| acsl1 | BC006692 | 2.11** | 1.77** | 1.07 | 1.97** |  | 0.8 | 0.67* | 1.13 | 0.68** | 1.33E-04 |
| acsl1 | BI413218 | 1.25 | 0.99 | 0.87 | 2.44** |  | 0.9 | 2.17** | 1.02 | 1.07 | 3.79E-02 |
| adc | BE946956 | 0.52** | 0.49** | 1.18 | 0.92 |  | 0.75** | 0.62** | 0.81 | 0.89 | 7.93E-04 |
| adk | BB555069 | 4.00** | 0.98 | 0.50** | 1.18 |  | 0.79 | 0.45** | 0.84 | 1.86** | 3.11E-03 |
| aga | AV052058 | 0.54* | 0.89 | 1.28 | 0.99 |  | 0.8 | 1.02 | 0.85 | 0.66 | 3.60E-02 |
| agt | AK018763 | 0.98 | 0.28** | 1.64** | 2.21** |  | 1.17 | 1.06 | 1.14 | 2.08** | 4.69E-02 |
| ak2 | NM_016895 | 2.65** | 2.29** | 1.71** | 1.75** |  | 0.97 | 0.87 | 1.24 | 1.04 | 2.26E-02 |
| ak2 | NM_016895 | 0.99 | 0.88 | 3.11** | 5.94** |  | 0.58** | 2.64** | 0.89 | 1.84** | 8.98E-03 |
| akt1 | NM_009652 | 1.01 | 0.97 | 1.71** | 2.31** |  | 0.82 | 3.23** | 0.95 | 2.29** | 4.43E-02 |
| akt2 | NM_007434 | 1 | 1.05 | 0.98 | 1.13 |  | 1.27 | 2.21** | 1.02 | 2.26** | 7.85E-03 |
| alas2 | M63244 | 0.88 | 0.96 | 1.18 | 0.8 |  | 1.33 | 2.18** | 0.96 | 1.37* | 1.02E-02 |
| alg5 | BC027160 | 1.18 | 0.68 | 3.34** | 3.15** |  | 1.07 | 2.47** | 0.97 | 2.09** | 3.46E-03 |
| amd1 | NM_009665 | 2.23** | 1.51** | 1.60** | 2.15** |  | 1.09 | 1.11 | 0.92 | 0.99 | 1.46E-04 |
| anxa6 | AK013026 | 1.01 | 0.66 | 0.37** | 0.96 |  | 0.96 | 7.30** | 0.75 | 6.96** | 2.47E-02 |
| apex1 | NM_009687 | 1.39* | 1.12 | 2.03** | 2.07** |  | 0.97 | 2.07** | 1.23 | 1.84** | 4.61E-02 |
| apex1 | AV100480 | 2.02** | 1.21 | 1.67** | 2.34** |  | 0.91 | 1.19 | 1.12 | 1.1 | 6.82E-03 |
| apex1 | AV263745 | 2.21** | 1.67** | 1.38** | 1.73** |  | 0.96 | 1.30** | 1.18 | 1.14 | 3.87E-05 |
| ar | BB148302 | 0.48** | 0.75 | 0.95 | 0.86 |  | 1.04 | 0.58* | 0.97 | 0.68 | 2.83E-03 |
| arf4 | BI653265 | 1.14 | 0.57 | 2.29* | 1.4 |  | 0.72 | 2.93** | 1.03 | 3.02** | 1.71E-02 |
| arpc4 | BG145444 | 0.99 | 1.07 | 1.33** | 1.45** |  | 1.18 | 1.99** | 1.29* | 1.74** | 1.65E-03 |
| ars2 | BC019117 | 1.21 | 1.40* | 2.11** | 3.34** |  | 0.66** | 2.15** | 0.89 | 2.42** | 3.64E-02 |
| arvcf | BE947943 | 1.33 | 1.88** | 0.99 | 1.02 |  | 0.72 | 3.50** | 0.84 | 1.58 | 3.47E-02 |
| atf6 | BB129063 | 0.74 | 0.20** | 0.78 | 0.96 |  | 0.8 | 1.53* | 1.04 | 1.14 | 9.54E-03 |
| atp5s | NM_026536 | 0.72* | 0.32** | 1.45* | 0.95 |  | 1.07 | 0.83 | 0.98 | 0.59** | 1.59E-02 |
| atr | AF236887 | 2.25** | 3.79** | 2.71** | 2.76** |  | 1.17 | 1.17 | 1.44* | 1.53** | 3.46E-02 |
| atrx | BB425841 | 1.57** | 1.98** | 1.55** | 1.78** |  | 0.9 | 0.93 | 1.27 | 1.15 | 3.95E-02 |
| baz1b | BB253608 | 0.93 | 0.66 | 2.51** | 2.06** |  | 1.32 | 3.17** | 1.37 | 1.35 | 3.06E-02 |
| bcat1 | X17502 | 1.08 | 3.35** | 0.93 | 3.67** |  | 1.11 | 2.50** | 1.47 | 1.52 | 7.50E-03 |
| bckdhb | AW047304 | 0.97 | 0.60** | 2.79** | 2.06** |  | 0.65** | 2.06** | 0.96 | 1.14 | 4.65E-02 |
| bcl11b | BM117007 | 1.28 | 0.85 | 0.79 | 1.03 |  | 1.01 | 0.40** | 0.96 | 0.53** | 2.18E-02 |
| bcl2 | BI664467 | 1.23 | 2.27** | 1 | 1.3 |  | 1.31 | 1.21 | 0.99 | 0.93 | 7.79E-03 |
| birc6 | BG071331 | 2.37** | 0.57* | 0.8 | 0.94 |  | 1.39 | 0.92 | 0.87 | 1.26 | 1.87E-02 |
| blm | NM_007550 | 1.18 | 2.92** | 5.96** | 2.80** |  | 0.71* | 0.68 | 1.13 | 1.01 | 1.38E-03 |
| blmh | AV149605 | 2.19** | 2.95** | 1.11 | 1.53* |  | 0.94 | 1.05 | 1.05 | 1.08 | 6.83E-03 |
| bmp4 | NM_007554 | 0.79 | 0.92 | 1.18 | 1.17 |  | 0.38** | 0.76 | 0.50** | 0.69* | 2.66E-02 |
| bop1 | BM213936 | 2.22** | 2.70** | 3.10** | 2.85** |  | 0.8 | 2.42** | 1.14 | 2.44** | 7.54E-03 |
| btg1 | L16846 | 0.47** | 0.35** | 1.14 | 0.56** |  | 0.65* | 0.79 | 0.75 | 0.97 | 2.21E-03 |
| btg1 | AW322026 | 0.61** | 0.40** | 0.93 | 0.73** |  | 0.78* | 0.71** | 0.82 | 0.76* | 2.36E-03 |
| bzw1 | AV144956 | 0.84* | 2.10** | 1.35** | 1.07 |  | 0.83* | 1.25** | 1 | 1.27** | 1.23E-02 |
| bzw1 | AV144956 | 1.75** | 0.73 | 0.91 | 1.17 |  | 1.78** | 2.24** | 1.78** | 1.16 | 1.02E-03 |
| c1qb | NM_009777 | 0.84 | 0.56 | 4.41** | 3.54** |  | 1.69 | 6.50** | 0.38* | 6.73** | 1.93E-02 |
| c1qb | BM938277 | 0.95 | 0.72 | 1.16 | 1.01 |  | 1.07 | 2.30** | 1.04 | 6.43** | 4.73E-02 |
| c1qbp | NM_007573 | 1.60* | 1.03 | 4.69** | 6.06** |  | 0.92 | 3.40** | 0.92 | 1.99** | 1.93E-02 |
| c1qbp | AV108824 | 1.97** | 2.85** | 1.03 | 1.67** |  | 1.70** | 1.15 | 1.32* | 0.92 | 1.45E-04 |
| c3 | K02782 | 0.94 | 0.52* | 2.28* | 2.22** |  | 0.83 | 1.01 | 0.40** | 0.87 | 5.19E-03 |
| cad | AK010453 | 2.94** | 1.09 | 2.99** | 4.82** |  | 1.33 | 1.80** | 1.22 | 3.64** | 5.24E-04 |
| cad | AK010453 | 2.02** | 2.28** | 2.28** | 1.92** |  | 1.08 | 1.84** | 1.16 | 1.49* | 1.12E-03 |
| cast | AB026997 | 0.67** | 0.43** | 1.16 | 1.05 |  | 0.81* | 0.51** | 1.05 | 0.82 | 4.80E-02 |
| cat | BM247599 | 0.9 | 0.75 | 1.08 | 1.1 |  | 0.72 | 0.42** | 0.43** | 1.01 | 3.80E-02 |
| ccna2 | X75483 | 0.96 | 4.52** | 10.49** | 3.25** |  | 0.81 | 0.67 | 1.39 | 0.97 | 2.05E-02 |
| ccnd1 | NM_007631 | 1.86** | 2.09** | 0.88 | 1.45 |  | 1.13 | 1.31 | 1.79* | 1.04 | 3.73E-02 |
| ccnd1 | NM_007631 | 1.69 | 2.38 | 1.41 | 2.27* |  | 1.05 | 1.33 | 1.72 | 1.26 | 4.55E-02 |
| ccnd1 | NM_007631 | 3.41** | 2.74* | 2.03* | 2.05 |  | 1.35 | 0.99 | 1.54 | 1.81 | 2.24E-02 |
| ccnd2 | NM_009829 | 2.06** | 2.04** | 1.27 | 2.30** |  | 0.9 | 0.84 | 1.29 | 0.84 | 2.62E-02 |
| ccnd2 | AK007904 | 1.99** | 0.64* | 1.88** | 2.62** |  | 1.66* | 2.57** | 1 | 2.13** | 1.48E-02 |
| ccnd3 | NM_007632 | 1.26* | 0.84 | 1.54** | 1.2 |  | 0.81* | 2.58** | 0.84 | 3.33** | 1.21E-02 |
| ccnd3 | BB167641 | 1.08 | 1.23 | 0.85 | 0.91 |  | 1.13 | 0.32** | 0.98 | 0.53** | 3.64E-03 |
| ccnd3 | BB043576 | 1.07 | 1.03 | 0.75* | 1 |  | 0.61** | 0.48** | 0.72** | 0.82 | 1.10E-03 |
| ccne1 | NM_007633 | 1.82** | 3.37** | 7.02** | 1.71* |  | 0.94 | 1.35 | 1.15 | 1.82** | 1.28E-03 |
| ccne1 | BB293079 | 1.83** | 6.68** | 1.87** | 2.04** |  | 0.85 | 1.50* | 1.16 | 1.29 | 2.38E-02 |
| ccng2 | U95826 | 0.39** | 0.41** | 1.44 | 0.60* |  | 0.77 | 0.59** | 0.8 | 0.93 | 4.65E-04 |
| cd47 | BM942688 | 1.06 | 1.02 | 0.58** | 0.65* |  | 1.01 | 0.27** | 0.66* | 0.35** | 7.65E-03 |
| cd9 | NM_007657 | 0.43** | 0.50* | 2.15* | 1.89* |  | 1.17 | 2.07* | 0.6 | 1.11 | 3.56E-02 |
| cdc25a | C76119 | 2.71** | 2.23** | 2.09** | 3.90** |  | 1.11 | 0.46** | 1.07 | 0.78 | 1.84E-03 |
| cdc25a | C76119 | 2.17** | 1.73* | 2.03** | 2.10** |  | 1.02 | 1.14 | 1.1 | 1.21 | 2.45E-02 |
| cdc6 | NM_011799 | 2.18* | 7.25** | 6.48** | 6.44** |  | 0.68 | 0.62 | 1.16 | 1.25 | 4.41E-03 |
| cdk4 | NM_009870 | 1.16 | 0.92 | 8.41** | 1.11 |  | 1.32 | 12.27** | 0.74 | 1.62 | 1.02E-02 |
| cdk4 | NM_009870 | 1.21 | 0.74 | 5.31** | 1.5 |  | 1.23 | 11.30** | 0.92 | 1.48 | 1.45E-02 |
| cdk4 | NM_009870 | 1.2 | 0.88 | 5.03** | 1.3 |  | 1.37 | 11.17** | 0.72 | 1.37 | 4.33E-02 |
| cdkn1a | AK007630 | 1.57** | 2.05** | 1.71** | 1.24 |  | 0.96 | 1.44* | 0.99 | 0.9 | 5.01E-03 |
| cdkn1b | NM_009875 | 1.03 | 0.19** | 0.71 | 0.95 |  | 0.23** | 0.42* | 0.48* | 0.89 | 1.02E-02 |
| cdkn1b | BB354528 | 0.98 | 0.47** | 0.63* | 0.65** |  | 1.02 | 0.41** | 1.03 | 0.68* | 8.84E-03 |
| cdkn2a | NM_009877 | 2.17** | 1.79** | 1.57** | 2.61** |  | 1.21 | 1.07 | 1.07 | 0.87 | 8.43E-03 |
| cdkn2b | AF059567 | 0.43** | 0.29** | 0.76 | 0.47** |  | 0.58** | 0.86 | 0.67* | 0.72 | 1.20E-03 |
| cdt1 | AF477481 | 4.81** | 15.83** | 28.12** | 6.15** |  | 0.7 | 0.64 | 1.45 | 1.22 | 4.22E-02 |
| cdt1 | AF477481 | 3.31** | 3.50** | 11.38** | 8.04** |  | 0.71 | 0.92 | 0.76 | 1.79 | 1.28E-02 |
| cept1 | BB361936 | 1 | 0.46** | 1.05 | 0.93 |  | 0.62* | 0.45** | 0.74 | 1.23 | 3.80E-02 |
| cfp | BB800282 | 1.01 | 0.85 | 0.74 | 0.8 |  | 1.29 | 6.53** | 0.30** | 8.03** | 1.21E-03 |
| ciao1 | AK004129 | 0.89 | 0.92 | 1.55* | 1.58 |  | 1.26 | 3.70** | 1.07 | 1.31 | 3.56E-02 |
| cks2 | NM_025415 | 0.92 | 3.03** | 10.08** | 4.80** |  | 0.51** | 0.76 | 1.27 | 0.71 | 3.31E-02 |
| cks2 | NM_025415 | 0.98 | 2.33** | 8.92** | 2.34** |  | 0.63* | 0.92 | 0.93 | 1.32 | 3.45E-02 |
| cnbp | BM237919 | 2.02** | 1.32 | 0.84 | 2.01** |  | 1.16 | 1.51 | 0.86 | 0.91 | 3.69E-02 |
| cnot4 | BB756908 | 1.73** | 0.51** | 1.12 | 1.36** |  | 0.83 | 1.3 | 1 | 1.32* | 2.03E-02 |
| cox7c | AA190297 | 2.39** | 0.81 | 0.92 | 0.99 |  | 0.86 | 0.70* | 0.86 | 1.28 | 2.30E-02 |
| cryab | NM_009964 | 1.06 | 1.13 | 1.08 | 1.47 |  | 0.96 | 4.03** | 1.09 | 1.80** | 8.20E-03 |
| csda | BB779100 | 2.24** | 1.79** | 1.24* | 2.78** |  | 1.11 | 0.79* | 1.12 | 0.73** | 1.04E-02 |
| csda | AV216648 | 4.14** | 2.01* | 2.10** | 6.29** |  | 0.56* | 1 | 0.69 | 0.98 | 4.51E-02 |
| ctgf | NM_010217 | 0.54* | 1.15 | 0.62 | 1.22 |  | 1.26 | 0.55* | 0.84 | 0.68 | 3.37E-03 |
| cth | BC019483 | 3.27** | 0.95 | 2.02* | 1.33 |  | 0.95 | 1.13 | 0.56* | 1.38 | 3.46E-02 |
| ctps | NM_016748 | 2.26** | 2.70** | 1.81** | 1.83** |  | 1.81** | 1.50** | 1.80** | 1 | 4.02E-02 |
| ctsb | M14222 | 0.82 | 0.45* | 3.36** | 9.23** |  | 1.82 | 4.09** | 0.52 | 2.84** | 4.23E-02 |
| cul5 | BB702110 | 2.49** | 0.83 | 1.70** | 0.93 |  | 1.01 | 0.75 | 1.11 | 1.11 | 4.08E-02 |
| cyba | AK018713 | 1.33 | 0.86 | 3.06** | 9.59** |  | 1.44 | 4.50** | 0.44** | 3.15** | 8.01E-03 |
| cycs | NM_007808 | 1.17* | 1.98** | 1.55** | 2.05** |  | 0.83* | 1 | 1.17 | 1.05 | 1.64E-03 |
| cyfip2 | AK005148 | 0.77 | 0.51** | 2.27** | 1.02 |  | 0.37** | 0.43** | 0.56** | 0.92 | 9.16E-03 |
| cyp51 | NM_020010 | 2.39** | 1.76* | 1.01 | 2.27** |  | 0.7 | 0.75 | 1.01 | 0.68* | 2.07E-02 |
| cyp51 | NM_020010 | 2.22** | 1.59* | 1.14 | 1.64* |  | 0.88 | 1.05 | 0.88 | 0.66* | 1.28E-02 |
| dap | BC024876 | 0.77* | 0.48** | 1.14 | 0.72* |  | 0.81 | 0.84 | 0.78 | 1.13 | 4.10E-03 |
| dbi | NM_007830 | 0.89 | 0.79 | 2.86** | 1.19 |  | 0.73* | 3.32** | 0.70* | 1.90** | 2.61E-02 |
| dck | BB030204 | 1.54* | 2.91** | 1.73** | 1.56* |  | 1 | 0.62* | 1.15 | 1.50* | 2.13E-02 |
| ddx10 | AK019495 | 2.41** | 2.21** | 2.32** | 3.63** |  | 1.61* | 1.39 | 1.21 | 0.92 | 4.89E-03 |
| ddx18 | BM233297 | 0.98 | 0.49** | 1.07 | 1.03 |  | 0.9 | 1.03 | 1.03 | 1.1 | 1.09E-02 |
| ddx21 | BM246099 | 1.82** | 2.03** | 1.16 | 1.66* |  | 1 | 1.21 | 1.28 | 0.87 | 4.64E-03 |
| deaf1 | NM_016874 | 2.03* | 0.9 | 1.79** | 3.22** |  | 0.91 | 2.24** | 0.55* | 1.32 | 4.16E-02 |
| dgkz | BC014860 | 1.06 | 1.37* | 3.20** | 3.47** |  | 0.29** | 1.47* | 0.60** | 1.94** | 8.67E-03 |
| dhfr | NM_010049 | 1.28 | 2.97** | 3.31** | 1.85** |  | 0.60* | 0.99 | 1.02 | 1.26 | 5.79E-03 |
| dhodh | NM_020046 | 2.09** | 1.68** | 1.35* | 2.26** |  | 0.73** | 1 | 0.87 | 1.14 | 3.16E-02 |
| dhodh | NM_020046 | 2.76** | 1.25* | 2.10** | 1.88** |  | 0.86 | 1.45** | 0.91 | 1.34** | 2.04E-02 |
| dkc1 | BG068512 | 2.23** | 1.85** | 1.80** | 2.08** |  | 1.02 | 1.28 | 0.95 | 2.13** | 1.21E-03 |
| dkc1 | BB779105 | 4.08** | 2.39** | 0.64 | 3.58** |  | 0.79 | 0.47** | 1.70* | 0.56* | 5.85E-03 |
| dleu2 | BB812902 | 0.73 | 0.24** | 0.94 | 1.02 |  | 0.97 | 0.74 | 0.62* | 0.81 | 1.81E-02 |
| dnaja2 | C77509 | 0.76 | 0.97 | 1.71** | 1.92** |  | 1.22 | 2.25** | 1.26 | 1.80** | 3.46E-02 |
| drg1 | BM506525 | 1.54** | 2.23** | 1.21 | 1.35** |  | 1.02 | 1.44** | 1.13 | 0.91 | 1.18E-02 |
| drg1 | AV127994 | 1.80** | 2.27** | 1.42** | 1.53** |  | 0.73* | 1.18 | 1.46** | 0.99 | 2.23E-04 |
| drg1 | NM_007879 | 1.18 | 1.37* | 4.20** | 2.41** |  | 0.82 | 2.50** | 0.94 | 1.78** | 2.45E-02 |
| dusp2 | L11330 | 2.68** | 1.29 | 1.3 | 1.89** |  | 1.48 | 1.09 | 0.95 | 1.33 | 5.51E-03 |
| e2f3 | BQ176318 | 2.47** | 1.42 | 1.12 | 1.58* |  | 1.35 | 0.83 | 1.74** | 0.74 | 4.65E-02 |
| ebf1 | BB364548 | 1 | 0.99 | 0.99 | 1.05 |  | 1.5 | 2.61** | 0.86 | 1.99** | 4.00E-02 |
| ebf1 | BB038386 | 1.03 | 1.01 | 0.49** | 1.50* |  | 1.63** | 2.40** | 0.74 | 2.80** | 7.19E-03 |
| ebna1bp2 | AK007491 | 2.41** | 0.96 | 2.55** | 2.68** |  | 1.08 | 1.45 | 1.56 | 1.38 | 3.53E-02 |
| eef1e1 | NM_025380 | 3.22** | 2.14** | 7.58** | 7.88** |  | 1.4 | 3.42** | 1.52* | 1.63** | 4.05E-02 |
| egf | NM_010113 | 1.05 | 0.79 | 0.92 | 1.38* |  | 1.62** | 0.47** | 0.91 | 0.84 | 1.28E-02 |
| egfr | AV369812 | 0.74* | 0.46** | 0.86 | 0.99 |  | 1.13 | 0.39** | 1.33* | 0.47** | 2.96E-02 |
| egr3 | AV346607 | 1.06 | 1.24 | 2.20** | 1.15 |  | 0.68** | 0.39** | 0.84 | 0.40** | 2.32E-02 |
| eif4a1 | BI656407 | 0.87 | 0.85 | 1.62* | 1.38 |  | 0.99 | 3.54** | 1.05 | 2.81** | 1.91E-02 |
| eif4a1 | BF320852 | 1.28* | 2.03** | 0.89 | 1.14 |  | 0.62** | 1.51** | 0.96 | 1.59** | 1.85E-03 |
| eif4e | BB406487 | 2.88** | 2.15** | 1.62** | 2.04** |  | 1.32** | 1.27** | 1.47** | 1.33** | 1.61E-05 |
| eif4e | BB406487 | 1.57* | 2.41** | 3.08** | 4.72** |  | 1.22 | 2.97** | 1.57* | 1.96** | 2.42E-04 |
| eif4e | BB406487 | 2.54** | 1.59 | 0.78 | 1.98* |  | 2.07* | 0.7 | 1.9 | 0.97 | 1.00E-02 |
| eif4ebp2 | NM_010124 | 1.15 | 1.09 | 1.1 | 1.85** |  | 2.52** | 1.28 | 1.54 | 1.19 | 8.54E-03 |
| eif5a | BF384094 | 1.38** | 1.44** | 1.75** | 1.46* |  | 1.42* | 2.53** | 1.36* | 1.51** | 5.95E-03 |
| elovl1 | BB339820 | 1.26 | 1.09 | 0.73 | 0.88 |  | 0.44** | 0.46** | 0.70* | 0.51** | 1.78E-02 |
| eno1 | NM_023119 | 1.33 | 1.36 | 6.33** | 4.74** |  | 1.15 | 2.06** | 1.09 | 1.31 | 1.35E-02 |
| eno2 | NM_013509 | 0.67** | 0.32** | 0.66** | 0.33** |  | 1.03 | 1.24 | 0.86 | 1.09 | 3.82E-04 |
| etf1 | C77379 | 1.91** | 2.23** | 1.30** | 1.76** |  | 1.21* | 0.70** | 1.33** | 0.99 | 1.03E-04 |
| etf1 | BC013717 | 2.23** | 1.45* | 2.48** | 3.17** |  | 0.7 | 1.28 | 1.29 | 1.77** | 4.76E-03 |
| exo1 | BE986864 | 0.95 | 2.17** | 3.81** | 2.36** |  | 0.94 | 1.12 | 1.28 | 1.2 | 1.20E-02 |
| exosc5 | NM_138586 | 1.97** | 1.18 | 4.30** | 1.91** |  | 0.8 | 2.01** | 1.17 | 1.57* | 1.62E-02 |
| f2r | AV024285 | 0.93 | 0.64** | 0.78 | 1.02 |  | 0.8 | 0.43** | 0.93 | 0.35** | 1.33E-02 |
| f2r | BQ173958 | 1.21 | 0.83 | 0.71 | 1.13 |  | 0.64* | 0.42** | 0.91 | 0.52** | 1.33E-02 |
| fabp5 | BC002008 | 3.42** | 2.43** | 2.54** | 4.77** |  | 0.98 | 0.88 | 0.99 | 0.78* | 2.45E-02 |
| fabp5 | BC002008 | 2.49** | 1.67* | 5.80** | 4.33** |  | 0.9 | 2.90** | 0.57* | 1.15 | 3.13E-02 |
| fads2 | NM_019699 | 0.86 | 2.05** | 2.55** | 1.23 |  | 0.91 | 1.2 | 1.05 | 1.15 | 7.18E-03 |
| fads3 | BE652876 | 1.26 | 1.17 | 0.94 | 1.01 |  | 0.88 | 2.00** | 1.16 | 1.31* | 1.52E-02 |
| fas | BG976607 | 6.55** | 6.15** | 3.85** | 11.34** |  | 0.82 | 1.22 | 1.61 | 1.35 | 3.79E-02 |
| fas | BG976607 | 3.61** | 7.00** | 1.13 | 4.43** |  | 1.33 | 0.63 | 1.35 | 0.46** | 6.32E-03 |
| fas | BG976607 | 5.08** | 12.85** | 5.07** | 10.92** |  | 0.72 | 5.14** | 0.74 | 0.48* | 1.12E-03 |
| fas | BG976607 | 2.43** | 1.78** | 2.28** | 2.50** |  | 0.83 | 1.33 | 1.09 | 0.39** | 2.21E-03 |
| fasn | AF127033 | 2.14** | 2.78** | 1.43** | 2.60** |  | 0.73** | 1.21** | 0.55** | 1.44** | 5.48E-03 |
| figf | BB359521 | 0.79* | 0.31** | 1.25 | 1.57** |  | 1.03 | 0.42** | 1.08 | 0.64** | 3.90E-02 |
| figf | BB359521 | 1.11 | 0.33** | 1.13 | 2.18** |  | 0.96 | 0.42** | 1.01 | 0.66* | 1.60E-02 |
| figf | NM_010216 | 0.83* | 0.89 | 0.92 | 1.21* |  | 0.77** | 0.48** | 0.96 | 1.59** | 3.69E-02 |
| fkbp4 | BB456860 | 1.88** | 0.64 | 3.57** | 1.93** |  | 1.63* | 2.37** | 1.03 | 1.61* | 1.12E-02 |
| fxc1 | NM_019502 | 1.27 | 2.03** | 3.49** | 4.84** |  | 0.73* | 3.13** | 0.77 | 1.98** | 1.29E-02 |
| fxc1 | BB609429 | 1.84** | 3.24** | 2.82** | 3.90** |  | 0.83 | 2.36** | 0.76 | 2.01** | 2.58E-02 |
| fxc1 | NM_019502 | 2.34** | 2.21** | 2.58** | 3.00** |  | 0.84 | 2.31** | 0.87 | 1.81** | 2.00E-02 |
| fxc1 | AK002949 | 3.88** | 1.95** | 0.77 | 1.58** |  | 1.2 | 0.94 | 1.75** | 1.01 | 1.49E-02 |
| gadd45g | AK007410 | 0.59* | 0.41** | 1.88** | 0.59** |  | 4.07** | 5.16** | 3.39** | 2.78** | 5.95E-03 |
| gal | NM_010253 | 0.96 | 0.98 | 1.27 | 1.38* |  | 0.82 | 3.49** | 2.43** | 4.56** | 1.21E-02 |
| gamt | AF015887 | 0.97 | 0.99 | 1.11 | 1.16 |  | 0.81 | 4.39** | 1.09 | 2.44** | 4.45E-02 |
| gart | NM_010256 | 2.35** | 0.72 | 7.35** | 4.56** |  | 1.33 | 1.62* | 1.24 | 1.37 | 9.02E-03 |
| gart | BB402208 | 2.42** | 2.15** | 1.72** | 2.82** |  | 0.86 | 1.28 | 1.32 | 0.88 | 1.83E-03 |
| gas1 | BB550400 | 0.88 | 0.89 | 0.47** | 0.94 |  | 1.08 | 0.41** | 0.93 | 0.39** | 2.37E-02 |
| gcn1l1 | BB309501 | 1.14 | 0.72 | 1.11 | 1.01 |  | 1.11 | 2.23** | 1.71** | 1.3 | 5.51E-03 |
| gcn1l1 | BB818980 | 1.28 | 0.84 | 1.57** | 1.77** |  | 0.84 | 2.15** | 1.15 | 1.16 | 8.62E-03 |
| gcn5l2 | NM_020004 | 1.61** | 0.59** | 2.15** | 2.14** |  | 0.95 | 2.50** | 0.96 | 1.92** | 3.63E-02 |
| gcs1 | NM_020619 | 2.00** | 1.27 | 1.78** | 2.20** |  | 1.12 | 1.84** | 1.06 | 1.89** | 1.52E-04 |
| gfer | BI901126 | 2.01** | 2.46** | 1.75* | 1.18 |  | 0.8 | 3.45** | 1.35 | 1.61* | 1.05E-04 |
| gmip | BB474868 | 7.06** | 4.17** | 2.05** | 6.46** |  | 0.81 | 1.78** | 0.78 | 1.52* | 1.95E-02 |
| gnal | BB502545 | 0.94 | 1.02 | 0.70** | 0.70** |  | 0.52** | 0.93 | 0.85* | 0.93 | 1.71E-02 |
| gnb1l | NM_023120 | 1.54* | 2.39** | 2.32** | 4.16** |  | 0.79 | 1.85** | 1.1 | 1.37* | 1.16E-02 |
| gnb1l | BG069873 | 1.69* | 2.80** | 1.06 | 4.01** |  | 0.81 | 1.34 | 1.18 | 0.50** | 2.00E-02 |
| gsk3b | BB831420 | 0.88 | 0.45** | 0.63** | 0.57** |  | 1.69** | 0.69** | 0.97 | 0.8 | 3.86E-02 |
| gtpbp4 | AI987834 | 0.82 | 1.24 | 2.00** | 1.69** |  | 0.86 | 2.19** | 1.21 | 1.27 | 3.66E-02 |
| h2afx | NM_010436 | 1.17 | 2.17** | 4.56** | 1.40** |  | 0.67** | 1.22* | 0.97 | 1.01 | 3.43E-03 |
| has2 | NM_008216 | 0.98 | 1.01 | 1.03 | 1.01 |  | 1.03 | 3.86** | 1.67** | 3.24** | 8.78E-03 |
| hbp1 | AK006835 | 0.58** | 0.50** | 0.96 | 1.53* |  | 1.38 | 1.17 | 0.71 | 1.22 | 7.67E-03 |
| hbp1 | BC026853 | 0.42** | 0.44** | 0.84 | 0.69* |  | 1.03 | 1.34 | 0.88 | 1 | 7.82E-03 |
| hdac6 | NM_010413 | 0.68* | 0.45** | 1.71** | 0.79 |  | 0.67* | 1.13 | 0.63** | 1.68** | 2.24E-02 |
| hdgf | NM_008231 | 1.29 | 1.03 | 4.17** | 2.39** |  | 0.81 | 2.34** | 0.72 | 2.22** | 1.87E-02 |
| hdgf | C80147 | 1.67* | 2.41** | 0.41** | 0.88 |  | 0.56* | 0.62* | 1.06 | 1.31 | 8.92E-03 |
| hip2 | AI551201 | 1.96** | 3.05** | 5.58** | 2.61** |  | 0.75 | 0.59** | 1.13 | 0.76 | 2.86E-03 |
| hivep2 | NM_010437 | 0.65* | 1.05 | 1.88** | 1.18 |  | 1.29 | 0.44** | 0.94 | 0.51** | 8.06E-03 |
| hmbs | AI325144 | 1.04 | 0.89 | 3.04** | 2.31** |  | 0.43** | 2.00** | 0.76 | 2.09** | 4.84E-02 |
| hmga1 | NM_016660 | 2.85** | 3.23** | 3.00** | 3.78** |  | 0.83 | 2.42** | 1.02 | 1.3 | 3.35E-02 |
| hmox1 | NM_010442 | 1.03 | 1.01 | 1.11 | 1.06 |  | 2.49** | 1.83** | 2.36** | 1.56* | 2.06E-02 |
| hnrpd | AI843818 | 2.35** | 2.24** | 2.23** | 1.07 |  | 1.27 | 0.78 | 0.98 | 1.56 | 2.68E-02 |
| hp | NM_017370 | 0.92 | 1.19* | 1.16 | 0.94 |  | 0.95 | 2.24** | 1.81** | 3.04** | 5.57E-03 |
| hspa4 | AW909503 | 2.04** | 1.51** | 0.89 | 1.78** |  | 0.93 | 0.91 | 1.04 | 1.24 | 1.48E-02 |
| hspa8 | BC006722 | 1.09 | 0.69* | 2.37** | 1.87** |  | 0.88 | 2.32** | 0.88 | 2.15** | 1.67E-02 |
| hspa8 | BC006722 | 0.87 | 0.60** | 3.05** | 2.15** |  | 1.04 | 2.47** | 0.9 | 2.47** | 2.16E-02 |
| hspa8 | AK004608 | 3.07** | 3.56** | 0.99 | 1.16 |  | 0.84 | 1.09 | 1.05 | 1.08 | 4.06E-04 |
| hspa9 | BB718260 | 1.41** | 1.98** | 1.31* | 1.2 |  | 1.03 | 1.06 | 1.25 | 0.86 | 3.18E-03 |
| hspe1 | NM_008303 | 1.02 | 1.21* | 2.25** | 1.53** |  | 1.25* | 2.43** | 1.14 | 1.07 | 3.78E-02 |
| iars | BE915338 | 2.00** | 0.84 | 2.36** | 2.51** |  | 0.95 | 2.02** | 1.06 | 1.73** | 2.28E-03 |
| idh2 | NM_008322 | 0.79 | 0.91 | 2.14** | 1.18 |  | 1.44 | 2.66** | 0.8 | 1.72** | 6.40E-03 |
| idh3b | C78231 | 1.08 | 1.02 | 1.1 | 1.02 |  | 0.53** | 2.49** | 1.07 | 1.49** | 4.16E-02 |
| ifi30 | NM_023065 | 4.86** | 3.83** | 3.23** | 4.91** |  | 1.28 | 1.96** | 0.79* | 1.40* | 4.02E-03 |
| ifi35 | BC008158 | 1.42* | 0.50** | 1.40* | 0.89 |  | 0.94 | 2.04** | 0.83 | 1.46* | 1.41E-02 |
| ifrd2 | BB540964 | 2.00** | 1.04 | 2.30** | 1.66 |  | 1.07 | 2.18** | 1.3 | 2.51** | 9.97E-03 |
| igf2 | NM_010514 | 0.50** | 0.68 | 1.01 | 0.86 |  | 0.83 | 1.01 | 1.01 | 1.02 | 1.46E-02 |
| il1rap | BE285634 | 0.9 | 0.66** | 1.15 | 1.13 |  | 0.82 | 0.41** | 0.91 | 0.41** | 9.62E-03 |
| il1rl1 | D13695 | 0.51** | 0.97 | 0.84 | 2.09** |  | 0.60** | 0.87 | 0.82 | 0.72* | 1.46E-02 |
| imp4 | AF334609 | 2.38** | 2.41** | 2.55** | 3.26** |  | 0.85 | 2.09** | 0.91 | 1.96** | 2.89E-02 |
| inhba | NM_008380 | 0.58** | 0.38** | 0.96 | 0.60** |  | 0.94 | 1.12 | 0.85 | 1.31* | 1.31E-02 |
| irf3 | BB668007 | 1.44* | 0.44** | 1.02 | 1.59** |  | 1.09 | 1.07 | 1 | 1.15 | 2.30E-02 |
| itga6 | BM935811 | 0.61* | 0.33** | 0.8 | 0.68 |  | 0.93 | 0.64* | 0.97 | 0.69 | 2.49E-02 |
| itgal | BI554446 | 0.99 | 1.15 | 0.96 | 1.21 |  | 0.84 | 2.34** | 1.74** | 2.09** | 4.47E-02 |
| kl | BQ175355 | 0.81 | 0.35** | 0.92 | 0.86 |  | 1.04 | 1.09 | 0.87 | 0.89 | 2.13E-02 |
| klk1 | BC010754 | 0.74 | 1.44 | 1.12 | 1.74 |  | 1.95 | 11.58** | 20.38** | 244.79** | 4.01E-02 |
| klk1 | BC010754 | 1.31 | 1.36 | 1.01 | 1.93 |  | 2.72* | 16.74** | 13.93** | 255.71** | 2.87E-02 |
| klk10 | NM_133712 | 0.91 | 1.07 | 1.07 | 0.93 |  | 0.39** | 1.34 | 0.48** | 0.76 | 3.91E-02 |
| kpna3 | BM213828 | 1.40* | 2.90** | 2.31** | 1.52* |  | 1.2 | 1.24 | 1.28 | 1.06 | 1.01E-03 |
| lamp2 | BB390704 | 0.99 | 0.50** | 1.18 | 0.87 |  | 0.71* | 0.99 | 0.85 | 1.11 | 4.19E-02 |
| lancl1 | AJ294535 | 1.1 | 1.84** | 3.35** | 3.06** |  | 0.77 | 2.21** | 1.16 | 2.09** | 9.85E-03 |
| lbp | NM_008489 | 0.94 | 0.94 | 1.03 | 1.23 |  | 1.09 | 3.58** | 0.95 | 2.96** | 4.25E-04 |
| ldha | NM_010699 | 1.18 | 1.12 | 3.50** | 1.80** |  | 0.85 | 2.19** | 1 | 2.07** | 5.15E-03 |
| lrp8 | BB750940 | 1.07 | 0.38** | 0.7 | 0.60** |  | 0.74 | 0.74 | 0.89 | 0.78 | 2.67E-02 |
| lsp1 | NM_019391 | 0.9 | 0.78* | 0.97 | 1.1 |  | 1.02 | 3.42** | 0.67** | 1.90** | 3.22E-02 |
| lxn | NM_016753 | 0.52** | 0.43** | 0.98 | 0.66* |  | 0.75 | 0.52** | 0.79 | 0.8 | 1.10E-04 |
| lztfl1 | NM_033322 | 0.64* | 0.44** | 1.25 | 1.05 |  | 0.86 | 0.65* | 1.05 | 0.89 | 9.13E-03 |
| lztfl1 | BB161653 | 0.95 | 0.29** | 0.87 | 0.66 |  | 1.01 | 0.51** | 0.9 | 0.84 | 1.23E-02 |
| lztfl1 | BB700884 | 0.34** | 1.17 | 1.16 | 1.29 |  | 0.67 | 0.68 | 0.88 | 1.58 | 4.20E-02 |
| lztr1 | AV094519 | 1.53* | 0.93 | 2.57** | 1.52* |  | 0.86 | 2.15** | 0.79 | 2.76** | 1.84E-03 |
| man2b1 | BC005430 | 0.75 | 0.52** | 1.68* | 0.95 |  | 0.68* | 1.76* | 0.46** | 1.78** | 1.78E-02 |
| mapk7 | NM_011841 | 0.94 | 0.40** | 1.17* | 0.99 |  | 0.78* | 0.91 | 0.77** | 1.49** | 2.74E-02 |
| mat2a | BC003451 | 2.25** | 1.80** | 1.39* | 2.23** |  | 1.74** | 2.07** | 1.35 | 1.15 | 3.40E-02 |
| mat2a | BG065061 | 2.85** | 2.73** | 1.05 | 2.01** |  | 0.62** | 2.17** | 0.68** | 1.57** | 4.77E-03 |
| mat2a | BB470596 | 2.08** | 1.63** | 0.97 | 1.88** |  | 1.02 | 1.26* | 1.03 | 1.14 | 2.78E-04 |
| mat2a | AV260654 | 2.16** | 2.11** | 1.05 | 1.66** |  | 0.79 | 1.40** | 0.94 | 1.19 | 3.45E-02 |
| mat2a | BB272730 | 2.36** | 2.14** | 0.98 | 2.05** |  | 1 | 1.36** | 1 | 1.16 | 1.85E-02 |
| mat2a | BB488978 | 2.28** | 1.76** | 1.06 | 2.05** |  | 1.11* | 1.19* | 0.89 | 1.20* | 2.54E-02 |
| mcc | BB794635 | 0.85 | 0.83 | 1.12 | 1.81** |  | 0.96 | 0.36** | 0.99 | 0.52** | 3.81E-02 |
| mcm3 | C80350 | 3.79** | 3.12** | 11.57** | 9.49** |  | 0.73 | 1.02 | 1.09 | 2.04* | 3.50E-02 |
| mcm3 | BI658327 | 1.24 | 2.84** | 2.06** | 1.75** |  | 1.14 | 1.45** | 1.38* | 1.2 | 9.57E-03 |
| mcm4 | BC013094 | 1.63* | 2.68** | 4.14** | 2.51** |  | 0.96 | 1.08 | 0.79 | 2.40** | 2.42E-02 |
| mcm4 | BB447978 | 2.16** | 6.36** | 2.47** | 2.54** |  | 0.84 | 0.8 | 1.01 | 0.98 | 8.24E-03 |
| mcm5 | AI324988 | 3.64** | 19.17** | 5.62** | 8.96** |  | 0.68 | 0.66 | 1.6 | 1.25 | 7.65E-03 |
| mcm6 | NM_008567 | 1.46 | 3.63** | 5.64** | 5.10** |  | 0.75 | 0.87 | 1.07 | 2.21* | 2.01E-02 |
| mcm6 | BB099487 | 2.19** | 9.76** | 8.15** | 4.94** |  | 0.50** | 0.63 | 1.12 | 1.11 | 2.78E-03 |
| mcm7 | NM_008568 | 1.66** | 2.59** | 3.84** | 2.38** |  | 0.97 | 1.34* | 1.04 | 1.35* | 1.97E-02 |
| mcm7 | BB464359 | 2.66** | 4.67** | 4.70** | 4.11** |  | 0.87 | 0.84 | 1 | 1.19 | 5.04E-03 |
| mcm7 | BB407228 | 2.00** | 4.75** | 3.07** | 3.07** |  | 0.72* | 0.67* | 1.05 | 0.88 | 4.06E-03 |
| me2 | BM235734 | 1.96** | 2.05** | 0.82 | 1.42* |  | 1.01 | 0.88 | 1.04 | 1.38 | 1.24E-03 |
| mecr | BB310008 | 2.86** | 1.92** | 0.74 | 1 |  | 0.92 | 1.37* | 1.05 | 0.82 | 2.65E-02 |
| meis1 | BG070088 | 0.81* | 0.49** | 1.18 | 1.51** |  | 1.09 | 0.93 | 0.98 | 0.68** | 1.44E-02 |
| mest | AW555393 | 0.83 | 0.89 | 0.57 | 2.05* |  | 1.22 | 2.35* | 1.7 | 0.96 | 1.26E-03 |
| mfng | NM_008595 | 1.21 | 1.28 | 2.10** | 2.62** |  | 0.79 | 2.76** | 0.74 | 2.89** | 2.45E-02 |
| mgat2 | AI481328 | 1.72 | 0.95 | 4.68** | 3.38** |  | 0.45* | 1.44 | 0.79 | 2.13* | 1.20E-02 |
| mgea5 | NM_023799 | 0.76 | 0.97 | 1.32 | 0.63* |  | 1.07 | 0.18** | 1.43 | 0.9 | 7.48E-03 |
| mitf | BB763517 | 0.49* | 0.34** | 0.82 | 0.91 |  | 0.88 | 0.79 | 0.95 | 0.59 | 1.72E-02 |
| mki67 | X82786 | 0.71 | 2.10* | 14.79** | 1.64* |  | 0.94 | 0.54* | 1.90** | 0.57* | 7.55E-03 |
| mki67ip | AY030275 | 1.63** | 1.41* | 2.23** | 2.80** |  | 0.93 | 2.90** | 1.40* | 1.73** | 5.00E-02 |
| mkrn1 | BQ176661 | 0.54** | 0.28** | 1.60* | 0.74 |  | 0.81 | 1.3 | 0.64* | 1.32 | 7.73E-03 |
| mkrn1 | BE133749 | 0.60** | 1.97** | 1.73** | 0.93 |  | 0.60** | 1.77** | 0.92 | 1.49** | 1.65E-02 |
| mmp16 | BB378819 | 2.08** | 2.53** | 1.78** | 1.09 |  | 1.15 | 0.84 | 1.11 | 0.78 | 3.23E-02 |
| mpo | NM_010824 | 0.94 | 1.16 | 1.02 | 0.93 |  | 0.43** | 2.04* | 0.25** | 0.18** | 2.94E-02 |
| mpp6 | AF199010 | 2.12** | 1.27 | 2.38** | 3.25** |  | 1.32* | 1.37* | 1.58** | 1.18 | 6.41E-03 |
| mrpl23 | NM_011288 | 1.13 | 0.82 | 2.43** | 1.38* |  | 1.2 | 2.23** | 0.8 | 0.99 | 1.36E-02 |
| mst1 | NM_008243 | 0.86 | 1.07 | 1.07 | 1.1 |  | 0.34** | 1.08 | 0.78* | 0.71** | 4.75E-02 |
| mt2 | AA796766 | 0.79 | 0.20** | 3.79** | 0.46* |  | 1.55 | 1.84 | 0.7 | 0.95 | 1.76E-02 |
| mtf1 | BE980297 | 0.96 | 0.27** | 1.15 | 1.04 |  | 1.04 | 0.82* | 1.02 | 0.97 | 6.13E-03 |
| mthfd1 | NM_138745 | 1.57* | 1.38 | 1.17 | 1.63* |  | 1.16 | 0.49** | 1.46 | 0.74 | 4.15E-02 |
| mthfd2 | BG076333 | 0.95 | 0.69 | 6.13** | 2.17** |  | 1.37 | 2.62** | 1.63 | 1.99* | 4.88E-02 |
| mthfr | BG069750 | 0.74 | 0.42** | 0.97 | 0.84 |  | 0.84 | 1.06 | 0.57* | 1.06 | 3.01E-02 |
| mxi1 | BB825697 | 2.24** | 1.44** | 3.24** | 3.09** |  | 1 | 0.91 | 0.93 | 1.02 | 2.17E-02 |
| mybbp1a | AW228043 | 1.5 | 0.50** | 1.09 | 2.34** |  | 1.16 | 1.71* | 1.05 | 1.38 | 5.57E-03 |
| myc | BC006728 | 1.1 | 0.99 | 1.12 | 1.02 |  | 0.32** | 0.65* | 0.53** | 0.99 | 1.97E-02 |
| naga | BC021631 | 0.54* | 0.39** | 1.3 | 0.61* |  | 0.57* | 1.56 | 0.65 | 1.27 | 8.06E-03 |
| nap1l1 | BG064031 | 2.00** | 1.49** | 2.05** | 2.08** |  | 1.03 | 1.32** | 1.28* | 1.34** | 1.49E-04 |
| nap1l1 | BG064031 | 0.97 | 0.98 | 1.80** | 1.33 |  | 0.51** | 1.73** | 1.12 | 1.70** | 4.54E-02 |
| ncam1 | BB698413 | 0.52** | 0.26** | 1.09 | 0.45** |  | 1.07 | 0.87 | 1.26 | 0.89 | 1.56E-02 |
| ncl | BF118393 | 1.21 | 0.39* | 2.67* | 4.03** |  | 2.32 | 2.16 | 3.00* | 1.44 | 1.39E-02 |
| ncl | BF118393 | 2.47** | 2.86** | 1.57* | 1.77* |  | 0.98 | 1.15 | 2.31** | 0.74 | 5.10E-03 |
| ndrg1 | AI987929 | 0.49** | 0.37** | 0.45** | 0.58* |  | 0.98 | 0.64* | 1.04 | 1.03 | 2.15E-02 |
| ndrg1 | AI987929 | 0.66* | 0.29** | 0.61* | 0.75 |  | 1.27 | 1.03 | 0.91 | 1.59** | 4.95E-02 |
| ndufb4 | BG968046 | 0.89 | 1.28 | 3.52** | 1.66* |  | 1.38 | 2.63** | 1.02 | 1.80* | 4.09E-02 |
| ndufs6 | C88200 | 0.83 | 1.01 | 2.65** | 1.56** |  | 1.07 | 2.24** | 1.03 | 1.18 | 2.38E-02 |
| nfil3 | AY061760 | 0.39** | 1.22 | 1.87** | 0.84 |  | 0.9 | 1.57* | 0.62* | 1.83** | 2.40E-02 |
| nit1 | BM121177 | 0.57** | 0.41** | 1.37 | 0.72 |  | 0.71 | 0.9 | 0.85 | 1.1 | 3.37E-03 |
| nit1 | BM121177 | 0.72* | 0.48** | 1.42* | 0.56** |  | 0.52** | 1.08 | 0.89 | 1.35* | 1.67E-02 |
| nme1 | BC005629 | 0.74 | 0.75 | 3.17** | 2.38** |  | 0.79 | 2.46** | 1.43 | 1.61** | 3.69E-02 |
| nod1 | BB138330 | 0.97 | 0.29** | 1.26 | 1.25 |  | 0.61* | 0.79 | 0.46** | 1.39 | 4.61E-02 |
| nol1 | BC007151 | 1.69** | 2.19** | 1.53** | 2.57** |  | 1.28 | 1.73** | 1.42 | 1.77** | 2.05E-04 |
| nol5 | BB729616 | 1.02 | 1.83* | 2.31** | 2.28** |  | 1.3 | 5.12** | 1.33 | 1.64* | 4.43E-03 |
| nol5a | BF660256 | 1.51** | 3.70** | 2.39** | 2.46** |  | 1.75** | 3.29** | 2.20** | 1.65** | 4.71E-02 |
| nol5a | BM249243 | 2.85** | 2.47** | 2.95** | 3.34** |  | 1.32 | 1.40* | 1.60* | 1.67** | 4.98E-02 |
| nolc1 | BM213850 | 1.79* | 2.35* | 0.95 | 2.99** |  | 1.59* | 1.81* | 1.87* | 1.03 | 1.10E-02 |
| nolc1 | BM213850 | 4.16** | 2.07* | 2.37** | 3.98** |  | 1.06 | 1.25 | 1.35 | 1.77* | 1.87E-04 |
| nolc1 | NM_053086 | 1.96* | 0.94 | 1.28 | 2.67** |  | 1.23 | 1.31 | 1.79 | 0.83 | 7.33E-03 |
| nsun2 | BC013625 | 2.18** | 1.44* | 6.21** | 3.42** |  | 0.93 | 2.46** | 1.42* | 1.36* | 8.79E-03 |
| nubp1 | NM_011955 | 1.1 | 1.51** | 2.81** | 3.36** |  | 0.61** | 2.44** | 1.07 | 2.96** | 4.96E-02 |
| nubp1 | NM_011955 | 0.99 | 2.14** | 1.27* | 1.46* |  | 0.85 | 1.73** | 0.88 | 1.54** | 5.57E-03 |
| nucb1 | NM_008749 | 0.63** | 0.73* | 0.94 | 0.9 |  | 0.75* | 1.99** | 0.72* | 1.02 | 1.33E-02 |
| nufip1 | NM_013745 | 2.10** | 1.08 | 2.80** | 2.25** |  | 1.26* | 1.58** | 1.13 | 1.13 | 1.12E-02 |
| nup54 | AK014260 | 2.08** | 1 | 1.06 | 0.77 |  | 1.43** | 1.38* | 1.42* | 0.87 | 3.58E-02 |
| nup54 | AW681678 | 2.22** | 2.84** | 2.83** | 2.92** |  | 1.72** | 1.64** | 1.22* | 1.12 | 1.14E-02 |
| nup54 | BB168451 | 2.12** | 3.00** | 1.54** | 1.98** |  | 1.04 | 1.42** | 1.23* | 0.88 | 2.56E-02 |
| nup62 | NM_053074 | 1.83** | 2.56** | 4.57** | 2.56** |  | 0.77** | 0.88 | 1.05 | 1.27** | 8.31E-04 |
| nup62 | AW240611 | 2.10** | 2.29** | 2.91** | 2.73** |  | 0.78 | 0.83 | 1.11 | 1.17 | 3.61E-03 |
| odc1 | S64539 | 1.72** | 2.29** | 1.83** | 1.82** |  | 1.11 | 1.62** | 0.99 | 1.81** | 2.43E-02 |
| pa2g4 | AA672939 | 1.88** | 4.40** | 1.43* | 1.39 |  | 0.82 | 1.40* | 1.38 | 0.8 | 1.44E-02 |
| pa2g4 | BM232515 | 2.13* | 1.08 | 1.98** | 2.86** |  | 1 | 1.14 | 1.28 | 1.12 | 4.89E-03 |
| pa2g4 | AI152156 | 2.47** | 1.71* | 1.02 | 2.25** |  | 1.26 | 0.56** | 3.08** | 0.87 | 4.77E-02 |
| pa2g4 | BM232515 | 2.16** | 4.62** | 2.95** | 2.20** |  | 0.72 | 1.36 | 1.06 | 1.13 | 3.36E-02 |
| pa2g4 | AA672939 | 2.06** | 2.15** | 0.49** | 1.05 |  | 0.97 | 0.87 | 1.39* | 1.25 | 9.80E-03 |
| pak3 | BB468082 | 0.39** | 0.61* | 0.82 | 0.48** |  | 0.92 | 0.7 | 1.04 | 1.08 | 9.33E-03 |
| pcm1 | NM_023662 | 0.48** | 0.60** | 0.77* | 0.64** |  | 1.37** | 0.91 | 1.64** | 1.13 | 1.77E-02 |
| pcna | BC010343 | 1 | 2.44** | 17.91** | 4.75** |  | 1.32 | 3.24** | 0.76 | 3.08** | 3.38E-02 |
| pdcd11 | AK003899 | 3.54** | 1.52* | 2.21** | 3.35** |  | 1.49* | 1.07 | 1.63* | 1.14 | 2.26E-04 |
| pdk1 | BC027196 | 3.86** | 3.60** | 3.29** | 5.30** |  | 0.99 | 1.24* | 1.57** | 0.87 | 2.29E-03 |
| pdk1 | BC027196 | 4.05** | 4.82** | 4.97** | 6.59** |  | 1.07 | 1.29* | 1.24 | 1.2 | 2.60E-02 |
| pdk1 | BB391928 | 2.11** | 2.79** | 1.06 | 1.82** |  | 1.26 | 0.95 | 0.99 | 1.37* | 1.64E-03 |
| pdk1 | BB553369 | 3.03** | 2.79** | 2.07** | 2.31** |  | 0.89 | 1.27 | 1.01 | 1.50* | 3.40E-02 |
| pdk3 | AV086243 | 0.46** | 0.37** | 2.53** | 2.00* |  | 0.55** | 1.01 | 0.71 | 1.31 | 4.43E-03 |
| pet112l | BC005709 | 0.97 | 0.89 | 2.01** | 0.93 |  | 0.82 | 2.96** | 0.96 | 1.26 | 1.49E-02 |
| phb2 | NM_007531 | 2.74** | 1.98** | 3.74** | 2.64** |  | 0.89 | 2.56** | 0.98 | 2.36** | 2.83E-02 |
| phb2 | AV212294 | 2.77** | 2.47** | 1.75** | 2.39** |  | 1.12 | 1.22 | 1.55** | 1.03 | 1.11E-04 |
| phb2 | BB791424 | 2.67** | 3.20** | 2.43** | 2.23** |  | 0.8 | 1.42** | 1.1 | 1.39** | 2.80E-02 |
| phb2 | BE457485 | 2.08* | 1.59 | 0.69 | 3.27** |  | 1.3 | 1.14 | 0.91 | 1.03 | 1.10E-02 |
| phgdh | L21027 | 1.03 | 1.08 | 2.09** | 2.04** |  | 0.86 | 2.33** | 0.95 | 0.82 | 2.55E-02 |
| phgdh | AV216768 | 1.50** | 2.48** | 2.08** | 2.31** |  | 0.54** | 1.50** | 0.75* | 1.21 | 7.28E-03 |
| phgdh | AA561726 | 1.32* | 2.57** | 2.43** | 1.80** |  | 0.68* | 1.68** | 0.73* | 1.31* | 2.36E-02 |
| phgdh | BB204486 | 1.34* | 2.60** | 2.04** | 1.86** |  | 0.67** | 1.75** | 0.72* | 1.09 | 5.96E-03 |
| phgdh | BB495884 | 1.62** | 2.49** | 1.65** | 2.99** |  | 0.8 | 1.42* | 0.74* | 0.92 | 1.61E-02 |
| pigf | NM_008838 | 0.98 | 2.22** | 2.07** | 1.37* |  | 0.9 | 1.14 | 1.06 | 0.83 | 1.33E-02 |
| pim1 | BE631223 | 0.60* | 1.39 | 0.66* | 0.39** |  | 1.03 | 0.32** | 1.02 | 0.72 | 2.35E-02 |
| pir | AK009757 | 0.73* | 1.12 | 0.91 | 1.06 |  | 0.48** | 1.1 | 0.60** | 1.07 | 3.44E-02 |
| pkm2 | NM_011099 | 0.87 | 1.25 | 3.30** | 3.62** |  | 0.77 | 5.51** | 0.91 | 2.08* | 4.52E-02 |
| pla1a | NM_134102 | 1.12 | 1.55 | 0.69 | 1.3 |  | 0.34** | 3.72** | 0.61 | 3.93** | 8.88E-03 |
| pld1 | BM228590 | 0.69** | 0.58** | 0.51** | 1.15 |  | 0.53** | 0.41** | 0.65** | 0.57** | 2.05E-02 |
| pls3 | BC005459 | 0.67** | 0.44** | 0.96 | 1.03 |  | 1.07 | 0.67** | 1.09 | 0.74* | 1.84E-02 |
| pmaip1 | NM_021451 | 1.15 | 1.13 | 1.02 | 0.85 |  | 1.33* | 0.45** | 0.77* | 0.87 | 6.77E-03 |
| pms2 | BM239600 | 1.32 | 4.82** | 3.12** | 0.9 |  | 0.69* | 1.32 | 0.93 | 1.76** | 4.19E-02 |
| pold1 | BB385244 | 1.43* | 4.25** | 4.86** | 1.81** |  | 0.64* | 0.53** | 1.07 | 1.01 | 1.24E-02 |
| pold2 | NM_008894 | 2.57** | 3.35** | 6.60** | 3.75** |  | 0.65** | 2.71** | 1.03 | 1.78** | 1.85E-02 |
| polr2f | BC024419 | 0.95 | 0.99 | 2.81** | 3.12** |  | 1.6 | 2.29** | 0.91 | 0.94 | 2.93E-02 |
| polr3k | AV260647 | 1.41** | 2.05** | 2.11** | 1.56** |  | 0.94 | 0.88 | 1.57** | 0.91 | 2.15E-02 |
| ppat | AV305746 | 2.24** | 2.87** | 1.32* | 1.82** |  | 1.37* | 0.78 | 1.46** | 0.88 | 1.57E-02 |
| ppp1cc | BG071790 | 1.42 | 0.40** | 2.62** | 2.50** |  | 1.01 | 1.07 | 1.37 | 1.91** | 2.52E-02 |
| ppp1r7 | NM_023200 | 1.41** | 2.32** | 1.48** | 1.09 |  | 0.84* | 0.74** | 1.17 | 0.82* | 3.74E-03 |
| ppp2r4 | BB369168 | 1.17 | 2.10** | 1.01 | 1.03 |  | 0.73 | 1.48* | 1.03 | 1.3 | 3.73E-02 |
| prdm8 | AV349236 | 1.06 | 0.89 | 1.04 | 1 |  | 0.46** | 0.27** | 0.35** | 0.96 | 2.80E-02 |
| prdx3 | NM_007452 | 1.06 | 1.42* | 1.63** | 0.88 |  | 1 | 2.27** | 1.12 | 1.27 | 5.69E-04 |
| prkcdbp | BC009660 | 1.19 | 1 | 1.23 | 1.42* |  | 0.62** | 3.14** | 0.64** | 2.02** | 2.34E-02 |
| prps2 | BC024942 | 1.55** | 2.21** | 1.72** | 1.83** |  | 1.05 | 1.49** | 1.22 | 1.07 | 3.68E-02 |
| prps2 | BM934034 | 2.09** | 1.85** | 1.90** | 2.45** |  | 1.32 | 0.86 | 1.2 | 1.09 | 2.84E-03 |
| prtn3 | U97073 | 0.94 | 0.95 | 1.22 | 1.01 |  | 1.24 | 2.09** | 1.56* | 3.45** | 1.37E-02 |
| pscdbp | BB503614 | 2.37** | 0.6 | 10.36** | 7.00** |  | 1 | 1 | 1 | 0.89 | 3.16E-02 |
| pscdbp | AI462064 | 1.04 | 0.9 | 0.93 | 0.96 |  | 0.87 | 0.49** | 1.46* | 0.75* | 3.46E-02 |
| psma5 | BC010709 | 0.87 | 0.99 | 2.80** | 1.77** |  | 0.88 | 2.05** | 1.01 | 1.65** | 2.13E-02 |
| psmc4 | NM_011874 | 1.21 | 0.74 | 3.27** | 2.46** |  | 0.95 | 2.30** | 0.91 | 2.10** | 1.92E-02 |
| psph | NM_133900 | 1.26 | 1.55* | 1.46* | 1.35 |  | 0.72 | 2.08** | 0.79 | 1.14 | 4.02E-02 |
| ptdss1 | AU044268 | 1.56** | 2.66** | 0.52** | 1.03 |  | 1.14 | 0.92 | 1.85** | 0.98 | 4.70E-02 |
| ptprn | NM_008985 | 0.38** | 0.46** | 0.82 | 1.79* |  | 0.83 | 0.98 | 0.85 | 0.97 | 8.88E-03 |
| rab11a | BC010722 | 0.54** | 0.37** | 2.21** | 0.93 |  | 0.51** | 1.04 | 0.64** | 1.44* | 3.13E-02 |
| rabif | BB085124 | 0.79 | 0.51** | 1.07 | 0.92 |  | 1 | 1.05 | 1.08 | 0.62** | 2.41E-02 |
| rac1 | BC003828 | 0.88 | 0.82 | 1.37 | 0.95 |  | 1.22 | 2.54** | 1.06 | 1.96** | 4.22E-02 |
| rad50 | NM_009012 | 1.69* | 3.12** | 1.35 | 3.98** |  | 0.98 | 1.33 | 1.09 | 1.2 | 2.11E-02 |
| rad51 | NM_011234 | 1.02 | 6.19** | 23.30** | 2.40** |  | 0.82 | 1.12 | 1.90** | 1.46 | 1.80E-02 |
| ran | AV090150 | 1.81** | 1.68** | 2.64** | 2.17** |  | 0.91 | 2.16** | 1.27 | 1.74** | 3.03E-02 |
| rbm4 | NM_009032 | 1.33** | 0.95 | 1.97** | 1.05 |  | 1.45** | 2.00** | 1.11 | 1.19* | 1.79E-02 |
| rfc4 | BB251459 | 1.09 | 6.55** | 4.76** | 3.77** |  | 0.75 | 0.54* | 1.29 | 0.83 | 2.79E-02 |
| rgs16 | BB100249 | 1.49* | 1.41* | 1.07 | 1.96** |  | 0.79 | 1.92** | 1.74** | 0.68* | 4.10E-04 |
| ripk2 | NM_138952 | 2.07** | 0.81 | 1.13 | 2.48** |  | 1.21 | 1.09 | 0.85 | 1.03 | 7.43E-03 |
| rnf4 | AV045658 | 0.88 | 0.93 | 1.43* | 1.69** |  | 1.12 | 2.10** | 1.16 | 1.1 | 2.81E-02 |
| rpa2 | BC004578 | 2.31** | 6.91** | 6.75** | 3.16** |  | 0.95 | 1.36 | 0.98 | 1.46 | 2.86E-03 |
| rpe | BG916066 | 0.70** | 1.25* | 1.42* | 0.85 |  | 0.87 | 2.22** | 1.05 | 0.82 | 9.99E-03 |
| rpl30 | BB283415 | 2.95** | 1.04 | 1 | 1 |  | 1.32 | 0.78 | 0.98 | 1.47* | 8.45E-03 |
| rps13 | NM_026533 | 1.97** | 0.77** | 1.12 | 1.67** |  | 1.07 | 0.53** | 1.06 | 1.30** | 3.28E-02 |
| rps24 | BM119287 | 3.12** | 1.17 | 0.68 | 1.33 |  | 0.7 | 1.54 | 0.84 | 2.09** | 1.79E-02 |
| rps25 | BM729504 | 1.49 | 0.74 | 0.71 | 1.1 |  | 1.08 | 2.32** | 1.53* | 1.41 | 4.78E-02 |
| rps6ka5 | BQ174267 | 0.7 | 0.83 | 0.91 | 1.08 |  | 1.02 | 0.39** | 0.57** | 0.85 | 1.41E-02 |
| rrm2 | NM_009104 | 0.98 | 0.97 | 1.21 | 0.88 |  | 0.46** | 0.53** | 0.55** | 1.07 | 9.23E-03 |
| s100a9 | NM_009114 | 1.32 | 0.88 | 1.73* | 0.93 |  | 0.39** | 0.71 | 0.77 | 0.23** | 4.69E-02 |
| sae1 | AK011772 | 1.08 | 1.01 | 1.01 | 1.51* |  | 1.44* | 2.70** | 1.35 | 1.03 | 2.87E-02 |
| sdf2l1 | NM_022324 | 0.68* | 1.94** | 1.82** | 1 |  | 1.06 | 3.26** | 1.31 | 3.78** | 6.92E-03 |
| sec23b | BC011160 | 0.46** | 0.43** | 1.88** | 0.92 |  | 0.78 | 1.59** | 0.95 | 1.1 | 4.40E-02 |
| sfrs1 | BF147037 | 1.69** | 1.28 | 1.91** | 0.95 |  | 0.83 | 2.47** | 0.86 | 1.45** | 1.73E-02 |
| sfrs1 | BF147037 | 3.06** | 2.06** | 1.07 | 3.42** |  | 1.47* | 1.22 | 1.21 | 1.28 | 8.63E-05 |
| sfrs1 | BF682801 | 1.80** | 1.36 | 3.11** | 2.31** |  | 0.9 | 2.47** | 1.21 | 2.80** | 6.40E-03 |
| sfrs1 | X66091 | 2.48** | 1.61 | 1.18 | 4.06** |  | 0.74 | 0.61 | 1.12 | 1.5 | 3.54E-02 |
| sfrs2 | AF250135 | 2.21** | 1.2 | 1.29* | 1.47** |  | 0.58** | 0.69** | 0.9 | 1.63** | 2.71E-02 |
| sfrs7 | BC014857 | 1.99** | 0.9 | 1.53** | 1.04 |  | 0.84 | 1.63** | 0.85 | 1.41** | 7.82E-03 |
| sfxn1 | BB478992 | 2.05** | 0.77 | 2.45** | 1.98** |  | 0.96 | 1.82** | 1.07 | 1.36 | 1.25E-02 |
| sgpl1 | NM_009163 | 1 | 0.72** | 1.40** | 0.79** |  | 0.76** | 0.46** | 0.72** | 1.30** | 4.84E-02 |
| shmt1 | AF237702 | 1.61** | 3.95** | 1 | 1.23 |  | 1.15 | 0.63* | 1.38 | 0.76 | 2.27E-02 |
| shmt1 | AF237702 | 1.54* | 2.55** | 1.21 | 2.10** |  | 0.82 | 0.78 | 1.11 | 1.14 | 2.21E-02 |
| shmt1 | AF237702 | 2.51** | 1.97 | 4.18** | 2.62** |  | 1.11 | 1.21 | 1.08 | 1.21 | 3.66E-02 |
| si | NM_021882 | 0.96 | 1.01 | 1.06 | 0.85 |  | 0.51** | 0.43** | 1.25 | 0.77 | 4.89E-03 |
| sip1 | AK013414 | 1.31 | 2.76** | 1.89** | 1.68** |  | 1.09 | 0.98 | 1.07 | 0.86 | 4.40E-02 |
| slc11a1 | NM_013612 | 0.97 | 1.49 | 2.45 | 1.95* |  | 0.67 | 1.77 | 0.88 | 3.06** | 1.64E-02 |
| slc16a1 | NM_009196 | 9.55** | 6.38** | 4.09** | 6.82** |  | 0.95 | 1.15 | 0.95 | 0.63* | 3.61E-03 |
| slc19a1 | AI323572 | 3.00** | 2.94** | 1.07 | 2.71** |  | 1.12 | 1 | 1.58* | 0.8 | 2.34E-04 |
| slc19a1 | NM_031196 | 3.22** | 2.18** | 1.99** | 4.45** |  | 1.08 | 1.84** | 1.42 | 1.27 | 1.75E-04 |
| slc20a1 | NM_015747 | 2.16** | 1.32 | 2.15** | 1.22 |  | 1.28 | 1 | 1.1 | 1.04 | 1.36E-03 |
| slc2a2 | NM_031197 | 0.84* | 0.43** | 0.94 | 0.76** |  | 0.74** | 1 | 1.19* | 0.91 | 2.91E-02 |
| slc7a1 | BB264620 | 3.27** | 1.83* | 1.47* | 2.73** |  | 2.28** | 0.9 | 3.08** | 0.46** | 2.89E-02 |
| slc7a1 | BB264620 | 2.33** | 1.26 | 1.66** | 2.49** |  | 1.59** | 1.43* | 2.30** | 1.06 | 4.94E-05 |
| slc7a5 | BC026131 | 1.68** | 1.46* | 1.38* | 1.32 |  | 1.44* | 3.07** | 2.24** | 1.16 | 1.90E-02 |
| slc9a3r2 | AK004710 | 1.28 | 0.73 | 1.41* | 0.76 |  | 1.14 | 2.48** | 1.04 | 1.53* | 4.09E-02 |
| snrpd3 | AW046420 | 2.16** | 1.1 | 2.14** | 0.88 |  | 1.2 | 0.75** | 1.66** | 0.63** | 2.21E-03 |
| sord | BI143942 | 0.89 | 0.50** | 1.53* | 0.67 |  | 0.51** | 0.36** | 0.68 | 1.65* | 3.18E-02 |
| sord | AV253518 | 0.79 | 0.66** | 1.21 | 0.99 |  | 0.66** | 0.33** | 0.63** | 0.83 | 4.29E-02 |
| sp4 | NM_009239 | 0.78* | 0.47** | 0.8 | 1.15 |  | 1 | 0.77* | 0.88 | 0.74* | 1.08E-03 |
| sp4 | AI324972 | 0.54* | 0.72* | 0.89 | 0.33** |  | 1.32 | 0.38** | 1.43 | 0.67 | 9.49E-03 |
| spna2 | AK011566 | 0.8 | 0.45** | 1.21 | 1.03 |  | 0.81 | 0.67** | 0.69 | 0.89 | 2.19E-03 |
| sqstm1 | BM237736 | 0.58** | 0.29** | 1.08 | 0.52** |  | 0.83 | 0.57** | 0.95 | 0.99 | 2.73E-04 |
| sqstm1 | BM232298 | 0.44** | 0.28** | 2.68** | 0.57* |  | 1.93* | 2.25** | 0.79 | 0.91 | 3.53E-02 |
| sri | AK008404 | 0.56** | 0.47** | 0.82 | 0.95 |  | 0.82 | 0.79 | 1.13 | 1.93** | 1.94E-02 |
| srm | NM_009272 | 1.69** | 1.61** | 1.72** | 2.18** |  | 0.67* | 2.94** | 1.29 | 2.34** | 2.04E-02 |
| srr | AK002636 | 0.60** | 0.93 | 1.17 | 1.18 |  | 1.89** | 0.82 | 1.3 | 0.93 | 1.64E-03 |
| st18 | AV347235 | 0.49** | 0.41** | 0.82 | 1.05 |  | 1.05 | 0.71* | 0.9 | 0.98 | 2.05E-02 |
| stmn1 | BC010581 | 0.69 | 2.83** | 6.24** | 1.74** |  | 1.04 | 0.91 | 1.02 | 0.93 | 3.30E-02 |
| styx | BB812465 | 2.36** | 2.24** | 1.17* | 1.55 |  | 0.77 | 0.98 | 1.21 | 0.95 | 4.80E-02 |
| styx | BB812465 | 4.35** | 3.00** | 1.58** | 2.53** |  | 0.79 | 0.76* | 1.08 | 1.03 | 3.90E-02 |
| supt16h | AW536705 | 2.61* | 1.45 | 0.98 | 7.03** |  | 2.14* | 1.89 | 1.54 | 0.72 | 8.71E-04 |
| syngr2 | BC004829 | 1.4 | 1.61* | 1.55** | 1.96** |  | 0.45** | 1.65* | 0.75 | 1.77** | 2.41E-02 |
| syngr2 | BC004829 | 1.44 | 1.46* | 2.53** | 1.82** |  | 0.60* | 2.56** | 0.76 | 1.49* | 2.15E-02 |
| synj1 | BM232846 | 0.72* | 0.36** | 0.66** | 0.55** |  | 0.88 | 0.60** | 1.12 | 0.81 | 5.73E-03 |
| synj1 | BM232846 | 0.55** | 0.29** | 0.62** | 0.68** |  | 0.93 | 0.9 | 0.71* | 1.12 | 8.56E-05 |
| sypl | AV232599 | 1.03 | 0.83 | 0.72 | 0.74 |  | 1.08 | 0.49** | 0.99 | 0.54** | 3.40E-02 |
| tagln | BB114067 | 0.98 | 0.56* | 1.31 | 3.52** |  | 1.56 | 2.83** | 0.47** | 2.56** | 2.85E-02 |
| tbl3 | BC019504 | 1.41* | 1.23 | 1.61** | 1.02 |  | 1.05 | 2.17** | 1.12 | 1.78** | 3.69E-02 |
| tcn2 | BB449960 | 0.43* | 0.82 | 0.83 | 0.55 |  | 0.57 | 1.19 | 0.53 | 1.44 | 2.68E-02 |
| tcn2 | NM_015749 | 0.59** | 0.68* | 0.93 | 1.01 |  | 0.60** | 2.14** | 0.42** | 1.47* | 2.08E-02 |
| tcta | BC019397 | 0.62* | 0.36** | 1.46* | 0.88 |  | 0.68* | 1.45* | 0.92 | 1.16 | 1.97E-03 |
| tes | BC003808 | 0.42** | 0.50** | 1.67* | 0.64* |  | 0.76 | 0.97 | 0.78 | 1.24 | 3.06E-03 |
| tes | BC010465 | 0.39** | 0.40** | 0.91 | 0.39** |  | 0.87 | 1.29* | 0.73** | 1.25* | 1.72E-02 |
| tfdp1 | BG075396 | 3.08** | 1.70** | 3.28** | 3.13** |  | 1.11 | 1.43* | 1.52* | 1.27 | 9.57E-03 |
| tfrc | BB810450 | 3.87** | 0.8 | 0.72 | 1.79* |  | 1.67* | 1.37 | 1.05 | 1.26 | 4.56E-02 |
| thop1 | NM_022653 | 2.11* | 2.35** | 4.46** | 9.23** |  | 0.83 | 3.07** | 0.71 | 1.04 | 2.41E-02 |
| timm9 | BC028435 | 2.96** | 2.11** | 1.08 | 1.25 |  | 1.13 | 0.97 | 0.93 | 1.54** | 1.32E-03 |
| timm9 | NM_013896 | 2.36** | 1.13 | 1.95** | 4.68** |  | 1.03 | 2.40** | 0.94 | 1.85** | 2.87E-02 |
| tmem4 | NM_019953 | 0.60** | 0.38** | 0.86 | 0.56** |  | 1 | 1 | 1.05 | 1.08 | 2.46E-02 |
| top1 | BB127876 | 2.44** | 0.82 | 0.85 | 1.06 |  | 1.35 | 1.02 | 0.73 | 2.16** | 1.44E-02 |
| topbp1 | BC007170 | 2.03** | 2.70** | 5.72** | 6.88** |  | 0.60** | 0.75 | 1.07 | 1.09 | 4.91E-07 |
| trip12 | BG923744 | 0.91 | 0.42** | 1.13 | 0.51** |  | 0.71* | 0.79 | 0.72* | 1.01 | 5.28E-03 |
| tro | AF288606 | 1.06 | 0.49** | 0.97 | 0.73** |  | 1.1 | 0.92 | 1.04 | 0.91 | 3.98E-02 |
| ttc4 | BC025435 | 0.85 | 0.83 | 1.66** | 1.38 |  | 0.69* | 2.07** | 0.68** | 1.86** | 2.42E-02 |
| txnl4 | AW552577 | 2.10** | 1.49** | 1.92** | 3.24** |  | 0.98 | 1.43** | 1.05 | 1.72** | 1.37E-02 |
| u2af1 | NM_024187 | 0.86 | 0.67 | 3.05** | 1.84* |  | 0.97 | 2.16** | 1.18 | 1.49 | 3.49E-02 |
| uap1 | NM_133806 | 0.77* | 0.9 | 3.27** | 0.58** |  | 1.12 | 2.10** | 1.21* | 1.23 | 1.10E-02 |
| uchl1 | NM_011670 | 0.47** | 0.32** | 1.54 | 3.22** |  | 1.07 | 1.05 | 0.99 | 1.18 | 3.02E-02 |
| urod | NM_009478 | 1.31 | 0.52** | 1.83** | 1.23 |  | 0.98 | 2.30** | 0.93 | 1.2 | 4.63E-02 |
| usp1 | BC018179 | 1.99** | 2.41** | 3.86** | 3.08** |  | 1.24 | 0.78 | 1.14 | 0.95 | 3.45E-03 |
| usp4 | BF321773 | 1.32* | 0.81 | 0.99 | 0.71** |  | 1.15 | 2.08** | 0.77* | 1.44** | 1.72E-02 |
| uxt | NM_013840 | 0.81 | 1.08 | 2.90** | 1.52** |  | 0.82 | 2.24** | 0.85 | 1.48** | 6.49E-03 |
| vamp2 | BG871810 | 0.54** | 0.63* | 1.31 | 0.83 |  | 0.50** | 2.42** | 0.65* | 1.58* | 1.11E-02 |
| vars2 | AV258022 | 1.19 | 2.25* | 1.4 | 2.42** |  | 0.89 | 1.15 | 1.23 | 0.96 | 2.72E-02 |
| vasp | BC015289 | 0.57** | 0.30** | 2.03** | 0.45** |  | 0.56** | 1.42 | 0.69* | 2.20** | 3.93E-02 |
| vldlr | AV333363 | 0.78 | 0.59** | 0.9 | 0.57** |  | 1.35 | 0.48** | 1.44 | 0.60** | 3.19E-02 |
| vldlr | BB127955 | 0.66* | 0.39** | 2.13** | 0.60* |  | 1.37 | 0.58* | 1.32 | 0.57** | 2.50E-02 |
| wdr3 | BG063575 | 2.43** | 1.73** | 0.99 | 2.11** |  | 0.95 | 0.87 | 1.25 | 1.04 | 2.70E-03 |
| wdr4 | BE854862 | 2.04** | 1.25 | 1.34** | 1.48* |  | 1.12 | 1.44** | 1.22 | 1.57** | 3.43E-05 |
| zdhhc3 | AV076190 | 2.30** | 0.99 | 0.62** | 1.55** |  | 1.30* | 0.67** | 1.07 | 0.72* | 1.22E-02 |
| zfp9 | BB008902 | 0.36** | 0.48** | 1.48 | 0.65* |  | 0.79 | 2.01** | 0.68 | 1.37 | 2.62E-02 |

**Table S2: Cell cycle related genes showing significant change in expression within 8 hours of MYC-ER^TAM^ activation.**

Genes relating to mitosis and cell-cycle progression showing a significant change in expression within 8 hours following activation of MYC-ER^TAM^. ‘MYC-response p-value’ is the p-value identified for the highest-order interaction of the MYC activation variable and represents the significance of this term within the selected model. Flags represent contrast p-values comparing 4OHT-treated and vehicle-treated samples at specific time points (‘*’, p ≤ 0.05; ‘**’ p ≤ 0.01). Cells are colour-coded based on a detected fold-change greater than 1.5-fold (red, up-regulated; blue, down-regulated).

|  |  | **Pancreatic β-cells** | | | |  | **Suprabasal Keratinocytes** | | | | **Myc-response p-value** |
| --- | --- | --- | --- | --- | --- | --- | --- | --- | --- | --- | --- |
| **Gene Symbol** | **RefSeq** | **4 hrs** | **8 hrs** | **16 hrs** | **32 hrs** |  | **4 hrs** | **8 hrs** | **16 hrs** | **32 hrs** |
| ahr | BE989096 | 1.02 | 0.65* | 0.93 | 1.49* |  | 1.14 | 0.45** | 0.89 | 0.49** | 3.14E-02 |
| ai467657 | AA419994 | 0.57** | 1.57* | 1.29 | 0.51** |  | 0.86 | 0.33** | 0.99 | 0.45** | 1.30E-02 |
| akap8 | BB037566 | 2.65** | 1.85** | 0.97 | 1.49** |  | 1.29* | 0.97 | 1.12 | 1.08 | 9.91E-03 |
| akap8 | BG069776 | 2.40** | 1.76* | 1.23 | 1.91** |  | 1.3 | 0.54** | 1.13 | 0.94 | 3.32E-02 |
| akt1 | NM_009652 | 1.01 | 0.97 | 1.71** | 2.31** |  | 0.82 | 3.23** | 0.95 | 2.29** | 4.43E-02 |
| akt2 | NM_007434 | 1 | 1.05 | 0.98 | 1.13 |  | 1.27 | 2.21** | 1.02 | 2.26** | 7.85E-03 |
| anapc1 | AV113524 | 2.35** | 0.44** | 4.33** | 2.32** |  | 1.07 | 0.99 | 0.98 | 1.34 | 2.10E-02 |
| appl1 | BG073343 | 0.97 | 0.35** | 0.45** | 0.99 |  | 1.25 | 0.62* | 1 | 0.89 | 5.96E-03 |
| atf5 | AF375476 | 1.05 | 0.66 | 1.57* | 1 |  | 0.92 | 2.94** | 0.88 | 3.18** | 3.70E-02 |
| b230120h23rik | BB561086 | 1.27 | 1.42 | 1.41 | 1.26 |  | 2.25** | 1.49* | 1.67* | 0.61* | 1.61E-03 |
| bin1 | U60884 | 1 | 0.46** | 2.08** | 1.05 |  | 1.04 | 3.12** | 1.05 | 2.84** | 1.92E-02 |
| bin1 | BG293813 | 0.99 | 0.32** | 0.40** | 0.51** |  | 1.06 | 0.84 | 1.02 | 1.11 | 3.76E-02 |
| bmp2 | AV239587 | 1.1 | 1.39 | 0.64* | 1.11 |  | 0.47** | 0.39** | 0.80* | 0.38** | 8.19E-03 |
| bmp4 | NM_007554 | 0.79 | 0.92 | 1.18 | 1.17 |  | 0.38** | 0.76 | 0.50** | 0.69* | 2.66E-02 |
| brms1 | NM_134155 | 0.96 | 0.91 | 1.19 | 1.42 |  | 1.61 | 3.49** | 1.52 | 1 | 3.70E-02 |
| brms1l | AK003055 | 1.01 | 2.35** | 1.59** | 1.08 |  | 0.84 | 1.14 | 1.17 | 1.16 | 4.85E-02 |
| btc | NM_007568 | 1.16 | 1.22 | 0.96 | 0.82 |  | 0.33** | 0.27** | 0.63** | 0.47** | 7.05E-04 |
| btc | AV231340 | 1.12 | 1.08 | 1.14 | 1.23 |  | 0.78 | 0.34** | 0.64** | 0.57** | 6.47E-03 |
| bub1b | AU045529 | 1.55* | 3.35** | 13.72** | 2.58** |  | 0.46** | 0.55* | 1.09 | 0.8 | 5.35E-05 |
| camk2d | NM_023813 | 0.97 | 1.32 | 0.64** | 0.94 |  | 0.85 | 0.45** | 0.8 | 0.53** | 4.56E-03 |
| camk2d | AF059029 | 1.17 | 1.2 | 1.17 | 1.08 |  | 0.74* | 2.02** | 0.93 | 1.15 | 1.63E-02 |
| camk2d | AV337193 | 0.96 | 1.51* | 1.02 | 0.92 |  | 0.51** | 0.50** | 0.59** | 0.35** | 4.42E-02 |
| ccna2 | X75483 | 0.96 | 4.52** | 10.49** | 3.25** |  | 0.81 | 0.67 | 1.39 | 0.97 | 2.05E-02 |
| ccnb1 | NM_007629 | 1.02 | 0.90 | 2.38** | 1.13 |  | 0.48** | 1.03 | 1.01 | 0.58** | 9.74E-03 |
| ccnb1 | NM_007629 | 1.06 | 2.85** | 8.15** | 3.62** |  | 0.6 | 0.46* | 1.39 | 0.61 | 3.73E-02 |
| ccnd1 | NM_007631 | 1.86** | 2.09** | 0.88 | 1.45 |  | 1.13 | 1.31 | 1.79* | 1.04 | 4.55E-02 |
| ccnd1 | NM_007631 | 1.69 | 2.38 | 1.41 | 2.27* |  | 1.05 | 1.33 | 1.72 | 1.26 | 2.24E-02 |
| ccnd1 | NM_007631 | 3.41** | 2.74* | 2.03* | 2.05 |  | 1.35 | 0.99 | 1.54 | 1.81 | 2.62E-02 |
| ccnd2 | NM_009829 | 2.06** | 2.04** | 1.27 | 2.30** |  | 0.9 | 0.84 | 1.29 | 0.84 | 1.48E-02 |
| ccnd2 | AK007904 | 1.99** | 0.64* | 1.88** | 2.62** |  | 1.66* | 2.57** | 1 | 2.13** | 1.21E-02 |
| ccnd3 | NM_007632 | 1.26* | 0.84 | 1.54** | 1.2 |  | 0.81* | 2.58** | 0.84 | 3.33** | 1.28E-03 |
| ccne1 | NM_007633 | 1.82** | 3.37** | 7.02** | 1.71* |  | 0.94 | 1.35 | 1.15 | 1.82** | 2.38E-02 |
| ccne1 | BB293079 | 1.83** | 6.68** | 1.87** | 2.04** |  | 0.85 | 1.50* | 1.16 | 1.29 | 8.71E-04 |
| ccne2 | AF091432 | 2.62** | 5.86** | 5.92** | 7.86** |  | 1.74 | 1.11 | 1.42 | 1.39 | 4.65E-04 |
| ccng2 | U95826 | 0.39** | 0.41** | 1.44 | 0.60* |  | 0.77 | 0.59** | 0.8 | 0.93 | 7.05E-03 |
| ccni | NM_017367 | 0.58** | 0.49** | 0.72** | 0.84 |  | 1.28* | 0.86 | 1.1 | 0.98 | 3.73E-02 |
| ccnl2 | AK008585 | 0.93 | 0.43** | 1.09 | 0.48** |  | 1.04 | 1.04 | 0.82 | 0.71* | 2.11E-02 |
| cdc14a | BB479310 | 0.56* | 0.44** | 0.77 | 0.73 |  | 1.11 | 0.71 | 0.96 | 0.97 | 1.52E-02 |
| cdc14b | AK013228 | 2.02** | 0.29** | 0.69 | 1.47 |  | 0.91 | 0.31** | 0.77 | 0.61* | 1.18E-02 |
| cdc23 | BB492440 | 0.9 | 0.43** | 1.01 | 1.47** |  | 1.13 | 1 | 0.82* | 1.18 | 1.84E-03 |
| cdc25a | C76119 | 2.71** | 2.23** | 2.09** | 3.90** |  | 1.11 | 0.46** | 1.07 | 0.78 | 2.45E-02 |
| cdc25a | C76119 | 2.17** | 1.73* | 2.03** | 2.10** |  | 1.02 | 1.14 | 1.1 | 1.21 | 2.52E-02 |
| cdc2a | NM_007659 | 0.95 | 2.91** | 11.93** | 3.35** |  | 0.51* | 1.17 | 0.89 | 1.12 | 4.84E-02 |
| cdc34 | BI794243 | 0.8 | 0.75 | 2.35** | 2.20** |  | 1.15 | 2.19** | 1.31 | 1.79* | 1.33E-02 |
| cdc37 | AK013255 | 1.16 | 0.8 | 3.62** | 5.05** |  | 1.01 | 2.48** | 1.32 | 1.53* | 4.50E-02 |
| cdc37 | AK013255 | 1.99** | 2.19** | 1.40** | 1.67** |  | 1.09 | 1.32* | 1.52 | 1.06 | 4.41E-03 |
| cdc6 | NM_011799 | 2.18* | 7.25** | 6.48** | 6.44** |  | 0.68 | 0.62 | 1.16 | 1.25 | 7.15E-04 |
| cdc7 | AB018574 | 3.73** | 3.30** | 2.68** | 4.28** |  | 1 | 1.31 | 1.16 | 1.22 | 3.73E-02 |
| cdc73 | BM935271 | 0.99 | 1.24 | 0.81 | 0.72** |  | 0.92 | 0.40** | 1.03 | 0.52** | 9.95E-03 |
| cdca5 | NM_026410 | 0.79 | 12.44** | 18.42** | 10.23** |  | 0.49** | 0.7 | 1.39 | 1 | 1.47E-02 |
| cdca5 | NM_026410 | 0.85 | 2.06** | 14.63** | 3.08** |  | 0.84 | 1.27 | 1.54 | 1.37 | 2.23E-04 |
| cdca7 | AK011289 | 2.72** | 1.70** | 1.26* | 1.48* |  | 0.61** | 1.09 | 0.92 | 2.26** | 3.35E-03 |
| cdca7l | BC006933 | 2.72** | 2.60** | 3.81** | 4.37** |  | 1.42 | 1.33 | 1.25 | 1.41 | 1.02E-02 |
| cdk4 | NM_009870 | 1.16 | 0.92 | 8.41** | 1.11 |  | 1.32 | 12.27** | 0.74 | 1.62 | 1.45E-02 |
| cdk4 | NM_009870 | 1.21 | 0.74 | 5.31** | 1.5 |  | 1.23 | 11.30** | 0.92 | 1.48 | 4.33E-02 |
| cdk4 | NM_009870 | 1.2 | 0.88 | 5.03** | 1.3 |  | 1.37 | 11.17** | 0.72 | 1.37 | 2.70E-02 |
| cdk7 | U11822 | 0.63* | 0.77 | 3.69** | 3.45** |  | 0.82 | 2.12** | 1.09 | 1.59* | 5.01E-03 |
| cdkn1a | AK007630 | 1.57** | 2.05** | 1.71** | 1.24 |  | 0.96 | 1.44* | 0.99 | 0.9 | 1.02E-02 |
| cdkn1b | NM_009875 | 1.03 | 0.19** | 0.71 | 0.95 |  | 0.23** | 0.42* | 0.48* | 0.89 | 8.43E-03 |
| cdkn2a | NM_009877 | 2.17** | 1.79** | 1.57** | 2.61** |  | 1.21 | 1.07 | 1.07 | 0.87 | 1.20E-03 |
| cdkn2b | AF059567 | 0.43** | 0.29** | 0.76 | 0.47** |  | 0.58** | 0.86 | 0.67* | 0.72 | 9.38E-03 |
| cdkn2c | BC027026 | 0.32** | 1.18 | 5.62** | 0.59* |  | 0.85 | 1.28 | 0.87 | 1.60* | 4.22E-02 |
| cdt1 | AF477481 | 4.81** | 15.83** | 28.12** | 6.15** |  | 0.7 | 0.64 | 1.45 | 1.22 | 1.28E-02 |
| cdt1 | AF477481 | 3.31** | 3.50** | 11.38** | 8.04** |  | 0.71 | 0.92 | 0.76 | 1.79 | 1.56E-02 |
| cenpm | NM_025639 | 1.1 | 2.62** | 8.17** | 1.71 |  | 0.97 | 1 | 1.29 | 1.34 | 4.58E-02 |
| cep55 | AK004655 | 1.14 | 2.47** | 7.81** | 3.17** |  | 0.87 | 0.79 | 1.26 | 1.38* | 3.57E-02 |
| cetn3 | BC002162 | 1.21 | 0.72 | 4.44** | 2.53** |  | 0.66 | 2.16** | 0.93 | 1.34 | 4.26E-02 |
| cfl1 | NM_007687 | 1.29 | 0.51** | 1.3 | 1.89** |  | 1.03 | 2.28** | 1.15 | 2.14** | 9.67E-03 |
| cgref1 | BC023116 | 0.97 | 1.50** | 0.84 | 1.21 |  | 0.66** | 2.23** | 0.78* | 2.39** | 2.29E-02 |
| cgrrf1 | AV305616 | 0.67** | 0.42** | 1.62** | 0.57** |  | 1.05 | 1.33** | 1.14 | 1.15 | 3.04E-02 |
| cgrrf1 | AK004156 | 0.49** | 0.60* | 2.01** | 1.78** |  | 1.04 | 0.71 | 0.92 | 0.8 | 4.05E-03 |
| chek1 | BB298208 | 1.1 | 3.70** | 2.35** | 1.92** |  | 0.91 | 1.03 | 1.31 | 1.23 | 1.62E-02 |
| chek1 | C85740 | 1.94* | 4.38** | 4.71** | 4.17** |  | 0.9 | 0.48* | 1.28 | 1.03 | 1.98E-02 |
| chek2 | NM_016681 | 1.24 | 2.07** | 2.65** | 2.29** |  | 0.82 | 0.63* | 1.13 | 1.34 | 2.23E-02 |
| cks1b | NM_016904 | 1.50** | 2.92** | 4.05** | 2.82** |  | 0.82* | 1.06 | 0.97 | 1.09 | 6.79E-03 |
| cks1b | NM_016904 | 1.35* | 3.20** | 7.65** | 4.09** |  | 0.72* | 2.03** | 0.72 | 1.33 | 3.31E-02 |
| cks2 | NM_025415 | 0.92 | 3.03** | 10.08** | 4.80** |  | 0.51** | 0.76 | 1.27 | 0.71 | 3.45E-02 |
| cks2 | NM_025415 | 0.98 | 2.33** | 8.92** | 2.34** |  | 0.63* | 0.92 | 0.93 | 1.32 | 3.52E-02 |
| clspn | BG067086 | 1.29 | 2.48** | 6.52** | 1.61 |  | 0.85 | 1 | 1.03 | 1.55 | 3.35E-02 |
| cops5 | NM_013715 | 1.01 | 0.89 | 2.65** | 1.3 |  | 0.92 | 1.98** | 0.94 | 1.69** | 1.33E-02 |
| csf1r | AK004947 | 0.96 | 0.76 | 1.12 | 1.27 |  | 1.17 | 2.62** | 0.58** | 2.58** | 4.08E-02 |
| cul5 | BB702110 | 2.49** | 0.83 | 1.70** | 0.93 |  | 1.01 | 0.75 | 1.11 | 1.11 | 2.07E-02 |
| cwf19l1 | BB749215 | 1.62* | 2.10** | 0.64 | 0.98 |  | 1.21 | 0.87 | 1.18 | 0.69 | 1.39E-02 |
| d5ertd40e | C77487 | 0.58** | 0.49** | 1.15 | 0.48** |  | 0.81 | 0.49** | 1.29 | 1.08 | 4.15E-03 |
| dbf4 | NM_013726 | 3.42** | 4.23** | 10.95** | 4.62** |  | 0.40** | 1.29* | 1.07 | 1.58** | 1.16E-02 |
| dis3 | BM232345 | 2.52** | 1.98** | 2.21** | 3.96** |  | 1.27 | 0.78 | 1.53* | 0.56** | 1.37E-02 |
| dnajc2 | BG067003 | 2.09** | 2.02** | 2.63** | 1.74** |  | 0.65** | 1.30* | 1.15 | 1.85** | 4.61E-02 |
| dnajc2 | BG067003 | 2.29** | 1 | 2.55** | 1.71** |  | 0.9 | 0.73 | 1.29 | 1.56* | 8.71E-04 |
| dock4 | BG068753 | 3.67** | 2.23** | 1.18 | 1.63** |  | 0.68* | 0.79 | 1.39 | 1.50* | 6.56E-04 |
| dock5 | AK004325 | 2.54** | 1.85** | 1.70** | 1.94** |  | 1.28** | 0.56** | 1.47** | 0.56** | 4.65E-02 |
| e2f3 | BQ176318 | 2.47** | 1.42 | 1.12 | 1.58* |  | 1.35 | 0.83 | 1.74** | 0.74 | 2.95E-02 |
| e2f5 | BC003220 | 2.13* | 0.50* | 1.49 | 2.18* |  | 1.23 | 1.66 | 1.23 | 1.27 | 4.05E-02 |
| eef1e1 | NM_025380 | 3.22** | 2.14** | 7.58** | 7.88** |  | 1.4 | 3.42** | 1.52* | 1.63** | 3.52E-02 |
| elk3 | BC005686 | 0.62* | 0.36** | 0.60* | 0.71 |  | 0.69* | 0.87 | 0.82 | 1.44 | 1.48E-02 |
| erbb3 | BF140685 | 0.45** | 0.51** | 0.59* | 1.5 |  | 0.87 | 0.44** | 0.7 | 0.43** | 7.23E-03 |
| ereg | NM_007950 | 0.88 | 1.02 | 1.07 | 0.87 |  | 0.17** | 0.27** | 0.58** | 0.88 | 2.98E-03 |
| erh | BB071632 | 1.90** | 2.03** | 6.92** | 3.91** |  | 0.62** | 3.56** | 0.84 | 2.17** | 1.02E-02 |
| esr1 | NM_007956 | 0.54* | 0.21** | 1.67* | 1.41 |  | 0.44** | 0.78 | 0.53* | 0.9 | 1.87E-04 |
| fes | BG867327 | 0.99 | 1.22** | 0.73** | 0.88 |  | 1.11 | 2.49** | 0.75** | 3.09** | 1.61E-02 |
| fgf1 | AI649186 | 0.47** | 0.71* | 0.74* | 0.86 |  | 0.74* | 0.72* | 0.97 | 0.86 | 3.69E-02 |
| figf | NM_010216 | 0.83* | 0.89 | 0.92 | 1.21* |  | 0.77** | 0.48** | 0.96 | 1.59** | 7.50E-03 |
| flcn | BC025820 | 0.47** | 0.57* | 1.56 | 0.55** |  | 1.05 | 1.34 | 0.73 | 1.33 | 3.43E-03 |
| frk | BB787292 | 0.60* | 0.34** | 0.46** | 0.81 |  | 0.45** | 0.95 | 0.50** | 0.71 | 1.52E-02 |
| frk | BB787292 | 2.01** | 1.19 | 0.86 | 0.60** |  | 1.07 | 0.95 | 1.13 | 0.96 | 5.57E-03 |
| gadd45b | AK010420 | 0.70* | 0.35** | 2.02** | 0.48** |  | 1.44* | 1.67* | 1.27 | 0.53** | 5.95E-03 |
| gadd45g | AK007410 | 0.59* | 0.41** | 1.88** | 0.59** |  | 4.07** | 5.16** | 3.39** | 2.78** | 8.53E-03 |
| gadd45gip1 | BE368753 | 1.04 | 0.66 | 2.61** | 2.08** |  | 0.47** | 3.26** | 0.44** | 3.77** | 2.37E-02 |
| gas1 | BB550400 | 0.88 | 0.89 | 0.47** | 0.94 |  | 1.08 | 0.41** | 0.93 | 0.39** | 2.07E-03 |
| gmnn | NM_020567 | 1.49* | 3.84** | 7.34** | 5.64** |  | 0.82 | 0.92 | 1.32 | 1.23 | 3.86E-02 |
| gsk3b | BB831420 | 0.88 | 0.45** | 0.63** | 0.57** |  | 1.69** | 0.69** | 0.97 | 0.8 | 4.88E-03 |
| gspt1 | AW537663 | 1.38 | 1.26 | 2.92** | 2.54** |  | 0.49** | 0.95 | 1.22 | 1.2 | 3.43E-03 |
| h2afx | NM_010436 | 1.17 | 2.17** | 4.56** | 1.40** |  | 0.67** | 1.22* | 0.97 | 1.01 | 1.59E-02 |
| hells | NM_008234 | 1.93* | 11.54** | 17.37** | 10.68** |  | 0.38** | 0.57 | 1.24 | 1.33 | 4.92E-02 |
| hells | AK021390 | 1.19 | 5.87** | 2.77** | 3.39** |  | 0.65 | 0.9 | 1.29 | 0.83 | 1.67E-02 |
| hspa8 | BC006722 | 1.09 | 0.69* | 2.37** | 1.87** |  | 0.88 | 2.32** | 0.88 | 2.15** | 2.16E-02 |
| hspa8 | BC006722 | 0.87 | 0.60** | 3.05** | 2.15** |  | 1.04 | 2.47** | 0.9 | 2.47** | 4.06E-04 |
| hspa8 | AK004608 | 3.07** | 3.56** | 0.99 | 1.16 |  | 0.84 | 1.09 | 1.05 | 1.08 | 1.66E-02 |
| htatip2 | AF061972 | 0.84 | 0.40** | 0.74** | 0.64** |  | 0.82 | 0.92 | 0.92 | 1.38** | 1.46E-02 |
| igf2 | NM_010514 | 0.50** | 0.68 | 1.01 | 0.86 |  | 0.83 | 1.01 | 1.01 | 1.02 | 1.60E-02 |
| il1a | BC003727 | 1.06 | 1 | 1.06 | 1.08 |  | 0.46** | 0.78 | 0.44** | 0.31** | 1.33E-02 |
| jag2 | AV264681 | 2.77** | 2.36* | 2.36** | 4.13** |  | 1.24 | 0.74 | 0.99 | 1.85* | 3.18E-02 |
| jag2 | AV264681 | 2.43** | 1.32 | 1.16 | 2.02** |  | 0.69 | 1.33 | 1.03 | 0.9 | 4.06E-02 |
| loh11cr2a | BC004727 | 0.87 | 1.26 | 1 | 1 |  | 0.84 | 0.45** | 1.61** | 0.78* | 1.56E-02 |
| mapk12 | BC021640 | 0.72* | 0.48** | 0.73* | 1.02 |  | 0.66** | 1.28 | 0.8 | 1.35* | 2.74E-02 |
| mapk7 | NM_011841 | 0.94 | 0.40** | 1.17* | 0.99 |  | 0.78* | 0.91 | 0.77** | 1.49** | 4.67E-03 |
| mcm2 | BB699415 | 1.60* | 2.98** | 3.22** | 3.03** |  | 0.84 | 0.59* | 1.60* | 1.23 | 3.50E-02 |
| mcm3 | C80350 | 3.79** | 3.12** | 11.57** | 9.49** |  | 0.73 | 1.02 | 1.09 | 2.04* | 9.57E-03 |
| mcm3 | BI658327 | 1.24 | 2.84** | 2.06** | 1.75** |  | 1.14 | 1.45** | 1.38* | 1.2 | 2.42E-02 |
| mcm4 | BC013094 | 1.63* | 2.68** | 4.14** | 2.51** |  | 0.96 | 1.08 | 0.79 | 2.40** | 7.65E-03 |
| mcm5 | AI324988 | 3.64** | 19.17** | 5.62** | 8.96** |  | 0.68 | 0.66 | 1.6 | 1.25 | 2.01E-02 |
| mcm6 | NM_008567 | 1.46 | 3.63** | 5.64** | 5.10** |  | 0.75 | 0.87 | 1.07 | 2.21* | 2.78E-03 |
| mcm6 | BB099487 | 2.19** | 9.76** | 8.15** | 4.94** |  | 0.50** | 0.63 | 1.12 | 1.11 | 1.97E-02 |
| mcm7 | NM_008568 | 1.66** | 2.59** | 3.84** | 2.38** |  | 0.97 | 1.34* | 1.04 | 1.35* | 5.04E-03 |
| mcm7 | BB464359 | 2.66** | 4.67** | 4.70** | 4.11** |  | 0.87 | 0.84 | 1 | 1.19 | 4.06E-03 |
| mcm7 | BB407228 | 2.00** | 4.75** | 3.07** | 3.07** |  | 0.72* | 0.67* | 1.05 | 0.88 | 3.37E-02 |
| mis12 | BC026790 | 0.92 | 1.32* | 3.00** | 1 |  | 0.85 | 2.05** | 1.03 | 1.78** | 2.98E-02 |
| mphosph9 | BG067775 | 2.41** | 1.36 | 1.52 | 2.01* |  | 1.05 | 1.07 | 0.91 | 0.91 | 3.59E-02 |
| mta3 | NM_054082 | 0.67* | 0.29** | 1.32* | 0.92 |  | 0.84 | 0.63** | 0.89 | 0.70* | 1.69E-02 |
| mtag2 | NM_016664 | 0.67** | 0.43** | 1.2 | 0.65** |  | 0.97 | 0.91 | 1.1 | 0.89 | 1.97E-02 |
| myc | BC006728 | 1.1 | 0.99 | 1.12 | 1.02 |  | 0.32** | 0.65* | 0.53** | 0.99 | 1.15E-02 |
| ncaph | BB725358 | 0.53 | 4.11** | 21.37** | 6.22** |  | 0.65 | 0.64 | 1.26 | 1.06 | 8.00E-03 |
| nek1 | BB418199 | 1.1 | 0.49** | 0.47** | 0.71* |  | 0.64** | 0.63** | 0.59** | 0.85 | 2.03E-03 |
| nek6 | BB528391 | 1.42 | 1.59* | 1.61* | 2.92** |  | 0.89 | 2.08** | 1.22 | 2.15** | 3.94E-03 |
| nfatc1 | NM_016791 | 0.88 | 0.40** | 1.03 | 1.19 |  | 1 | 0.78* | 0.91 | 0.65** | 1.26E-03 |
| nfu1 | BC018355 | 0.91 | 0.37** | 3.44** | 4.99** |  | 1 | 2.22** | 0.9 | 1.53 | 4.34E-02 |
| nipbl | BF661272 | 0.96 | 0.73* | 0.97 | 0.69* |  | 1.01 | 0.49** | 1.17 | 0.71* | 2.62E-02 |
| nr2c2 | AV162817 | 1.08 | 0.43** | 1.21 | 1.1 |  | 0.99 | 0.58** | 0.85 | 0.73* | 6.79E-03 |
| orc2l | BB830976 | 2.08** | 1.91** | 1.61** | 2.41** |  | 1.05 | 1.07 | 1.11 | 1.07 | 3.76E-02 |
| orc2l | BB830976 | 0.83 | 3.83** | 1.70* | 2.46** |  | 1.62* | 0.96 | 2.17** | 0.76 | 1.25E-02 |
| orc4l | BB620704 | 1.01 | 2.01** | 0.72* | 1.49** |  | 0.94 | 1.13 | 0.87 | 1.06 | 1.81E-02 |
| orc6l | NM_019716 | 2.06** | 1.73** | 1.63** | 2.62** |  | 0.7 | 1.11 | 0.87 | 1.24 | 1.94E-03 |
| p42pop | AF364868 | 1.37* | 2.72** | 1.39* | 1.78** |  | 1.09 | 0.9 | 1.3 | 1.36 | 1.15E-02 |
| pard3 | AW543460 | 0.88 | 0.49** | 0.7 | 0.85 |  | 1.16 | 0.44** | 1.03 | 0.73 | 2.02E-02 |
| pard3 | BE199556 | 0.54** | 0.49** | 1.33 | 0.59** |  | 1.1 | 0.55** | 0.74 | 0.83 | 2.43E-02 |
| pard3 | BG063922 | 0.98 | 0.70* | 1.09 | 1.08 |  | 0.75* | 0.44** | 0.69* | 1.09 | 1.75E-02 |
| pard6a | NM_019695 | 0.69** | 0.32** | 1.62** | 0.49** |  | 0.85* | 1.21 | 0.88 | 1.18 | 4.10E-02 |
| pard6b | BE953582 | 0.9 | 1.35 | 0.63 | 0.77 |  | 0.53* | 0.32** | 0.8 | 0.89 | 1.13E-02 |
| pard6b | BE953582 | 0.82 | 0.95 | 0.77 | 0.94 |  | 0.69 | 0.37** | 1.09 | 0.48** | 3.38E-02 |
| pcna | BC010343 | 1 | 2.44** | 17.91** | 4.75** |  | 1.32 | 3.24** | 0.76 | 3.08** | 2.52E-02 |
| pgf | NM_008827 | 0.50** | 0.39** | 1.03 | 0.43** |  | 1.03 | 2.29** | 2.24** | 6.64** | 1.99E-02 |
| pkd1 | NM_013630 | 0.77 | 0.38** | 1.25 | 0.76 |  | 1.15 | 1.3 | 0.49** | 1.67** | 2.95E-02 |
| pola1 | NM_008892 | 1.2 | 2.50* | 5.38** | 1.61 |  | 0.74 | 0.69 | 2.07* | 1.24 | 2.63E-02 |
| polr3d | BC016102 | 0.96 | 1.1 | 1.3 | 1.18 |  | 1.44* | 3.51** | 1.33 | 2.57** | 2.52E-02 |
| ppp1cc | BG071790 | 1.42 | 0.40** | 2.62** | 2.50** |  | 1.01 | 1.07 | 1.37 | 1.91** | 5.34E-03 |
| ppp1r13b | BG064715 | 0.64* | 0.62** | 0.81 | 0.73 |  | 0.70* | 0.47** | 0.8 | 1.01 | 3.81E-03 |
| pstpip1 | U87814 | 0.93 | 1.01 | 1.07 | 1 |  | 1.02 | 2.66** | 1.29 | 2.00** | 3.62E-02 |
| ptprv | NM_007955 | 0.97 | 0.97 | 1.08 | 0.96 |  | 0.51** | 1.26 | 0.92 | 1.98** | 4.71E-02 |
| pycard | BG084230 | 1.33* | 0.64* | 4.64** | 2.06** |  | 0.48** | 1.24 | 0.78 | 1.42* | 6.56E-03 |
| racgap1 | NM_012025 | 1.03 | 1.35 | 8.64** | 2.05* |  | 0.47* | 0.88 | 1.02 | 1.59 | 3.77E-03 |
| rad1 | NM_011232 | 2.12** | 2.27** | 2.13* | 1.58 |  | 0.67 | 3.18** | 0.79 | 1.94* | 2.11E-02 |
| rad50 | NM_009012 | 1.69* | 3.12** | 1.35 | 3.98** |  | 0.98 | 1.33 | 1.09 | 1.2 | 3.03E-02 |
| ran | AV090150 | 1.81** | 1.68** | 2.64** | 2.17** |  | 0.91 | 2.16** | 1.27 | 1.74** | 5.51E-03 |
| rassf2 | AK018504 | 0.77 | 0.99 | 0.68** | 1 |  | 1 | 2.99** | 0.99 | 1.50** | 3.49E-03 |
| rassf4 | AV291679 | 0.40** | 0.25** | 0.72* | 0.55** |  | 0.74 | 0.83 | 0.84 | 1.74** | 8.52E-04 |
| rb1cc1 | BE570980 | 0.87 | 1.07 | 0.62** | 0.61** |  | 1.06 | 0.49** | 1.01 | 0.66** | 3.70E-02 |
| rb1cc1 | BE570980 | 0.93 | 0.37** | 0.62 | 0.75 |  | 0.59 | 0.64 | 0.79 | 0.9 | 1.30E-02 |
| rcc2 | AV122997 | 2.16* | 2.12* | 2.25** | 7.60** |  | 0.68 | 1.71* | 1.43 | 1.76* | 1.64E-04 |
| reck | NM_016678 | 0.35** | 0.54** | 0.9 | 0.86 |  | 0.97 | 0.62* | 0.92 | 0.85 | 1.19E-02 |
| rgs2 | AF215668 | 1 | 0.32** | 2.41** | 1.03 |  | 0.81 | 1 | 0.7 | 1.05 | 2.62E-02 |
| rif1 | AK018316 | 3.82** | 1.57* | 1.65** | 1.97** |  | 0.64* | 0.51** | 1.51* | 0.71* | 2.18E-02 |
| rif1 | AK018316 | 2.25** | 1.16 | 1.44* | 2.50** |  | 1.33* | 0.50** | 1.46** | 0.65** | 2.74E-02 |
| rnf6 | BI738010 | 0.50** | 0.56** | 1.07 | 0.48** |  | 0.89 | 0.86 | 1.28 | 0.81 | 4.03E-02 |
| rnf6 | BI738010 | 0.75* | 0.43** | 1.18 | 0.45** |  | 1.30* | 0.95 | 1.11 | 1.1 | 3.52E-02 |
| ruvbl1 | NM_019685 | 2.34** | 1.74* | 4.19** | 6.62** |  | 0.97 | 2.31** | 1.36 | 2.04** | 4.32E-02 |
| s100a6 | NM_011313 | 0.49** | 0.92 | 1.36* | 1.51** |  | 0.57** | 1.45** | 0.68** | 1.42** | 4.17E-02 |
| sash1 | BI658899 | 0.46** | 0.98 | 0.99 | 0.98 |  | 0.71** | 0.53** | 0.71* | 0.57** | 4.21E-02 |
| Sep-11 | AV229846 | 1.09 | 1.07 | 1.1 | 0.99 |  | 2.05** | 1.05 | 1.43** | 0.89 | 5.01E-03 |
| Sep-09 | NM_017380 | 1.28 | 0.84 | 1.45* | 0.94 |  | 0.78 | 2.10** | 0.94 | 2.03** | 1.53E-02 |
| sesn3 | NM_030261 | 0.78* | 0.49** | 1.15 | 0.73** |  | 1.1 | 0.78* | 1.06 | 1.28* | 1.20E-02 |
| sgol1 | NM_028232 | 1.19 | 2.98** | 11.39** | 2.89** |  | 0.54** | 0.76 | 1.22 | 0.96 | 1.70E-02 |
| siah2 | AA414485 | 0.47** | 0.64** | 1.37** | 0.81 |  | 0.56** | 1.65** | 0.63** | 2.85** | 1.81E-02 |
| skp1a | AV347477 | 1.09 | 1.34** | 1.15 | 1.21* |  | 0.84* | 2.11** | 0.91 | 1.03 | 3.59E-02 |
| skp2 | AV259620 | 2.03* | 2.44** | 3.55** | 1.74* |  | 0.6 | 0.53* | 1.09 | 2.20* | 1.80E-02 |
| skp2 | BB055741 | 2.05** | 2.99** | 4.89** | 3.58** |  | 0.8 | 0.54** | 1.21 | 1.33 | 4.67E-02 |
| skp2 | BB784099 | 2.41** | 1.55 | 2.65** | 2.08* |  | 0.9 | 0.71 | 1.15 | 1.04 | 3.33E-02 |
| skp2 | AV259620 | 2.42** | 3.56** | 6.78** | 1.66* |  | 0.57 | 0.50* | 0.95 | 1 | 9.93E-03 |
| smarcb1 | NM_011418 | 0.95 | 0.95 | 2.41** | 1.51** |  | 0.47** | 1.15 | 0.83 | 2.08** | 4.73E-02 |
| smpd3 | BF456582 | 1.32 | 1.95** | 1.80** | 2.88** |  | 0.87 | 0.92 | 0.93 | 0.86 | 6.24E-03 |
| spc24 | BF577722 | 0.85 | 2.39** | 13.01** | 1.45* |  | 0.50** | 1.01 | 1.12 | 1.15 | 3.12E-03 |
| stat5a | U36502 | 0.56** | 0.40** | 0.82 | 1.01 |  | 0.71* | 0.69* | 0.91 | 0.9 | 3.30E-02 |
| stmn1 | BC010581 | 0.69 | 2.83** | 6.24** | 1.74** |  | 1.04 | 0.91 | 1.02 | 0.93 | 9.57E-03 |
| tfdp1 | BG075396 | 3.08** | 1.70** | 3.28** | 3.13** |  | 1.11 | 1.43* | 1.52* | 1.27 | 4.84E-02 |
| tgfa | M92420 | 0.78 | 0.87 | 1.09 | 0.56** |  | 0.8 | 0.32** | 0.77 | 0.42** | 1.68E-03 |
| tial1 | NM_009383 | 1.06 | 0.97 | 1.97** | 0.76* |  | 0.71** | 2.25** | 0.88 | 2.52** | 1.95E-03 |
| tnfsf5ip1 | BC016606 | 1.16 | 0.92 | 3.22** | 2.64** |  | 0.98 | 3.12** | 1.03 | 1.99** | 5.23E-03 |
| tnfsf5ip1 | NM_134138 | 2.23** | 1.34 | 2.43** | 5.11** |  | 1.21 | 1.42 | 1.25 | 1.15 | 6.53E-04 |
| triap1 | AK007514 | 1.69** | 1.11 | 1.88** | 1.65* |  | 1.01 | 3.43** | 0.88 | 1.99** | 3.77E-02 |
| trp53bp2 | BB814564 | 0.86 | 0.41** | 1.50* | 0.98 |  | 0.61** | 0.79 | 0.81 | 0.92 | 6.57E-03 |
| tubb1 | AW493179 | 0.43** | 0.95 | 1.18 | 0.81* |  | 0.88* | 1.01 | 1.02 | 0.95 | 3.70E-02 |
| tubb5 | BG064086 | 1.08 | 2.68** | 2.13** | 2.17** |  | 0.79 | 1.41* | 1.18 | 1.54* | 1.37E-02 |
| txnl4 | AW552577 | 2.10** | 1.49** | 1.92** | 3.24** |  | 0.98 | 1.43** | 1.05 | 1.72** | 3.49E-02 |
| u2af1 | NM_024187 | 0.86 | 0.67 | 3.05** | 1.84* |  | 0.97 | 2.16** | 1.18 | 1.49 | 3.06E-02 |
| uhrf1 | BB702754 | 1.68 | 7.49** | 7.70** | 3.80** |  | 0.68 | 0.76 | 1.47 | 1.42 | 1.96E-02 |
| unknown | BF471533 | 0.56** | 0.40** | 0.88 | 1.12 |  | 0.76* | 0.64** | 1.32* | 0.92 | 1.40E-02 |
| unknown | BB071777 | 1.44** | 1.24 | 0.78* | 1.58** |  | 0.86 | 0.44** | 0.79* | 0.52** | 2.21E-02 |
| vegfc | BB089170 | 1.18 | 0.69** | 0.60** | 1.72** |  | 0.76* | 0.40** | 0.81 | 0.77* | 3.18E-02 |
| ywhab | NM_018753 | 0.74* | 0.70* | 1.30* | 3.85** |  | 0.83 | 2.17** | 1.05 | 2.43** | 2.39E-02 |
| ywhaq | NM_011739 | 0.67 | 0.63* | 1.94** | 1.18 |  | 1.04 | 3.70** | 0.84 | 1.67* | 3.11E-02 |
| zbtb16 | Z47205 | 0.96 | 0.67* | 0.79 | 0.61* |  | 1.83** | 0.51** | 0.87 | 0.43** | 1.03E-02 |
| zwint | BC013559 | 1.14 | 0.87 | 1.15 | 1.07 |  | 0.60** | 0.48** | 1.17 | 0.74** |  |

**Table S3: Cell growth, DNA metabolism and cytoskeleton maintenance genes showing significant change in expression within 8 hours of MYC-ER^TAM^ activation.**

Genes relating to cell growth, DNA metabolism and cytoskeleton maintenance showing a significant change in expression within 8 hours following activation of MYC-ER^TAM^. ‘MYC-response p-value’ is the p-value identified for the highest-order interaction of the MYC activation variable and represents the significance of this term within the selected model. Flags represent contrast p-values comparing 4OHT-treated and vehicle-treated samples at specific time points (‘*’, p ≤ 0.05; ‘**’ p ≤ 0.01). Cells are colour-coded based on a detected fold-change greater than 1.5-fold (red, up-regulated; blue, down-regulated).

|  |  | **Pancreatic β-cells** | | | |  | **Suprabasal Keratinocytes** | | | | **Myc-response p-value** |
| --- | --- | --- | --- | --- | --- | --- | --- | --- | --- | --- | --- |
| **Gene Symbol** | **RefSeq** | **4 hrs** | **8 hrs** | **16 hrs** | **32 hrs** |  | **4 hrs** | **8 hrs** | **16 hrs** | **32 hrs** |
| 1110002l01rik | BB752416 | 1.11 | 2.02** | 0.89 | 0.87 |  | 1.44* | 0.89 | 1.23 | 0.94 | 4.63E-02 |
| 1110018g07rik | AV257687 | 0.91 | 0.23** | 1.03 | 1.32 |  | 1.03 | 1.04 | 0.94 | 1.19 | 2.27E-02 |
| 1700012g19rik | W59405 | 1.98** | 2.07** | 2.33** | 1.73** |  | 1.08 | 1.32** | 0.98 | 1.05 | 9.57E-03 |
| 1700045i19rik | NM_028842 | 0.37** | 0.42** | 1.61* | 0.96 |  | 0.99 | 0.87 | 1.07 | 0.72 | 5.57E-03 |
| 2310038e17rik | AK009671 | 1.01 | 1.07 | 1.04 | 0.91 |  | 0.47** | 0.40** | 0.9 | 0.48** | 2.49E-02 |
| 2410016o06rik | NM_023633 | 2.05** | 1.36 | 2.20** | 4.76** |  | 0.99 | 2.06** | 1.2 | 1.1 | 3.51E-03 |
| 2410019a14rik | AK010555 | 2.03** | 1.08 | 1.48* | 2.24** |  | 1.11 | 0.95 | 1.44 | 0.88 | 3.98E-03 |
| 2610206b13rik | AK011896 | 1.27 | 2.44** | 2.32** | 3.00** |  | 0.95 | 1.1 | 1.45* | 1.01 | 7.86E-04 |
| 2610209a20rik | AK013127 | 1.75** | 1.23 | 1.06 | 1.86** |  | 0.67* | 2.07** | 0.84 | 1.19 | 1.02E-02 |
| 2810457i06rik | BG076317 | 0.99 | 1.03 | 0.86 | 1.12 |  | 0.81 | 2.13** | 0.85 | 2.99** | 2.69E-02 |
| 4732435n03rik | AV371987 | 0.64* | 0.77 | 0.25** | 0.96 |  | 0.79 | 0.44** | 0.46** | 0.74 | 1.57E-02 |
| 4932441k18rik | BB022577 | 3.79** | 1.28 | 1.82** | 3.84** |  | 1.08 | 0.95 | 0.97 | 1.19 | 1.19E-02 |
| 4933406e20rik | BB306202 | 1.60* | 1.16 | 1.43 | 1.29 |  | 0.57* | 3.37** | 0.69 | 1.35 | 2.73E-03 |
| 6330505n24rik | AV329790 | 0.76** | 0.48** | 1.11 | 0.69** |  | 0.57** | 0.99 | 0.64** | 0.96 | 5.29E-03 |
| 6430704m03rik | BB258560 | 0.45** | 0.50** | 2.36** | 1.1 |  | 0.95 | 0.87 | 1.06 | 0.93 | 2.21E-02 |
| 6530401d17rik | BC016270 | 2.17** | 1.54* | 1.31* | 4.41** |  | 0.56** | 1.79** | 1.70** | 1.17 | 1.25E-04 |
| 8030451f13rik | AV171553 | 1.46** | 1.14 | 1.15 | 0.87 |  | 1.21 | 2.00** | 1.14 | 1.06 | 4.96E-02 |
| 9130227c08rik | BM220028 | 0.54** | 0.59** | 0.72** | 1.05 |  | 0.88 | 0.49** | 0.94 | 0.99 | 9.27E-03 |
| 9630033f20rik | BB278948 | 2.14** | 2.18** | 0.87 | 1.93** |  | 0.92 | 0.82 | 1.42 | 0.63** | 7.05E-03 |
| 9630033f20rik | BB128741 | 1.47* | 2.29** | 1.73** | 2.02** |  | 0.73 | 1.57* | 1.07 | 1.3 | 4.41E-02 |
| abcb1b | NM_011075 | 2.24** | 1.40* | 0.97 | 3.64** |  | 0.8 | 0.58** | 0.88 | 0.96 | 2.46E-02 |
| abcd2 | AW456685 | 0.99 | 0.68* | 0.89 | 1.25 |  | 1.27 | 0.35** | 1.06 | 0.62** | 1.45E-02 |
| abhd4 | NM_134076 | 0.45** | 0.34** | 1.29 | 1.14 |  | 0.68** | 0.91 | 0.71* | 1.09 | 6.90E-04 |
| abhd6 | NM_025341 | 0.77 | 0.62* | 2.29** | 1.22 |  | 0.42** | 1.14 | 0.56** | 1.37 | 2.52E-02 |
| abi2 | BB051811 | 2.05** | 2.14** | 0.8 | 0.86 |  | 1.21 | 0.67 | 1.46 | 0.84 | 1.97E-03 |
| actl6a | NM_019673 | 0.87 | 1.73** | 4.63** | 1.65** |  | 0.76 | 2.36** | 0.85 | 1.94** | 2.59E-02 |
| actr1b | BG801851 | 1.12 | 0.79 | 2.00** | 0.94 |  | 0.94 | 2.37** | 1.24 | 1.81** | 1.46E-02 |
| acyp1 | NM_025421 | 0.87 | 0.35** | 1.81** | 0.94 |  | 0.74* | 1.09 | 0.68** | 0.93 | 1.98E-02 |
| adam3 | NM_009619 | 0.93 | 1.1 | 1.01 | 0.95 |  | 0.83 | 1.92** | 0.55** | 0.37** | 1.27E-02 |
| adcyap1r1 | AK013587 | 0.85 | 0.86 | 0.86 | 1.71** |  | 0.83 | 0.31** | 0.76* | 0.49** | 6.59E-04 |
| add1 | BF140063 | 0.99 | 0.96 | 1.16 | 0.96 |  | 0.85 | 2.57** | 0.86 | 3.93** | 1.94E-02 |
| aff4 | BM230280 | 0.75* | 0.58** | 0.75* | 1.32* |  | 1.03 | 0.44** | 1.18 | 0.85 | 2.41E-02 |
| agpat4 | AK005139 | 0.30** | 0.38** | 1.35 | 0.71 |  | 1.01 | 1.31 | 0.79 | 1.41 | 1.47E-02 |
| agpat5 | BG065500 | 3.38** | 1.83** | 2.54** | 2.10** |  | 0.84 | 0.75* | 1.03 | 1.26 | 1.52E-02 |
| agt | AK018763 | 0.98 | 0.28** | 1.64** | 2.21** |  | 1.17 | 1.06 | 1.14 | 2.08** | 4.69E-02 |
| ahctf1 | BC023122 | 2.23** | 1.28* | 1.34** | 2.77** |  | 0.94 | 0.67** | 1.36** | 0.71** | 1.13E-02 |
| ahctf1 | BC023122 | 2.78** | 1.12 | 1.94** | 2.40** |  | 0.88 | 0.79 | 1.19 | 1.46 | 9.32E-03 |
| ahr | BE989096 | 1.02 | 0.65* | 0.93 | 1.49* |  | 1.14 | 0.45** | 0.89 | 0.49** | 3.14E-02 |
| ai467657 | AA419994 | 0.57** | 1.57* | 1.29 | 0.51** |  | 0.86 | 0.33** | 0.99 | 0.45** | 1.30E-02 |
| akr1b3 | BB469763 | 2.56** | 1.72** | 1.56** | 3.19** |  | 0.85 | 1.06 | 0.98 | 1.2 | 3.82E-02 |
| akr1e1 | BC012692 | 1.43 | 0.28** | 3.14** | 2.01* |  | 0.87 | 1.34 | 0.88 | 3.05** | 2.79E-02 |
| akt1 | NM_009652 | 1.01 | 0.97 | 1.71** | 2.31** |  | 0.82 | 3.23** | 0.95 | 2.29** | 4.43E-02 |
| akt2 | NM_007434 | 1 | 1.05 | 0.98 | 1.13 |  | 1.27 | 2.21** | 1.02 | 2.26** | 7.85E-03 |
| alg13 | NM_026247 | 1.33* | 2.43** | 2.32** | 2.20** |  | 0.54** | 1.27 | 1.01 | 1.45* | 1.74E-02 |
| amd1 | NM_009665 | 2.23** | 1.51** | 1.60** | 2.15** |  | 1.09 | 1.11 | 0.92 | 0.99 | 1.46E-04 |
| amn | NM_033603 | 6.51** | 1.69 | 1.38 | 10.39** |  | 1.37 | 2.43* | 2.91* | 2.51* | 5.19E-04 |
| amph | BQ177140 | 0.43** | 0.51** | 0.98 | 0.68* |  | 0.95 | 0.7 | 1.16 | 0.81 | 6.07E-03 |
| angpt1 | BB453314 | 1.04 | 1.06 | 1.06 | 1.13 |  | 0.40** | 0.50** | 0.44** | 1.22 | 2.13E-03 |
| ankib1 | BE952940 | 1.50** | 2.02** | 1.42** | 1.07 |  | 0.98 | 0.95 | 1.15 | 1.17 | 1.29E-03 |
| ankrd1 | AK009959 | 1 | 0.9 | 1.36 | 1.06 |  | 0.99 | 2.35** | 7.94** | 1.92** | 4.12E-02 |
| apaf1 | AK018076 | 0.54 | 1.06 | 1.04 | 1.12 |  | 0.83 | 0.65 | 0.54* | 1.04 | 2.05E-02 |
| apol3 | AK018646 | 0.97 | 0.97 | 1.08 | 1.18 |  | 1.05 | 0.14** | 0.46** | 0.88 | 1.34E-03 |
| ar | BB148302 | 0.48** | 0.75 | 0.95 | 0.86 |  | 1.04 | 0.58* | 0.97 | 0.68 | 2.83E-03 |
| arpc4 | BG145444 | 0.99 | 1.07 | 1.33** | 1.45** |  | 1.18 | 1.99** | 1.29* | 1.74** | 1.65E-03 |
| arvcf | BE947943 | 1.33 | 1.88** | 0.99 | 1.02 |  | 0.72 | 3.50** | 0.84 | 1.58 | 3.47E-02 |
| arx | BB322201 | 0.49** | 0.59** | 0.40** | 0.55** |  | 0.99 | 0.63** | 0.83 | 1.36* | 4.44E-04 |
| atf5 | AF375476 | 1.05 | 0.66 | 1.57* | 1 |  | 0.92 | 2.94** | 0.88 | 3.18** | 3.70E-02 |
| atf6 | BB129063 | 0.74 | 0.20** | 0.78 | 0.96 |  | 0.8 | 1.53* | 1.04 | 1.14 | 9.54E-03 |
| atp1b1 | AV152334 | 0.47** | 0.47** | 1 | 0.85 |  | 0.93 | 0.71* | 0.75* | 1.07 | 2.07E-02 |
| atp5s | NM_026536 | 0.72* | 0.32** | 1.45* | 0.95 |  | 1.07 | 0.83 | 0.98 | 0.59** | 1.59E-02 |
| atp7a | U03434 | 0.36** | 0.86 | 0.82 | 0.31** |  | 0.51** | 0.46** | 1.45* | 0.66* | 4.62E-02 |
| atrnl1 | AW555641 | 0.98 | 1.02 | 0.64* | 0.81 |  | 1.08 | 0.50** | 0.99 | 0.43** | 1.92E-02 |
| atrx | BB425841 | 1.57** | 1.98** | 1.55** | 1.78** |  | 0.9 | 0.93 | 1.27 | 1.15 | 3.95E-02 |
| b230120h23rik | BB561086 | 1.27 | 1.42 | 1.41 | 1.26 |  | 2.25** | 1.49* | 1.67* | 0.61* | 1.61E-03 |
| b3galnt2 | BB107552 | 2.24** | 1.46 | 1.68* | 3.14** |  | 0.81 | 1.17 | 0.82 | 1.26 | 7.59E-03 |
| b3galnt2 | AI853240 | 2.16** | 1.11 | 1.01 | 1.97** |  | 0.66 | 0.86 | 0.82 | 1.12 | 4.49E-02 |
| bace1 | BB114336 | 1.07 | 0.87 | 0.42** | 0.71* |  | 0.97 | 0.50** | 0.96 | 0.76* | 1.95E-02 |
| bag1 | NM_009736 | 1.38 | 1.46* | 3.04** | 2.00** |  | 0.72 | 2.28** | 1.03 | 1.95** | 3.80E-03 |
| baz1b | BB253608 | 0.93 | 0.66 | 2.51** | 2.06** |  | 1.32 | 3.17** | 1.37 | 1.35 | 3.06E-02 |
| baz2b | AV377356 | 0.97 | 0.44** | 0.86 | 0.94 |  | 0.83 | 0.43** | 1.07 | 0.51** | 3.56E-02 |
| bbs1 | BB121315 | 0.26** | 0.34** | 1.41 | 0.67* |  | 0.67* | 0.52** | 0.98 | 1.01 | 3.64E-02 |
| bc003885 | BC003885 | 2.02** | 2.46** | 2.25** | 2.86** |  | 1.52* | 4.87** | 1.36 | 1.42* | 1.99E-02 |
| bc032204 | BG066664 | 1.93** | 1.11 | 1.55* | 1.60* |  | 0.99 | 2.95** | 0.58** | 5.27** | 5.75E-03 |
| bc037032 | BB532500 | 0.81 | 0.44** | 0.84 | 1.47** |  | 1.16 | 0.89 | 1.31* | 0.66** | 4.06E-02 |
| bc038479 | AV367395 | 0.29** | 0.56** | 1.21 | 0.43** |  | 0.60** | 2.33** | 0.62** | 1.57** | 1.30E-02 |
| bcat1 | X17502 | 1.08 | 3.35** | 0.93 | 3.67** |  | 1.11 | 2.50** | 1.47 | 1.52 | 7.50E-03 |
| bcl2 | BI664467 | 1.23 | 2.27** | 1 | 1.3 |  | 1.31 | 1.21 | 0.99 | 0.93 | 7.79E-03 |
| bdh1 | BF322712 | 1.38 | 1.46 | 0.93 | 2.00* |  | 0.50* | 0.88 | 0.85 | 1.97* | 3.40E-02 |
| bin1 | U60884 | 1 | 0.46** | 2.08** | 1.05 |  | 1.04 | 3.12** | 1.05 | 2.84** | 1.92E-02 |
| bin1 | BG293813 | 0.99 | 0.32** | 0.40** | 0.51** |  | 1.06 | 0.84 | 1.02 | 1.11 | 3.76E-02 |
| birc4 | BF134200 | 0.69* | 0.58* | 0.78 | 0.81 |  | 0.53** | 0.31** | 1.14 | 0.75 | 5.29E-03 |
| blm | NM_007550 | 1.18 | 2.92** | 5.96** | 2.80** |  | 0.71* | 0.68 | 1.13 | 1.01 | 1.38E-03 |
| blvra | AK010847 | 0.75* | 0.57** | 2.07** | 0.96 |  | 1.15 | 1.93** | 1.2 | 2.59** | 4.09E-02 |
| bmf | BB212341 | 1.08 | 0.88* | 0.80* | 1.84** |  | 0.61** | 0.29** | 0.49** | 0.64** | 1.20E-02 |
| bmp1 | L24755 | 0.53** | 0.48** | 1.33 | 0.75 |  | 0.70* | 1.55* | 0.56** | 1.97** | 1.26E-02 |
| bmp2 | AV239587 | 1.1 | 1.39 | 0.64* | 1.11 |  | 0.47** | 0.39** | 0.80* | 0.38** | 8.19E-03 |
| bmp4 | NM_007554 | 0.79 | 0.92 | 1.18 | 1.17 |  | 0.38** | 0.76 | 0.50** | 0.69* | 2.66E-02 |
| bnip1 | BG073508 | 2.06** | 1 | 3.84** | 4.62** |  | 0.60** | 1.96** | 0.78 | 2.40** | 6.79E-03 |
| bop1 | BM213936 | 2.22** | 2.70** | 3.10** | 2.85** |  | 0.8 | 2.42** | 1.14 | 2.44** | 7.54E-03 |
| braf | BG064099 | 1.03 | 0.28** | 0.77 | 0.84 |  | 0.62 | 0.49** | 1.12 | 0.8 | 1.26E-02 |
| brd8 | BM219644 | 0.75 | 0.45** | 1.47* | 0.74 |  | 0.92 | 0.74 | 0.77 | 1.17 | 4.34E-02 |
| brms1 | NM_134155 | 0.96 | 0.91 | 1.19 | 1.42 |  | 1.61 | 3.49** | 1.52 | 1 | 3.70E-02 |
| brms1l | AK003055 | 1.01 | 2.35** | 1.59** | 1.08 |  | 0.84 | 1.14 | 1.17 | 1.16 | 4.85E-02 |
| btf3l4 | AK011367 | 0.44** | 0.49** | 1.22 | 0.96 |  | 0.72** | 1.51** | 0.73** | 1.2 | 2.93E-02 |
| btg1 | L16846 | 0.47** | 0.35** | 1.14 | 0.56** |  | 0.65* | 0.79 | 0.75 | 0.97 | 2.21E-03 |
| btg1 | AW322026 | 0.61** | 0.40** | 0.93 | 0.73** |  | 0.78* | 0.71** | 0.82 | 0.76* | 2.36E-03 |
| bxdc2 | BC014832 | 2.42** | 1.17 | 1.73** | 1.76** |  | 1.02 | 0.86 | 1.34 | 0.96 | 1.56E-02 |
| bzw2 | BM932775 | 4.06** | 2.16** | 4.14** | 4.97** |  | 0.95 | 2.29** | 1.32 | 1.89** | 2.45E-02 |
| c1qtnf6 | AK012868 | 0.69* | 0.92 | 0.73 | 0.91 |  | 0.63* | 3.05** | 0.54** | 5.83** | 6.04E-04 |
| c1qtnf7 | BB039211 | 0.92 | 1.07 | 0.99 | 1.02 |  | 0.59** | 0.30** | 0.65** | 0.51** | 8.64E-03 |
| cad | AK010453 | 2.94** | 1.09 | 2.99** | 4.82** |  | 1.33 | 1.80** | 1.22 | 3.64** | 5.24E-04 |
| cad | AK010453 | 2.02** | 2.28** | 2.28** | 1.92** |  | 1.08 | 1.84** | 1.16 | 1.49* | 1.12E-03 |
| camk1 | NM_133926 | 0.95 | 0.88 | 1.44 | 0.8 |  | 0.82 | 4.62** | 0.69 | 5.19** | 1.10E-02 |
| cant1 | BC020003 | 0.53** | 0.43** | 0.93 | 0.44** |  | 0.99 | 1.41* | 0.98 | 1.1 | 1.56E-02 |
| cap1 | NM_007598 | 4.88* | 0.8 | 1.16 | 1.14 |  | 1.5 | 8.59** | 0.87 | 0.17** | 4.61E-02 |
| capg | BB136012 | 1.02 | 0.96 | 1.05 | 1.17 |  | 1.42** | 0.45** | 0.87 | 1.05 | 1.28E-02 |
| capg | NM_007599 | 1 | 0.68 | 1.34 | 3.16** |  | 0.91 | 1.85* | 0.7 | 2.49** | 1.17E-02 |
| card6 | BB766747 | 0.42** | 0.54** | 0.75* | 0.50** |  | 0.67** | 0.75** | 1.22 | 0.89 | 6.13E-03 |
| cask | Y17137 | 0.93 | 0.86 | 0.68** | 0.99 |  | 0.81 | 2.34** | 0.84 | 2.17** | 1.21E-02 |
| cblb | AW545867 | 1.39* | 0.46** | 0.60* | 1.17 |  | 0.91 | 0.31** | 1.06 | 0.52** | 1.20E-02 |
| ccbl1 | AK008165 | 1.09 | 1.16 | 1.29 | 1.31 |  | 0.68* | 2.41** | 0.88 | 1.54* | 3.53E-02 |
| ccdc6 | AW061011 | 0.68* | 0.46** | 1 | 1.06 |  | 1.37* | 0.66* | 1.14 | 0.84 | 4.25E-02 |
| ccl21a | NM_011335 | 0.98 | 1.17 | 0.94 | 1.78** |  | 1.26 | 3.03** | 0.65** | 1.68** | 2.83E-02 |
| ccnd1 | NM_007631 | 1.86** | 2.09** | 0.88 | 1.45 |  | 1.13 | 1.31 | 1.79* | 1.04 | 3.73E-02 |
| ccnd1 | NM_007631 | 1.69 | 2.38 | 1.41 | 2.27* |  | 1.05 | 1.33 | 1.72 | 1.26 | 4.55E-02 |
| ccnd1 | NM_007631 | 3.41** | 2.74* | 2.03* | 2.05 |  | 1.35 | 0.99 | 1.54 | 1.81 | 2.24E-02 |
| ccnd2 | NM_009829 | 2.06** | 2.04** | 1.27 | 2.30** |  | 0.9 | 0.84 | 1.29 | 0.84 | 2.62E-02 |
| ccnd2 | AK007904 | 1.99** | 0.64* | 1.88** | 2.62** |  | 1.66* | 2.57** | 1 | 2.13** | 1.48E-02 |
| ccnd3 | NM_007632 | 1.26* | 0.84 | 1.54** | 1.2 |  | 0.81* | 2.58** | 0.84 | 3.33** | 1.21E-02 |
| cd209b | AF374471 | 0.86 | 0.74* | 0.73** | 1.30* |  | 1.09 | 1.95** | 0.33** | 1.18 | 7.49E-03 |
| cd99l2 | BB334959 | 0.28 | 0.35 | 0.87 | 0.17* |  | 0.17* | 0.38 | 1.33 | 0.43 | 9.62E-03 |
| cd99l2 | BB334959 | 0.58 | 0.41 | 1.28 | 0.39* |  | 0.36* | 0.5 | 2.45 | 0.47 | 3.18E-02 |
| cdc23 | BB492440 | 0.9 | 0.43** | 1.01 | 1.47** |  | 1.13 | 1 | 0.82* | 1.18 | 1.18E-02 |
| cdc2a | NM_007659 | 0.95 | 2.91** | 11.93** | 3.35** |  | 0.51* | 1.17 | 0.89 | 1.12 | 2.52E-02 |
| cdc42bpa | BG518726 | 0.70** | 0.49** | 1.03 | 0.88 |  | 1.02 | 1.31* | 1.14 | 1 | 1.84E-02 |
| cdc42ep4 | NM_020006 | 0.64** | 0.51** | 1.24 | 0.96 |  | 0.53** | 1.13 | 0.67* | 0.78 | 1.57E-02 |
| cdc73 | BM935271 | 0.99 | 1.24 | 0.81 | 0.72** |  | 0.92 | 0.40** | 1.03 | 0.52** | 3.73E-02 |
| cdk4 | NM_009870 | 1.16 | 0.92 | 8.41** | 1.11 |  | 1.32 | 12.27** | 0.74 | 1.62 | 1.02E-02 |
| cdk4 | NM_009870 | 1.21 | 0.74 | 5.31** | 1.5 |  | 1.23 | 11.30** | 0.92 | 1.48 | 1.45E-02 |
| cdk4 | NM_009870 | 1.2 | 0.88 | 5.03** | 1.3 |  | 1.37 | 11.17** | 0.72 | 1.37 | 4.33E-02 |
| cdk5r1 | BB177836 | 0.74 | 0.58** | 1.07 | 0.58* |  | 0.87 | 0.45** | 1.46 | 0.84 | 4.49E-02 |
| cdkn1a | AK007630 | 1.57** | 2.05** | 1.71** | 1.24 |  | 0.96 | 1.44* | 0.99 | 0.9 | 5.01E-03 |
| cdkn1b | NM_009875 | 1.03 | 0.19** | 0.71 | 0.95 |  | 0.23** | 0.42* | 0.48* | 0.89 | 1.02E-02 |
| cdkn2a | NM_009877 | 2.17** | 1.79** | 1.57** | 2.61** |  | 1.21 | 1.07 | 1.07 | 0.87 | 8.43E-03 |
| cds1 | AK014670 | 0.52** | 0.36** | 0.96 | 0.52** |  | 0.86 | 0.91 | 0.87 | 0.85 | 1.89E-02 |
| cenpj | BG068259 | 0.86 | 0.43** | 1.53* | 0.68* |  | 0.91 | 1 | 0.79 | 1.44* | 1.88E-02 |
| cenpk | NM_021790 | 1.2 | 4.44** | 22.14** | 3.08** |  | 0.60** | 0.70* | 1.37* | 1.2 | 2.35E-02 |
| cep68 | AW550283 | 2.19** | 1.82** | 0.85 | 2.37** |  | 1.19* | 0.77** | 1.20* | 1.12 | 4.75E-02 |
| cfl1 | NM_007687 | 1.29 | 0.51** | 1.3 | 1.89** |  | 1.03 | 2.28** | 1.15 | 2.14** | 4.26E-02 |
| cgref1 | BC023116 | 0.97 | 1.50** | 0.84 | 1.21 |  | 0.66** | 2.23** | 0.78* | 2.39** | 9.67E-03 |
| cgrrf1 | AV305616 | 0.67** | 0.42** | 1.62** | 0.57** |  | 1.05 | 1.33** | 1.14 | 1.15 | 2.29E-02 |
| cgrrf1 | AK004156 | 0.49** | 0.60* | 2.01** | 1.78** |  | 1.04 | 0.71 | 0.92 | 0.8 | 3.04E-02 |
| chm | NM_018818 | 0.49** | 0.59** | 1.43* | 0.60** |  | 0.63** | 0.69** | 1.12 | 0.88 | 4.91E-02 |
| ciao1 | AK004129 | 0.89 | 0.92 | 1.55* | 1.58 |  | 1.26 | 3.70** | 1.07 | 1.31 | 3.56E-02 |
| ckap4 | BB312117 | 1.37 | 1.57** | 1.01 | 0.95 |  | 0.37** | 2.22** | 0.79 | 3.30** | 3.23E-02 |
| clic5 | BB236747 | 0.51** | 0.44** | 0.54* | 1.21 |  | 1.19 | 1.51 | 1.24 | 1.71* | 3.05E-02 |
| cnbp | BM237919 | 2.02** | 1.32 | 0.84 | 2.01** |  | 1.16 | 1.51 | 0.86 | 0.91 | 3.69E-02 |
| cnn3 | AI314104 | 0.96 | 0.50** | 1.88** | 1.14 |  | 0.85 | 1.02 | 0.76* | 1.16 | 4.39E-02 |
| cnot4 | BB756908 | 1.73** | 0.51** | 1.12 | 1.36** |  | 0.83 | 1.3 | 1 | 1.32* | 2.03E-02 |
| cnot7 | AK007767 | 2.15* | 1.25 | 1.61* | 0.95 |  | 0.87 | 2.44** | 0.9 | 3.82** | 3.82E-02 |
| cops5 | NM_013715 | 1.01 | 0.89 | 2.65** | 1.3 |  | 0.92 | 1.98** | 0.94 | 1.69** | 3.35E-02 |
| cotl1 | NM_028071 | 0.77 | 0.63** | 1.22 | 1.85** |  | 0.89 | 2.31** | 0.77 | 6.37** | 3.09E-03 |
| cox10 | AK010385 | 1.81** | 0.91 | 1.61* | 2.24** |  | 0.96 | 2.51** | 0.68 | 1.55* | 8.49E-03 |
| cox10 | BG073490 | 2.18** | 1.18 | 1.1 | 2.77** |  | 0.8 | 0.78 | 0.84 | 1.19 | 3.06E-02 |
| cplx2 | NM_009946 | 0.45* | 0.17** | 0.15** | 0.43** |  | 2.15* | 0.73 | 1.17 | 0.87 | 1.64E-02 |
| cplx2 | BE946238 | 0.73** | 0.47** | 0.82** | 0.64** |  | 0.92 | 0.93 | 0.77** | 0.98 | 2.04E-02 |
| cpm | AK017670 | 0.44** | 0.23** | 1.08 | 0.62* |  | 0.69 | 0.43** | 0.65* | 0.71 | 2.13E-05 |
| cpm | AK004327 | 0.59* | 0.24** | 1.35 | 1.01 |  | 0.54** | 0.41** | 0.48** | 0.8 | 3.58E-02 |
| cpt1a | BB021753 | 0.66** | 0.35** | 0.60** | 0.80* |  | 0.73** | 0.61** | 1.32* | 0.80* | 6.62E-03 |
| cpt1a | BB119196 | 0.54** | 0.38** | 0.48** | 0.87 |  | 0.67* | 0.58** | 1.18 | 0.75 | 5.99E-03 |
| creg1 | BC027426 | 2.08** | 2.12** | 1.60** | 2.16** |  | 0.69* | 0.85 | 0.98 | 0.92 | 3.68E-02 |
| cryab | NM_009964 | 1.06 | 1.13 | 1.08 | 1.47 |  | 0.96 | 4.03** | 1.09 | 1.80** | 8.20E-03 |
| cspg5 | NM_013884 | 2.23** | 0.81 | 1.24 | 1.81* |  | 1.01 | 0.89 | 1.26 | 0.89 | 2.00E-02 |
| ctgf | NM_010217 | 0.54* | 1.15 | 0.62 | 1.22 |  | 1.26 | 0.55* | 0.84 | 0.68 | 3.37E-03 |
| ctnna1 | NM_009818 | 0.47** | 0.56** | 1.52** | 0.9 |  | 0.78* | 0.84 | 0.88 | 1.04 | 3.73E-02 |
| ctnnb1 | BI134907 | 1.11 | 1.04 | 1.01 | 0.92 |  | 1.22 | 2.36** | 1.04 | 1.54** | 3.46E-02 |
| ctr9 | BG079166 | 1.24 | 0.44** | 0.46** | 1.15 |  | 0.83 | 0.43** | 0.92 | 0.88 | 2.23E-03 |
| cttnbp2 | BB357580 | 1.35* | 0.54** | 1.54** | 1.41* |  | 0.68** | 0.33** | 0.69* | 0.45** | 6.43E-03 |
| cutl1 | BC014289 | 1.04 | 1.07 | 1.18* | 0.92 |  | 0.64** | 0.49** | 1.01 | 0.84** | 2.42E-02 |
| cyba | AK018713 | 1.33 | 0.86 | 3.06** | 9.59** |  | 1.44 | 4.50** | 0.44** | 3.15** | 8.01E-03 |
| cycs | NM_007808 | 1.17* | 1.98** | 1.55** | 2.05** |  | 0.83* | 1 | 1.17 | 1.05 | 1.64E-03 |
| cyfip2 | AK005148 | 0.77 | 0.51** | 2.27** | 1.02 |  | 0.37** | 0.43** | 0.56** | 0.92 | 9.16E-03 |
| cyp4f16 | AK009445 | 0.44** | 1.03 | 1.09 | 1 |  | 0.65** | 1.51** | 1.11 | 1.44** | 7.93E-03 |
| d15wsu169e | AI843066 | 2.63** | 1.51* | 0.97 | 1.16 |  | 0.95 | 0.75 | 1.3 | 0.89 | 2.66E-02 |
| daam1 | AW988556 | 0.7 | 0.50** | 1.16 | 0.63 |  | 1.75* | 0.38** | 1.57 | 0.54* | 3.86E-02 |
| daam1 | BB794633 | 0.34** | 0.86 | 1.03 | 0.55** |  | 0.67** | 0.61** | 0.91 | 0.68* | 2.30E-02 |
| dab2 | NM_023118 | 0.88 | 0.48* | 4.13** | 2.64** |  | 1.74* | 3.75** | 1.31 | 3.85** | 3.79E-03 |
| dab2 | BC006588 | 0.8 | 0.81 | 1.53 | 1.23 |  | 1.27 | 2.75** | 0.82 | 2.22** | 6.40E-03 |
| dab2 | AK017619 | 1.31 | 0.76 | 0.96 | 1.16 |  | 1.41 | 3.55** | 0.58* | 2.62** | 2.93E-02 |
| dach1 | BB374930 | 1.85** | 0.24** | 0.36** | 0.77 |  | 0.84 | 0.43** | 0.68* | 0.62** | 8.16E-04 |
| dach2 | NM_033605 | 0.39** | 1.94** | 2.64** | 0.88 |  | 1 | 1.01 | 1.25 | 0.73* | 3.53E-03 |
| daglb | BC016105 | 1.46** | 1.91** | 0.83 | 1.57** |  | 0.91 | 0.52** | 1.07 | 0.82 | 2.70E-02 |
| dbnl | AV328035 | 1.17 | 0.86 | 1.4 | 1.82* |  | 0.93 | 2.26** | 0.87 | 1.46 | 3.38E-02 |
| dck | BB030204 | 1.54* | 2.91** | 1.73** | 1.56* |  | 1 | 0.62* | 1.15 | 1.50* | 2.13E-02 |
| dctd | BG069699 | 4.97** | 3.52** | 3.51** | 4.98** |  | 1.17 | 1.44 | 1.61 | 1.36 | 2.59E-05 |
| ddef1 | BG064109 | 1.01 | 1.04 | 1.03 | 1.04 |  | 1.2 | 2.13** | 1.48** | 1.05 | 4.58E-02 |
| ddx56 | BC018291 | 1.08 | 0.42** | 1.77** | 2.15** |  | 1.3 | 2.05** | 0.87 | 2.03** | 1.37E-02 |
| deaf1 | NM_016874 | 2.03* | 0.9 | 1.79** | 3.22** |  | 0.91 | 2.24** | 0.55* | 1.32 | 4.16E-02 |
| decr2 | BE952632 | 0.23** | 0.12** | 0.64* | 0.60* |  | 0.86 | 0.58* | 0.49* | 0.96 | 6.06E-05 |
| dek | AK007546 | 1.83** | 2.01** | 2.68** | 2.25** |  | 0.81 | 0.59** | 1.04 | 0.72 | 4.41E-03 |
| dennd2a | AV260555 | 0.87 | 0.95 | 1.01 | 1.06 |  | 1.26* | 2.32** | 0.9 | 2.80** | 3.69E-02 |
| dhcr7 | NM_007856 | 2.28** | 2.57** | 1.26* | 1.54** |  | 0.50** | 0.49** | 0.83 | 1.39* | 2.52E-02 |
| dhrs13 | AK011939 | 2.44** | 1.69** | 3.88** | 1.34 |  | 0.9 | 1.35* | 1.32 | 1.09 | 4.64E-02 |
| dip2a | BB794700 | 0.56** | 0.30** | 0.81 | 0.49** |  | 1.05 | 0.74 | 1.03 | 0.63** | 6.53E-04 |
| dkc1 | BG068512 | 2.23** | 1.85** | 1.80** | 2.08** |  | 1.02 | 1.28 | 0.95 | 2.13** | 1.21E-03 |
| dkc1 | BB779105 | 4.08** | 2.39** | 0.64 | 3.58** |  | 0.79 | 0.47** | 1.70* | 0.56* | 5.85E-03 |
| dlc1 | BB768194 | 0.44** | 0.59** | 0.81 | 0.48** |  | 0.9 | 1.03 | 1.08 | 0.8 | 1.04E-02 |
| dleu2 | BB812902 | 0.73 | 0.24** | 0.94 | 1.02 |  | 0.97 | 0.74 | 0.62* | 0.81 | 1.81E-02 |
| dlg5 | BC021314 | 0.70* | 0.44** | 0.62** | 0.89 |  | 0.8 | 0.64** | 0.88 | 0.82 | 3.39E-03 |
| dmd | NM_007868 | 1.29* | 1.01 | 1.11 | 1.98** |  | 0.83 | 0.46** | 0.87 | 0.92 | 2.76E-02 |
| dnahc1 | BB497449 | 0.98 | 0.92 | 0.96 | 1.02 |  | 0.46** | 0.46** | 0.72** | 0.75** | 2.20E-02 |
| dner | AF370126 | 0.67* | 0.27** | 0.25** | 0.78 |  | 1.2 | 1.01 | 0.95 | 0.93 | 4.26E-02 |
| dnm3 | BE988832 | 0.7 | 0.20** | 1.06 | 0.58* |  | 0.60* | 0.50** | 0.96 | 1.1 | 4.82E-02 |
| dnmt1 | NM_010066 | 1.02 | 2.39** | 2.99** | 1.35* |  | 0.67** | 0.91 | 1.02 | 1.55** | 1.62E-02 |
| dnmt3a | BB795491 | 1.13 | 0.38** | 0.68** | 0.60** |  | 1.08 | 0.60** | 0.72** | 0.72** | 2.82E-03 |
| dnmt3b | NM_010068 | 2.26** | 1.75** | 0.87 | 1.1 |  | 0.89 | 0.72** | 1.15 | 0.51** | 3.45E-02 |
| drg1 | BM506525 | 1.54** | 2.23** | 1.21 | 1.35** |  | 1.02 | 1.44** | 1.13 | 0.91 | 1.18E-02 |
| drg1 | AV127994 | 1.80** | 2.27** | 1.42** | 1.53** |  | 0.73* | 1.18 | 1.46** | 0.99 | 2.23E-04 |
| drg1 | NM_007879 | 1.18 | 1.37* | 4.20** | 2.41** |  | 0.82 | 2.50** | 0.94 | 1.78** | 2.45E-02 |
| drg2 | NM_021354 | 1.76** | 1.31 | 1.89** | 2.19** |  | 0.83 | 2.28** | 1.12 | 1.25 | 4.32E-02 |
| dsc2 | BC004663 | 0.35** | 0.57** | 1.19 | 0.78 |  | 0.79 | 0.63* | 1.06 | 0.65* | 6.61E-03 |
| dsg1b | AV253195 | 1.07 | 1.43* | 0.83 | 0.93 |  | 0.32** | 0.45** | 0.92 | 0.72 | 1.10E-02 |
| dtd1 | AI451865 | 1.02 | 1.43* | 1.43** | 1.13 |  | 0.95 | 2.27** | 1.36 | 1.25 | 4.58E-02 |
| dut | AF091101 | 1.95** | 1.18 | 2.25** | 2.97** |  | 1.38 | 0.95 | 1.02 | 0.70* | 4.34E-02 |
| dynlrb2 | AK005789 | 0.9 | 0.45** | 0.97 | 0.89 |  | 0.98 | 1.07 | 0.97 | 0.93 | 2.81E-03 |
| dynlt1 | BG093881 | 0.43* | 0.44** | 1.42 | 1.47 |  | 1.61 | 0.39** | 0.42* | 1.49 | 1.81E-03 |
| dzip1 | AI509011 | 0.49** | 0.79 | 0.89 | 0.98 |  | 0.89 | 0.98 | 0.77 | 1.35* | 2.60E-02 |
| e2f3 | BQ176318 | 2.47** | 1.42 | 1.12 | 1.58* |  | 1.35 | 0.83 | 1.74** | 0.74 | 4.65E-02 |
| ears2 | AK014324 | 0.50** | 0.48** | 1.15 | 0.52** |  | 0.77 | 2.26** | 0.84 | 1.60* | 2.45E-02 |
| ebf1 | BB364548 | 1 | 0.99 | 0.99 | 1.05 |  | 1.5 | 2.61** | 0.86 | 1.99** | 4.00E-02 |
| ebf1 | BB038386 | 1.03 | 1.01 | 0.49** | 1.50* |  | 1.63** | 2.40** | 0.74 | 2.80** | 7.19E-03 |
| ebf3 | AK014058 | 0.96 | 1.03 | 0.76* | 1.1 |  | 0.81 | 0.48** | 0.93 | 0.85 | 1.81E-02 |
| ebf3 | AK014058 | 0.93 | 0.88 | 1.21 | 0.97 |  | 0.54** | 0.45** | 1.02 | 1.36 | 2.19E-02 |
| ebna1bp2 | AK007491 | 2.41** | 0.96 | 2.55** | 2.68** |  | 1.08 | 1.45 | 1.56 | 1.38 | 3.53E-02 |
| ecsit | NM_012029 | 2.00** | 1.37 | 3.35** | 3.49** |  | 0.94 | 2.74** | 1.22 | 2.29** | 1.12E-03 |
| ednra | AW558570 | 1.05 | 0.79* | 0.75* | 1.38** |  | 0.93 | 0.43** | 0.81* | 0.46** | 2.04E-02 |
| ednrb | BB451714 | 0.64* | 0.47** | 1.14 | 0.95 |  | 1.4 | 4.30** | 1.13 | 3.05** | 2.54E-02 |
| ednrb | BF100813 | 1.07 | 0.49** | 1.51 | 1.11 |  | 1.17 | 1 | 1.53* | 2.52** | 1.29E-02 |
| eef1e1 | NM_025380 | 3.22** | 2.14** | 7.58** | 7.88** |  | 1.4 | 3.42** | 1.52* | 1.63** | 4.05E-02 |
| efhc1 | AK006489 | 0.58** | 0.41** | 0.97 | 0.63** |  | 1.01 | 0.77* | 0.95 | 0.68** | 1.06E-03 |
| efhd2 | AK007560 | 0.76 | 0.44** | 2.51** | 2.03** |  | 0.60* | 1.68* | 1.34 | 5.72** | 8.01E-03 |
| efnb1 | NM_010110 | 0.59** | 0.70** | 0.62* | 0.52** |  | 0.40** | 0.50** | 0.98 | 1.08 | 2.07E-02 |
| egf | NM_010113 | 1.05 | 0.79 | 0.92 | 1.38* |  | 1.62** | 0.47** | 0.91 | 0.84 | 1.28E-02 |
| egfr | AV369812 | 0.74* | 0.46** | 0.86 | 0.99 |  | 1.13 | 0.39** | 1.33* | 0.47** | 2.96E-02 |
| egr3 | AV346607 | 1.06 | 1.24 | 2.20** | 1.15 |  | 0.68** | 0.39** | 0.84 | 0.40** | 2.32E-02 |
| elf5 | BC012424 | 1 | 0.97 | 1.2 | 1.06 |  | 0.50** | 0.19** | 0.65* | 1.35 | 4.14E-03 |
| elk3 | BC005686 | 0.62* | 0.36** | 0.60* | 0.71 |  | 0.69* | 0.87 | 0.82 | 1.44 | 3.52E-02 |
| elmo3 | AI481208 | 1.16 | 0.28** | 0.77 | 0.47** |  | 0.53** | 0.82 | 0.68* | 0.82 | 2.12E-02 |
| emg1 | NM_013536 | 1.1 | 1.01 | 2.29** | 1.4 |  | 0.91 | 2.09** | 1.01 | 1.46* | 4.16E-02 |
| eml4 | AK019611 | 2.17** | 1.89** | 1.05 | 2.54** |  | 1.91** | 1.2 | 2.36** | 0.76 | 6.10E-05 |
| enpp5 | BC011294 | 1.12 | 0.69* | 1.78** | 1.13 |  | 0.76* | 2.84** | 0.65** | 1.25 | 2.95E-02 |
| ensmusg00000075401 | BQ175722 | 1.16 | 0.70* | 0.75 | 0.99 |  | 0.95 | 0.42** | 1.08 | 0.58** | 2.52E-02 |
| epb4.1l3 | AF177146 | 1.2 | 0.23** | 1.25 | 1.33 |  | 0.66 | 0.86 | 0.74 | 0.58* | 2.39E-02 |
| epb4.1l4a | NM_013512 | 0.87 | 0.34** | 1.73** | 2.52** |  | 0.93 | 0.68* | 0.84 | 0.61** | 3.81E-02 |
| erbb3 | BF140685 | 0.45** | 0.51** | 0.59* | 1.5 |  | 0.87 | 0.44** | 0.7 | 0.43** | 1.48E-02 |
| ereg | NM_007950 | 0.88 | 1.02 | 1.07 | 0.87 |  | 0.17** | 0.27** | 0.58** | 0.88 | 7.23E-03 |
| esr1 | NM_007956 | 0.54* | 0.21** | 1.67* | 1.41 |  | 0.44** | 0.78 | 0.53* | 0.9 | 1.02E-02 |
| etv1 | NM_007960 | 0.47** | 0.61** | 0.43** | 0.71* |  | 1.03 | 1.02 | 1.04 | 1.68** | 1.83E-02 |
| exo1 | BE986864 | 0.95 | 2.17** | 3.81** | 2.36** |  | 0.94 | 1.12 | 1.28 | 1.2 | 1.20E-02 |
| eya1 | BB760085 | 0.71* | 0.40** | 1.64** | 1.08 |  | 1.63** | 0.58** | 1.22 | 0.92 | 1.71E-02 |
| f2r | BQ173958 | 1.21 | 0.83 | 0.71 | 1.13 |  | 0.64* | 0.42** | 0.91 | 0.52** | 1.33E-02 |
| fahd1 | BC026949 | 1.35 | 0.99 | 2.23** | 2.41** |  | 1.27 | 2.34** | 1.11 | 1.97** | 4.89E-02 |
| fahd2a | BI872590 | 1.07 | 0.49** | 2.24** | 1.19 |  | 1.01 | 1.80** | 1.07 | 1.28 | 2.98E-02 |
| fancc | BE952454 | 0.48** | 0.75** | 1.42** | 0.88 |  | 0.89 | 1.15 | 0.95 | 1.59** | 2.36E-02 |
| farp2 | BC009153 | 2.18** | 0.9 | 0.96 | 1.25 |  | 0.89 | 1.42 | 0.85 | 1.25 | 4.41E-02 |
| farsb | AK012154 | 3.12** | 1.99** | 0.99 | 0.58* |  | 1.47 | 0.63* | 1.24 | 0.7 | 3.21E-02 |
| fat4 | BB536078 | 1.09 | 1 | 0.73* | 1.03 |  | 0.55** | 0.28** | 0.39** | 0.45** | 2.16E-02 |
| fcer1g | NM_010185 | 1.05 | 1.42* | 1.76* | 1.59* |  | 0.89 | 3.51** | 0.9 | 4.30** | 1.86E-02 |
| fes | BG867327 | 0.99 | 1.22** | 0.73** | 0.88 |  | 1.11 | 2.49** | 0.75** | 3.09** | 1.87E-04 |
| fgf1 | AI649186 | 0.47** | 0.71* | 0.74* | 0.86 |  | 0.74* | 0.72* | 0.97 | 0.86 | 1.61E-02 |
| fgfr1op2 | BB435465 | 1.18 | 0.52** | 0.49** | 1.1 |  | 0.58** | 0.48** | 0.46** | 1.80** | 8.01E-03 |
| figf | NM_010216 | 0.83* | 0.89 | 0.92 | 1.21* |  | 0.77** | 0.48** | 0.96 | 1.59** | 3.69E-02 |
| flii | NM_022009 | 0.82 | 0.16** | 1.56* | 0.77 |  | 0.60** | 1.41 | 0.64* | 1.89** | 2.68E-02 |
| flna | BM233746 | 0.86 | 0.87 | 1.85** | 2.31** |  | 0.54** | 2.27** | 0.8 | 4.01** | 4.96E-02 |
| fmn2 | BM228488 | 0.41** | 0.21** | 1.34 | 0.37** |  | 0.97 | 0.82 | 1.1 | 1.02 | 5.04E-03 |
| fmo2 | BM936480 | 0.43** | 1.17 | 0.83 | 1.79* |  | 0.34** | 0.79 | 0.27** | 0.65 | 6.24E-03 |
| frem1 | BI452538 | 0.95 | 1.19 | 0.71* | 0.91 |  | 0.69** | 0.47** | 0.71** | 0.75* | 1.21E-03 |
| frmd4b | BG067753 | 0.42** | 0.82 | 0.73 | 1.04 |  | 0.84 | 0.8 | 0.78 | 0.86 | 4.56E-02 |
| fuca2 | BM054266 | 0.65** | 0.41** | 0.94 | 0.62** |  | 0.54** | 0.73* | 0.79 | 1.19 | 4.53E-04 |
| fzd3 | AU043193 | 0.49** | 0.87 | 1.31 | 0.78 |  | 1.24 | 1.3 | 0.77 | 0.92 | 2.89E-02 |
| fzd6 | NM_008056 | 1.07 | 0.41** | 0.65* | 0.93 |  | 0.93 | 0.53** | 1.06 | 0.64** | 3.23E-02 |
| gadd45b | AK010420 | 0.70* | 0.35** | 2.02** | 0.48** |  | 1.44* | 1.67* | 1.27 | 0.53** | 5.57E-03 |
| gadd45g | AK007410 | 0.59* | 0.41** | 1.88** | 0.59** |  | 4.07** | 5.16** | 3.39** | 2.78** | 5.95E-03 |
| gal | NM_010253 | 0.96 | 0.98 | 1.27 | 1.38* |  | 0.82 | 3.49** | 2.43** | 4.56** | 1.21E-02 |
| gas6 | NM_019521 | 0.60** | 0.73** | 1.02 | 0.9 |  | 1.39** | 2.61** | 1.43** | 5.62** | 2.31E-02 |
| gcat | AK013138 | 1.3 | 2.20** | 1.29* | 1.78** |  | 0.83 | 1.47** | 0.88 | 1.03 | 1.56E-02 |
| gcdh | NM_008097 | 0.91 | 0.96 | 2.89** | 1.94** |  | 0.66* | 2.36** | 0.91 | 1.18 | 1.05E-02 |
| gdpd1 | AK016023 | 0.9 | 0.83 | 0.91 | 1.04 |  | 0.59** | 0.45** | 0.66** | 0.52** | 3.65E-03 |
| gdpd1 | AK016023 | 0.53* | 0.54* | 1.84* | 1.14 |  | 0.17** | 0.7 | 0.63 | 0.38** | 1.65E-02 |
| gemin8 | BC023488 | 2.24** | 1.52** | 1.52** | 1.72** |  | 0.76 | 0.96 | 1.08 | 1.23 | 1.63E-03 |
| gja5 | AK017840 | 0.86 | 0.36** | 1.22 | 1.39* |  | 0.81 | 1 | 1.25 | 0.86 | 2.43E-02 |
| glis2 | NM_031184 | 0.52** | 0.45** | 0.76* | 0.67 |  | 1.01 | 0.79 | 0.69 | 1.12 | 1.97E-03 |
| glo1 | BC024663 | 0.74 | 0.76 | 1.23 | 0.81 |  | 1 | 0.38** | 1.19 | 1.11 | 3.94E-02 |
| glo1 | BC024663 | 0.72 | 0.64** | 1.24 | 0.98 |  | 1.15 | 0.34** | 1.23 | 0.75 | 2.63E-02 |
| gmip | BB474868 | 7.06** | 4.17** | 2.05** | 6.46** |  | 0.81 | 1.78** | 0.78 | 1.52* | 1.95E-02 |
| gmppa | NM_133708 | 0.50** | 0.46** | 2.54** | 0.8 |  | 0.70* | 1.84** | 0.88 | 1.41 | 2.61E-02 |
| gmppa | AU044197 | 0.53** | 0.39** | 2.06** | 0.78 |  | 0.89 | 1.50** | 1.03 | 1.51** | 2.38E-02 |
| gmppa | AU044197 | 0.57** | 0.38** | 1.92** | 0.87 |  | 0.79* | 1.67** | 0.94 | 1.49** | 4.40E-02 |
| gna13 | BI662324 | 0.94 | 0.47** | 0.40** | 0.82 |  | 0.89 | 0.68 | 0.82 | 0.47** | 4.42E-02 |
| gnl2 | BI666155 | 3.09** | 1.1 | 1.26 | 0.27** |  | 0.8 | 0.79 | 0.9 | 1.79* | 3.54E-02 |
| gnpnat1 | AK008566 | 1.80** | 3.60** | 0.9 | 1.11 |  | 0.71* | 1.23 | 1.01 | 1.04 | 4.83E-02 |
| gnptab | BG144467 | 5.26** | 0.84 | 1.41* | 5.60** |  | 0.9 | 0.58* | 0.76 | 0.87 | 2.82E-03 |
| golga3 | D78270 | 0.64* | 0.46** | 1.2 | 0.57** |  | 0.86 | 1.07 | 1.03 | 1.03 | 3.07E-02 |
| gpd1l | BB727537 | 3.99** | 0.72 | 1.84* | 6.60** |  | 1.09 | 1.22 | 0.87 | 1.13 | 3.74E-02 |
| gpd2 | BQ175968 | 0.55* | 0.39** | 0.73 | 0.61* |  | 1.03 | 0.98 | 0.74 | 0.73 | 5.18E-03 |
| gpsm1 | BC026486 | 0.74 | 0.46** | 1.72** | 0.84 |  | 0.42** | 0.95 | 0.81 | 0.68* | 3.49E-02 |
| grit | BI408524 | 1.23 | 0.70** | 1.05 | 0.72** |  | 0.64** | 0.47** | 0.92 | 0.68** | 3.21E-02 |
| gsk3b | BB831420 | 0.88 | 0.45** | 0.63** | 0.57** |  | 1.69** | 0.69** | 0.97 | 0.8 | 3.86E-02 |
| gsr | AK019177 | 1.76** | 2.73** | 1.44** | 2.18** |  | 1.18 | 1.2 | 1.31 | 1.25 | 9.46E-03 |
| gsto2 | AK019582 | 0.27** | 0.84 | 1.28 | 1.55 |  | 0.94 | 1.15 | 0.93 | 1.4 | 9.20E-03 |
| gtf2h4 | NM_010364 | 0.88 | 1.42 | 2.09** | 2.53** |  | 0.55* | 2.71** | 0.89 | 1.93** | 1.77E-02 |
| gtf2h4 | NM_010364 | 1.18 | 0.82 | 2.48** | 1.74* |  | 0.55* | 3.66** | 0.83 | 3.24** | 4.33E-02 |
| gtpbp4 | AI987834 | 0.82 | 1.24 | 2.00** | 1.69** |  | 0.86 | 2.19** | 1.21 | 1.27 | 3.66E-02 |
| gusb | AK002832 | 0.88 | 1.49* | 2.09** | 2.82** |  | 0.91 | 2.19** | 0.74 | 3.92** | 1.72E-02 |
| h2afj | BF661121 | 1.4 | 0.93 | 1.7 | 1.21 |  | 1.81 | 1.47 | 1.56 | 1.54 | 4.92E-02 |
| h2afv | BG092470 | 0.52** | 0.76 | 2.17** | 0.67* |  | 0.71* | 0.94 | 0.91 | 1.05 | 2.11E-02 |
| h2afx | NM_010436 | 1.17 | 2.17** | 4.56** | 1.40** |  | 0.67** | 1.22* | 0.97 | 1.01 | 3.43E-03 |
| haghl | AK021220 | 1.31 | 0.49** | 2.71** | 5.28** |  | 0.95 | 2.51** | 0.9 | 2.47** | 4.54E-02 |
| hdac6 | NM_010413 | 0.68* | 0.45** | 1.71** | 0.79 |  | 0.67* | 1.13 | 0.63** | 1.68** | 2.24E-02 |
| hells | NM_008234 | 1.93* | 11.54** | 17.37** | 10.68** |  | 0.38** | 0.57 | 1.24 | 1.33 | 1.59E-02 |
| hells | AK021390 | 1.19 | 5.87** | 2.77** | 3.39** |  | 0.65 | 0.9 | 1.29 | 0.83 | 4.92E-02 |
| hes6 | AI326893 | 2.81** | 1.91** | 1 | 3.33** |  | 0.91 | 1.80* | 0.83 | 1.46 | 3.40E-02 |
| hexb | NM_010422 | 0.47** | 0.67 | 1.60* | 1.32 |  | 0.48** | 1.15 | 0.39** | 0.98 | 1.88E-03 |
| hexim1 | BI411874 | 0.69** | 0.48** | 1.47** | 1.08 |  | 0.84 | 0.53** | 0.89 | 1.12 | 1.74E-02 |
| heyl | BG695100 | 0.56** | 0.35** | 1.09 | 1.76** |  | 1.07 | 1.18 | 1.07 | 1.2 | 1.27E-02 |
| hist1h1c | NM_015786 | 0.57** | 0.32** | 0.77** | 0.69** |  | 1.13 | 0.88 | 0.91 | 0.79* | 3.00E-04 |
| hist1h1c | BB533903 | 0.72* | 0.40** | 0.63** | 0.85 |  | 0.94 | 0.77* | 1.11 | 0.69** | 3.69E-02 |
| hist2h2aa1 | BC010564 | 1.04 | 0.32** | 1.27 | 1.24 |  | 1.2 | 1.26 | 0.76 | 0.65* | 1.97E-02 |
| hlf | BB744589 | 5.21** | 3.08** | 1.1 | 3.50** |  | 1.77* | 0.51* | 1.28 | 0.86 | 3.29E-03 |
| hmga1 | NM_016660 | 2.85** | 3.23** | 3.00** | 3.78** |  | 0.83 | 2.42** | 1.02 | 1.3 | 3.35E-02 |
| hmga2-ps1 | AV377334 | 1.17 | 2.02** | 0.72 | 2.29** |  | 1.67** | 3.82** | 0.73* | 1.25 | 4.02E-03 |
| hmgb2 | C85885 | 0.98 | 2.24** | 5.26** | 1.31 |  | 0.69 | 0.82 | 1.25 | 0.92 | 3.27E-03 |
| hnf4a | BF580781 | 2.03** | 0.96 | 0.81 | 1.36* |  | 1.03 | 1.11 | 1.09 | 0.84 | 1.92E-03 |
| hook3 | BB476531 | 0.79 | 0.46** | 0.48** | 0.62** |  | 1.27 | 0.74* | 1.22 | 0.59** | 3.24E-02 |
| hook3 | BM250446 | 0.85 | 0.39** | 0.51** | 0.73* |  | 0.9 | 0.62** | 0.92 | 0.71** | 9.99E-03 |
| hoxa10 | AK002670 | 1.09 | 0.97 | 0.93 | 0.84 |  | 1.16 | 2.78** | 0.98 | 1.61** | 2.43E-02 |
| hoxc10 | BB779859 | 0.93 | 1.12 | 1 | 1.07 |  | 0.65* | 3.51** | 0.61* | 1.71** | 1.93E-02 |
| hps1 | BB188040 | 1.02 | 0.57 | 0.59* | 0.41** |  | 0.25** | 0.79 | 0.38** | 1.47 | 3.93E-02 |
| hps3 | AF393780 | 2.06** | 1.16 | 1.25 | 1.69** |  | 1.06 | 0.89 | 1.02 | 1.64* | 4.82E-02 |
| hras1 | NM_008284 | 1.16 | 1.16 | 3.06** | 2.60** |  | 1.14 | 2.89** | 0.61* | 1.35 | 7.09E-03 |
| hras1 | BC011083 | 1.43 | 1.43 | 3.24** | 2.40** |  | 1.03 | 2.15** | 0.7 | 1.34 | 1.14E-02 |
| hrb | BB130716 | 2.36** | 0.99 | 0.87 | 2.43** |  | 1.06 | 1.09 | 1.81** | 1.81** | 1.43E-04 |
| hrc | BC021623 | 1.01 | 0.9 | 1.14 | 1.03 |  | 1.13 | 0.40** | 1.06 | 0.65** | 2.30E-02 |
| hsbp1 | AK010939 | 0.92 | 0.71 | 2.64** | 1.68* |  | 0.79 | 2.02** | 0.74 | 1.81** | 2.91E-02 |
| hspa9 | BB718260 | 1.41** | 1.98** | 1.31* | 1.2 |  | 1.03 | 1.06 | 1.25 | 0.86 | 3.18E-03 |
| htatip2 | AF061972 | 0.84 | 0.40** | 0.74** | 0.64** |  | 0.82 | 0.92 | 0.92 | 1.38** | 1.66E-02 |
| hus1 | NM_008316 | 0.85 | 1.97** | 2.04** | 1.62** |  | 0.97 | 0.96 | 1.24 | 1.23 | 1.46E-02 |
| ick | BB376918 | 2.62** | 1.22 | 1.28* | 1.90** |  | 1.22 | 0.61** | 1.11 | 0.69** | 8.05E-03 |
| ifrd2 | BB540964 | 2.00** | 1.04 | 2.30** | 1.66 |  | 1.07 | 2.18** | 1.3 | 2.51** | 9.97E-03 |
| igf1 | NM_010512 | 2.08* | 0.63 | 0.74 | 1.2 |  | 1.17 | 2.33** | 1.47 | 2.00* | 4.75E-02 |
| igf1 | AF440694 | 1.05 | 1 | 1.59 | 3.71** |  | 1.56* | 2.24** | 1.24 | 3.08** | 4.66E-02 |
| igfbp4 | BB787243 | 0.28** | 0.56** | 1.35 | 1.78** |  | 1.07 | 2.05** | 0.72 | 1.73** | 1.18E-02 |
| igfbp6 | NM_008344 | 0.92 | 1.12 | 0.85 | 0.92 |  | 1.57 | 10.04** | 0.28** | 6.05** | 1.57E-03 |
| ihpk1 | AK004808 | 0.60* | 0.35** | 1.48 | 0.8 |  | 1.44 | 1.28 | 0.79 | 1.92** | 1.35E-02 |
| imp4 | AF334609 | 2.38** | 2.41** | 2.55** | 3.26** |  | 0.85 | 2.09** | 0.91 | 1.96** | 2.89E-02 |
| ing3 | BB298005 | 0.9 | 1.12 | 1.15 | 0.66** |  | 0.79 | 0.49** | 0.77* | 1.34* | 1.50E-02 |
| inhba | NM_008380 | 0.58** | 0.38** | 0.96 | 0.60** |  | 0.94 | 1.12 | 0.85 | 1.31* | 1.31E-02 |
| irf2bp1 | BC019164 | 1.11 | 0.77 | 1.30* | 0.96 |  | 0.70** | 1.99** | 1.13 | 0.9 | 3.70E-02 |
| irf2bp2 | BB183385 | 0.88 | 0.39** | 0.64* | 0.9 |  | 1.60** | 0.60** | 1.11 | 0.69* | 2.15E-02 |
| irf2bp2 | BB183385 | 0.51** | 0.44** | 1.4 | 1.05 |  | 0.93 | 0.8 | 1.12 | 0.95 | 4.08E-02 |
| irx4 | NM_018885 | 0.99 | 0.96 | 1.11 | 0.89 |  | 0.8 | 0.47** | 0.78* | 1.12 | 7.78E-03 |
| isoc1 | AK010892 | 0.95 | 2.27** | 1.75* | 2.21** |  | 0.9 | 1.33 | 1.33 | 1.42 | 2.79E-02 |
| itch | NM_008395 | 0.89 | 0.55** | 0.83* | 1.16* |  | 0.93 | 0.50** | 1.03 | 0.61** | 2.33E-02 |
| itga6 | BM935811 | 0.61* | 0.33** | 0.8 | 0.68 |  | 0.93 | 0.64* | 0.97 | 0.69 | 2.49E-02 |
| itga7 | NM_008398 | 0.85 | 0.86 | 1.40* | 2.86** |  | 0.50* | 1.67* | 0.76 | 2.48** | 2.14E-02 |
| itgb2 | NM_008404 | 1.27 | 1 | 2.18** | 3.99** |  | 1.27 | 2.81** | 0.65 | 4.16** | 8.45E-03 |
| itgb6 | NM_021359 | 0.96 | 0.97 | 0.95 | 1.04 |  | 1.11 | 2.22** | 1.33** | 1.55** | 3.45E-02 |
| itgb8 | BB504737 | 1.06 | 0.57* | 0.6 | 2.70** |  | 0.76 | 0.31** | 1.47 | 0.52* | 1.51E-03 |
| itih5 | AV239969 | 0.50** | 0.76 | 0.77* | 1.42 |  | 1.07 | 0.99 | 0.76 | 0.66** | 1.02E-02 |
| jag1 | AV359819 | 0.81* | 1.21 | 0.88 | 1.40** |  | 0.69** | 0.48** | 0.91 | 0.48** | 9.46E-03 |
| jag2 | AV264681 | 2.77** | 2.36* | 2.36** | 4.13** |  | 1.24 | 0.74 | 0.99 | 1.85* | 1.33E-02 |
| jag2 | AV264681 | 2.43** | 1.32 | 1.16 | 2.02** |  | 0.69 | 1.33 | 1.03 | 0.9 | 3.18E-02 |
| jakmip1 | AV290082 | 0.50** | 0.55** | 0.85 | 0.99 |  | 0.97 | 1.04 | 0.99 | 0.89 | 6.94E-03 |
| jakmip2 | AI850334 | 0.47** | 0.65* | 0.17** | 0.9 |  | 0.95 | 1.1 | 0.97 | 0.93 | 1.70E-02 |
| jmjd6 | AK017622 | 0.8 | 1.13 | 1.07 | 2.95** |  | 1.1 | 2.32** | 1.04 | 2.11** | 6.93E-03 |
| josd3 | BE996326 | 1.72** | 2.82** | 0.73 | 1.29 |  | 0.93 | 0.95 | 1.02 | 0.98 | 1.97E-02 |
| jub | NM_010590 | 0.68* | 0.24** | 2.34** | 1.11 |  | 1.1 | 0.87 | 0.96 | 0.99 | 1.96E-02 |
| jup | BI525123 | 0.67* | 0.55** | 0.71 | 0.58** |  | 0.51** | 0.64** | 0.93 | 1.12 | 4.95E-02 |
| kazald1 | AI842353 | 0.85 | 0.50** | 1.29* | 0.57** |  | 0.86 | 0.79* | 0.66** | 0.75** | 3.23E-02 |
| kctd4 | AI843843 | 1.04 | 1.05 | 0.97 | 1.01 |  | 0.51** | 0.31** | 0.80* | 0.43** | 6.70E-04 |
| kif13a | BB040841 | 0.85 | 0.59 | 0.43* | 0.66 |  | 1.31 | 0.34** | 1.13 | 0.50* | 2.40E-02 |
| kif13b | BB767898 | 0.48** | 0.49** | 1.18 | 0.88 |  | 1.04 | 1 | 0.92 | 1.12 | 8.40E-04 |
| kif13b | AK020905 | 0.98 | 0.49** | 1.11 | 0.93 |  | 1.12 | 0.60** | 0.76* | 0.91 | 6.18E-03 |
| kif1b | BQ175246 | 0.82 | 0.38** | 1.08 | 0.9 |  | 0.83 | 0.67 | 0.77 | 0.88 | 3.95E-02 |
| kif1b | AB023656 | 0.7 | 0.76 | 1.27 | 1.13 |  | 0.62* | 0.43** | 0.78 | 0.57* | 3.23E-02 |
| kif21a | NM_016705 | 0.86 | 0.63* | 1.43* | 1.13 |  | 0.54** | 0.40** | 0.55** | 0.91 | 8.21E-03 |
| kif22 | BB251322 | 0.36** | 0.54** | 2.47** | 0.81 |  | 0.8 | 0.69* | 1.2 | 0.72* | 3.61E-02 |
| kif2a | NM_008442 | 2.22** | 1.16 | 1.69** | 2.00** |  | 0.89 | 0.43** | 1.43* | 0.98 | 4.59E-02 |
| kif5a | AU067810 | 1.12 | 0.48** | 0.91 | 1.19* |  | 1.09 | 0.83** | 1.01 | 0.82* | 2.81E-02 |
| kif5b | BI328541 | 0.23* | 1 | 0.67 | 0.86 |  | 0.81 | 0.36 | 1.28 | 0.34* | 3.44E-02 |
| kif5b | BI328541 | 0.69* | 0.63** | 0.84 | 0.51** |  | 1.33* | 0.50** | 1.60** | 0.48** | 1.70E-02 |
| kitl | BB815530 | 0.8 | 0.97 | 1.05 | 1 |  | 1.08 | 0.43** | 1.19 | 0.69** | 1.08E-02 |
| kitl | BB815530 | 0.67* | 0.36** | 0.34** | 0.43** |  | 1 | 0.29** | 1.1 | 1.02 | 9.96E-03 |
| klc1 | AK014256 | 0.79* | 0.54** | 1.39** | 1.49** |  | 0.49** | 1.31* | 0.69** | 2.00** | 3.54E-02 |
| klc4 | NM_029091 | 0.45** | 0.31** | 1.11 | 0.53** |  | 0.73** | 0.9 | 0.77* | 0.95 | 3.59E-02 |
| klf16 | NM_078477 | 2.28** | 1.32 | 1.01 | 1.54** |  | 0.89 | 1.21 | 0.85 | 1.39* | 3.48E-04 |
| klk6 | NM_011177 | 1.09 | 0.96 | 1.07 | 0.71 |  | 0.40** | 0.50* | 0.59* | 0.41** | 3.96E-02 |
| kpna1 | U20619 | 2.66** | 0.94 | 0.97 | 1.85** |  | 0.77 | 0.66* | 1.05 | 0.75 | 3.09E-02 |
| krt16 | NM_008470 | 0.92 | 0.97 | 1.29 | 0.78 |  | 0.40** | 0.81 | 0.51** | 0.19** | 6.57E-03 |
| krt18 | NM_010664 | 0.38** | 0.26** | 0.88 | 0.24** |  | 0.72 | 1.17 | 1.05 | 1.07 | 2.40E-02 |
| krt24 | AK009986 | 0.97 | 1.32 | 1.24* | 0.96 |  | 0.52** | 1.74** | 2.08** | 1.04 | 1.75E-02 |
| krt27 | NM_010666 | 0.9 | 1 | 0.91 | 0.86 |  | 0.92 | 0.31** | 0.96 | 0.87 | 3.44E-02 |
| krt6b | NM_010669 | 1.62 | 1.02 | 1.5 | 0.92 |  | 0.06** | 1.03 | 0.27** | 0.00** | 8.00E-03 |
| ktn1 | BF162017 | 3.22** | 1.36* | 1.90** | 2.85** |  | 1.18 | 0.65** | 1.32* | 0.70** | 2.75E-02 |
| l7rn6 | BC003916 | 2.52** | 1.74** | 1.89** | 4.25** |  | 1.05 | 1.36* | 1.24 | 1.22 | 4.45E-02 |
| lasp1 | BB377636 | 0.35** | 0.37** | 0.88 | 0.72* |  | 1.13 | 1.16 | 1.35** | 0.97 | 2.91E-02 |
| lasp1 | BB105164 | 0.38** | 0.39** | 0.83 | 0.60** |  | 1.02 | 1.18 | 1.48* | 1 | 3.97E-02 |
| lasp1 | BB433798 | 0.42** | 0.39** | 1.07 | 0.89 |  | 1.08 | 1.04 | 1.19 | 1.03 | 4.69E-02 |
| lasp1 | AV027151 | 0.34** | 0.37** | 1 | 0.75* |  | 0.81 | 1.43* | 0.93 | 1.39* | 9.92E-03 |
| lasp1 | BG146595 | 0.49** | 0.62** | 0.88 | 0.71** |  | 0.58** | 1.40** | 0.84 | 1.2 | 6.43E-03 |
| ldb2 | NM_010698 | 0.45** | 0.72* | 0.59* | 1.27 |  | 0.84 | 0.51** | 1.28 | 1.26 | 8.50E-03 |
| limch1 | BM117827 | 0.88 | 0.29** | 0.43** | 0.48** |  | 1.25 | 0.53* | 0.95 | 0.67 | 4.13E-02 |
| limk1 | NM_010717 | 2.52** | 2.01** | 0.92 | 2.57** |  | 1.15 | 1.1 | 1.14 | 2.18** | 1.85E-03 |
| limk2 | BE981766 | 0.36** | 0.20** | 1.61* | 1.35 |  | 0.42** | 1.3 | 0.73 | 1.53* | 2.11E-03 |
| lmo1 | NM_057173 | 0.37** | 1.17 | 2.15** | 0.60* |  | 0.69 | 1.57* | 0.7 | 1.33 | 1.91E-02 |
| lnx1 | BB131619 | 0.74 | 0.78 | 1 | 0.92 |  | 0.67* | 0.47** | 0.94 | 0.44** | 4.33E-02 |
| loc100040631 | BG094946 | 0.30** | 0.26** | 1.85 | 2.01* |  | 1.75 | 0.52 | 0.38** | 1.41 | 2.19E-03 |
| loc665622 | BC011440 | 2.08** | 1.02 | 0.87 | 2.76** |  | 1.04 | 0.85 | 1.02 | 0.97 | 1.15E-02 |
| lpp | BB557975 | 0.95 | 0.56** | 0.76 | 0.8 |  | 1.25 | 0.39** | 1.29 | 0.49** | 2.32E-02 |
| lpp | BB089138 | 0.99 | 0.28** | 1.05 | 0.9 |  | 1 | 0.56* | 0.86 | 0.59* | 1.26E-02 |
| lpp | BM236111 | 1.11 | 0.47** | 0.68* | 0.38** |  | 1.22 | 0.48** | 0.8 | 0.62** | 1.70E-02 |
| lrrc49 | BC016574 | 0.87 | 0.68* | 1.55** | 0.93 |  | 1.2 | 2.39** | 2.26** | 4.38** | 4.66E-02 |
| lrsam1 | AW557823 | 0.71** | 0.69** | 1.02 | 0.53** |  | 0.39** | 0.60** | 0.74** | 1.64** | 3.35E-02 |
| lsp1 | NM_019391 | 0.9 | 0.78* | 0.97 | 1.1 |  | 1.02 | 3.42** | 0.67** | 1.90** | 3.22E-02 |
| lypla1 | AK016021 | 1.15 | 0.44** | 1.45* | 4.23** |  | 0.62* | 1.08 | 0.98 | 1.87** | 3.40E-02 |
| lyst | BB463428 | 0.38** | 1.48 | 2.90** | 1.15 |  | 0.65* | 0.8 | 0.86 | 1.13 | 3.63E-02 |
| maged2 | AF319976 | 0.29** | 0.33** | 0.93 | 0.77 |  | 0.55** | 2.32** | 0.63* | 2.07** | 2.12E-02 |
| map1lc3a | BC010596 | 0.65 | 0.46** | 1.70* | 1.12 |  | 0.7 | 1.4 | 0.76 | 2.33** | 4.73E-02 |
| map1lc3b | AU080586 | 0.62** | 0.45** | 0.97 | 0.95 |  | 1.07 | 0.97 | 0.87 | 0.81* | 9.33E-04 |
| mapk10 | L35236 | 0.48** | 0.22** | 1.50** | 1.73** |  | 0.91 | 1 | 1.06 | 0.92 | 2.96E-02 |
| mapk1ip1 | AK009250 | 0.62** | 0.46** | 0.77** | 0.81* |  | 0.99 | 0.88 | 0.73** | 0.96 | 1.11E-03 |
| mapk8ip1 | BB546463 | 0.62** | 0.35** | 0.74 | 0.53** |  | 0.89 | 1.14 | 0.83 | 1.06 | 1.56E-02 |
| mapk8ip2 | AW536912 | 0.47** | 0.93 | 1.60* | 1.49* |  | 0.88 | 0.96 | 1.09 | 0.94 | 2.29E-02 |
| mark1 | AW491150 | 0.72** | 0.42** | 0.82 | 1.18 |  | 0.87 | 0.66** | 1.18 | 0.86 | 7.86E-04 |
| mark1 | AW491150 | 0.78* | 1.28 | 0.39** | 1.01 |  | 1.06 | 0.49** | 1.30* | 0.70** | 3.77E-02 |
| mast2 | BB233292 | 1.18* | 1.06 | 1.12 | 0.84* |  | 1.24* | 0.50** | 0.98 | 1.09 | 3.30E-02 |
| mat2a | BB470596 | 2.08** | 1.63** | 0.97 | 1.88** |  | 1.02 | 1.26* | 1.03 | 1.14 | 2.78E-04 |
| mat2a | AV260654 | 2.16** | 2.11** | 1.05 | 1.66** |  | 0.79 | 1.40** | 0.94 | 1.19 | 3.45E-02 |
| mbnl3 | NM_134163 | 1 | 0.86 | 1.59** | 0.95 |  | 0.39** | 0.38** | 1.2 | 0.56** | 7.93E-03 |
| mbnl3 | BB211386 | 1.05 | 0.94 | 1.12 | 0.94 |  | 0.67* | 0.37** | 0.74* | 0.64* | 1.81E-02 |
| mcc | BB794635 | 0.85 | 0.83 | 1.12 | 1.81** |  | 0.96 | 0.36** | 0.99 | 0.52** | 3.81E-02 |
| med13 | BB667559 | 1 | 0.44** | 0.66* | 0.9 |  | 1.35 | 0.68* | 1.04 | 0.73* | 1.08E-02 |
| med24 | BC005409 | 1.16 | 0.77 | 1.25* | 1.02 |  | 0.78 | 2.07** | 0.8 | 2.65** | 1.57E-02 |
| med30 | NM_027212 | 1.24 | 2.10** | 2.64** | 2.45** |  | 0.61** | 1.53* | 0.7 | 1.3 | 2.53E-02 |
| meis1 | BG070088 | 0.81* | 0.49** | 1.18 | 1.51** |  | 1.09 | 0.93 | 0.98 | 0.68** | 1.44E-02 |
| mesdc2 | NM_023403 | 1.05 | 0.83* | 1.30** | 1.33** |  | 1.15* | 2.00** | 0.9 | 1.78** | 1.52E-02 |
| mfng | NM_008595 | 1.21 | 1.28 | 2.10** | 2.62** |  | 0.79 | 2.76** | 0.74 | 2.89** | 2.45E-02 |
| mgll | AK006949 | 1.01 | 1.19 | 1.11 | 0.95 |  | 1.01 | 3.19** | 0.74 | 1.54* | 6.24E-03 |
| mia3 | AI847406 | 1.24* | 0.42** | 0.36** | 0.83* |  | 1 | 0.78** | 0.98 | 0.9 | 2.62E-03 |
| mia3 | AI552688 | 0.9 | 0.31** | 0.68* | 0.41** |  | 0.93 | 0.57** | 0.91 | 0.50** | 1.48E-02 |
| mical1 | NM_138315 | 1.17 | 1.47** | 1.01 | 0.98 |  | 0.50** | 1.22* | 0.55** | 2.43** | 1.02E-02 |
| mical1 | BB209438 | 1.2 | 2.33** | 1.58* | 0.84 |  | 0.68** | 0.99 | 0.76 | 1.40* | 2.75E-02 |
| minpp1 | AK017558 | 1.07 | 1.26 | 1.03 | 0.95 |  | 0.55** | 0.52** | 0.96 | 0.66* | 3.35E-02 |
| mitf | BB763517 | 0.49* | 0.34** | 0.82 | 0.91 |  | 0.88 | 0.79 | 0.95 | 0.59 | 1.72E-02 |
| mlf1ip | BB667813 | 1.09 | 2.11** | 6.30** | 1.34 |  | 1.06 | 0.94 | 1.32 | 1.01 | 2.47E-02 |
| mll3 | AV297525 | 1.63** | 0.87 | 0.67* | 0.93 |  | 0.73* | 0.45** | 0.75* | 1.13 | 3.99E-02 |
| mll5 | BB295149 | 0.77 | 0.35** | 0.66 | 0.72 |  | 1.28 | 0.48* | 1.07 | 0.65 | 4.20E-02 |
| mll5 | BF021054 | 0.99 | 0.59** | 0.97 | 0.82 |  | 0.96 | 0.46** | 1.1 | 0.75* | 7.36E-03 |
| mll5 | BF021054 | 1.01 | 0.57** | 0.44** | 0.45** |  | 1.57** | 0.27** | 1.26 | 0.50** | 3.49E-02 |
| mllt11 | NM_019914 | 2.38** | 2.26** | 1.55** | 3.06** |  | 0.98 | 0.63* | 1.16 | 1.13 | 2.63E-03 |
| mllt3 | AK011386 | 1.80* | 2.68** | 0.91 | 1.48 |  | 0.98 | 0.54* | 1.1 | 1.4 | 3.56E-02 |
| morf4l2 | NM_019768 | 0.67* | 1.02 | 1.77** | 0.67** |  | 0.78* | 2.72** | 0.99 | 2.34** | 2.21E-02 |
| mrpl22 | BF018217 | 2.61** | 2.30** | 1.80** | 2.59** |  | 1.25 | 1.36 | 1.33 | 1.62* | 1.29E-04 |
| mrpl23 | NM_011288 | 1.13 | 0.82 | 2.43** | 1.38* |  | 1.2 | 2.23** | 0.8 | 0.99 | 1.36E-02 |
| mrto4 | BC005734 | 1.33 | 1.41* | 2.63** | 1.97** |  | 0.74* | 2.74** | 1.32 | 1.65** | 2.93E-02 |
| mtap | BG075139 | 2.68** | 2.72** | 1.57** | 2.51** |  | 1.27* | 1.37* | 1.48** | 0.94 | 4.70E-02 |
| mtap | BG075139 | 4.12** | 5.19** | 2.39** | 6.41** |  | 1.36* | 1.21 | 1.24 | 1.54** | 6.43E-03 |
| mtap | BG075139 | 1.99** | 1.33 | 1 | 1.52* |  | 1.06 | 1.64** | 1.12 | 2.28** | 4.82E-03 |
| mtap | BG075139 | 2.71** | 2.43** | 1.44** | 3.46** |  | 0.91 | 1.69** | 1.09 | 1.97** | 1.35E-02 |
| mtap9 | BB698742 | 1.02 | 1.56* | 0.57** | 1.31 |  | 1.12 | 2.00** | 1.21 | 1.25 | 5.38E-03 |
| mtf1 | BE980297 | 0.96 | 0.27** | 1.15 | 1.04 |  | 1.04 | 0.82* | 1.02 | 0.97 | 6.13E-03 |
| mtvr2 | NM_023166 | 1.41* | 1.17 | 2.30** | 2.83** |  | 0.59** | 2.16** | 0.58** | 2.16** | 2.01E-02 |
| mxi1 | BB825697 | 2.24** | 1.44** | 3.24** | 3.09** |  | 1 | 0.91 | 0.93 | 1.02 | 2.17E-02 |
| mybbp1a | AW228043 | 1.5 | 0.50** | 1.09 | 2.34** |  | 1.16 | 1.71* | 1.05 | 1.38 | 5.57E-03 |
| myc | BC006728 | 1.1 | 0.99 | 1.12 | 1.02 |  | 0.32** | 0.65* | 0.53** | 0.99 | 1.97E-02 |
| myd116 | NM_008654 | 0.89 | 0.47** | 1.70** | 0.96 |  | 0.53** | 1.42 | 0.48** | 1.48* | 3.56E-02 |
| myh14 | AK010278 | 0.71* | 0.49** | 1.06 | 0.68** |  | 1.07 | 0.62** | 1.11 | 1.03 | 4.44E-03 |
| myh4 | BG794681 | 0.99 | 1.25 | 0.65 | 1.06 |  | 1.72 | 2.94* | 0.94 | 1.55 | 8.16E-03 |
| myo15b | BF581819 | 0.40** | 0.25** | 0.66** | 0.44** |  | 0.81 | 0.9 | 1.03 | 0.89 | 1.37E-03 |
| myo15b | BB072116 | 0.73** | 0.48** | 1.04 | 1.16 |  | 1.52** | 0.89 | 1.22* | 0.79* | 3.66E-02 |
| myo1c | NM_008659 | 0.98 | 1.01 | 1.08 | 0.99 |  | 1.29* | 2.22** | 1.56** | 2.87** | 2.24E-03 |
| myo1f | AK021181 | 1.04 | 1.84* | 1.2 | 5.83** |  | 0.52 | 3.64* | 0.8 | 2.08* | 1.12E-02 |
| myo5b | AW546331 | 1.19 | 1.03 | 0.62 | 0.57 |  | 0.92 | 0.32** | 0.64 | 0.45** | 5.57E-03 |
| myo6 | BE133806 | 0.61* | 0.30** | 1.88** | 0.60* |  | 0.53** | 0.68* | 0.73 | 0.79 | 3.53E-02 |
| myo6 | BB200233 | 0.49* | 0.69 | 2.61** | 0.60* |  | 0.81 | 0.48* | 1.01 | 0.78 | 3.06E-02 |
| nanp | NM_026086 | 2.14** | 0.84 | 1.48** | 1.56** |  | 0.82 | 1.12 | 1.01 | 0.93 | 2.52E-02 |
| narg1 | BG067031 | 1.05 | 0.97 | 1.40** | 1.74** |  | 0.92 | 2.03** | 1.68** | 1.05 | 2.23E-02 |
| ncoa2 | BM234716 | 3.01** | 0.98 | 1.38* | 2.23** |  | 0.65* | 0.42** | 0.86 | 0.86 | 3.76E-02 |
| ndel1 | BC021434 | 1.14 | 0.71 | 1 | 1.16 |  | 2.12* | 0.24** | 0.33** | 0.73 | 1.67E-02 |
| ndrg4 | AV006122 | 0.42** | 0.42** | 0.70* | 0.79 |  | 0.92 | 1.19 | 0.98 | 0.98 | 2.90E-02 |
| ndrg4 | AI837704 | 0.30** | 0.36** | 1.15 | 0.95 |  | 0.72 | 1.51 | 0.8 | 1.52 | 4.65E-02 |
| neu2 | AK009828 | 5.26** | 2.82** | 2.99** | 5.55** |  | 0.81 | 1.11 | 1.03 | 1.47 | 1.77E-03 |
| nfatc1 | NM_016791 | 0.88 | 0.40** | 1.03 | 1.19 |  | 1 | 0.78* | 0.91 | 0.65** | 3.94E-03 |
| nhp2l1 | NM_011482 | 2.13** | 1.86** | 1.91** | 2.07** |  | 0.96 | 1.12 | 1.34* | 1.22* | 2.20E-02 |
| nkx6-1 | AF357883 | 0.53* | 0.32** | 2.29** | 1.13 |  | 0.91 | 0.94 | 1.09 | 0.98 | 4.29E-02 |
| nme1 | BC005629 | 0.74 | 0.75 | 3.17** | 2.38** |  | 0.79 | 2.46** | 1.43 | 1.61** | 3.69E-02 |
| noc2l | BC020013 | 2.72** | 1.88** | 1.1 | 1.55 |  | 1.34 | 1.81** | 1.37 | 1.39 | 1.66E-02 |
| nol5 | BB729616 | 1.02 | 1.83* | 2.31** | 2.28** |  | 1.3 | 5.12** | 1.33 | 1.64* | 4.43E-03 |
| nol5a | BF660256 | 1.51** | 3.70** | 2.39** | 2.46** |  | 1.75** | 3.29** | 2.20** | 1.65** | 4.71E-02 |
| nol5a | BM249243 | 2.85** | 2.47** | 2.95** | 3.34** |  | 1.32 | 1.40* | 1.60* | 1.67** | 4.98E-02 |
| nola2 | BC024944 | 1.95** | 2.57** | 5.41** | 4.00** |  | 1.12 | 3.30** | 1.04 | 1.5 | 1.66E-02 |
| nola2 | BC024944 | 2.25** | 3.68** | 2.98** | 1.98** |  | 0.95 | 2.52** | 1.31 | 1.37* | 4.75E-02 |
| nola3 | AK004120 | 1.13 | 1.47** | 2.11** | 2.13** |  | 1.05 | 2.04** | 1.21* | 1.27* | 1.95E-02 |
| nolc1 | BM213850 | 1.79* | 2.35* | 0.95 | 2.99** |  | 1.59* | 1.81* | 1.87* | 1.03 | 1.10E-02 |
| nolc1 | BM213850 | 4.16** | 2.07* | 2.37** | 3.98** |  | 1.06 | 1.25 | 1.35 | 1.77* | 1.87E-04 |
| nolc1 | NM_053086 | 1.96* | 0.94 | 1.28 | 2.67** |  | 1.23 | 1.31 | 1.79 | 0.83 | 7.33E-03 |
| notch3 | NM_008716 | 0.83 | 1.1 | 0.55* | 0.88 |  | 1.04 | 0.48** | 1.17 | 0.69 | 4.34E-02 |
| nov | X96585 | 0.8 | 0.82 | 0.46** | 0.85 |  | 0.99 | 0.45** | 0.49** | 0.51** | 9.88E-04 |
| noxo1 | BC019525 | 0.50** | 0.60** | 1.19 | 0.31** |  | 0.95 | 0.97 | 1.06 | 1.17 | 3.28E-03 |
| nr1h3 | NM_013839 | 1.2 | 0.91 | 1.19 | 1.09 |  | 0.87 | 2.49** | 0.86 | 1.09 | 2.03E-02 |
| nr2c1 | NM_011629 | 0.51** | 0.71* | 1.76** | 1.04 |  | 0.94 | 1.46* | 0.78 | 1.78** | 4.64E-02 |
| nr2c1 | AV118678 | 0.68* | 0.94 | 0.73* | 0.72* |  | 0.84 | 0.49** | 1.14 | 0.87 | 1.77E-02 |
| nt5c3 | AV037573 | 0.60** | 0.47** | 1.62** | 0.76* |  | 0.73* | 1.09 | 0.83 | 0.84 | 1.08E-02 |
| nuak2 | AK004737 | 0.50** | 1.02 | 1.88** | 0.64** |  | 0.71* | 1.37* | 0.82 | 1.48** | 2.45E-02 |
| nufip1 | NM_013745 | 2.10** | 1.08 | 2.80** | 2.25** |  | 1.26* | 1.58** | 1.13 | 1.13 | 1.12E-02 |
| numb | U70674 | 0.69** | 0.37** | 1 | 0.49** |  | 1.13 | 1.05 | 0.82* | 1.07 | 4.20E-04 |
| nup85 | BB320388 | 2.69** | 5.80** | 2.41** | 2.54** |  | 0.97 | 1.34* | 1.51* | 1.50* | 4.05E-02 |
| nupr1 | NM_019738 | 0.58** | 0.30** | 1.26 | 0.51** |  | 1.22 | 1.28 | 0.46** | 1.14 | 6.78E-03 |
| nupr1 | NM_019738 | 0.62* | 0.46** | 1.23 | 0.66* |  | 1.24 | 1.3 | 0.49** | 1.04 | 3.42E-02 |
| obfc2a | AV313559 | 0.43** | 0.22** | 0.74 | 0.75 |  | 1.03 | 1.53** | 0.97 | 1.42* | 8.73E-03 |
| odc1 | S64539 | 1.72** | 2.29** | 1.83** | 1.82** |  | 1.11 | 1.62** | 0.99 | 1.81** | 2.43E-02 |
| olfml2a | BM202709 | 1.14 | 0.98 | 0.86 | 1.36 |  | 0.89 | 0.38** | 0.66** | 1.15 | 2.69E-02 |
| ovol1 | BC021411 | 1.01 | 1.28 | 0.81 | 0.89 |  | 0.78 | 0.41** | 0.54** | 0.68* | 5.67E-03 |
| p42pop | AF364868 | 1.37* | 2.72** | 1.39* | 1.78** |  | 1.09 | 0.9 | 1.3 | 1.36 | 1.94E-03 |
| pacsin2 | NM_011862 | 1.46** | 2.07** | 1.56** | 1.41** |  | 0.64** | 0.83 | 0.87 | 1.11 | 1.70E-02 |
| pak3 | BB468082 | 0.39** | 0.61* | 0.82 | 0.48** |  | 0.92 | 0.7 | 1.04 | 1.08 | 9.33E-03 |
| palld | BB534971 | 1.03 | 1 | 1.05 | 0.99 |  | 0.98 | 2.06** | 0.59** | 1.81** | 3.31E-02 |
| palm | BC015297 | 0.77 | 0.83 | 0.79 | 0.85 |  | 0.46** | 3.02** | 0.42** | 2.42** | 1.71E-02 |
| papss2 | BF786072 | 0.21** | 0.18** | 1.26 | 0.23** |  | 1 | 0.81 | 0.77 | 0.95 | 4.63E-02 |
| papss2 | BF786072 | 0.31** | 0.16** | 0.65** | 0.29** |  | 1.02 | 0.62** | 0.92 | 0.91 | 4.71E-02 |
| papss2 | BF780807 | 0.59** | 0.11** | 0.97 | 0.39** |  | 0.72* | 0.72* | 0.77 | 0.84 | 2.84E-02 |
| pard6a | NM_019695 | 0.69** | 0.32** | 1.62** | 0.49** |  | 0.85* | 1.21 | 0.88 | 1.18 | 1.75E-02 |
| parp1 | BB767586 | 2.36** | 2.22** | 0.97 | 1.95** |  | 0.99 | 0.89 | 1.50** | 0.84* | 8.08E-05 |
| parp11 | BB026163 | 0.58** | 0.47** | 0.8 | 0.96 |  | 0.73* | 0.63** | 1 | 1.27 | 1.16E-02 |
| parp14 | BC021340 | 0.52** | 0.42** | 0.46** | 0.64* |  | 1.14 | 1.07 | 0.55** | 1.17 | 9.24E-03 |
| parp3 | AW990611 | 1.02 | 0.86* | 0.9 | 0.97 |  | 1.66** | 2.14** | 1.1 | 1.59** | 4.35E-03 |
| parp8 | BC022679 | 0.40** | 0.36** | 1.84* | 0.98 |  | 0.78 | 0.67 | 0.79 | 1.04 | 3.33E-02 |
| pbx1 | L27453 | 0.84 | 0.42** | 0.94 | 1.05 |  | 1.26 | 0.98 | 1.01 | 0.95 | 3.76E-02 |
| pbx1 | BG070361 | 0.61* | 0.33** | 1.67* | 1.08 |  | 1.16 | 0.91 | 0.8 | 1.12 | 2.08E-02 |
| pbxip1 | AV220340 | 0.42** | 0.56** | 0.41** | 0.56** |  | 1.27 | 0.72* | 1.23 | 1.33* | 4.92E-03 |
| pck2 | BB024477 | 0.64* | 0.49** | 1.32 | 1.09 |  | 0.85 | 0.63** | 1.05 | 1.04 | 2.76E-02 |
| pclo | AW493746 | 0.51** | 0.35** | 0.46** | 0.52** |  | 1.05 | 0.98 | 1.1 | 1.09 | 6.91E-03 |
| pcm1 | NM_023662 | 0.48** | 0.60** | 0.77* | 0.64** |  | 1.37** | 0.91 | 1.64** | 1.13 | 1.77E-02 |
| pcmtd2 | BG261527 | 1.22 | 0.78 | 0.38** | 0.36** |  | 1.01 | 0.40** | 0.73 | 0.91 | 4.40E-02 |
| pcna | BC010343 | 1 | 2.44** | 17.91** | 4.75** |  | 1.32 | 3.24** | 0.76 | 3.08** | 3.38E-02 |
| pcnt | NM_008787 | 2.70** | 3.79** | 5.22** | 4.18** |  | 1.14 | 1.89** | 1.1 | 1.82** | 1.37E-02 |
| pcsk2 | NM_008792 | 0.42** | 0.39** | 1.74** | 2.59** |  | 0.94 | 1.01 | 1.1 | 0.96 | 4.15E-02 |
| pcsk9 | AV010795 | 2.69** | 2.00** | 1.32* | 2.22** |  | 1.11 | 1.24 | 1.48* | 0.66** | 2.55E-02 |
| pcyt1b | BE996519 | 0.78** | 0.42** | 0.77** | 0.87* |  | 1.01 | 0.97 | 0.94 | 0.95 | 4.43E-03 |
| pdgfrl | AK004179 | 0.21** | 0.23** | 1.94 | 0.46* |  | 0.92 | 1.19 | 0.57 | 1.34 | 4.38E-02 |
| pdlim3 | NM_016798 | 1 | 1.09 | 1.07 | 0.78 |  | 0.85 | 2.73** | 1.49* | 2.29** | 4.52E-02 |
| pdx1 | AK020261 | 0.74* | 0.20** | 0.71* | 0.47** |  | 1.96** | 1.11 | 1.3 | 0.95 | 3.47E-02 |
| pdx1 | AK020261 | 0.60** | 0.37** | 0.70* | 0.62** |  | 1.11 | 1.08 | 1.2 | 0.92 | 4.14E-02 |
| pex26 | BC019144 | 2.11** | 1.24 | 1.38 | 2.01** |  | 0.95 | 1.01 | 0.97 | 0.92 | 2.12E-02 |
| pfkfb2 | AV256368 | 1.23 | 0.33** | 0.51** | 0.43** |  | 1.37 | 0.51** | 1.1 | 0.66* | 4.51E-02 |
| pgam5 | BC021317 | 2.59** | 2.07** | 2.75** | 4.04** |  | 0.74 | 2.09** | 1.08 | 1.75** | 4.77E-05 |
| pgf | NM_008827 | 0.50** | 0.39** | 1.03 | 0.43** |  | 1.03 | 2.29** | 2.24** | 6.64** | 2.52E-02 |
| pgm2 | BC008527 | 0.94 | 0.68* | 1.25 | 1.16 |  | 1.15 | 2.37** | 1.25 | 2.07** | 1.76E-02 |
| phf7 | AI427892 | 1.49** | 2.30** | 0.99 | 0.79* |  | 0.99 | 1.13 | 1.1 | 0.96 | 3.76E-03 |
| phgdh | AV216768 | 1.50** | 2.48** | 2.08** | 2.31** |  | 0.54** | 1.50** | 0.75* | 1.21 | 7.28E-03 |
| phkb | C80697 | 1.01 | 0.99 | 1.06 | 1.57** |  | 1.15 | 0.49** | 0.87 | 1.12 | 2.90E-02 |
| phldb1 | BC025856 | 1.98** | 1.16 | 0.75 | 2.08** |  | 0.74 | 0.9 | 0.67** | 1.27 | 4.85E-03 |
| pias3 | BC023128 | 0.45** | 0.31** | 2.60** | 0.93 |  | 0.52* | 1.45 | 0.59 | 1.63 | 7.34E-03 |
| pik3ca | AI528567 | 0.78* | 0.49** | 1.52** | 0.89 |  | 0.77* | 0.71** | 0.81 | 1.02 | 2.48E-03 |
| pip5k1b | NM_008846 | 0.28** | 0.74 | 2.51** | 1.07 |  | 1.01 | 0.66 | 1.14 | 1.36 | 8.44E-03 |
| pip5k1b | NM_008846 | 0.64* | 0.45** | 1.75** | 1.55* |  | 0.9 | 0.99 | 1.1 | 0.87 | 1.98E-02 |
| pip5k2c | NM_054097 | 0.60* | 0.38** | 1.94** | 0.78 |  | 0.51** | 1.21 | 0.84 | 1.49 | 4.13E-02 |
| pkd1 | NM_013630 | 0.77 | 0.38** | 1.25 | 0.76 |  | 1.15 | 1.3 | 0.49** | 1.67** | 1.99E-02 |
| pkd1l2 | U58494 | 0.94 | 0.48** | 0.96 | 0.82 |  | 0.62** | 0.94 | 0.63** | 0.9 | 7.93E-03 |
| pkhd1 | AI182499 | 2.17** | 1.03 | 2.04** | 2.38** |  | 1.11 | 1.07 | 1.2 | 0.85 | 2.40E-02 |
| pkp2 | AA516617 | 3.17** | 0.94 | 0.27** | 0.9 |  | 1.21 | 1.09 | 0.81 | 1.01 | 2.91E-02 |
| pkp3 | AW475993 | 1.03 | 1.06 | 1.11 | 1 |  | 1.21 | 2.05** | 1.68* | 2.26** | 3.32E-02 |
| pla2g7 | AK005158 | 0.63 | 0.76 | 0.8 | 1.75* |  | 0.56** | 1.91* | 0.29** | 1.47 | 2.23E-03 |
| plagl1 | AF147785 | 0.95 | 0.43** | 0.78 | 1.17 |  | 1.29 | 0.76 | 1.37 | 0.47** | 2.62E-02 |
| plcd1 | NM_019676 | 0.9 | 0.99 | 1.02 | 1.09 |  | 0.29** | 0.83 | 0.58** | 2.09** | 4.89E-02 |
| plcd1 | NM_019676 | 0.82 | 1.06 | 1.12 | 0.69* |  | 0.49** | 1.39 | 0.65* | 1.27 | 4.34E-02 |
| pld1 | BM228590 | 0.69** | 0.58** | 0.51** | 1.15 |  | 0.53** | 0.41** | 0.65** | 0.57** | 2.05E-02 |
| plec1 | BM210485 | 1.31 | 0.49* | 1.25 | 3.10** |  | 0.53 | 1.38 | 1.04 | 2.87** | 2.42E-02 |
| plec1 | BM232239 | 1.2 | 0.77 | 1.35 | 2.46** |  | 3.23** | 4.56** | 2.82** | 0.59* | 2.09E-03 |
| plek2 | NM_013738 | 2.32** | 1.82** | 2.02** | 1.59** |  | 0.64** | 0.86 | 0.98 | 0.60** | 1.12E-02 |
| plxdc2 | AK017369 | 1.33 | 0.83 | 0.75 | 1.16 |  | 0.74 | 0.36** | 1.01 | 0.32** | 9.60E-03 |
| plxnb1 | BM119522 | 0.40** | 0.59* | 1.2 | 0.62* |  | 1.21 | 0.89 | 1.21 | 0.77 | 4.17E-02 |
| pnp | AK008143 | 1.12 | 0.52* | 2.62** | 1.62* |  | 1.74* | 6.69** | 2.41** | 3.88** | 1.62E-04 |
| pnpt1 | BB777815 | 2.76** | 2.58** | 3.05** | 3.07** |  | 0.87 | 0.84 | 1.15 | 1.83** | 2.58E-02 |
| pogk | AV377712 | 0.95 | 0.43** | 0.69** | 0.63** |  | 0.82* | 0.40** | 0.87 | 0.50** | 4.25E-02 |
| pola1 | NM_008892 | 1.2 | 2.50* | 5.38** | 1.61 |  | 0.74 | 0.69 | 2.07* | 1.24 | 2.95E-02 |
| pold1 | BB385244 | 1.43* | 4.25** | 4.86** | 1.81** |  | 0.64* | 0.53** | 1.07 | 1.01 | 1.24E-02 |
| pold2 | NM_008894 | 2.57** | 3.35** | 6.60** | 3.75** |  | 0.65** | 2.71** | 1.03 | 1.78** | 1.85E-02 |
| pole4 | BF577544 | 1.18 | 1.13 | 1.76** | 2.02** |  | 0.82 | 2.43** | 0.98 | 1.42** | 4.41E-02 |
| polr1e | NM_022811 | 2.92** | 1.4 | 2.70** | 3.63** |  | 0.92 | 1.73** | 1.31 | 1.13 | 2.58E-02 |
| polr1e | BE334128 | 2.38** | 1.71** | 1.18* | 2.52** |  | 0.85 | 1.42** | 1.34* | 0.9 | 3.42E-02 |
| polr2f | BC024419 | 0.95 | 0.99 | 2.81** | 3.12** |  | 1.6 | 2.29** | 0.91 | 0.94 | 2.93E-02 |
| polr2l | AV102258 | 1.14 | 1.56* | 2.48** | 1.52* |  | 0.82 | 2.58** | 0.95 | 1.28 | 1.52E-02 |
| polr3d | BC016102 | 0.96 | 1.1 | 1.3 | 1.18 |  | 1.44* | 3.51** | 1.33 | 2.57** | 2.63E-02 |
| polr3g | NM_026190 | 4.09** | 2.55** | 2.39** | 6.23** |  | 0.82 | 1.26 | 1.64 | 1.52* | 4.94E-05 |
| polr3h | AK019868 | 2.59** | 3.01** | 1.68** | 2.18** |  | 1.49** | 2.01** | 1.76** | 1.75** | 2.87E-02 |
| polr3h | AK019868 | 1.49 | 2.25* | 1.54 | 2.45** |  | 1.19 | 1.2 | 0.83 | 1.29 | 4.26E-02 |
| polr3k | AV260647 | 1.41** | 2.05** | 2.11** | 1.56** |  | 0.94 | 0.88 | 1.57** | 0.91 | 2.15E-02 |
| ppan | BC014688 | 1.95* | 0.87 | 1.46 | 2.29* |  | 1.15 | 2.48** | 1.3 | 1.55 | 1.08E-02 |
| ppargc1a | BB745167 | 0.47** | 0.64 | 0.37** | 0.32** |  | 2.47** | 0.78 | 0.74 | 0.65 | 2.29E-03 |
| ppargc1b | NM_133249 | 4.38** | 0.83 | 1.19 | 1.69* |  | 1.06 | 0.54* | 0.91 | 1.23 | 1.86E-02 |
| ppat | AV305746 | 2.24** | 2.87** | 1.32* | 1.82** |  | 1.37* | 0.78 | 1.46** | 0.88 | 1.57E-02 |
| ppil6 | AK013818 | 0.51** | 0.49** | 0.93 | 0.98 |  | 1.32 | 0.8 | 1.06 | 1.15 | 9.74E-03 |
| ppp1cc | BG071790 | 1.42 | 0.40** | 2.62** | 2.50** |  | 1.01 | 1.07 | 1.37 | 1.91** | 2.52E-02 |
| ppp1r8 | BC025479 | 2.28** | 0.48** | 2.49** | 2.09** |  | 0.83 | 0.83 | 1.09 | 1.62 | 3.17E-02 |
| ppp2r4 | BB369168 | 1.17 | 2.10** | 1.01 | 1.03 |  | 0.73 | 1.48* | 1.03 | 1.3 | 3.73E-02 |
| ppp4c | NM_019674 | 0.97 | 1.09 | 3.09** | 0.97 |  | 0.83 | 2.03** | 1.19 | 1.75** | 1.10E-02 |
| prdm1 | NM_007548 | 0.91 | 0.38** | 1.83** | 0.46** |  | 0.63** | 0.39** | 0.65* | 0.43** | 9.63E-03 |
| prim1 | J04620 | 1.31* | 2.87** | 13.53** | 5.81** |  | 0.99 | 1.50** | 0.77* | 1.75** | 2.29E-03 |
| prim1 | J04620 | 1.36** | 4.20** | 4.98** | 2.90** |  | 0.60** | 0.83 | 0.87 | 1.29* | 2.01E-02 |
| prim2 | NM_008922 | 0.7 | 3.03** | 4.86** | 2.97** |  | 0.72* | 0.96 | 1.09 | 1.52* | 2.40E-02 |
| prmt5 | AF167573 | 1.03 | 0.76 | 5.76** | 2.73** |  | 1.39 | 3.52** | 1.91** | 1.62** | 2.24E-02 |
| prox1 | BE994433 | 0.75 | 0.97 | 0.95 | 0.86 |  | 1.17 | 0.53** | 1.02 | 0.86 | 3.87E-02 |
| prps2 | BC024942 | 1.55** | 2.21** | 1.72** | 1.83** |  | 1.05 | 1.49** | 1.22 | 1.07 | 3.68E-02 |
| prr15 | AJ132433 | 0.30** | 0.21** | 0.28** | 0.58** |  | 0.84 | 0.77 | 0.93 | 0.97 | 7.26E-03 |
| prrx2 | AK019971 | 0.86 | 1.26* | 1.06 | 1.17 |  | 0.98 | 2.24** | 2.14** | 6.54** | 2.66E-02 |
| psat1 | AV216491 | 3.56** | 3.76** | 4.58** | 6.79** |  | 1 | 0.99 | 1.26 | 0.85 | 2.94E-03 |
| ptges3 | BC003708 | 0.92 | 2.02** | 0.78 | 0.99 |  | 0.79 | 1.39* | 1.15 | 2.14** | 1.76E-02 |
| ptrf | BC012674 | 1.1 | 0.57* | 1.48 | 1.13 |  | 1.14 | 2.59** | 1.12 | 2.21** | 2.11E-02 |
| pttg1ip | AU018448 | 0.64** | 0.45** | 1.21 | 1.03 |  | 1.08 | 1.01 | 1.15 | 0.89 | 1.53E-02 |
| pttg1ip | AU018448 | 0.99 | 0.78 | 0.13** | 0.27** |  | 0.77 | 0.39** | 0.99 | 0.65 | 6.14E-03 |
| pxn | BB530368 | 0.87 | 0.77** | 0.88 | 0.73** |  | 1.12 | 0.48** | 1.20* | 0.89 | 1.29E-02 |
| rab11a | BC010722 | 0.54** | 0.37** | 2.21** | 0.93 |  | 0.51** | 1.04 | 0.64** | 1.44* | 3.13E-02 |
| rab19 | BM241400 | 0.61* | 0.36** | 1.91* | 0.44** |  | 0.76 | 1.32 | 0.86 | 1.12 | 9.91E-06 |
| rab22a | BC006596 | 1.26 | 0.36** | 1.03 | 0.50** |  | 0.95 | 0.39** | 1.3 | 0.41** | 2.87E-03 |
| rab27b | BB121269 | 0.99 | 0.97 | 1.08 | 0.96 |  | 0.30** | 0.24** | 0.72* | 0.96 | 4.08E-04 |
| rab27b | BE980253 | 0.78 | 0.98 | 1.24 | 1.45 |  | 0.50** | 0.33** | 0.70* | 0.45** | 6.43E-03 |
| rab33a | NM_011228 | 3.75** | 1.63 | 1.81** | 4.81** |  | 0.99 | 1.01 | 1.26 | 1.47 | 1.63E-03 |
| rab34 | AF327929 | 0.41** | 0.39** | 1.93 | 0.67 |  | 0.57 | 1.16 | 0.63 | 1.19 | 1.24E-02 |
| rab36 | BQ173929 | 0.80* | 0.46** | 1.37* | 0.48** |  | 1.09 | 1.1 | 0.97 | 0.96 | 1.73E-02 |
| rab37 | BB433704 | 0.42** | 0.13** | 0.63* | 0.18** |  | 1.46 | 0.60* | 0.96 | 1.32 | 2.13E-02 |
| rab3d | BB349707 | 0.46** | 0.51** | 2.16** | 1.02 |  | 0.59** | 1.68* | 0.56** | 1.47* | 8.57E-03 |
| rab3d | BB349707 | 0.40** | 0.46** | 0.93 | 0.57** |  | 0.86 | 1.27 | 0.85 | 1.55** | 5.91E-03 |
| rac1 | BC003828 | 0.88 | 0.82 | 1.37 | 0.95 |  | 1.22 | 2.54** | 1.06 | 1.96** | 4.22E-02 |
| racgap1 | NM_012025 | 1.03 | 1.35 | 8.64** | 2.05* |  | 0.47* | 0.88 | 1.02 | 1.59 | 6.56E-03 |
| rad50 | NM_009012 | 1.69* | 3.12** | 1.35 | 3.98** |  | 0.98 | 1.33 | 1.09 | 1.2 | 2.11E-02 |
| rad51 | NM_011234 | 1.02 | 6.19** | 23.30** | 2.40** |  | 0.82 | 1.12 | 1.90** | 1.46 | 1.80E-02 |
| ralb | BB465250 | 0.47** | 1.14 | 0.81* | 0.55** |  | 0.55** | 1.34** | 0.86 | 1.50** | 9.74E-03 |
| ran | AV090150 | 1.81** | 1.68** | 2.64** | 2.17** |  | 0.91 | 2.16** | 1.27 | 1.74** | 3.03E-02 |
| rbbp6 | BC016578 | 2.43** | 0.89 | 0.64** | 0.60** |  | 1.38* | 1.36* | 0.96 | 1.15 | 3.61E-02 |
| rbm14 | BC010294 | 0.54** | 2.04** | 1.58* | 1.22 |  | 0.86 | 1.12 | 1.15 | 1.52** | 4.21E-02 |
| rcc2 | AV122997 | 2.16* | 2.12* | 2.25** | 7.60** |  | 0.68 | 1.71* | 1.43 | 1.76* | 1.30E-02 |
| reck | NM_016678 | 0.35** | 0.54** | 0.9 | 0.86 |  | 0.97 | 0.62* | 0.92 | 0.85 | 1.64E-04 |
| rfc5 | AK011489 | 1.80** | 3.83** | 6.42** | 2.15** |  | 0.73* | 1.31* | 1.04 | 1.62** | 4.27E-03 |
| rffl | AW123157 | 1.62* | 0.55** | 0.48** | 0.68* |  | 0.89 | 0.45** | 0.93 | 0.57** | 1.15E-02 |
| rhoq | BI081723 | 0.50** | 0.79* | 0.87 | 1.1 |  | 0.96 | 0.77* | 1.08 | 0.75* | 6.11E-04 |
| rnf14 | BB504639 | 1.02 | 0.89 | 0.91 | 0.81 |  | 0.83 | 0.50** | 1.11 | 0.63** | 2.13E-02 |
| rnf141 | AF353167 | 0.46** | 1.08 | 2.30** | 1.05 |  | 0.98 | 0.78 | 1.03 | 1.13 | 4.19E-02 |
| rnf167 | AK017523 | 0.66* | 0.23** | 2.17** | 1.24 |  | 0.58* | 1.63 | 0.62* | 0.88 | 2.86E-02 |
| rnf181 | BC005559 | 0.48** | 0.50** | 1.44 | 0.58** |  | 1.02 | 1.49* | 0.89 | 1.04 | 2.93E-02 |
| rnf186 | BC011492 | 0.46** | 0.20** | 1.21 | 0.25** |  | 1.03 | 0.96 | 1.11 | 0.89 | 1.72E-02 |
| rnf215 | C77903 | 0.60** | 0.42** | 0.87 | 0.8 |  | 0.52** | 2.00** | 0.65** | 1.76** | 3.39E-02 |
| rnf4 | AV045658 | 0.88 | 0.93 | 1.43* | 1.69** |  | 1.12 | 2.10** | 1.16 | 1.1 | 2.81E-02 |
| rnf6 | BI738010 | 0.50** | 0.56** | 1.07 | 0.48** |  | 0.89 | 0.86 | 1.28 | 0.81 | 2.74E-02 |
| rnf6 | BI738010 | 0.75* | 0.43** | 1.18 | 0.45** |  | 1.30* | 0.95 | 1.11 | 1.1 | 4.03E-02 |
| robo1 | BG065230 | 0.91 | 0.74 | 0.57 | 0.89 |  | 0.85 | 0.34** | 0.66 | 0.45* | 1.08E-02 |
| robo1 | BB176702 | 1.12 | 1.16 | 0.31** | 1.03 |  | 1.14 | 0.46** | 0.57** | 1.68* | 1.15E-02 |
| rorc | AJ132394 | 1.48 | 0.98 | 1.38 | 1.19 |  | 1.69 | 3.39** | 1.21 | 2.93** | 7.34E-03 |
| rp23-143a14.5 | NM_027136 | 1.05 | 1.09 | 1.60** | 1.45 |  | 0.71 | 2.21** | 0.85 | 1.76** | 4.68E-02 |
| rp23-157o10.7 | BQ174549 | 1.07 | 0.64** | 0.98 | 1.41** |  | 1.17* | 0.47** | 0.9 | 0.99 | 2.30E-02 |
| rpo1-2 | NM_009086 | 3.89** | 1.55* | 2.63** | 3.91** |  | 1 | 1.81** | 1.29 | 1.55* | 5.14E-05 |
| rpo1-3 | NM_009087 | 1.23* | 1.34** | 2.71** | 2.60** |  | 1.1 | 2.00** | 1.1 | 1.39** | 3.63E-02 |
| rpo1-3 | BC024394 | 1.49 | 0.82 | 6.09** | 3.62** |  | 1.42 | 3.35** | 0.83 | 2.01** | 1.27E-02 |
| rps24 | BM119287 | 3.12** | 1.17 | 0.68 | 1.33 |  | 0.7 | 1.54 | 0.84 | 2.09** | 1.79E-02 |
| rras2 | NM_025846 | 2.91** | 1.02 | 1.50** | 1.81** |  | 0.95 | 0.82* | 0.95 | 0.64** | 2.82E-03 |
| rrm1 | BB758819 | 1.08 | 5.33** | 9.83** | 2.49** |  | 0.95 | 1.02 | 1 | 1.38 | 7.19E-03 |
| rrm2 | NM_009104 | 0.98 | 0.97 | 1.21 | 0.88 |  | 0.46** | 0.53** | 0.55** | 1.07 | 9.23E-03 |
| rrp1b | BG293527 | 3.20** | 1.96** | 1.99** | 3.94** |  | 0.85 | 1.04 | 0.97 | 1.40* | 4.37E-02 |
| rrp1b | AV228374 | 3.67** | 3.62** | 0.98 | 2.85** |  | 1.93** | 1.19 | 1.54 | 1.47* | 4.97E-02 |
| rtn4 | BE988775 | 0.67 | 0.49** | 0.61* | 0.64* |  | 1.09 | 0.56* | 1.09 | 0.60* | 1.42E-03 |
| rtn4 | AK003859 | 0.57** | 0.43** | 1.24* | 0.60** |  | 0.98 | 0.98 | 1 | 1.06 | 1.75E-02 |
| runx2 | D14636 | 1.04 | 0.98 | 1.29* | 1.32* |  | 0.50** | 0.58** | 0.77* | 0.79* | 6.48E-03 |
| ruvbl1 | NM_019685 | 2.34** | 1.74* | 4.19** | 6.62** |  | 0.97 | 2.31** | 1.36 | 2.04** | 3.52E-02 |
| rxrg | NM_009107 | 0.97 | 0.99 | 1.11 | 1.08 |  | 1.39** | 2.29** | 0.83 | 1.51** | 1.29E-02 |
| s100a11 | BC021916 | 0.91 | 0.37** | 1.32 | 1.84* |  | 0.54* | 2.58** | 0.69 | 0.47** | 1.30E-02 |
| s100a6 | NM_011313 | 0.49** | 0.92 | 1.36* | 1.51** |  | 0.57** | 1.45** | 0.68** | 1.42** | 4.32E-02 |
| s100a9 | NM_009114 | 1.32 | 0.88 | 1.73* | 0.93 |  | 0.39** | 0.71 | 0.77 | 0.23** | 4.69E-02 |
| sae1 | AK011772 | 1.08 | 1.01 | 1.01 | 1.51* |  | 1.44* | 2.70** | 1.35 | 1.03 | 2.87E-02 |
| scin | NM_009132 | 1.21 | 0.91 | 0.60** | 1.14 |  | 1.07 | 0.51** | 1.08 | 0.63** | 7.55E-03 |
| scmh1 | BB297140 | 0.97 | 0.9 | 1.01 | 1.05 |  | 0.91 | 0.41** | 0.62** | 1.01 | 2.94E-02 |
| scn8a | BB429612 | 2.60** | 0.68 | 1 | 2.21** |  | 1.09 | 1.05 | 0.96 | 1 | 8.32E-03 |
| sdc1 | BI788645 | 1.1 | 1.95* | 0.92 | 1.95* |  | 0.39** | 1 | 1.41 | 1.18 | 1.71E-02 |
| sdc1 | BB533095 | 1.72* | 4.86** | 0.88 | 4.40** |  | 0.73 | 0.7 | 1.16 | 0.81 | 4.89E-02 |
| sdc3 | BB528350 | 1.34 | 0.67 | 1.14 | 1.32 |  | 1.22 | 2.11** | 0.84 | 4.26** | 4.59E-02 |
| sdc4 | BC005679 | 0.33** | 1.56 | 0.46** | 0.39** |  | 1.05 | 1.16 | 1.03 | 0.45** | 1.71E-02 |
| sdcbp2 | BC005556 | 0.49** | 0.56** | 2.25** | 0.98 |  | 0.71* | 1.12 | 0.83 | 1.13 | 1.40E-02 |
| sema3d | BB499147 | 1.02 | 1.26 | 0.93 | 0.9 |  | 0.75 | 0.32** | 1.38 | 0.55** | 2.72E-02 |
| sema3e | NM_011348 | 1.17 | 1.12 | 1.08 | 1.17 |  | 0.61** | 0.39** | 0.83* | 0.69** | 1.44E-03 |
| sema3e | Z93948 | 1.08 | 1.22 | 1.07 | 1.01 |  | 0.82* | 0.37** | 1.17 | 0.53** | 1.81E-02 |
| sema6d | BB462688 | 0.51** | 0.51** | 1.50* | 0.63** |  | 0.70* | 1.26 | 0.9 | 1.34 | 1.63E-02 |
| Sep-09 | NM_017380 | 1.28 | 0.84 | 1.45* | 0.94 |  | 0.78 | 2.10** | 0.94 | 2.03** | 5.01E-03 |
| serinc3 | BM239368 | 0.69* | 0.91 | 0.94 | 0.95 |  | 0.95 | 0.45** | 0.65* | 0.9 | 3.83E-02 |
| serpinb12 | AK009018 | 1.08 | 1.22 | 1.03 | 0.87 |  | 0.40** | 1.05 | 0.37** | 1.02 | 2.96E-02 |
| sgce | NM_011360 | 0.50* | 0.98 | 1.22 | 1.66* |  | 0.93 | 0.84 | 0.74 | 0.9 | 2.07E-02 |
| sh2b2 | NM_018825 | 2.10** | 0.85 | 1.75** | 5.45** |  | 0.53** | 1.01 | 0.61* | 1.55* | 4.11E-02 |
| shmt2 | BM222403 | 1.19 | 1.68* | 2.04** | 1.42 |  | 0.67 | 2.08** | 0.79 | 1.52 | 4.62E-02 |
| shmt2 | BB758291 | 3.29** | 2.27** | 4.19** | 7.26** |  | 0.75 | 1.81* | 1.47 | 0.92 | 4.52E-02 |
| shroom3 | NM_015756 | 0.9 | 0.64** | 1.34* | 1.29 |  | 0.65** | 0.35** | 0.95 | 0.63** | 4.40E-02 |
| siah2 | AA414485 | 0.47** | 0.64** | 1.37** | 0.81 |  | 0.56** | 1.65** | 0.63** | 2.85** | 1.70E-02 |
| siva1 | NM_013929 | 1.45* | 0.99 | 4.89** | 1.85** |  | 1.03 | 2.18** | 0.85 | 1.46* | 1.03E-02 |
| siva1 | AF033112 | 1.23 | 2.23** | 3.10** | 1.38 |  | 0.82 | 1.99** | 0.79 | 1.33 | 2.07E-02 |
| siva1 | AF033112 | 1.26 | 1.97** | 3.56** | 1.89** |  | 0.99 | 2.23** | 1.21 | 1.36 | 6.50E-03 |
| six6os1 | AK015397 | 2.77** | 0.48** | 0.52** | 0.95 |  | 1.12 | 1.06 | 0.98 | 0.91 | 4.18E-03 |
| slc30a1 | BE685959 | 1.92** | 1.27* | 3.45** | 1.65** |  | 1.08 | 0.35** | 1.23* | 0.69** | 3.93E-02 |
| slc5a1 | AF208031 | 0.89 | 1 | 1.11 | 0.93 |  | 0.32** | 0.31** | 0.70* | 0.26** | 1.29E-03 |
| slc5a1 | AV371434 | 1.09 | 1.03 | 0.9 | 0.94 |  | 0.50** | 0.41** | 0.73** | 0.54** | 1.65E-03 |
| smarca1 | NM_053123 | 0.43** | 0.32** | 0.57** | 0.62* |  | 1.13 | 0.76 | 1.27 | 0.99 | 3.29E-02 |
| smarcb1 | NM_011418 | 0.95 | 0.95 | 2.41** | 1.51** |  | 0.47** | 1.15 | 0.83 | 2.08** | 9.93E-03 |
| smpd3 | BF456582 | 1.32 | 1.95** | 1.80** | 2.88** |  | 0.87 | 0.92 | 0.93 | 0.86 | 4.73E-02 |
| smpdl3b | NM_133888 | 9.87** | 12.60** | 11.99** | 18.85** |  | 1.36 | 1.11 | 3.43** | 1.26 | 3.35E-03 |
| smurf1 | BB201890 | 0.99 | 0.94 | 0.71* | 0.91 |  | 1.23 | 0.46** | 0.71** | 0.89 | 8.92E-03 |
| smyd1 | C78565 | 1.02 | 1.19* | 0.96 | 0.94 |  | 0.63** | 2.59** | 1.01 | 2.22** | 1.60E-02 |
| smyd1 | NM_009762 | 1.01 | 0.98 | 1.13 | 1.07 |  | 0.71* | 3.14** | 1.18 | 1.85** | 2.88E-02 |
| socs2 | NM_007706 | 0.82 | 0.39** | 1.58* | 0.93 |  | 1.24 | 1.31 | 0.92 | 0.91 | 1.16E-02 |
| sod2 | NM_013671 | 1.1 | 0.86 | 2.03** | 2.13** |  | 0.67* | 2.03** | 0.85 | 1.80** | 2.79E-02 |
| sorl1 | AK013519 | 1.14 | 0.34** | 1.16 | 0.62* |  | 0.88 | 0.50** | 0.95 | 0.53** | 1.73E-02 |
| sox7 | NM_011446 | 0.87 | 0.78 | 0.71* | 0.81 |  | 0.79 | 0.48** | 0.99 | 0.88 | 1.14E-02 |
| sp4 | NM_009239 | 0.78* | 0.47** | 0.8 | 1.15 |  | 1 | 0.77* | 0.88 | 0.74* | 1.08E-03 |
| sp4 | AI324972 | 0.54* | 0.72* | 0.89 | 0.33** |  | 1.32 | 0.38** | 1.43 | 0.67 | 9.49E-03 |
| spag9 | BM938614 | 0.88 | 0.49** | 1.45** | 1.07 |  | 0.82 | 1.08 | 0.82 | 1.22 | 2.87E-02 |
| spna2 | AK011566 | 0.8 | 0.45** | 1.21 | 1.03 |  | 0.81 | 0.67** | 0.69 | 0.89 | 2.19E-03 |
| spnb2 | BM213516 | 1.19 | 0.61** | 0.60** | 0.96 |  | 1.42** | 0.44** | 1.1 | 0.63** | 2.42E-02 |
| spnb2 | AV016275 | 0.68 | 1.08 | 0.84 | 1.84** |  | 1.16 | 0.41** | 0.93 | 0.65* | 7.36E-03 |
| spon2 | NM_133903 | 0.39** | 0.6 | 1.52 | 0.59 |  | 0.97 | 1.23 | 0.44* | 2.22* | 3.42E-02 |
| sprr1b | NM_009265 | 1.14 | 1.13 | 1.1 | 0.76 |  | 0.24** | 0.33** | 0.67 | 0.27** | 1.27E-02 |
| spry2 | BB529691 | 0.51* | 0.57* | 1.71 | 1.91* |  | 0.77 | 0.43** | 1.09 | 0.65 | 3.55E-02 |
| spsb3 | AV245208 | 2.26** | 1.14 | 0.92 | 1.4 |  | 1.02 | 1.65* | 1.29 | 0.96 | 1.09E-02 |
| sqstm1 | BM232298 | 0.44** | 0.28** | 2.68** | 0.57* |  | 1.93* | 2.25** | 0.79 | 0.91 | 3.53E-02 |
| srr | AK002636 | 0.60** | 0.93 | 1.17 | 1.18 |  | 1.89** | 0.82 | 1.3 | 0.93 | 1.64E-03 |
| ss18l1 | BG075210 | 0.71** | 0.36** | 0.95 | 0.80* |  | 0.91 | 0.95 | 1.02 | 1.23* | 1.42E-02 |
| st18 | AV347235 | 0.49** | 0.41** | 0.82 | 1.05 |  | 1.05 | 0.71* | 0.9 | 0.98 | 2.05E-02 |
| stat5a | U36502 | 0.56** | 0.40** | 0.82 | 1.01 |  | 0.71* | 0.69* | 0.91 | 0.9 | 3.12E-03 |
| stk38l | AV257215 | 0.85 | 1.35 | 1.36 | 0.84 |  | 0.48** | 0.35** | 0.97 | 0.82 | 4.13E-03 |
| stmn1 | BC010581 | 0.69 | 2.83** | 6.24** | 1.74** |  | 1.04 | 0.91 | 1.02 | 0.93 | 3.30E-02 |
| strap | AW557906 | 2.21** | 1.63** | 1.70** | 2.07** |  | 1.08 | 0.76* | 1.55** | 1.14 | 2.00E-02 |
| strap | AW557906 | 2.07** | 1.6 | 0.76 | 2.14** |  | 0.77 | 0.57* | 1.48 | 0.98 | 3.02E-03 |
| sult1a1 | AK002700 | 0.39** | 0.72 | 1.99* | 1.53 |  | 1 | 1.48 | 0.28** | 0.74 | 5.87E-03 |
| supt3h | BM114601 | 1.09 | 1.1 | 1.23 | 1.63* |  | 1.22 | 2.09** | 0.79 | 1.81** | 4.54E-02 |
| svil | BM203457 | 0.81 | 0.50** | 0.81 | 0.94 |  | 0.88 | 0.70* | 0.92 | 0.92 | 2.18E-02 |
| syne1 | AV237615 | 1.17* | 0.28** | 0.83** | 0.79** |  | 0.97 | 0.86* | 0.99 | 0.98 | 7.10E-04 |
| syne1 | BI734306 | 0.94 | 0.66* | 0.66* | 0.84 |  | 1.19 | 0.37** | 0.94 | 0.71 | 1.83E-02 |
| tacstd1 | BC005618 | 0.53* | 0.30** | 0.9 | 0.34** |  | 1.35 | 0.99 | 0.86 | 0.49** | 1.26E-03 |
| taf1 | BB733926 | 0.33** | 0.52* | 1.31 | 1.19 |  | 1.1 | 1.05 | 1.12 | 0.55** | 1.58E-02 |
| taf1c | BI903692 | 3.00** | 2.34** | 0.95 | 1.64** |  | 0.83 | 0.79 | 0.88 | 1.17 | 1.99E-02 |
| taf4b | AK012135 | 2.77** | 1.6 | 0.73 | 1.86* |  | 1.57 | 0.95 | 1.03 | 1.1 | 2.17E-03 |
| taf4b | AV373814 | 3.69** | 2.65** | 1.45* | 3.99** |  | 1.3 | 1.02 | 1.52* | 1.26 | 1.06E-04 |
| taf4b | AV373814 | 2.29 | 1.18 | 1.28 | 2.91* |  | 1.04 | 1.28 | 1.22 | 1.06 | 2.42E-02 |
| taf9b | AW555571 | 0.50** | 0.49** | 1.37* | 0.62** |  | 0.54** | 0.77* | 0.85 | 1.49** | 3.73E-03 |
| tagln | BB114067 | 0.98 | 0.56* | 1.31 | 3.52** |  | 1.56 | 2.83** | 0.47** | 2.56** | 2.85E-02 |
| tars2 | BB549252 | 2.03** | 2.73** | 1.41** | 1.50** |  | 0.74** | 1.16 | 1.16 | 1.50** | 1.18E-05 |
| tax1bp1 | C85320 | 1.25 | 0.62** | 0.55** | 1.3 |  | 1 | 0.47** | 1.05 | 0.87 | 4.40E-02 |
| tbcb | NM_025548 | 0.74* | 0.78* | 2.37** | 1.2 |  | 0.66** | 2.02** | 1.07 | 1.94** | 2.10E-02 |
| tbcel | BB540721 | 0.71 | 0.48** | 0.7 | 0.49** |  | 0.72 | 0.7 | 0.49** | 1.95* | 3.43E-02 |
| tbpl1 | AK005604 | 0.79 | 0.66** | 1.94** | 1.29* |  | 0.85 | 2.08** | 0.93 | 1.84** | 1.82E-02 |
| tcfap2a | NM_011547 | 1.01 | 1.14 | 0.93 | 0.99 |  | 0.88 | 0.53** | 1.17 | 0.49** | 7.05E-03 |
| tcfap2c | BC003778 | 1.05 | 1.23 | 0.98 | 0.89 |  | 0.78* | 0.49** | 1.09 | 0.93 | 4.01E-02 |
| tcta | BC019397 | 0.62* | 0.36** | 1.46* | 0.88 |  | 0.68* | 1.45* | 0.92 | 1.16 | 1.97E-03 |
| tera | NM_019643 | 3.25** | 1.59* | 1.27* | 2.14** |  | 1.54** | 2.10** | 0.98 | 1.87** | 3.03E-05 |
| tfdp1 | BG075396 | 3.08** | 1.70** | 3.28** | 3.13** |  | 1.11 | 1.43* | 1.52* | 1.27 | 9.57E-03 |
| tgfa | M92420 | 0.78 | 0.87 | 1.09 | 0.56** |  | 0.8 | 0.32** | 0.77 | 0.42** | 4.84E-02 |
| tial1 | NM_009383 | 1.06 | 0.97 | 1.97** | 0.76* |  | 0.71** | 2.25** | 0.88 | 2.52** | 1.68E-03 |
| timeless | BM230269 | 1.05 | 3.50** | 2.49** | 3.47** |  | 0.73 | 1.77** | 1.23 | 2.11** | 1.30E-03 |
| timeless | BM230269 | 1 | 0.94 | 3.00** | 1.63** |  | 0.77 | 0.50** | 1.45* | 1.83** | 3.15E-02 |
| tlr1 | AF316985 | 0.94 | 1.01 | 0.73* | 1.32 |  | 2.12** | 3.71** | 1.69** | 4.60** | 7.66E-03 |
| tmod1 | NM_021883 | 0.50** | 0.33** | 0.66** | 0.37** |  | 0.93 | 0.85 | 1.73** | 1.31 | 7.58E-03 |
| tmod2 | AK018223 | 0.96 | 0.38** | 1.03 | 2.10** |  | 0.76 | 0.46** | 0.72 | 0.76 | 2.55E-02 |
| tmod2 | BB667124 | 1.04 | 0.92 | 0.57** | 0.82 |  | 0.95 | 0.38** | 0.78 | 1.31 | 4.53E-02 |
| tmod3 | BB476471 | 1.01 | 1.11 | 0.73** | 1.12 |  | 0.74** | 0.47** | 0.71** | 1.1 | 2.43E-02 |
| tnfrsf12a | NM_013749 | 0.59** | 0.85 | 2.53** | 0.72** |  | 1.11 | 3.15** | 1.38** | 1.17 | 4.16E-02 |
| tnfrsf12a | NM_013749 | 0.86 | 0.87 | 2.29** | 0.85 |  | 1.18 | 2.77** | 1.39** | 1.11 | 4.89E-03 |
| tnfrsf22 | BB366863 | 0.37** | 0.70* | 1.58* | 0.86 |  | 0.73* | 0.94 | 1.06 | 1.81** | 3.76E-02 |
| tnfrsf4 | NM_011659 | 1.18 | 0.60* | 1.46* | 1.64** |  | 1.27 | 2.12** | 1.76** | 1.03 | 5.78E-03 |
| tnfsf13 | NM_023517 | 0.33** | 0.99 | 1.57 | 1.81 |  | 0.87 | 1.98* | 0.38** | 1.34 | 4.42E-02 |
| tnfsf5ip1 | BC016606 | 1.16 | 0.92 | 3.22** | 2.64** |  | 0.98 | 3.12** | 1.03 | 1.99** | 1.95E-03 |
| tnfsf5ip1 | NM_134138 | 2.23** | 1.34 | 2.43** | 5.11** |  | 1.21 | 1.42 | 1.25 | 1.15 | 5.23E-03 |
| tnfsf9 | NM_009404 | 1.02 | 1.04 | 2.76** | 1.28 |  | 0.92 | 2.05** | 1.06 | 0.81 | 1.21E-02 |
| tnks2 | BB711615 | 1.14 | 0.69** | 0.63** | 1.1 |  | 1.32** | 0.47** | 0.84 | 1.31** | 1.16E-02 |
| tnrc6a | BI080625 | 0.68 | 0.23** | 0.69 | 1.41 |  | 1.38 | 0.59 | 0.98 | 0.69 | 2.59E-02 |
| tns4 | BB142697 | 0.97 | 0.98 | 1.14 | 1 |  | 0.68* | 0.42** | 0.89 | 0.82 | 5.47E-03 |
| top1 | BB127876 | 2.44** | 0.82 | 0.85 | 1.06 |  | 1.35 | 1.02 | 0.73 | 2.16** | 1.44E-02 |
| top1mt | AF362952 | 4.59** | 3.29** | 3.17** | 4.42** |  | 0.93 | 1.37 | 0.89 | 1.22 | 1.56E-02 |
| topbp1 | BC007170 | 2.03** | 2.70** | 5.72** | 6.88** |  | 0.60** | 0.75 | 1.07 | 1.09 | 4.91E-07 |
| tpd52l2 | BG069764 | 1.21 | 0.85 | 1.88** | 1.35 |  | 0.75* | 2.13** | 0.86 | 1.79** | 3.71E-02 |
| tpm2 | AK003186 | 1 | 0.93 | 1.25 | 1.13 |  | 1.11 | 6.32** | 1.33 | 1.46 | 4.67E-02 |
| traf6 | AV244412 | 0.76* | 0.33** | 0.99 | 1.04 |  | 1.11 | 1.12 | 1.16 | 0.97 | 2.96E-02 |
| trdmt1 | BB010597 | 2.43** | 0.99 | 2.09** | 1.04 |  | 0.79 | 1.51* | 1.16 | 1.46* | 2.45E-02 |
| trim27 | NM_009054 | 1.55** | 1.54** | 1.47** | 1.89** |  | 0.68* | 2.67** | 0.53** | 2.43** | 2.66E-02 |
| trim30 | BM240719 | 0.9 | 0.86 | 0.56** | 1.44* |  | 2.48** | 0.95 | 0.65 | 0.54** | 4.36E-02 |
| trip4 | AV350958 | 0.81* | 0.38** | 0.96 | 0.70** |  | 0.85 | 0.60** | 1.08 | 0.87 | 6.66E-03 |
| tro | AF288606 | 1.06 | 0.49** | 0.97 | 0.73** |  | 1.1 | 0.92 | 1.04 | 0.91 | 3.98E-02 |
| trp53bp1 | BB503221 | 1.14 | 1.28 | 0.94 | 0.87 |  | 0.84 | 0.45** | 1.33* | 0.87 | 6.71E-04 |
| trp53bp2 | BB814564 | 0.86 | 0.41** | 1.50* | 0.98 |  | 0.61** | 0.79 | 0.81 | 0.92 | 3.77E-02 |
| tshz1 | AV291373 | 1.33* | 0.64** | 0.83 | 1 |  | 0.83 | 0.26** | 1.15 | 0.51** | 5.36E-03 |
| tshz2 | BC028776 | 0.24** | 1.14 | 0.76 | 1.05 |  | 1.05 | 1.01 | 1.03 | 1.38 | 1.23E-02 |
| ttc3 | BB426368 | 0.96 | 0.46** | 0.76 | 1.28 |  | 1.24 | 0.56** | 1.29 | 0.71* | 6.65E-03 |
| ttll5 | AK009255 | 1.2 | 0.33** | 0.64* | 0.63** |  | 1.07 | 1.05 | 0.82 | 0.86 | 3.88E-03 |
| tuba1b | BC008117 | 1.37** | 2.53** | 1.65** | 1.39** |  | 1.1 | 1.25* | 1.35** | 0.94 | 2.59E-02 |
| tuba3a | NM_009446 | 0.97 | 0.97 | 1.13 | 1 |  | 0.83 | 3.09** | 0.89 | 3.20** | 1.22E-02 |
| tuba4a | AW491660 | 1.78** | 2.11** | 3.53** | 1.34 |  | 0.95 | 1.77** | 0.99 | 1.45* | 1.52E-02 |
| tubb1 | AW493179 | 0.43** | 0.95 | 1.18 | 0.81* |  | 0.88* | 1.01 | 1.02 | 0.95 | 6.57E-03 |
| tubb2b | AA986082 | 0.29** | 0.96 | 1.16 | 5.99** |  | 0.66 | 0.72 | 0.89 | 0.59 | 2.71E-02 |
| tubb5 | BG064086 | 1.08 | 2.68** | 2.13** | 2.17** |  | 0.79 | 1.41* | 1.18 | 1.54* | 3.70E-02 |
| tubb6 | NM_026473 | 1.21 | 0.9 | 2.95** | 1.71* |  | 0.97 | 2.99** | 1.28 | 3.49** | 2.41E-02 |
| tube1 | AK010005 | 2.66** | 2.38** | 2.19** | 3.24** |  | 0.9 | 0.76 | 0.88 | 0.99 | 8.54E-03 |
| tube1 | AV100095 | 2.06* | 5.81** | 2.83** | 6.59** |  | 1.34 | 0.79 | 0.95 | 0.94 | 2.29E-02 |
| ube1l | AK004894 | 0.75 | 0.8 | 0.60** | 1.11 |  | 1.04 | 0.48** | 0.53** | 1.37 | 3.72E-02 |
| ube1l2 | BB417360 | 1.2 | 1.13 | 0.78 | 1.64** |  | 2.23** | 1.73** | 2.04** | 0.94 | 4.55E-02 |
| ube2h | BB183512 | 0.8 | 0.37** | 1.54 | 1.8 |  | 0.50* | 1.36 | 0.53* | 1.3 | 1.57E-02 |
| ube2h | BB447627 | 0.82* | 0.49** | 0.72** | 0.91 |  | 1.12 | 0.98 | 0.93 | 0.69** | 8.54E-04 |
| ube2w | BB796558 | 2.01** | 0.99 | 1.13 | 1.59* |  | 0.83 | 1.04 | 1 | 1.25 | 5.74E-03 |
| ube3a | AI154956 | 2.45** | 0.6 | 1.01 | 1.94** |  | 1.44 | 0.53** | 1.44 | 1.35 | 4.67E-03 |
| ubr2 | AK018693 | 0.71 | 0.49** | 0.83 | 0.78 |  | 1.2 | 0.74 | 0.86 | 1.01 | 2.67E-02 |
| uhrf1 | BB702754 | 1.68 | 7.49** | 7.70** | 3.80** |  | 0.68 | 0.76 | 1.47 | 1.42 | 3.06E-02 |
| ung | BC004037 | 9.50** | 13.27** | 0.77 | 2.88** |  | 1.39 | 1.04 | 1.36 | 1.21 | 3.68E-03 |
| unkl | AK004898 | 0.45** | 0.77** | 1 | 0.82* |  | 0.71** | 0.65** | 0.86 | 0.70** | 2.30E-02 |
| unknown | BQ175796 | 0.29** | 0.75* | 0.76* | 0.40** |  | 0.8 | 0.70* | 0.72* | 0.73* | 7.66E-03 |
| unknown | BF471533 | 0.56** | 0.40** | 0.88 | 1.12 |  | 0.76* | 0.64** | 1.32* | 0.92 | 1.96E-02 |
| unknown | BB235876 | 1.4 | 0.99 | 0.86 | 0.99 |  | 3.60** | 1.14 | 5.35** | 0.95 | 3.03E-02 |
| unknown | BB189640 | 0.48** | 0.62** | 0.65** | 0.71* |  | 0.77* | 0.79 | 0.9 | 0.99 | 1.07E-02 |
| unknown | BB209878 | 1.21 | 4.62** | 1.57** | 1.02 |  | 1.01 | 1.39* | 1.13 | 0.91 | 1.61E-02 |
| unknown | BB729922 | 0.77 | 0.36** | 0.87 | 0.86 |  | 0.93 | 0.63 | 0.57 | 0.68 | 4.35E-03 |
| unknown | AW047257 | 1.07 | 0.20** | 0.95 | 0.71 |  | 0.52* | 0.47** | 0.75 | 0.7 | 3.76E-02 |
| unknown | BB071777 | 1.44** | 1.24 | 0.78* | 1.58** |  | 0.86 | 0.44** | 0.79* | 0.52** | 1.40E-02 |
| unknown | AI414004 | 2.30** | 1.75** | 1.05 | 1.65** |  | 0.79 | 0.65** | 1.02 | 1.13 | 2.87E-02 |
| unknown | AU042527 | 1.07 | 1.02 | 1.26 | 0.94 |  | 1.33 | 0.42** | 0.79 | 0.9 | 1.45E-02 |
| unknown | BG069583 | 2.56** | 1.58** | 1.17 | 0.93 |  | 1.56** | 0.79 | 1.29* | 0.79 | 2.31E-02 |
| usp4 | BF321773 | 1.32* | 0.81 | 0.99 | 0.71** |  | 1.15 | 2.08** | 0.77* | 1.44** | 1.72E-02 |
| utrn | AW541437 | 1 | 0.97 | 0.74 | 0.98 |  | 0.45** | 0.68 | 0.34** | 1.37 | 1.12E-02 |
| uxt | NM_013840 | 0.81 | 1.08 | 2.90** | 1.52** |  | 0.82 | 2.24** | 0.85 | 1.48** | 6.49E-03 |
| vasp | BC015289 | 0.57** | 0.30** | 2.03** | 0.45** |  | 0.56** | 1.42 | 0.69* | 2.20** | 3.93E-02 |
| vegfc | BB089170 | 1.18 | 0.69** | 0.60** | 1.72** |  | 0.76* | 0.40** | 0.81 | 0.77* | 2.21E-02 |
| vps13a | AV255519 | 2.07** | 1.85** | 1.62** | 2.27** |  | 1.08 | 0.53** | 1.35 | 0.60** | 2.58E-02 |
| wasf2 | BB500616 | 0.44** | 0.61* | 0.71 | 0.99 |  | 0.66 | 0.86 | 0.94 | 0.8 | 4.60E-02 |
| wdr46 | C78559 | 1.59* | 1.09 | 1.56** | 1.69** |  | 0.69* | 2.02** | 1.19 | 1.78** | 2.20E-02 |
| wisp2 | NM_016873 | 0.89 | 0.98 | 0.89 | 1.14 |  | 1.63** | 2.56** | 1.55** | 1.73** | 6.49E-03 |
| wnt4 | NM_009523 | 0.76 | 0.24** | 1.41 | 0.52* |  | 0.56 | 1.12 | 0.71 | 0.76 | 3.11E-02 |
| wnt5b | AV303043 | 2.83** | 1.02 | 2.04** | 2.01** |  | 0.91 | 0.83 | 0.84 | 0.57** | 4.45E-02 |
| wwp2 | AK004087 | 0.44** | 0.34** | 1.25 | 0.83 |  | 0.8 | 1.22 | 0.85 | 1.17 | 1.32E-02 |
| yars2 | AK008774 | 2.29** | 1.55* | 3.87** | 2.43** |  | 1.01 | 1.83** | 0.92 | 2.71** | 1.20E-02 |
| yars2 | BB204225 | 3.16** | 1.94** | 1.45** | 3.05** |  | 0.8 | 0.87 | 1.35 | 0.85 | 2.79E-02 |
| zbtb16 | Z47205 | 0.96 | 0.67* | 0.79 | 0.61* |  | 1.83** | 0.51** | 0.87 | 0.43** | 3.11E-02 |
| zcchc12 | AK012833 | 0.35** | 0.48** | 1.89** | 1.31 |  | 1.07 | 1.12 | 1.38* | 1.01 | 2.04E-02 |
| zeb2 | AK012377 | 0.92 | 0.91 | 0.71** | 0.98 |  | 3.16** | 2.13** | 1.25 | 1.62** | 6.83E-03 |
| zfand3 | BG976649 | 1.04 | 0.68* | 1.73** | 2.04** |  | 0.98 | 2.03** | 1.11 | 1.76** | 1.09E-02 |
| zfp367 | BE629588 | 1.27 | 3.47** | 10.42** | 2.27* |  | 0.68 | 0.56 | 0.82 | 2.15* | 2.49E-02 |
| zfp39 | BB311524 | 1.22 | 0.58** | 0.89 | 1.03 |  | 1.52** | 0.25** | 0.92 | 0.91 | 8.76E-03 |

**Table S4: Cell death and apoptosis genes showing significant change in expression within 8 hours of MYC-ER^TAM^ activation.**

Genes relating to cell death and apoptosis showing a significant change in expression within 8 hours following activation of MYC-ER^TAM^. ‘MYC-response p-value’ is the p-value identified for the highest-order interaction of the MYC activation variable and represents the significance of this term within the selected model. Flags represent contrast p-values comparing 4OHT-treated and vehicle-treated samples at specific time points (‘*’, p ≤ 0.05; ‘**’ p ≤ 0.01). Cells are colour-coded based on a detected fold-change greater than 1.5-fold (red, up-regulated; blue, down-regulated).

|  |  | **Pancreatic β-cells** | | | |  | **Suprabasal Keratinocytes** | | | | **Myc-response p-value** |
| --- | --- | --- | --- | --- | --- | --- | --- | --- | --- | --- | --- |
| **Gene Symbol** | **RefSeq** | **4 hrs** | **8 hrs** | **16 hrs** | **32 hrs** |  | **4 hrs** | **8 hrs** | **16 hrs** | **32 hrs** |
| aatk | NM_007377 | 0.64** | 0.40** | 0.92 | 1.11 |  | 0.81* | 1.11 | 0.96 | 1.05 | 1.73E-02 |
| agt | AK018763 | 0.98 | 0.28** | 1.64** | 2.21** |  | 1.17 | 1.06 | 1.14 | 2.08** | 4.69E-02 |
| ahr | BE989096 | 1.02 | 0.65* | 0.93 | 1.49* |  | 1.14 | 0.45** | 0.89 | 0.49** | 3.14E-02 |
| ai467657 | AA419994 | 0.57** | 1.57* | 1.29 | 0.51** |  | 0.86 | 0.33** | 0.99 | 0.45** | 1.30E-02 |
| akt1 | NM_009652 | 1.01 | 0.97 | 1.71** | 2.31** |  | 0.82 | 3.23** | 0.95 | 2.29** | 4.43E-02 |
| akt2 | NM_007434 | 1 | 1.05 | 0.98 | 1.13 |  | 1.27 | 2.21** | 1.02 | 2.26** | 7.85E-03 |
| apaf1 | AK018076 | 0.54 | 1.06 | 1.04 | 1.12 |  | 0.83 | 0.65 | 0.54* | 1.04 | 2.05E-02 |
| asah2 | NM_018830 | 0.44** | 0.25** | 0.53** | 0.70* |  | 0.66** | 0.46** | 0.9 | 0.87 | 3.77E-02 |
| atf5 | AF375476 | 1.05 | 0.66 | 1.57* | 1 |  | 0.92 | 2.94** | 0.88 | 3.18** | 3.70E-02 |
| b230120h23rik | BB561086 | 1.27 | 1.42 | 1.41 | 1.26 |  | 2.25** | 1.49* | 1.67* | 0.61* | 1.61E-03 |
| bag1 | NM_009736 | 1.38 | 1.46* | 3.04** | 2.00** |  | 0.72 | 2.28** | 1.03 | 1.95** | 3.80E-03 |
| bcl2 | BI664467 | 1.23 | 2.27** | 1 | 1.3 |  | 1.31 | 1.21 | 0.99 | 0.93 | 7.79E-03 |
| bcl2l11 | BB667581 | 1.11 | 0.56** | 0.64** | 1.01 |  | 1.92** | 0.42** | 0.96 | 0.62** | 2.07E-02 |
| bcl2l2 | BB485989 | 1.19 | 0.40** | 0.54** | 0.53** |  | 1.05 | 0.72** | 0.94 | 0.71** | 1.66E-02 |
| birc4 | BF134200 | 0.69* | 0.58* | 0.78 | 0.81 |  | 0.53** | 0.31** | 1.14 | 0.75 | 5.29E-03 |
| birc6 | BG071331 | 2.37** | 0.57* | 0.8 | 0.94 |  | 1.39 | 0.92 | 0.87 | 1.26 | 1.87E-02 |
| bmf | BB212341 | 1.08 | 0.88* | 0.80* | 1.84** |  | 0.61** | 0.29** | 0.49** | 0.64** | 1.20E-02 |
| bnip1 | BG073508 | 2.06** | 1 | 3.84** | 4.62** |  | 0.60** | 1.96** | 0.78 | 2.40** | 6.79E-03 |
| bnip3 | NM_009760 | 0.78 | 0.46** | 1.53** | 0.65** |  | 1.48* | 0.81 | 0.95 | 0.88 | 1.49E-02 |
| btg1 | L16846 | 0.47** | 0.35** | 1.14 | 0.56** |  | 0.65* | 0.79 | 0.75 | 0.97 | 2.21E-03 |
| btg1 | AW322026 | 0.61** | 0.40** | 0.93 | 0.73** |  | 0.78* | 0.71** | 0.82 | 0.76* | 2.36E-03 |
| btg2 | NM_007570 | 0.29** | 0.32** | 1.17 | 0.7 |  | 1.72 | 1.96* | 0.88 | 0.68 | 1.57E-02 |
| btg2 | NM_007570 | 0.44** | 0.29** | 1.68* | 0.99 |  | 0.79 | 0.96 | 0.76 | 1.14 | 2.66E-02 |
| c1qtnf6 | AK012868 | 0.69* | 0.92 | 0.73 | 0.91 |  | 0.63* | 3.05** | 0.54** | 5.83** | 6.04E-04 |
| c1qtnf7 | BB039211 | 0.92 | 1.07 | 0.99 | 1.02 |  | 0.59** | 0.30** | 0.65** | 0.51** | 8.64E-03 |
| camk1d | BG071931 | 2.75** | 2.12** | 0.79 | 1.29 |  | 0.95 | 0.87 | 0.85 | 0.82 | 1.01E-02 |
| card6 | BB766747 | 0.42** | 0.54** | 0.75* | 0.50** |  | 0.67** | 0.75** | 1.22 | 0.89 | 6.13E-03 |
| cd209b | AF374471 | 0.86 | 0.74* | 0.73** | 1.30* |  | 1.09 | 1.95** | 0.33** | 1.18 | 7.49E-03 |
| cd3g | M58149 | 1.11 | 1.08 | 0.94 | 0.9 |  | 1.33 | 1.99** | 1.03 | 0.82 | 3.59E-02 |
| cd40 | BB220422 | 2.76** | 1.37 | 1.99** | 1.02 |  | 0.55** | 1.18 | 0.83 | 2.13** | 1.37E-02 |
| cdc2a | NM_007659 | 0.95 | 2.91** | 11.93** | 3.35** |  | 0.51* | 1.17 | 0.89 | 1.12 | 2.52E-02 |
| cdkn1a | AK007630 | 1.57** | 2.05** | 1.71** | 1.24 |  | 0.96 | 1.44* | 0.99 | 0.9 | 5.01E-03 |
| cdkn2a | NM_009877 | 2.17** | 1.79** | 1.57** | 2.61** |  | 1.21 | 1.07 | 1.07 | 0.87 | 8.43E-03 |
| ciapin1 | NM_134141 | 1.08 | 0.68* | 2.75** | 1.01 |  | 0.97 | 2.58** | 1.07 | 2.30** | 1.69E-02 |
| cse1l | NM_023565 | 1.62** | 2.14** | 3.21** | 1.82** |  | 0.83* | 1.07 | 1.18 | 1.23* | 6.88E-03 |
| ctnna1 | NM_009818 | 0.47** | 0.56** | 1.52** | 0.9 |  | 0.78* | 0.84 | 0.88 | 1.04 | 3.73E-02 |
| cugbp2 | BB644164 | 1.40** | 2.26** | 1.19 | 1.02 |  | 0.94 | 1.05 | 0.99 | 0.88 | 1.06E-02 |
| cycs | NM_007808 | 1.17* | 1.98** | 1.55** | 2.05** |  | 0.83* | 1 | 1.17 | 1.05 | 1.64E-03 |
| dap | BC024876 | 0.77* | 0.48** | 1.14 | 0.72* |  | 0.81 | 0.84 | 0.78 | 1.13 | 4.10E-03 |
| dlg5 | BC021314 | 0.70* | 0.44** | 0.62** | 0.89 |  | 0.8 | 0.64** | 0.88 | 0.82 | 3.39E-03 |
| dnaja3 | AK004575 | 1.33 | 0.98 | 0.91 | 0.94 |  | 1.12 | 2.65** | 1.2 | 2.13** | 4.24E-02 |
| eef1e1 | NM_025380 | 3.22** | 2.14** | 7.58** | 7.88** |  | 1.4 | 3.42** | 1.52* | 1.63** | 4.05E-02 |
| efhc1 | AK006489 | 0.58** | 0.41** | 0.97 | 0.63** |  | 1.01 | 0.77* | 0.95 | 0.68** | 1.06E-03 |
| eif5a | BF384094 | 1.38** | 1.44** | 1.75** | 1.46* |  | 1.42* | 2.53** | 1.36* | 1.51** | 5.95E-03 |
| elmo3 | AI481208 | 1.16 | 0.28** | 0.77 | 0.47** |  | 0.53** | 0.82 | 0.68* | 0.82 | 2.12E-02 |
| endog | NM_007931 | 2.49** | 2.90** | 2.40** | 1.96** |  | 0.75* | 1.46** | 1.2 | 1.74** | 2.48E-02 |
| eya1 | BB760085 | 0.71* | 0.40** | 1.64** | 1.08 |  | 1.63** | 0.58** | 1.22 | 0.92 | 1.71E-02 |
| fas | BG976607 | 6.55** | 6.15** | 3.85** | 11.34** |  | 0.82 | 1.22 | 1.61 | 1.35 | 3.79E-02 |
| fas | BG976607 | 3.61** | 7.00** | 1.13 | 4.43** |  | 1.33 | 0.63 | 1.35 | 0.46** | 6.32E-03 |
| fas | BG976607 | 5.08** | 12.85** | 5.07** | 10.92** |  | 0.72 | 5.14** | 0.74 | 0.48* | 1.12E-03 |
| fas | BG976607 | 2.43** | 1.78** | 2.28** | 2.50** |  | 0.83 | 1.33 | 1.09 | 0.39** | 2.21E-03 |
| fastkd1 | BE957020 | 2.47** | 1.13 | 1.81** | 1.50* |  | 1.04 | 0.88 | 1.11 | 1.07 | 1.11E-02 |
| fcer1g | NM_010185 | 1.05 | 1.42* | 1.76* | 1.59* |  | 0.89 | 3.51** | 0.9 | 4.30** | 1.86E-02 |
| gadd45b | AK010420 | 0.70* | 0.35** | 2.02** | 0.48** |  | 1.44* | 1.67* | 1.27 | 0.53** | 5.57E-03 |
| gadd45g | AK007410 | 0.59* | 0.41** | 1.88** | 0.59** |  | 4.07** | 5.16** | 3.39** | 2.78** | 5.95E-03 |
| gas1 | BB550400 | 0.88 | 0.89 | 0.47** | 0.94 |  | 1.08 | 0.41** | 0.93 | 0.39** | 2.37E-02 |
| glo1 | BC024663 | 0.74 | 0.76 | 1.23 | 0.81 |  | 1 | 0.38** | 1.19 | 1.11 | 3.94E-02 |
| glo1 | BC024663 | 0.72 | 0.64** | 1.24 | 0.98 |  | 1.15 | 0.34** | 1.23 | 0.75 | 2.63E-02 |
| gsk3b | BB831420 | 0.88 | 0.45** | 0.63** | 0.57** |  | 1.69** | 0.69** | 0.97 | 0.8 | 3.86E-02 |
| hells | NM_008234 | 1.93* | 11.54** | 17.37** | 10.68** |  | 0.38** | 0.57 | 1.24 | 1.33 | 1.59E-02 |
| hells | AK021390 | 1.19 | 5.87** | 2.77** | 3.39** |  | 0.65 | 0.9 | 1.29 | 0.83 | 4.92E-02 |
| hras1 | NM_008284 | 1.16 | 1.16 | 3.06** | 2.60** |  | 1.14 | 2.89** | 0.61* | 1.35 | 7.09E-03 |
| hras1 | BC011083 | 1.43 | 1.43 | 3.24** | 2.40** |  | 1.03 | 2.15** | 0.7 | 1.34 | 1.14E-02 |
| hspa5 | AJ002387 | 1.03 | 1.43* | 1.27 | 1.22 |  | 1.14 | 2.68** | 0.89 | 2.27** | 8.20E-03 |
| htatip2 | AF061972 | 0.84 | 0.40** | 0.74** | 0.64** |  | 0.82 | 0.92 | 0.92 | 1.38** | 1.66E-02 |
| htra2 | AW323050 | 1.39* | 2.20** | 1.15 | 1.49* |  | 0.84 | 1.56** | 0.59** | 1.57** | 3.59E-02 |
| igf1 | NM_010512 | 2.08* | 0.63 | 0.74 | 1.2 |  | 1.17 | 2.33** | 1.47 | 2.00* | 4.75E-02 |
| igf1 | AF440694 | 1.05 | 1 | 1.59 | 3.71** |  | 1.56* | 2.24** | 1.24 | 3.08** | 4.66E-02 |
| ikbkg | BB821318 | 1.65** | 0.50** | 0.73* | 2.90** |  | 1.1 | 0.74** | 0.9 | 1.40** | 1.74E-02 |
| il1a | BC003727 | 1.06 | 1 | 1.06 | 1.08 |  | 0.46** | 0.78 | 0.44** | 0.31** | 1.60E-02 |
| il1r1 | NM_008362 | 0.53** | 0.41** | 1.56* | 0.62* |  | 0.57** | 1.29 | 1.02 | 1.33 | 5.95E-03 |
| il1rap | BE285634 | 0.9 | 0.66** | 1.15 | 1.13 |  | 0.82 | 0.41** | 0.91 | 0.41** | 9.62E-03 |
| inhba | NM_008380 | 0.58** | 0.38** | 0.96 | 0.60** |  | 0.94 | 1.12 | 0.85 | 1.31* | 1.31E-02 |
| irf6 | NM_016851 | 0.62* | 0.42** | 1.2 | 0.73 |  | 0.50** | 0.76 | 0.78 | 0.82 | 1.99E-02 |
| jag2 | AV264681 | 2.77** | 2.36* | 2.36** | 4.13** |  | 1.24 | 0.74 | 0.99 | 1.85* | 1.33E-02 |
| jag2 | AV264681 | 2.43** | 1.32 | 1.16 | 2.02** |  | 0.69 | 1.33 | 1.03 | 0.9 | 3.18E-02 |
| jmjd6 | AK017622 | 0.8 | 1.13 | 1.07 | 2.95** |  | 1.1 | 2.32** | 1.04 | 2.11** | 6.93E-03 |
| kitl | BB815530 | 0.8 | 0.97 | 1.05 | 1 |  | 1.08 | 0.43** | 1.19 | 0.69** | 1.08E-02 |
| kitl | BB815530 | 0.67* | 0.36** | 0.34** | 0.43** |  | 1 | 0.29** | 1.1 | 1.02 | 9.96E-03 |
| litaf | AV360881 | 2.55** | 0.83 | 1.71** | 1.67** |  | 1.72** | 1.08 | 1.56* | 1.04 | 3.79E-03 |
| lsp1 | NM_019391 | 0.9 | 0.78* | 0.97 | 1.1 |  | 1.02 | 3.42** | 0.67** | 1.90** | 3.22E-02 |
| mapk10 | BB453775 | 0.46** | 0.43** | 1.13 | 0.87 |  | 0.92 | 0.77** | 1.13 | 0.68** | 4.76E-03 |
| mapk8ip1 | BB546463 | 0.62** | 0.35** | 0.74 | 0.53** |  | 0.89 | 1.14 | 0.83 | 1.06 | 1.56E-02 |
| mapk8ip2 | AW536912 | 0.47** | 0.93 | 1.60* | 1.49* |  | 0.88 | 0.96 | 1.09 | 0.94 | 2.29E-02 |
| mitf | BB763517 | 0.49* | 0.34** | 0.82 | 0.91 |  | 0.88 | 0.79 | 0.95 | 0.59 | 1.72E-02 |
| myc | BC006728 | 1.1 | 0.99 | 1.12 | 1.02 |  | 0.32** | 0.65* | 0.53** | 0.99 | 1.97E-02 |
| nek6 | BB528391 | 1.42 | 1.59* | 1.61* | 2.92** |  | 0.89 | 2.08** | 1.22 | 2.15** | 2.03E-03 |
| nfkbia | BB096843 | 0.62** | 0.43** | 1.42* | 0.82 |  | 0.61* | 1.11 | 0.96 | 0.99 | 4.03E-02 |
| nod1 | BB138330 | 0.97 | 0.29** | 1.26 | 1.25 |  | 0.61* | 0.79 | 0.46** | 1.39 | 4.61E-02 |
| nuak2 | AK004737 | 0.50** | 1.02 | 1.88** | 0.64** |  | 0.71* | 1.37* | 0.82 | 1.48** | 2.45E-02 |
| nup62 | NM_053074 | 1.83** | 2.56** | 4.57** | 2.56** |  | 0.77** | 0.88 | 1.05 | 1.27** | 8.31E-04 |
| nup62 | AW240611 | 2.10** | 2.29** | 2.91** | 2.73** |  | 0.78 | 0.83 | 1.11 | 1.17 | 3.61E-03 |
| parp1 | BB767586 | 2.36** | 2.22** | 0.97 | 1.95** |  | 0.99 | 0.89 | 1.50** | 0.84* | 8.08E-05 |
| pdcd11 | AK003899 | 3.54** | 1.52* | 2.21** | 3.35** |  | 1.49* | 1.07 | 1.63* | 1.14 | 2.26E-04 |
| pdcd2l | AK003339 | 1.07 | 0.59* | 2.56** | 1.91** |  | 0.8 | 2.71** | 0.78 | 1.92** | 4.33E-02 |
| pdcd2l | AK003339 | 1.14 | 1.04 | 1.75** | 2.07** |  | 0.96 | 2.09** | 0.67** | 1.58** | 9.26E-03 |
| peg3 | AB003040 | 0.47** | 0.24** | 0.75* | 0.52** |  | 1.23 | 1.02 | 1 | 0.71* | 4.63E-02 |
| phlda1 | NM_009344 | 0.50** | 0.99 | 1.51* | 0.67* |  | 0.65* | 0.87 | 0.97 | 1.04 | 3.01E-02 |
| pigt | AK019717 | 1.18 | 0.53** | 1.50** | 1.31 |  | 1.03 | 1.96** | 0.93 | 1.3 | 1.15E-02 |
| pik3ca | AI528567 | 0.78* | 0.49** | 1.52** | 0.89 |  | 0.77* | 0.71** | 0.81 | 1.02 | 2.48E-03 |
| pmaip1 | NM_021451 | 1.15 | 1.13 | 1.02 | 0.85 |  | 1.33* | 0.45** | 0.77* | 0.87 | 6.77E-03 |
| ppp1r13b | BG064715 | 0.64* | 0.62** | 0.81 | 0.73 |  | 0.70* | 0.47** | 0.8 | 1.01 | 5.34E-03 |
| prkar1b | NM_008923 | 0.61** | 0.34** | 2.19** | 1.07 |  | 1.53** | 0.84 | 1.47** | 0.64** | 1.71E-02 |
| prkar2a | AK004336 | 2.52** | 2.59** | 1.72** | 2.36** |  | 1.15 | 0.75 | 1.19 | 0.84 | 4.12E-02 |
| prkar2a | AK004336 | 2.16** | 2.49** | 1.29* | 3.03** |  | 0.99 | 0.79 | 1.52 | 0.83 | 3.47E-03 |
| prkar2b | BB216074 | 1.29 | 0.85 | 1.76** | 1.72** |  | 0.48** | 1.15 | 0.84 | 1.48* | 9.51E-03 |
| ptprv | NM_007955 | 0.97 | 0.97 | 1.08 | 0.96 |  | 0.51** | 1.26 | 0.92 | 1.98** | 3.62E-02 |
| ptrh2 | BC026947 | 1.57 | 0.77 | 2.71** | 3.80** |  | 1.05 | 2.12** | 0.78 | 1.34 | 3.55E-02 |
| ptrh2 | BB178232 | 2.11** | 2.07* | 0.63 | 1.53 |  | 1.07 | 1.18 | 1 | 1.83* | 2.37E-02 |
| pycard | BG084230 | 1.33* | 0.64* | 4.64** | 2.06** |  | 0.48** | 1.24 | 0.78 | 1.42* | 4.71E-02 |
| rffl | AW123157 | 1.62* | 0.55** | 0.48** | 0.68* |  | 0.89 | 0.45** | 0.93 | 0.57** | 1.15E-02 |
| ripk2 | NM_138952 | 2.07** | 0.81 | 1.13 | 2.48** |  | 1.21 | 1.09 | 0.85 | 1.03 | 7.43E-03 |
| rtn4 | BE988775 | 0.67 | 0.49** | 0.61* | 0.64* |  | 1.09 | 0.56* | 1.09 | 0.60* | 1.42E-03 |
| rtn4 | AK003859 | 0.57** | 0.43** | 1.24* | 0.60** |  | 0.98 | 0.98 | 1 | 1.06 | 1.75E-02 |
| scin | NM_009132 | 1.21 | 0.91 | 0.60** | 1.14 |  | 1.07 | 0.51** | 1.08 | 0.63** | 7.55E-03 |
| serinc3 | BM244064 | 0.66** | 0.49** | 1.15 | 0.76** |  | 1.02 | 1.06 | 0.77* | 1.06 | 2.25E-03 |
| serinc3 | BM239368 | 0.69* | 0.91 | 0.94 | 0.95 |  | 0.95 | 0.45** | 0.65* | 0.9 | 3.83E-02 |
| sgk | NM_011361 | 0.88 | 0.48** | 1.21 | 1.09 |  | 0.92 | 0.65* | 0.88 | 0.75 | 1.11E-02 |
| sgpl1 | NM_009163 | 1 | 0.72** | 1.40** | 0.79** |  | 0.76** | 0.46** | 0.72** | 1.30** | 4.84E-02 |
| sh3kbp1 | BB326929 | 0.63* | 0.48** | 0.47** | 1 |  | 0.84 | 0.71 | 1 | 0.68* | 1.93E-02 |
| siah2 | AA414485 | 0.47** | 0.64** | 1.37** | 0.81 |  | 0.56** | 1.65** | 0.63** | 2.85** | 1.70E-02 |
| siva1 | NM_013929 | 1.45* | 0.99 | 4.89** | 1.85** |  | 1.03 | 2.18** | 0.85 | 1.46* | 1.03E-02 |
| siva1 | AF033112 | 1.23 | 2.23** | 3.10** | 1.38 |  | 0.82 | 1.99** | 0.79 | 1.33 | 2.07E-02 |
| siva1 | AF033112 | 1.26 | 1.97** | 3.56** | 1.89** |  | 0.99 | 2.23** | 1.21 | 1.36 | 6.50E-03 |
| sphk2 | AK016616 | 1.08 | 0.56** | 0.94 | 0.66** |  | 0.42** | 0.59** | 0.91 | 0.70* | 1.84E-02 |
| sqstm1 | BM232298 | 0.44** | 0.28** | 2.68** | 0.57* |  | 1.93* | 2.25** | 0.79 | 0.91 | 3.53E-02 |
| stambp | AA289490 | 0.74** | 0.34** | 0.55** | 0.71** |  | 1.37** | 1.07 | 1.30** | 0.89 | 2.35E-02 |
| stat5a | U36502 | 0.56** | 0.40** | 0.82 | 1.01 |  | 0.71* | 0.69* | 0.91 | 0.9 | 3.12E-03 |
| tbrg4 | BB139935 | 2.00** | 1.59* | 1.28* | 2.23** |  | 1.12 | 0.9 | 1.68* | 0.96 | 1.84E-02 |
| tfdp1 | BG075396 | 3.08** | 1.70** | 3.28** | 3.13** |  | 1.11 | 1.43* | 1.52* | 1.27 | 9.57E-03 |
| thoc1 | BC024951 | 2.03** | 0.88 | 1.34* | 1.56** |  | 0.81 | 0.66** | 1.28 | 1 | 3.15E-02 |
| thoc1 | BC024951 | 2.02** | 0.84 | 1.64** | 1.19 |  | 0.92 | 0.72 | 1.32 | 1.06 | 4.45E-02 |
| thoc1 | BG066490 | 2.12** | 3.14** | 1.36* | 2.16** |  | 1.02 | 1.11 | 1.39 | 1 | 1.30E-03 |
| tial1 | NM_009383 | 1.06 | 0.97 | 1.97** | 0.76* |  | 0.71** | 2.25** | 0.88 | 2.52** | 1.68E-03 |
| tlr1 | AF316985 | 0.94 | 1.01 | 0.73* | 1.32 |  | 2.12** | 3.71** | 1.69** | 4.60** | 7.66E-03 |
| tm2d1 | AF353993 | 1.33 | 1.37 | 1.77** | 1.29 |  | 0.44** | 0.99 | 0.9 | 1.75** | 2.36E-02 |
| tnfrsf12a | NM_013749 | 0.59** | 0.85 | 2.53** | 0.72** |  | 1.11 | 3.15** | 1.38** | 1.17 | 4.16E-02 |
| tnfrsf12a | NM_013749 | 0.86 | 0.87 | 2.29** | 0.85 |  | 1.18 | 2.77** | 1.39** | 1.11 | 4.89E-03 |
| tnfrsf22 | BB366863 | 0.37** | 0.70* | 1.58* | 0.86 |  | 0.73* | 0.94 | 1.06 | 1.81** | 3.76E-02 |
| tnfrsf4 | NM_011659 | 1.18 | 0.60* | 1.46* | 1.64** |  | 1.27 | 2.12** | 1.76** | 1.03 | 5.78E-03 |
| tnfsf13 | NM_023517 | 0.33** | 0.99 | 1.57 | 1.81 |  | 0.87 | 1.98* | 0.38** | 1.34 | 4.42E-02 |
| tnfsf5ip1 | BC016606 | 1.16 | 0.92 | 3.22** | 2.64** |  | 0.98 | 3.12** | 1.03 | 1.99** | 1.95E-03 |
| tnfsf5ip1 | NM_134138 | 2.23** | 1.34 | 2.43** | 5.11** |  | 1.21 | 1.42 | 1.25 | 1.15 | 5.23E-03 |
| tnfsf9 | NM_009404 | 1.02 | 1.04 | 2.76** | 1.28 |  | 0.92 | 2.05** | 1.06 | 0.81 | 1.21E-02 |
| tns4 | BB142697 | 0.97 | 0.98 | 1.14 | 1 |  | 0.68* | 0.42** | 0.89 | 0.82 | 5.47E-03 |
| tradd | BB749262 | 0.96 | 0.55* | 1.57* | 1.70* |  | 0.81 | 3.29** | 0.71 | 1.33 | 5.90E-03 |
| traf6 | AV244412 | 0.76* | 0.33** | 0.99 | 1.04 |  | 1.11 | 1.12 | 1.16 | 0.97 | 2.96E-02 |
| triap1 | AK007514 | 1.69** | 1.11 | 1.88** | 1.65* |  | 1.01 | 3.43** | 0.88 | 1.99** | 6.53E-04 |
| trib3 | BB508622 | 0.56* | 0.58* | 3.35** | 0.69 |  | 0.94 | 0.48** | 0.76 | 0.59* | 1.63E-02 |
| trp53bp2 | BB814564 | 0.86 | 0.41** | 1.50* | 0.98 |  | 0.61** | 0.79 | 0.81 | 0.92 | 3.77E-02 |
| tsc22d3 | NM_010286 | 0.8 | 0.75 | 1.32 | 0.65** |  | 1.31 | 2.38** | 0.47** | 0.96 | 1.28E-02 |
| usp14 | AW107924 | 1.89** | 2.20** | 0.87 | 0.98 |  | 0.95 | 1 | 1.12 | 0.94 | 2.85E-03 |
| usp14 | AW107924 | 1.99** | 3.23** | 0.40** | 0.75 |  | 0.69 | 0.69 | 1.29 | 1.3 | 2.25E-02 |
| zbtb16 | Z47205 | 0.96 | 0.67* | 0.79 | 0.61* |  | 1.83** | 0.51** | 0.87 | 0.43** | 3.11E-02 |

**Table S5: Differentiation-related genes showing significant change in expression within 8 hours of MYC-ER^TAM^ activation.**

Genes relating to differentiation showing a significant change in expression within 8 hours following activation of MYC-ER^TAM^. ‘MYC-response p-value’ is the p-value identified for the highest-order interaction of the MYC activation variable and represents the significance of this term within the selected model. Flags represent contrast p-values comparing 4OHT-treated and vehicle-treated samples at specific time points (‘*’, p ≤ 0.05; ‘**’ p ≤ 0.01). Cells are colour-coded based on a detected fold-change greater than 1.5-fold (red, up-regulated; blue, down-regulated).

|  |  | **Pancreatic β-cells** | | | |  | **Suprabasal Keratinocytes** | | | | **Myc-response p-value** |
| --- | --- | --- | --- | --- | --- | --- | --- | --- | --- | --- | --- |
| **Gene Symbol** | **RefSeq** | **4 hrs** | **8 hrs** | **16 hrs** | **32 hrs** |  | **4 hrs** | **8 hrs** | **16 hrs** | **32 hrs** |
| 4732435n03rik | AV371987 | 0.64* | 0.77 | 0.25** | 0.96 |  | 0.79 | 0.44** | 0.46** | 0.74 | 1.57E-02 |
| 8030451f13rik | AV171553 | 1.46** | 1.14 | 1.15 | 0.87 |  | 1.21 | 2.00** | 1.14 | 1.06 | 4.96E-02 |
| abcb1b | NM_011075 | 2.24** | 1.40* | 0.97 | 3.64** |  | 0.8 | 0.58** | 0.88 | 0.96 | 2.46E-02 |
| abi2 | BB051811 | 2.05** | 2.14** | 0.8 | 0.86 |  | 1.21 | 0.67 | 1.46 | 0.84 | 1.97E-03 |
| adcyap1r1 | AK013587 | 0.85 | 0.86 | 0.86 | 1.71** |  | 0.83 | 0.31** | 0.76* | 0.49** | 6.59E-04 |
| aff4 | BM230280 | 0.75* | 0.58** | 0.75* | 1.32* |  | 1.03 | 0.44** | 1.18 | 0.85 | 2.41E-02 |
| agt | AK018763 | 0.98 | 0.28** | 1.64** | 2.21** |  | 1.17 | 1.06 | 1.14 | 2.08** | 4.69E-02 |
| ahctf1 | BC023122 | 2.23** | 1.28* | 1.34** | 2.77** |  | 0.94 | 0.67** | 1.36** | 0.71** | 1.13E-02 |
| ahctf1 | BC023122 | 2.78** | 1.12 | 1.94** | 2.40** |  | 0.88 | 0.79 | 1.19 | 1.46 | 9.32E-03 |
| ai467657 | AA419994 | 0.57** | 1.57* | 1.29 | 0.51** |  | 0.86 | 0.33** | 0.99 | 0.45** | 1.30E-02 |
| akt1 | NM_009652 | 1.01 | 0.97 | 1.71** | 2.31** |  | 0.82 | 3.23** | 0.95 | 2.29** | 4.43E-02 |
| alas2 | M63244 | 0.88 | 0.96 | 1.18 | 0.8 |  | 1.33 | 2.18** | 0.96 | 1.37* | 1.02E-02 |
| amd1 | NM_009665 | 2.23** | 1.51** | 1.60** | 2.15** |  | 1.09 | 1.11 | 0.92 | 0.99 | 1.46E-04 |
| amn | NM_033603 | 6.51** | 1.69 | 1.38 | 10.39** |  | 1.37 | 2.43* | 2.91* | 2.51* | 5.19E-04 |
| angpt1 | BB453314 | 1.04 | 1.06 | 1.06 | 1.13 |  | 0.40** | 0.50** | 0.44** | 1.22 | 2.13E-03 |
| apaf1 | AK018076 | 0.54 | 1.06 | 1.04 | 1.12 |  | 0.83 | 0.65 | 0.54* | 1.04 | 2.05E-02 |
| ar | BB148302 | 0.48** | 0.75 | 0.95 | 0.86 |  | 1.04 | 0.58* | 0.97 | 0.68 | 2.83E-03 |
| arx | BB322201 | 0.49** | 0.59** | 0.40** | 0.55** |  | 0.99 | 0.63** | 0.83 | 1.36* | 4.44E-04 |
| atp7a | U03434 | 0.36** | 0.86 | 0.82 | 0.31** |  | 0.51** | 0.46** | 1.45* | 0.66* | 4.62E-02 |
| atrnl1 | AW555641 | 0.98 | 1.02 | 0.64* | 0.81 |  | 1.08 | 0.50** | 0.99 | 0.43** | 1.92E-02 |
| atrx | BB425841 | 1.57** | 1.98** | 1.55** | 1.78** |  | 0.9 | 0.93 | 1.27 | 1.15 | 3.95E-02 |
| bag1 | NM_009736 | 1.38 | 1.46* | 3.04** | 2.00** |  | 0.72 | 2.28** | 1.03 | 1.95** | 3.80E-03 |
| bbs1 | BB121315 | 0.26** | 0.34** | 1.41 | 0.67* |  | 0.67* | 0.52** | 0.98 | 1.01 | 3.64E-02 |
| bcl11a | NM_016707 | 1.18 | 0.45** | 1.15 | 1.24 |  | 0.99 | 0.49** | 0.86 | 0.53** | 1.87E-03 |
| bcl11a | BB424718 | 1.27 | 0.48* | 0.39* | 0.6 |  | 0.86 | 0.42** | 0.91 | 0.44* | 4.50E-02 |
| bin1 | U60884 | 1 | 0.46** | 2.08** | 1.05 |  | 1.04 | 3.12** | 1.05 | 2.84** | 1.92E-02 |
| bin1 | BG293813 | 0.99 | 0.32** | 0.40** | 0.51** |  | 1.06 | 0.84 | 1.02 | 1.11 | 3.76E-02 |
| bmp1 | L24755 | 0.53** | 0.48** | 1.33 | 0.75 |  | 0.70* | 1.55* | 0.56** | 1.97** | 1.26E-02 |
| bmp2 | AV239587 | 1.1 | 1.39 | 0.64* | 1.11 |  | 0.47** | 0.39** | 0.80* | 0.38** | 8.19E-03 |
| bmp4 | NM_007554 | 0.79 | 0.92 | 1.18 | 1.17 |  | 0.38** | 0.76 | 0.50** | 0.69* | 2.66E-02 |
| btg1 | L16846 | 0.47** | 0.35** | 1.14 | 0.56** |  | 0.65* | 0.79 | 0.75 | 0.97 | 2.21E-03 |
| btg1 | AW322026 | 0.61** | 0.40** | 0.93 | 0.73** |  | 0.78* | 0.71** | 0.82 | 0.76* | 2.36E-03 |
| btg2 | NM_007570 | 0.29** | 0.32** | 1.17 | 0.7 |  | 1.72 | 1.96* | 0.88 | 0.68 | 1.57E-02 |
| btg2 | NM_007570 | 0.44** | 0.29** | 1.68* | 0.99 |  | 0.79 | 0.96 | 0.76 | 1.14 | 2.66E-02 |
| bzw2 | BM932775 | 4.06** | 2.16** | 4.14** | 4.97** |  | 0.95 | 2.29** | 1.32 | 1.89** | 2.45E-02 |
| camk1 | NM_133926 | 0.95 | 0.88 | 1.44 | 0.8 |  | 0.82 | 4.62** | 0.69 | 5.19** | 1.10E-02 |
| cbfa2t3h | BB471309 | 0.87 | 0.42** | 0.39** | 0.58** |  | 1.09 | 0.76* | 1.1 | 1.14 | 1.44E-02 |
| ccl21a | NM_011335 | 0.98 | 1.17 | 0.94 | 1.78** |  | 1.26 | 3.03** | 0.65** | 1.68** | 2.83E-02 |
| ccnd1 | NM_007631 | 1.86** | 2.09** | 0.88 | 1.45 |  | 1.13 | 1.31 | 1.79* | 1.04 | 3.73E-02 |
| ccnd1 | NM_007631 | 1.69 | 2.38 | 1.41 | 2.27* |  | 1.05 | 1.33 | 1.72 | 1.26 | 4.55E-02 |
| ccnd1 | NM_007631 | 3.41** | 2.74* | 2.03* | 2.05 |  | 1.35 | 0.99 | 1.54 | 1.81 | 2.24E-02 |
| ccndbp1 | NM_010761 | 0.42* | 0.14** | 2.54* | 2.56** |  | 1.04 | 2.37* | 0.63 | 0.93 | 1.86E-02 |
| ccr1 | AV231648 | 0.9 | 0.92 | 0.97 | 1.01 |  | 1.19 | 2.42** | 0.89 | 2.35** | 3.58E-02 |
| cdkn1b | NM_009875 | 1.03 | 0.19** | 0.71 | 0.95 |  | 0.23** | 0.42* | 0.48* | 0.89 | 1.02E-02 |
| cdkn2a | NM_009877 | 2.17** | 1.79** | 1.57** | 2.61** |  | 1.21 | 1.07 | 1.07 | 0.87 | 8.43E-03 |
| chm | NM_018818 | 0.49** | 0.59** | 1.43* | 0.60** |  | 0.63** | 0.69** | 1.12 | 0.88 | 4.91E-02 |
| cryab | NM_009964 | 1.06 | 1.13 | 1.08 | 1.47 |  | 0.96 | 4.03** | 1.09 | 1.80** | 8.20E-03 |
| csf1 | BM233698 | 1.17 | 0.95 | 0.70* | 1.26 |  | 1.81** | 2.63** | 1.79** | 3.72** | 5.68E-04 |
| cspg5 | NM_013884 | 2.23** | 0.81 | 1.24 | 1.81* |  | 1.01 | 0.89 | 1.26 | 0.89 | 2.00E-02 |
| ctgf | NM_010217 | 0.54* | 1.15 | 0.62 | 1.22 |  | 1.26 | 0.55* | 0.84 | 0.68 | 3.37E-03 |
| ctnnb1 | BI134907 | 1.11 | 1.04 | 1.01 | 0.92 |  | 1.22 | 2.36** | 1.04 | 1.54** | 3.46E-02 |
| cutl1 | BC014289 | 1.04 | 1.07 | 1.18* | 0.92 |  | 0.64** | 0.49** | 1.01 | 0.84** | 2.42E-02 |
| dab2 | NM_023118 | 0.88 | 0.48* | 4.13** | 2.64** |  | 1.74* | 3.75** | 1.31 | 3.85** | 3.79E-03 |
| dab2 | BC006588 | 0.8 | 0.81 | 1.53 | 1.23 |  | 1.27 | 2.75** | 0.82 | 2.22** | 6.40E-03 |
| dab2 | AK017619 | 1.31 | 0.76 | 0.96 | 1.16 |  | 1.41 | 3.55** | 0.58* | 2.62** | 2.93E-02 |
| dach1 | BB374930 | 1.85** | 0.24** | 0.36** | 0.77 |  | 0.84 | 0.43** | 0.68* | 0.62** | 8.16E-04 |
| dach2 | NM_033605 | 0.39** | 1.94** | 2.64** | 0.88 |  | 1 | 1.01 | 1.25 | 0.73* | 3.53E-03 |
| ddef1 | BG064109 | 1.01 | 1.04 | 1.03 | 1.04 |  | 1.2 | 2.13** | 1.48** | 1.05 | 4.58E-02 |
| dep1 | BB088198 | 1.14 | 1.1 | 0.79 | 0.95 |  | 0.92 | 0.44** | 1.18 | 1.08 | 2.94E-02 |
| dhcr7 | NM_007856 | 2.28** | 2.57** | 1.26* | 1.54** |  | 0.50** | 0.49** | 0.83 | 1.39* | 2.52E-02 |
| dip2a | BB794700 | 0.56** | 0.30** | 0.81 | 0.49** |  | 1.05 | 0.74 | 1.03 | 0.63** | 6.53E-04 |
| dmd | NM_007868 | 1.29* | 1.01 | 1.11 | 1.98** |  | 0.83 | 0.46** | 0.87 | 0.92 | 2.76E-02 |
| dmrt2 | BC027669 | 1 | 1.03 | 1.06 | 1.07 |  | 1.06 | 2.64** | 0.58** | 1.46* | 3.62E-06 |
| dner | AF370126 | 0.67* | 0.27** | 0.25** | 0.78 |  | 1.2 | 1.01 | 0.95 | 0.93 | 4.26E-02 |
| drg1 | BM506525 | 1.54** | 2.23** | 1.21 | 1.35** |  | 1.02 | 1.44** | 1.13 | 0.91 | 1.18E-02 |
| drg1 | AV127994 | 1.80** | 2.27** | 1.42** | 1.53** |  | 0.73* | 1.18 | 1.46** | 0.99 | 2.23E-04 |
| drg1 | NM_007879 | 1.18 | 1.37* | 4.20** | 2.41** |  | 0.82 | 2.50** | 0.94 | 1.78** | 2.45E-02 |
| drg2 | NM_021354 | 1.76** | 1.31 | 1.89** | 2.19** |  | 0.83 | 2.28** | 1.12 | 1.25 | 4.32E-02 |
| dzip1 | AI509011 | 0.49** | 0.79 | 0.89 | 0.98 |  | 0.89 | 0.98 | 0.77 | 1.35* | 2.60E-02 |
| ebf1 | BB364548 | 1 | 0.99 | 0.99 | 1.05 |  | 1.5 | 2.61** | 0.86 | 1.99** | 4.00E-02 |
| ebf1 | BB038386 | 1.03 | 1.01 | 0.49** | 1.50* |  | 1.63** | 2.40** | 0.74 | 2.80** | 7.19E-03 |
| ebf3 | AK014058 | 0.96 | 1.03 | 0.76* | 1.1 |  | 0.81 | 0.48** | 0.93 | 0.85 | 1.81E-02 |
| ebf3 | AK014058 | 0.93 | 0.88 | 1.21 | 0.97 |  | 0.54** | 0.45** | 1.02 | 1.36 | 2.19E-02 |
| edg2 | U70622 | 0.54 | 0.30** | 0.31* | 0.65 |  | 1.26 | 0.46 | 0.75 | 0.59 | 3.33E-02 |
| ednra | AW558570 | 1.05 | 0.79* | 0.75* | 1.38** |  | 0.93 | 0.43** | 0.81* | 0.46** | 2.04E-02 |
| ednrb | BB451714 | 0.64* | 0.47** | 1.14 | 0.95 |  | 1.4 | 4.30** | 1.13 | 3.05** | 2.54E-02 |
| ednrb | BF100813 | 1.07 | 0.49** | 1.51 | 1.11 |  | 1.17 | 1 | 1.53* | 2.52** | 1.29E-02 |
| eef1e1 | NM_025380 | 3.22** | 2.14** | 7.58** | 7.88** |  | 1.4 | 3.42** | 1.52* | 1.63** | 4.05E-02 |
| efhc1 | AK006489 | 0.58** | 0.41** | 0.97 | 0.63** |  | 1.01 | 0.77* | 0.95 | 0.68** | 1.06E-03 |
| efhd2 | AK007560 | 0.76 | 0.44** | 2.51** | 2.03** |  | 0.60* | 1.68* | 1.34 | 5.72** | 8.01E-03 |
| efnb1 | NM_010110 | 0.59** | 0.70** | 0.62* | 0.52** |  | 0.40** | 0.50** | 0.98 | 1.08 | 2.07E-02 |
| egr3 | AV346607 | 1.06 | 1.24 | 2.20** | 1.15 |  | 0.68** | 0.39** | 0.84 | 0.40** | 2.32E-02 |
| ehf | BC008249 | 1.06 | 0.94 | 0.83 | 1.29* |  | 0.55** | 0.49** | 0.80* | 0.70** | 1.84E-02 |
| ehf | BC006789 | 0.73 | 1.29 | 1.06 | 1.13 |  | 0.68 | 0.36** | 0.82 | 0.76 | 2.82E-02 |
| elf5 | BC012424 | 1 | 0.97 | 1.2 | 1.06 |  | 0.50** | 0.19** | 0.65* | 1.35 | 4.14E-03 |
| ensmusg00000075401 | BQ175722 | 1.16 | 0.70* | 0.75 | 0.99 |  | 0.95 | 0.42** | 1.08 | 0.58** | 2.52E-02 |
| erbb3 | BF140685 | 0.45** | 0.51** | 0.59* | 1.5 |  | 0.87 | 0.44** | 0.7 | 0.43** | 1.48E-02 |
| ereg | NM_007950 | 0.88 | 1.02 | 1.07 | 0.87 |  | 0.17** | 0.27** | 0.58** | 0.88 | 7.23E-03 |
| etv1 | NM_007960 | 0.47** | 0.61** | 0.43** | 0.71* |  | 1.03 | 1.02 | 1.04 | 1.68** | 1.83E-02 |
| eya1 | BB760085 | 0.71* | 0.40** | 1.64** | 1.08 |  | 1.63** | 0.58** | 1.22 | 0.92 | 1.71E-02 |
| fancc | BE952454 | 0.48** | 0.75** | 1.42** | 0.88 |  | 0.89 | 1.15 | 0.95 | 1.59** | 2.36E-02 |
| fgf1 | AI649186 | 0.47** | 0.71* | 0.74* | 0.86 |  | 0.74* | 0.72* | 0.97 | 0.86 | 1.61E-02 |
| figf | NM_010216 | 0.83* | 0.89 | 0.92 | 1.21* |  | 0.77** | 0.48** | 0.96 | 1.59** | 3.69E-02 |
| fkbp4 | BB456860 | 1.88** | 0.64 | 3.57** | 1.93** |  | 1.63* | 2.37** | 1.03 | 1.61* | 1.12E-02 |
| flii | NM_022009 | 0.82 | 0.16** | 1.56* | 0.77 |  | 0.60** | 1.41 | 0.64* | 1.89** | 2.68E-02 |
| fmn2 | BM228488 | 0.41** | 0.21** | 1.34 | 0.37** |  | 0.97 | 0.82 | 1.1 | 1.02 | 5.04E-03 |
| frem1 | BI452538 | 0.95 | 1.19 | 0.71* | 0.91 |  | 0.69** | 0.47** | 0.71** | 0.75* | 1.21E-03 |
| fzd3 | AU043193 | 0.49** | 0.87 | 1.31 | 0.78 |  | 1.24 | 1.3 | 0.77 | 0.92 | 2.89E-02 |
| fzd6 | NM_008056 | 1.07 | 0.41** | 0.65* | 0.93 |  | 0.93 | 0.53** | 1.06 | 0.64** | 3.23E-02 |
| gadd45b | AK010420 | 0.70* | 0.35** | 2.02** | 0.48** |  | 1.44* | 1.67* | 1.27 | 0.53** | 5.57E-03 |
| gadd45g | AK007410 | 0.59* | 0.41** | 1.88** | 0.59** |  | 4.07** | 5.16** | 3.39** | 2.78** | 5.95E-03 |
| gal | NM_010253 | 0.96 | 0.98 | 1.27 | 1.38* |  | 0.82 | 3.49** | 2.43** | 4.56** | 1.21E-02 |
| gdf10 | L42114 | 0.29** | 0.75 | 0.33** | 2.68** |  | 0.41** | 1.06 | 0.73 | 0.76 | 1.87E-03 |
| gdf15 | NM_011819 | 1.05 | 0.94 | 1.75 | 1.14 |  | 3.17** | 3.76** | 2.71** | 1.36 | 2.29E-03 |
| gja5 | AK017840 | 0.86 | 0.36** | 1.22 | 1.39* |  | 0.81 | 1 | 1.25 | 0.86 | 2.43E-02 |
| gna13 | BI662324 | 0.94 | 0.47** | 0.40** | 0.82 |  | 0.89 | 0.68 | 0.82 | 0.47** | 4.42E-02 |
| gnptab | BG144467 | 5.26** | 0.84 | 1.41* | 5.60** |  | 0.9 | 0.58* | 0.76 | 0.87 | 2.82E-03 |
| golga3 | D78270 | 0.64* | 0.46** | 1.2 | 0.57** |  | 0.86 | 1.07 | 1.03 | 1.03 | 3.07E-02 |
| gpsm1 | BC026486 | 0.74 | 0.46** | 1.72** | 0.84 |  | 0.42** | 0.95 | 0.81 | 0.68* | 3.49E-02 |
| gsk3b | BB831420 | 0.88 | 0.45** | 0.63** | 0.57** |  | 1.69** | 0.69** | 0.97 | 0.8 | 3.86E-02 |
| h2-aa | AV086906 | 3.97* | 1.76 | 0.65 | 4.15* |  | 0.27* | 15.37** | 0.48 | 0.92 | 6.05E-03 |
| h2-dma | NM_010386 | 1.32 | 1.4 | 1.77 | 2.13 |  | 0.8 | 3.47** | 0.74 | 2.13* | 2.90E-02 |
| hells | NM_008234 | 1.93* | 11.54** | 17.37** | 10.68** |  | 0.38** | 0.57 | 1.24 | 1.33 | 1.59E-02 |
| hells | AK021390 | 1.19 | 5.87** | 2.77** | 3.39** |  | 0.65 | 0.9 | 1.29 | 0.83 | 4.92E-02 |
| hes6 | AI326893 | 2.81** | 1.91** | 1 | 3.33** |  | 0.91 | 1.80* | 0.83 | 1.46 | 3.40E-02 |
| hexb | NM_010422 | 0.47** | 0.67 | 1.60* | 1.32 |  | 0.48** | 1.15 | 0.39** | 0.98 | 1.88E-03 |
| hexim1 | BI411874 | 0.69** | 0.48** | 1.47** | 1.08 |  | 0.84 | 0.53** | 0.89 | 1.12 | 1.74E-02 |
| hmgb3 | NM_008253 | 0.89 | 1.32 | 3.54** | 2.14** |  | 1.17 | 2.12** | 1.02 | 1.4 | 2.16E-02 |
| hoxa10 | AK002670 | 1.09 | 0.97 | 0.93 | 0.84 |  | 1.16 | 2.78** | 0.98 | 1.61** | 2.43E-02 |
| hoxc10 | BB779859 | 0.93 | 1.12 | 1 | 1.07 |  | 0.65* | 3.51** | 0.61* | 1.71** | 1.93E-02 |
| hps1 | BB188040 | 1.02 | 0.57 | 0.59* | 0.41** |  | 0.25** | 0.79 | 0.38** | 1.47 | 3.93E-02 |
| hrb | BB130716 | 2.36** | 0.99 | 0.87 | 2.43** |  | 1.06 | 1.09 | 1.81** | 1.81** | 1.43E-04 |
| hs1bp3 | AW541327 | 0.61** | 0.46** | 1.07 | 0.96 |  | 0.68** | 0.96 | 0.72* | 1.13 | 2.37E-02 |
| htatip2 | AF061972 | 0.84 | 0.40** | 0.74** | 0.64** |  | 0.82 | 0.92 | 0.92 | 1.38** | 1.66E-02 |
| hus1 | NM_008316 | 0.85 | 1.97** | 2.04** | 1.62** |  | 0.97 | 0.96 | 1.24 | 1.23 | 1.46E-02 |
| ick | BB376918 | 2.62** | 1.22 | 1.28* | 1.90** |  | 1.22 | 0.61** | 1.11 | 0.69** | 8.05E-03 |
| ifrd2 | BB540964 | 2.00** | 1.04 | 2.30** | 1.66 |  | 1.07 | 2.18** | 1.3 | 2.51** | 9.97E-03 |
| igf1 | NM_010512 | 2.08* | 0.63 | 0.74 | 1.2 |  | 1.17 | 2.33** | 1.47 | 2.00* | 4.75E-02 |
| igf1 | AF440694 | 1.05 | 1 | 1.59 | 3.71** |  | 1.56* | 2.24** | 1.24 | 3.08** | 4.66E-02 |
| itgb2 | NM_008404 | 1.27 | 1 | 2.18** | 3.99** |  | 1.27 | 2.81** | 0.65 | 4.16** | 8.45E-03 |
| itgb6 | NM_021359 | 0.96 | 0.97 | 0.95 | 1.04 |  | 1.11 | 2.22** | 1.33** | 1.55** | 3.45E-02 |
| itgb8 | BB504737 | 1.06 | 0.57* | 0.6 | 2.70** |  | 0.76 | 0.31** | 1.47 | 0.52* | 1.51E-03 |
| jag1 | AV359819 | 0.81* | 1.21 | 0.88 | 1.40** |  | 0.69** | 0.48** | 0.91 | 0.48** | 9.46E-03 |
| jag2 | AV264681 | 2.77** | 2.36* | 2.36** | 4.13** |  | 1.24 | 0.74 | 0.99 | 1.85* | 1.33E-02 |
| jag2 | AV264681 | 2.43** | 1.32 | 1.16 | 2.02** |  | 0.69 | 1.33 | 1.03 | 0.9 | 3.18E-02 |
| jmjd6 | AK017622 | 0.8 | 1.13 | 1.07 | 2.95** |  | 1.1 | 2.32** | 1.04 | 2.11** | 6.93E-03 |
| kazald1 | AI842353 | 0.85 | 0.50** | 1.29* | 0.57** |  | 0.86 | 0.79* | 0.66** | 0.75** | 3.23E-02 |
| kif1b | BQ175246 | 0.82 | 0.38** | 1.08 | 0.9 |  | 0.83 | 0.67 | 0.77 | 0.88 | 3.95E-02 |
| kif1b | AB023656 | 0.7 | 0.76 | 1.27 | 1.13 |  | 0.62* | 0.43** | 0.78 | 0.57* | 3.23E-02 |
| kitl | BB815530 | 0.8 | 0.97 | 1.05 | 1 |  | 1.08 | 0.43** | 1.19 | 0.69** | 1.08E-02 |
| kitl | BB815530 | 0.67* | 0.36** | 0.34** | 0.43** |  | 1 | 0.29** | 1.1 | 1.02 | 9.96E-03 |
| klk6 | NM_011177 | 1.09 | 0.96 | 1.07 | 0.71 |  | 0.40** | 0.50* | 0.59* | 0.41** | 3.96E-02 |
| l7rn6 | BC003916 | 2.52** | 1.74** | 1.89** | 4.25** |  | 1.05 | 1.36* | 1.24 | 1.22 | 4.45E-02 |
| ldb2 | NM_010698 | 0.45** | 0.72* | 0.59* | 1.27 |  | 0.84 | 0.51** | 1.28 | 1.26 | 8.50E-03 |
| lgals1 | NM_008495 | 0.78 | 0.83 | 4.32** | 5.41** |  | 1.25 | 2.98** | 0.75 | 1.80* | 1.64E-03 |
| lilrb3 | U96693 | 1.28 | 0.99 | 0.96 | 1.05 |  | 0.7 | 2.59** | 0.42** | 4.03** | 2.78E-03 |
| lmo1 | NM_057173 | 0.37** | 1.17 | 2.15** | 0.60* |  | 0.69 | 1.57* | 0.7 | 1.33 | 1.91E-02 |
| mall | AV378589 | 0.79 | 1.03 | 1.12 | 1.09 |  | 0.41** | 0.72 | 1.02 | 1.07 | 3.72E-02 |
| map4k1 | BB546619 | 1.14 | 1.14 | 1.58** | 1.24 |  | 0.85 | 2.04** | 0.82 | 2.87** | 1.26E-02 |
| mapk12 | BC021640 | 0.72* | 0.48** | 0.73* | 1.02 |  | 0.66** | 1.28 | 0.8 | 1.35* | 1.56E-02 |
| mapk1ip1 | AK009250 | 0.62** | 0.46** | 0.77** | 0.81* |  | 0.99 | 0.88 | 0.73** | 0.96 | 1.11E-03 |
| mast2 | BB233292 | 1.18* | 1.06 | 1.12 | 0.84* |  | 1.24* | 0.50** | 0.98 | 1.09 | 3.30E-02 |
| mbnl3 | NM_134163 | 1 | 0.86 | 1.59** | 0.95 |  | 0.39** | 0.38** | 1.2 | 0.56** | 7.93E-03 |
| mbnl3 | BB211386 | 1.05 | 0.94 | 1.12 | 0.94 |  | 0.67* | 0.37** | 0.74* | 0.64* | 1.81E-02 |
| mesdc2 | NM_023403 | 1.05 | 0.83* | 1.30** | 1.33** |  | 1.15* | 2.00** | 0.9 | 1.78** | 1.52E-02 |
| metrnl | BB544962 | 0.99 | 1 | 1.08 | 0.88 |  | 1.18 | 2.42** | 0.93 | 1.24* | 1.74E-02 |
| mfng | NM_008595 | 1.21 | 1.28 | 2.10** | 2.62** |  | 0.79 | 2.76** | 0.74 | 2.89** | 2.45E-02 |
| mitf | BB763517 | 0.49* | 0.34** | 0.82 | 0.91 |  | 0.88 | 0.79 | 0.95 | 0.59 | 1.72E-02 |
| mmd | AA472735 | 0.87 | 1.35* | 1.04 | 1.25 |  | 0.84 | 2.75** | 0.81 | 1.33* | 3.10E-02 |
| myd116 | NM_008654 | 0.89 | 0.47** | 1.70** | 0.96 |  | 0.53** | 1.42 | 0.48** | 1.48* | 3.56E-02 |
| myo6 | BE133806 | 0.61* | 0.30** | 1.88** | 0.60* |  | 0.53** | 0.68* | 0.73 | 0.79 | 3.53E-02 |
| narg1 | BG067031 | 1.05 | 0.97 | 1.40** | 1.74** |  | 0.92 | 2.03** | 1.68** | 1.05 | 2.23E-02 |
| ndel1 | BC021434 | 1.14 | 0.71 | 1 | 1.16 |  | 2.12* | 0.24** | 0.33** | 0.73 | 1.67E-02 |
| ndrg1 | AI987929 | 0.49** | 0.37** | 0.45** | 0.58* |  | 0.98 | 0.64* | 1.04 | 1.03 | 2.15E-02 |
| ndrg1 | AI987929 | 0.66* | 0.29** | 0.61* | 0.75 |  | 1.27 | 1.03 | 0.91 | 1.59** | 4.95E-02 |
| ndrg4 | AV006122 | 0.42** | 0.42** | 0.70* | 0.79 |  | 0.92 | 1.19 | 0.98 | 0.98 | 2.90E-02 |
| ndrg4 | AI837704 | 0.30** | 0.36** | 1.15 | 0.95 |  | 0.72 | 1.51 | 0.8 | 1.52 | 4.65E-02 |
| nfatc1 | NM_016791 | 0.88 | 0.40** | 1.03 | 1.19 |  | 1 | 0.78* | 0.91 | 0.65** | 3.94E-03 |
| nfkbia | BB096843 | 0.62** | 0.43** | 1.42* | 0.82 |  | 0.61* | 1.11 | 0.96 | 0.99 | 4.03E-02 |
| nkx6-1 | AF357883 | 0.53* | 0.32** | 2.29** | 1.13 |  | 0.91 | 0.94 | 1.09 | 0.98 | 4.29E-02 |
| nme1 | BC005629 | 0.74 | 0.75 | 3.17** | 2.38** |  | 0.79 | 2.46** | 1.43 | 1.61** | 3.69E-02 |
| notch3 | NM_008716 | 0.83 | 1.1 | 0.55* | 0.88 |  | 1.04 | 0.48** | 1.17 | 0.69 | 4.34E-02 |
| ntrk3 | BM245880 | 0.82 | 0.89 | 1.13 | 1.44** |  | 0.89 | 0.39** | 0.67* | 0.99 | 3.76E-02 |
| numb | U70674 | 0.69** | 0.37** | 1 | 0.49** |  | 1.13 | 1.05 | 0.82* | 1.07 | 4.20E-04 |
| odc1 | S64539 | 1.72** | 2.29** | 1.83** | 1.82** |  | 1.11 | 1.62** | 0.99 | 1.81** | 2.43E-02 |
| ovol1 | BC021411 | 1.01 | 1.28 | 0.81 | 0.89 |  | 0.78 | 0.41** | 0.54** | 0.68* | 5.67E-03 |
| p42pop | AF364868 | 1.37* | 2.72** | 1.39* | 1.78** |  | 1.09 | 0.9 | 1.3 | 1.36 | 1.94E-03 |
| pak3 | BB468082 | 0.39** | 0.61* | 0.82 | 0.48** |  | 0.92 | 0.7 | 1.04 | 1.08 | 9.33E-03 |
| papss2 | BF786072 | 0.21** | 0.18** | 1.26 | 0.23** |  | 1 | 0.81 | 0.77 | 0.95 | 4.63E-02 |
| papss2 | BF786072 | 0.31** | 0.16** | 0.65** | 0.29** |  | 1.02 | 0.62** | 0.92 | 0.91 | 4.71E-02 |
| papss2 | BF780807 | 0.59** | 0.11** | 0.97 | 0.39** |  | 0.72* | 0.72* | 0.77 | 0.84 | 2.84E-02 |
| pbx1 | L27453 | 0.84 | 0.42** | 0.94 | 1.05 |  | 1.26 | 0.98 | 1.01 | 0.95 | 3.76E-02 |
| pcsk2 | NM_008792 | 0.42** | 0.39** | 1.74** | 2.59** |  | 0.94 | 1.01 | 1.1 | 0.96 | 4.15E-02 |
| pcsk9 | AV010795 | 2.69** | 2.00** | 1.32* | 2.22** |  | 1.11 | 1.24 | 1.48* | 0.66** | 2.55E-02 |
| pcyt1b | BE996519 | 0.78** | 0.42** | 0.77** | 0.87* |  | 1.01 | 0.97 | 0.94 | 0.95 | 4.43E-03 |
| pdlim3 | NM_016798 | 1 | 1.09 | 1.07 | 0.78 |  | 0.85 | 2.73** | 1.49* | 2.29** | 4.52E-02 |
| pdx1 | AK020261 | 0.74* | 0.20** | 0.71* | 0.47** |  | 1.96** | 1.11 | 1.3 | 0.95 | 3.47E-02 |
| pdx1 | AK020261 | 0.60** | 0.37** | 0.70* | 0.62** |  | 1.11 | 1.08 | 1.2 | 0.92 | 4.14E-02 |
| pgf | NM_008827 | 0.50** | 0.39** | 1.03 | 0.43** |  | 1.03 | 2.29** | 2.24** | 6.64** | 2.52E-02 |
| pigt | AK019717 | 1.18 | 0.53** | 1.50** | 1.31 |  | 1.03 | 1.96** | 0.93 | 1.3 | 1.15E-02 |
| pira6 | NM_011093 | 1 | 1.16 | 1.04 | 0.95 |  | 0.82 | 3.08** | 0.9 | 5.23** | 1.86E-02 |
| pkd1 | NM_013630 | 0.77 | 0.38** | 1.25 | 0.76 |  | 1.15 | 1.3 | 0.49** | 1.67** | 1.99E-02 |
| pkhd1 | AI182499 | 2.17** | 1.03 | 2.04** | 2.38** |  | 1.11 | 1.07 | 1.2 | 0.85 | 2.40E-02 |
| pkp2 | AA516617 | 3.17** | 0.94 | 0.27** | 0.9 |  | 1.21 | 1.09 | 0.81 | 1.01 | 2.91E-02 |
| plcd1 | NM_019676 | 0.9 | 0.99 | 1.02 | 1.09 |  | 0.29** | 0.83 | 0.58** | 2.09** | 4.89E-02 |
| plcd1 | NM_019676 | 0.82 | 1.06 | 1.12 | 0.69* |  | 0.49** | 1.39 | 0.65* | 1.27 | 4.34E-02 |
| plxdc2 | AK017369 | 1.33 | 0.83 | 0.75 | 1.16 |  | 0.74 | 0.36** | 1.01 | 0.32** | 9.60E-03 |
| plxnb1 | BM119522 | 0.40** | 0.59* | 1.2 | 0.62* |  | 1.21 | 0.89 | 1.21 | 0.77 | 4.17E-02 |
| pogk | AV377712 | 0.95 | 0.43** | 0.69** | 0.63** |  | 0.82* | 0.40** | 0.87 | 0.50** | 4.25E-02 |
| ppargc1a | BB745167 | 0.47** | 0.64 | 0.37** | 0.32** |  | 2.47** | 0.78 | 0.74 | 0.65 | 2.29E-03 |
| ppp1r8 | BC025479 | 2.28** | 0.48** | 2.49** | 2.09** |  | 0.83 | 0.83 | 1.09 | 1.62 | 3.17E-02 |
| prdm1 | NM_007548 | 0.91 | 0.38** | 1.83** | 0.46** |  | 0.63** | 0.39** | 0.65* | 0.43** | 9.63E-03 |
| prox1 | BE994433 | 0.75 | 0.97 | 0.95 | 0.86 |  | 1.17 | 0.53** | 1.02 | 0.86 | 3.87E-02 |
| prr15 | AJ132433 | 0.30** | 0.21** | 0.28** | 0.58** |  | 0.84 | 0.77 | 0.93 | 0.97 | 7.26E-03 |
| prrx2 | AK019971 | 0.86 | 1.26* | 1.06 | 1.17 |  | 0.98 | 2.24** | 2.14** | 6.54** | 2.66E-02 |
| psrc1 | NM_019976 | 1.38* | 2.19** | 4.33** | 3.12** |  | 0.49** | 0.40** | 0.87 | 0.88 | 2.30E-02 |
| ptgs1 | AA833146 | 0.85 | 1.16 | 0.78 | 1.19 |  | 0.68* | 0.46** | 0.77* | 0.63** | 3.23E-02 |
| ptgs2 | M94967 | 0.92 | 1.18 | 0.81 | 0.96 |  | 0.29** | 0.41** | 0.74** | 0.43** | 3.42E-04 |
| pttg1ip | AU018448 | 0.64** | 0.45** | 1.21 | 1.03 |  | 1.08 | 1.01 | 1.15 | 0.89 | 1.53E-02 |
| pttg1ip | AU018448 | 0.99 | 0.78 | 0.13** | 0.27** |  | 0.77 | 0.39** | 0.99 | 0.65 | 6.14E-03 |
| rac1 | BC003828 | 0.88 | 0.82 | 1.37 | 0.95 |  | 1.22 | 2.54** | 1.06 | 1.96** | 4.22E-02 |
| racgap1 | NM_012025 | 1.03 | 1.35 | 8.64** | 2.05* |  | 0.47* | 0.88 | 1.02 | 1.59 | 6.56E-03 |
| rnf6 | BI738010 | 0.50** | 0.56** | 1.07 | 0.48** |  | 0.89 | 0.86 | 1.28 | 0.81 | 2.74E-02 |
| rnf6 | BI738010 | 0.75* | 0.43** | 1.18 | 0.45** |  | 1.30* | 0.95 | 1.11 | 1.1 | 4.03E-02 |
| robo1 | BG065230 | 0.91 | 0.74 | 0.57 | 0.89 |  | 0.85 | 0.34** | 0.66 | 0.45* | 1.08E-02 |
| robo1 | BB176702 | 1.12 | 1.16 | 0.31** | 1.03 |  | 1.14 | 0.46** | 0.57** | 1.68* | 1.15E-02 |
| rod1 | BB519382 | 0.47** | 0.59** | 0.22** | 0.84 |  | 0.92 | 0.38** | 1.63** | 0.81 | 1.20E-02 |
| rorc | AJ132394 | 1.48 | 0.98 | 1.38 | 1.19 |  | 1.69 | 3.39** | 1.21 | 2.93** | 7.34E-03 |
| rtn4 | BE988775 | 0.67 | 0.49** | 0.61* | 0.64* |  | 1.09 | 0.56* | 1.09 | 0.60* | 1.42E-03 |
| rtn4 | AK003859 | 0.57** | 0.43** | 1.24* | 0.60** |  | 0.98 | 0.98 | 1 | 1.06 | 1.75E-02 |
| runx1t1 | X79989 | 0.94 | 0.38** | 0.94 | 1.54* |  | 0.86 | 0.77 | 0.37** | 0.98 | 2.95E-02 |
| runx1t1 | AW550878 | 1.01 | 0.84 | 0.68** | 0.79 |  | 0.54** | 0.37** | 0.85 | 0.67** | 1.45E-02 |
| runx1t1 | AV327778 | 0.87 | 0.66** | 0.85 | 1.15 |  | 0.82 | 0.46** | 1.08 | 0.87 | 9.26E-03 |
| runx2 | D14636 | 1.04 | 0.98 | 1.29* | 1.32* |  | 0.50** | 0.58** | 0.77* | 0.79* | 6.48E-03 |
| s100a11 | BC021916 | 0.91 | 0.37** | 1.32 | 1.84* |  | 0.54* | 2.58** | 0.69 | 0.47** | 1.30E-02 |
| s100a6 | NM_011313 | 0.49** | 0.92 | 1.36* | 1.51** |  | 0.57** | 1.45** | 0.68** | 1.42** | 4.32E-02 |
| scin | NM_009132 | 1.21 | 0.91 | 0.60** | 1.14 |  | 1.07 | 0.51** | 1.08 | 0.63** | 7.55E-03 |
| scmh1 | BB297140 | 0.97 | 0.9 | 1.01 | 1.05 |  | 0.91 | 0.41** | 0.62** | 1.01 | 2.94E-02 |
| scn8a | BB429612 | 2.60** | 0.68 | 1 | 2.21** |  | 1.09 | 1.05 | 0.96 | 1 | 8.32E-03 |
| sdcbp2 | BC005556 | 0.49** | 0.56** | 2.25** | 0.98 |  | 0.71* | 1.12 | 0.83 | 1.13 | 1.40E-02 |
| sema3d | BB499147 | 1.02 | 1.26 | 0.93 | 0.9 |  | 0.75 | 0.32** | 1.38 | 0.55** | 2.72E-02 |
| sema3e | NM_011348 | 1.17 | 1.12 | 1.08 | 1.17 |  | 0.61** | 0.39** | 0.83* | 0.69** | 1.44E-03 |
| sema3e | Z93948 | 1.08 | 1.22 | 1.07 | 1.01 |  | 0.82* | 0.37** | 1.17 | 0.53** | 1.81E-02 |
| sema6d | BB462688 | 0.51** | 0.51** | 1.50* | 0.63** |  | 0.70* | 1.26 | 0.9 | 1.34 | 1.63E-02 |
| serinc3 | BM239368 | 0.69* | 0.91 | 0.94 | 0.95 |  | 0.95 | 0.45** | 0.65* | 0.9 | 3.83E-02 |
| sfpi1 | NM_011355 | 1.1 | 0.66* | 0.97 | 1.31 |  | 0.82 | 2.42** | 0.85 | 4.02** | 1.29E-02 |
| sfxn1 | BB478992 | 2.05** | 0.77 | 2.45** | 1.98** |  | 0.96 | 1.82** | 1.07 | 1.36 | 1.25E-02 |
| siah2 | AA414485 | 0.47** | 0.64** | 1.37** | 0.81 |  | 0.56** | 1.65** | 0.63** | 2.85** | 1.70E-02 |
| six6os1 | AK015397 | 2.77** | 0.48** | 0.52** | 0.95 |  | 1.12 | 1.06 | 0.98 | 0.91 | 4.18E-03 |
| skil | AK018608 | 1.06 | 0.67* | 1.16 | 0.76 |  | 0.75 | 0.49** | 1.05 | 0.46** | 7.56E-03 |
| slc30a1 | BE685959 | 1.92** | 1.27* | 3.45** | 1.65** |  | 1.08 | 0.35** | 1.23* | 0.69** | 3.93E-02 |
| slc5a1 | AF208031 | 0.89 | 1 | 1.11 | 0.93 |  | 0.32** | 0.31** | 0.70* | 0.26** | 1.29E-03 |
| slc5a1 | AV371434 | 1.09 | 1.03 | 0.9 | 0.94 |  | 0.50** | 0.41** | 0.73** | 0.54** | 1.65E-03 |
| smap1 | BC006946 | 0.78* | 0.91 | 0.93 | 0.95 |  | 0.88 | 0.48** | 1.15 | 0.60** | 7.93E-03 |
| smarca1 | NM_053123 | 0.43** | 0.32** | 0.57** | 0.62* |  | 1.13 | 0.76 | 1.27 | 0.99 | 3.29E-02 |
| smarcb1 | NM_011418 | 0.95 | 0.95 | 2.41** | 1.51** |  | 0.47** | 1.15 | 0.83 | 2.08** | 9.93E-03 |
| smpd3 | BF456582 | 1.32 | 1.95** | 1.80** | 2.88** |  | 0.87 | 0.92 | 0.93 | 0.86 | 4.73E-02 |
| smurf1 | BB201890 | 0.99 | 0.94 | 0.71* | 0.91 |  | 1.23 | 0.46** | 0.71** | 0.89 | 8.92E-03 |
| smyd1 | C78565 | 1.02 | 1.19* | 0.96 | 0.94 |  | 0.63** | 2.59** | 1.01 | 2.22** | 1.60E-02 |
| smyd1 | NM_009762 | 1.01 | 0.98 | 1.13 | 1.07 |  | 0.71* | 3.14** | 1.18 | 1.85** | 2.88E-02 |
| socs2 | NM_007706 | 0.82 | 0.39** | 1.58* | 0.93 |  | 1.24 | 1.31 | 0.92 | 0.91 | 1.16E-02 |
| sorl1 | AK013519 | 1.14 | 0.34** | 1.16 | 0.62* |  | 0.88 | 0.50** | 0.95 | 0.53** | 1.73E-02 |
| speg | NM_007463 | 0.36** | 0.33** | 1.37 | 4.92** |  | 0.7 | 1.21 | 1.15 | 1.05 | 2.59E-02 |
| spon2 | NM_133903 | 0.39** | 0.6 | 1.52 | 0.59 |  | 0.97 | 1.23 | 0.44* | 2.22* | 3.42E-02 |
| spry2 | BB529691 | 0.51* | 0.57* | 1.71 | 1.91* |  | 0.77 | 0.43** | 1.09 | 0.65 | 3.55E-02 |
| sqstm1 | BM232298 | 0.44** | 0.28** | 2.68** | 0.57* |  | 1.93* | 2.25** | 0.79 | 0.91 | 3.53E-02 |
| srpk2 | NM_009274 | 0.81* | 0.50** | 1.11 | 0.87 |  | 0.84 | 0.76** | 0.9 | 0.85 | 6.07E-03 |
| srpk2 | BB127386 | 0.95 | 0.53** | 1.19 | 0.75* |  | 0.58** | 0.39** | 0.91 | 1.06 | 2.47E-02 |
| srpk2 | BE979921 | 1.51** | 0.66** | 0.87 | 1.04 |  | 0.96 | 0.26** | 0.52** | 1.40* | 6.61E-03 |
| ss18l1 | BG075210 | 0.71** | 0.36** | 0.95 | 0.80* |  | 0.91 | 0.95 | 1.02 | 1.23* | 1.42E-02 |
| stat5a | U36502 | 0.56** | 0.40** | 0.82 | 1.01 |  | 0.71* | 0.69* | 0.91 | 0.9 | 3.12E-03 |
| stmn1 | BC010581 | 0.69 | 2.83** | 6.24** | 1.74** |  | 1.04 | 0.91 | 1.02 | 0.93 | 3.30E-02 |
| stx2 | NM_007941 | 0.48** | 0.81 | 0.84 | 1.04 |  | 1.05 | 1.04 | 0.76 | 1.4 | 1.51E-03 |
| tagln | BB114067 | 0.98 | 0.56* | 1.31 | 3.52** |  | 1.56 | 2.83** | 0.47** | 2.56** | 2.85E-02 |
| tcfap2c | BC003778 | 1.05 | 1.23 | 0.98 | 0.89 |  | 0.78* | 0.49** | 1.09 | 0.93 | 4.01E-02 |
| tfdp1 | BG075396 | 3.08** | 1.70** | 3.28** | 3.13** |  | 1.11 | 1.43* | 1.52* | 1.27 | 9.57E-03 |
| tial1 | NM_009383 | 1.06 | 0.97 | 1.97** | 0.76* |  | 0.71** | 2.25** | 0.88 | 2.52** | 1.68E-03 |
| timeless | BM230269 | 1.05 | 3.50** | 2.49** | 3.47** |  | 0.73 | 1.77** | 1.23 | 2.11** | 1.30E-03 |
| timeless | BM230269 | 1 | 0.94 | 3.00** | 1.63** |  | 0.77 | 0.50** | 1.45* | 1.83** | 3.15E-02 |
| tlr3 | NM_126166 | 0.45** | 0.78 | 0.67 | 0.55* |  | 0.78 | 0.78 | 0.85 | 0.84 | 3.66E-03 |
| tnfrsf12a | NM_013749 | 0.59** | 0.85 | 2.53** | 0.72** |  | 1.11 | 3.15** | 1.38** | 1.17 | 4.16E-02 |
| tnfrsf12a | NM_013749 | 0.86 | 0.87 | 2.29** | 0.85 |  | 1.18 | 2.77** | 1.39** | 1.11 | 4.89E-03 |
| tpm2 | AK003186 | 1 | 0.93 | 1.25 | 1.13 |  | 1.11 | 6.32** | 1.33 | 1.46 | 4.67E-02 |
| traf6 | AV244412 | 0.76* | 0.33** | 0.99 | 1.04 |  | 1.11 | 1.12 | 1.16 | 0.97 | 2.96E-02 |
| trim27 | NM_009054 | 1.55** | 1.54** | 1.47** | 1.89** |  | 0.68* | 2.67** | 0.53** | 2.43** | 2.66E-02 |
| trp53bp2 | BB814564 | 0.86 | 0.41** | 1.50* | 0.98 |  | 0.61** | 0.79 | 0.81 | 0.92 | 3.77E-02 |
| tshz1 | AV291373 | 1.33* | 0.64** | 0.83 | 1 |  | 0.83 | 0.26** | 1.15 | 0.51** | 5.36E-03 |
| tshz2 | BC028776 | 0.24** | 1.14 | 0.76 | 1.05 |  | 1.05 | 1.01 | 1.03 | 1.38 | 1.23E-02 |
| tspan5 | AK015705 | 0.70* | 0.36** | 0.52** | 1.14 |  | 0.79 | 1.32 | 0.86 | 1.15 | 4.55E-02 |
| uhrf1 | BB702754 | 1.68 | 7.49** | 7.70** | 3.80** |  | 0.68 | 0.76 | 1.47 | 1.42 | 3.06E-02 |
| unknown | BQ175796 | 0.29** | 0.75* | 0.76* | 0.40** |  | 0.8 | 0.70* | 0.72* | 0.73* | 7.66E-03 |
| unknown | BF471533 | 0.56** | 0.40** | 0.88 | 1.12 |  | 0.76* | 0.64** | 1.32* | 0.92 | 1.96E-02 |
| unknown | BB235876 | 1.4 | 0.99 | 0.86 | 0.99 |  | 3.60** | 1.14 | 5.35** | 0.95 | 3.03E-02 |
| unknown | AW047257 | 1.07 | 0.20** | 0.95 | 0.71 |  | 0.52* | 0.47** | 0.75 | 0.7 | 3.76E-02 |
| unknown | BB071777 | 1.44** | 1.24 | 0.78* | 1.58** |  | 0.86 | 0.44** | 0.79* | 0.52** | 1.40E-02 |
| unknown | AU042527 | 1.07 | 1.02 | 1.26 | 0.94 |  | 1.33 | 0.42** | 0.79 | 0.9 | 1.45E-02 |
| utrn | AW541437 | 1 | 0.97 | 0.74 | 0.98 |  | 0.45** | 0.68 | 0.34** | 1.37 | 1.12E-02 |
| vegfc | BB089170 | 1.18 | 0.69** | 0.60** | 1.72** |  | 0.76* | 0.40** | 0.81 | 0.77* | 2.21E-02 |
| vps13a | AV255519 | 2.07** | 1.85** | 1.62** | 2.27** |  | 1.08 | 0.53** | 1.35 | 0.60** | 2.58E-02 |
| wnt4 | NM_009523 | 0.76 | 0.24** | 1.41 | 0.52* |  | 0.56 | 1.12 | 0.71 | 0.76 | 3.11E-02 |
| wnt5b | AV303043 | 2.83** | 1.02 | 2.04** | 2.01** |  | 0.91 | 0.83 | 0.84 | 0.57** | 4.45E-02 |
| zbtb16 | Z47205 | 0.96 | 0.67* | 0.79 | 0.61* |  | 1.83** | 0.51** | 0.87 | 0.43** | 3.11E-02 |
| zeb2 | AK012377 | 0.92 | 0.91 | 0.71** | 0.98 |  | 3.16** | 2.13** | 1.25 | 1.62** | 6.83E-03 |
| zfhx3 | NM_007496 | 0.65* | 0.18** | 0.38** | 0.82 |  | 0.8 | 0.71 | 0.92 | 0.91 | 4.38E-02 |
| zfp39 | BB311524 | 1.22 | 0.58** | 0.89 | 1.03 |  | 1.52** | 0.25** | 0.92 | 0.91 | 8.76E-03 |

**Table S6: Mature β-cell differentiation genes showing significant change in expression within 8 hours of MYC-ER^TAM^ activation.**

**Genes relating to mature β-cell development showing a significant change in expression within 8 hours following activation of MYC-ER^TAM^. ‘MYC-response p-value’ is the p-value identified for the highest-order interaction of the MYC activation variable and represents the significance of this term within the selected model. Flags represent contrast p-values comparing 4OHT-treated and vehicle-treated samples at specific time points (‘*’, p ≤ 0.05; ‘**’ p ≤ 0.01). Cells are colour-coded based on a detected fold-change greater than 1.5-fold (red, up-regulated; blue, down-regulated).**

|  |  | **Pancreatic β-cells** | | | |  | **Suprabasal Keratinocytes** | | | | **Myc-response p-value** |
| --- | --- | --- | --- | --- | --- | --- | --- | --- | --- | --- | --- |
| **Gene Symbol** | **RefSeq** | **4 hrs** | **8 hrs** | **16 hrs** | **32 hrs** |  | **4 hrs** | **8 hrs** | **16 hrs** | **32 hrs** |
| 1110003e01rik | NM_133697 | 0.64** | 0.35** | 1.61** | 0.62** |  | 0.88 | 1.26 | 0.71* | 1.34* | 3.81E-02 |
| 1110003e01rik | BB701294 | 0.60** | 0.64** | 0.84 | 0.46** |  | 0.94 | 0.58** | 0.89 | 0.66** | 1.15E-04 |
| 1810015c04rik | NM_025459 | 0.77 | 0.33** | 0.94 | 0.44** |  | 2.01** | 0.96 | 1.06 | 0.69* | 1.67E-02 |
| acot11 | NM_025590 | 0.65** | 0.30** | 1.07 | 0.83 |  | 0.88 | 0.66** | 1.08 | 0.9 | 4.44E-04 |
| amy1 | NM_007446 | 1.37* | 1.11 | 1.3 | 1.15 |  | 1.16 | 2.10** | 0.71* | 1.54** | 1.99E-02 |
| arfgap3 | BG067878 | 0.50** | 0.51** | 0.96 | 0.40** |  | 0.96 | 1.66** | 0.73 | 1.89** | 1.44E-02 |
| atf5 | AF375476 | 1.05 | 0.66 | 1.57* | 1 |  | 0.92 | 2.94** | 0.88 | 3.18** | 3.70E-02 |
| c1qb | NM_009777 | 0.84 | 0.56 | 4.41** | 3.54** |  | 1.69 | 6.50** | 0.38* | 6.73** | 1.93E-02 |
| c1qb | AW227993 | 1.31* | 0.77* | 0.87 | 1.59** |  | 0.98 | 1.24* | 0.91 | 2.00** | 1.06E-02 |
| c1qb | BB111335 | 1.26* | 0.99 | 0.89 | 1.93** |  | 0.63** | 1.24* | 1.05 | 2.12** | 3.61E-03 |
| c3 | K02782 | 0.94 | 0.52* | 2.28* | 2.22** |  | 0.83 | 1.01 | 0.40** | 0.87 | 5.19E-03 |
| ccl19 | NM_011888 | 0.78 | 1.4 | 2.75** | 2.03** |  | 1.34 | 1.06 | 0.81 | 2.66** | 5.15E-03 |
| cd84 | NM_013489 | 0.96 | 0.93 | 1.55** | 2.20** |  | 0.87 | 1.06 | 0.88 | 2.25** | 3.40E-02 |
| creg1 | BC027426 | 2.08** | 2.12** | 1.60** | 2.16** |  | 0.69* | 0.85 | 0.98 | 0.92 | 3.68E-02 |
| ddc | AF071068 | 0.63** | 0.41** | 1.53* | 1.11 |  | 0.94 | 0.81 | 0.65* | 1.07 | 1.79E-02 |
| defb1 | BC024380 | 0.34** | 0.42** | 0.9 | 0.22** |  | 0.83 | 0.50** | 0.85 | 0.27** | 4.86E-02 |
| defb1 | BC024380 | 0.31** | 0.47** | 0.8 | 0.18** |  | 0.8 | 0.51** | 0.6 | 0.30** | 3.33E-02 |
| dio1 | NM_007860 | 0.63* | 0.54** | 0.79 | 0.27** |  | 1.06 | 0.82 | 1.03 | 0.89 | 7.49E-03 |
| egf | NM_010113 | 1.05 | 0.79 | 0.92 | 1.38* |  | 1.62** | 0.47** | 0.91 | 0.84 | 1.28E-02 |
| fkbp1b | NM_016863 | 0.86 | 0.47** | 1.09 | 0.67* |  | 0.87 | 0.82 | 0.56** | 1.34 | 2.90E-02 |
| gimap4 | BC005577 | 0.64** | 0.57** | 1.07 | 0.72* |  | 0.65* | 2.38** | 1.76** | 2.04** | 2.21E-02 |
| glrb | BB345174 | 0.80* | 1.05 | 0.98 | 0.92 |  | 0.58** | 0.80* | 0.65** | 0.41** | 8.88E-03 |
| gramd3 | AV259880 | 0.70** | 0.36** | 0.61** | 0.68** |  | 0.60** | 1.15 | 0.81* | 1.41** | 2.83E-04 |
| h2-aa | AV086906 | 3.97* | 1.76 | 0.65 | 4.15* |  | 0.27* | 15.37** | 0.48 | 0.92 | 6.05E-03 |
| h2-d1 | M33151 | 1.04 | 1.98* | 0.74 | 0.93 |  | 0.57 | 16.95** | 0.54 | 1.18 | 1.99E-02 |
| h2-d1 | X00246 | 0.94 | 0.91 | 0.88 | 0.7 |  | 0.96 | 0.40** | 1.17 | 1.37 | 8.64E-03 |
| h2-d1 | NM_010380 | 0.29* | 0.38 | 2.22 | 1 |  | 0.98 | 0.03** | 0.61 | 1.62 | 4.78E-03 |
| h2-d1 | M58156 | 1.07 | 1.06 | 0.95 | 0.99 |  | 1.29* | 2.46** | 0.97 | 0.63** | 3.40E-02 |
| h2-d1 | M34962 | 2.81 | 2.55* | 1.4 | 5.07** |  | 0.28* | 39.62** | 0.53 | 1.31 | 2.31E-03 |
| h2-d1 | L36068 | 1.51 | 1.28 | 1.89 | 3.61* |  | 0.35 | 36.81** | 0.88 | 1.05 | 1.25E-02 |
| h2-d1 | J00406 | 0.7 | 0.9 | 0.89 | 0.67* |  | 0.96 | 0.33** | 0.64* | 1.54* | 2.31E-03 |
| h2-l | M86502 | 2.32 | 1.79 | 1.83 | 6.04* |  | 0.24 | 65.12** | 0.8 | 1.04 | 5.14E-03 |
| h2-l | M69068 | 2.71 | 1.98 | 1.72 | 6.72** |  | 0.32 | 48.04** | 0.64 | 0.91 | 4.38E-02 |
| ifi203 | NM_008328 | 0.88 | 0.93 | 0.45** | 1.49* |  | 0.84 | 0.41** | 0.53** | 0.65** | 8.14E-03 |
| il1r1 | NM_008362 | 0.53** | 0.41** | 1.56* | 0.62* |  | 0.57** | 1.29 | 1.02 | 1.33 | 5.95E-03 |
| il6ra | X53802 | 0.35** | 0.45* | 0.87 | 0.42** |  | 1.4 | 0.77 | 1.06 | 0.7 | 4.70E-03 |
| itga7 | NM_008398 | 0.85 | 0.86 | 1.40* | 2.86** |  | 0.50* | 1.67* | 0.76 | 2.48** | 2.14E-02 |
| kl | BQ175355 | 0.81 | 0.35** | 0.92 | 0.86 |  | 1.04 | 1.09 | 0.87 | 0.89 | 2.13E-02 |
| klk1 | BC010754 | 0.74 | 1.44 | 1.12 | 1.74 |  | 1.95 | 11.58** | 20.38** | 244.79** | 1.13E-04 |
| klk1 | BC010754 | 1.31 | 1.36 | 1.01 | 1.93 |  | 2.72* | 16.74** | 13.93** | 255.71** | 4.94E-05 |
| klk1 | AV053098 | 1 | 0.72 | 1.88* | 1.56 |  | 1.75* | 1.74 | 6.60** | 11.37** | 1.69E-03 |
| klk21 | AB039276 | 0.63** | 0.87 | 1.24 | 1.12 |  | 1.11 | 3.36** | 4.16** | 17.78** | 8.87E-03 |
| lamp2 | BB390704 | 0.99 | 0.50** | 1.18 | 0.87 |  | 0.71* | 0.99 | 0.85 | 1.11 | 4.19E-02 |
| mapk10 | BB453775 | 0.46** | 0.43** | 1.13 | 0.87 |  | 0.92 | 0.77** | 1.13 | 0.68** | 4.76E-03 |
| mapk10 | L35236 | 0.48** | 0.22** | 1.50** | 1.73** |  | 0.91 | 1 | 1.06 | 0.92 | 2.96E-02 |
| ndrg4 | AV006122 | 0.42** | 0.42** | 0.70* | 0.79 |  | 0.92 | 1.19 | 0.98 | 0.98 | 2.90E-02 |
| ndrg4 | AI837704 | 0.30** | 0.36** | 1.15 | 0.95 |  | 0.72 | 1.51 | 0.8 | 1.52 | 4.65E-02 |
| nr1h3 | NM_013839 | 1.2 | 0.91 | 1.19 | 1.09 |  | 0.87 | 2.49** | 0.86 | 1.09 | 2.03E-02 |
| nupr1 | NM_019738 | 0.58** | 0.30** | 1.26 | 0.51** |  | 1.22 | 1.28 | 0.46** | 1.14 | 6.78E-03 |
| nupr1 | NM_019738 | 0.62* | 0.46** | 1.23 | 0.66* |  | 1.24 | 1.3 | 0.49** | 1.04 | 3.42E-02 |
| papss2 | BF786072 | 0.21** | 0.18** | 1.26 | 0.23** |  | 1 | 0.81 | 0.77 | 0.95 | 4.63E-02 |
| papss2 | BF786072 | 0.31** | 0.16** | 0.65** | 0.29** |  | 1.02 | 0.62** | 0.92 | 0.91 | 4.71E-02 |
| papss2 | BF780807 | 0.59** | 0.11** | 0.97 | 0.39** |  | 0.72* | 0.72* | 0.77 | 0.84 | 2.84E-02 |
| pcnt | NM_008787 | 2.70** | 3.79** | 5.22** | 4.18** |  | 1.14 | 1.89** | 1.1 | 1.82** | 1.37E-02 |
| pcp4 | NM_008791 | 0.59** | 0.59 | 0.86 | 0.29** |  | 0.82 | 1.22 | 0.73 | 0.73 | 9.36E-03 |
| pcsk2 | BB357975 | 2.24** | 0.56* | 0.69* | 1.08 |  | 1.24 | 1.12 | 1.04 | 0.83 | 1.33E-02 |
| pcsk2 | AI839700 | 0.98 | 0.48** | 0.71** | 0.93 |  | 1.03 | 1.08 | 0.89 | 0.89 | 3.31E-02 |
| pcsk2 | NM_008792 | 0.42** | 0.39** | 1.74** | 2.59** |  | 0.94 | 1.01 | 1.1 | 0.96 | 4.15E-02 |
| pftk1 | AI327038 | 0.33** | 0.99 | 1.3 | 0.8 |  | 1.1 | 0.96 | 1 | 1.11 | 3.86E-02 |
| pftk1 | BB027193 | 0.94 | 1.11 | 0.46** | 1.1 |  | 0.72 | 0.81 | 0.64** | 0.97 | 4.12E-02 |
| pgcp | BB468025 | 0.74 | 0.51** | 1 | 0.85 |  | 0.89 | 1.48* | 0.67* | 1.32 | 2.74E-02 |
| pip5k1b | NM_008846 | 0.28** | 0.74 | 2.51** | 1.07 |  | 1.01 | 0.66 | 1.14 | 1.36 | 8.44E-03 |
| pip5k1b | NM_008846 | 0.64* | 0.45** | 1.75** | 1.55* |  | 0.9 | 0.99 | 1.1 | 0.87 | 1.98E-02 |
| prkce | AK017901 | 0.98 | 0.65** | 1 | 1.19 |  | 1.25 | 1.13 | 1.89** | 3.13** | 1.69E-03 |
| prkcz | BB430502 | 0.75* | 0.68** | 1.53** | 0.41** |  | 1.06 | 1.2 | 1 | 0.82 | 1.29E-02 |
| psmb9 | NM_013585 | 0.41** | 0.21** | 2.01** | 0.64* |  | 1.26 | 1.45 | 0.7 | 1.76** | 4.89E-02 |
| ptprn | NM_008985 | 0.38** | 0.46** | 0.82 | 1.79* |  | 0.83 | 0.98 | 0.85 | 0.97 | 8.88E-03 |
| rab7l1 | BC016133 | 0.87 | 0.65** | 2.04** | 2.18** |  | 0.94 | 1.50** | 0.97 | 1.83** | 2.49E-02 |
| rgs2 | AF215668 | 1 | 0.32** | 2.41** | 1.03 |  | 0.81 | 1 | 0.7 | 1.05 | 1.19E-02 |
| slc25a5 | AA823938 | 0.93 | 0.94 | 3.62** | 2.54** |  | 0.86 | 1.55* | 0.94 | 1.51* | 1.23E-02 |
| slc25a5 | BM210336 | 0.82 | 0.85 | 3.76** | 2.47** |  | 0.72* | 1.52* | 0.82 | 1.36* | 9.44E-03 |
| slc25a5 | C81442 | 0.7 | 1.08 | 2.93** | 1.78** |  | 0.66** | 1.59** | 1 | 1.34* | 2.29E-02 |
| slc2a2 | NM_031197 | 0.84* | 0.43** | 0.94 | 0.76** |  | 0.74** | 1 | 1.19* | 0.91 | 2.91E-02 |
| slc7a8 | NM_016972 | 0.84 | 0.58** | 1.02 | 0.78 |  | 0.53** | 1.64** | 1.84** | 2.72** | 1.94E-02 |
| snap25 | BE952593 | 0.57** | 0.46** | 1.53** | 1.09 |  | 0.91 | 1.15 | 0.97 | 0.93 | 1.24E-02 |
| sqrdl | AF174535 | 0.52** | 0.37** | 1.48* | 0.77 |  | 0.94 | 1.35* | 0.68* | 1.19 | 3.08E-03 |
| srxn1 | BC011325 | 1.06 | 0.97 | 1.12 | 0.95 |  | 1.02 | 1.51** | 1.08 | 2.08** | 3.42E-02 |
| tapbpl | BC017613 | 0.55** | 0.41** | 1.80** | 0.77 |  | 0.63** | 1.29 | 0.74* | 1.35* | 1.01E-03 |
| tgoln1 | AI314055 | 1.1 | 0.93 | 0.76 | 1.49 |  | 2.05** | 1.55* | 1.27 | 1.45* | 2.53E-02 |
| tie1 | NM_011587 | 1.24 | 0.66* | 1.03 | 0.96 |  | 0.99 | 1.36 | 1.04 | 2.36** | 1.67E-02 |
| tmem56 | BB667728 | 0.83 | 0.91 | 1.33** | 0.88 |  | 0.92 | 0.70** | 0.85 | 0.46** | 1.14E-02 |
| tmem70 | BC011320 | 1.45 | 0.77 | 1.14 | 2.17** |  | 0.58* | 1.4 | 0.97 | 1.83** | 1.61E-02 |
| tspyl4 | BC017540 | 0.58* | 0.41** | 1.05 | 0.95 |  | 0.85 | 0.60* | 0.47** | 0.51** | 1.43E-03 |
| vsnl1 | NM_012038 | 0.54** | 0.62** | 1.24 | 0.48** |  | 0.49** | 0.84 | 1.01 | 0.94 | 2.12E-02 |

**Table S7: Gene Set Enrichment Analysis for genes showing significant change in expression within 8 hours of MYC-ER^TAM^ activation in the pancreatic beta cells.**

Gene sets enriched in genes up-regulated (red) or down-regulated (blue) in the pancreatic β-cells within 8 hours following activation of MYC-ER^TAM^. Gene sets are taken from the Molecular Signature Database and from published datasets, and only those with FDR < 0.01 are shown. Size = The number of genes in the gene set that are also in the expression data set; ES = Enrichment score; NES = Normalized enrichment score; NOM p-value = Nominal p-value; FDR q-value = False discovery rate; FWER p-value = Family-wise error rate.

| **Gene Set** | **Size** | **ES** | **NES** | **NOM**  **p-val** | **FDR**  **q-val** | **FWER**  **p-val** |
| --- | --- | --- | --- | --- | --- | --- |
| COLLER_MYC_UP | 15 | 0.736 | 3.029 | 0 | 0 | 0 |
| CHROMATIN | 23 | 0.745 | 2.808 | 0 | 0 | 0 |
| MYC_TARGETS | 31 | 0.534 | 2.727 | 0 | 0 | 0 |
| MANALO_HYPOXIA_DN | 64 | 0.691 | 2.724 | 0 | 0 | 0 |
| PENG_GLUTAMINE_DN | 190 | 0.517 | 2.631 | 0 | 0 | 0 |
| CELL_CYCLE_KEGG | 57 | 0.534 | 2.614 | 0 | 0 | 0 |
| ADIP_DIFF_CLUSTER4 | 27 | 0.739 | 2.552 | 0 | 0 | 0 |
| BRCA1_OVEREXP_DN | 74 | 0.564 | 2.532 | 0 | 0 | 0 |
| LE_MYELIN_UP | 89 | 0.485 | 2.463 | 0 | 0 | 0 |
| HESS_HOXAANMEIS1_UP | 52 | 0.638 | 2.457 | 0 | 0 | 0 |
| CMV_IE86_UP | 38 | 0.581 | 2.406 | 0 | 0 | 0 |
| SCHUMACHER_MYC_UP | 46 | 0.61 | 2.395 | 0 | 0 | 0 |
| DNA_REPLICATION_REACTOME | 33 | 0.711 | 2.382 | 0 | 0 | 0 |
| TARTE_PLASMA_BLASTIC | 220 | 0.468 | 2.346 | 0 | 0 | 0 |
| NUCLEAR_MEMBRANE_PART | 32 | 0.709 | 2.34 | 0 | 0 | 0 |
| DOX_RESIST_GASTRIC_UP | 28 | 0.512 | 2.306 | 0 | 0 | 0 |
| GENOTOXINS_ALL_24HRS_REG | 18 | 0.515 | 2.295 | 0 | 0 | 0 |
| SANSOM_APC_LOSS4_UP | 84 | 0.486 | 2.293 | 0 | 0 | 0 |
| YU_CMYC_UP | 40 | 0.604 | 2.262 | 0 | 0 | 0 |
| NUCLEAR_ENVELOPE | 58 | 0.579 | 2.25 | 0 | 0 | 0 |
| CHANG_SERUM_RESPONSE_UP | 102 | 0.508 | 2.244 | 0 | 0 | 0 |
| LEE_TCELLS3_UP | 61 | 0.462 | 2.22 | 0 | 0 | 0 |
| BRCA_PROGNOSIS_NEG | 62 | 0.471 | 2.219 | 0 | 0 | 0 |
| MEMBRANE_ENCLOSED_LUMEN | 238 | 0.468 | 2.217 | 0 | 0 | 0 |
| VERNELL_PRB_CLSTR1 | 43 | 0.651 | 2.211 | 0 | 0 | 0 |
| BADPATHWAY | 16 | 0.382 | 2.209 | 0 | 0 | 0 |
| NUCLEOLUS | 64 | 0.588 | 2.208 | 0 | 0 | 0 |
| HDACI_COLON_BUT12HRS_DN | 44 | 0.44 | 2.205 | 0 | 0 | 0 |
| BRENTANI_CELL_CYCLE | 60 | 0.393 | 2.193 | 0 | 0 | 0 |
| HG_PROGERIA_DN | 15 | 0.677 | 2.179 | 0 | 0 | 0 |
| CMV_HCMV_TIMECOURSE_24HRS_UP | 35 | 0.47 | 2.166 | 0 | 0 | 0 |
| PEART_HISTONE_DN | 46 | 0.448 | 2.155 | 0 | 0 | 0 |
| BRENTANI_REPAIR | 23 | 0.603 | 2.154 | 0 | 0 | 0 |
| HSA00260_GLYCINE_SERINE_AND_THREONINE_ METABOLISM | 21 | 0.606 | 2.139 | 0 | 0 | 0 |
| P21_P53_ANY_DN | 33 | 0.48 | 2.137 | 0 | 0 | 0 |
| HSC_INTERMEDIATEPROGENITORS_SHARED | 74 | 0.428 | 2.135 | 0 | 0 | 0 |
| CHROMOSOME | 76 | 0.551 | 2.129 | 0 | 0 | 0 |
| MOREAUX_TACI_HI_IN_PPC_UP | 45 | 0.531 | 2.124 | 0 | 0 | 0 |
| BHATTACHARYA_ESC_UP | 38 | 0.511 | 2.111 | 0 | 0 | 0 |
| CHROMOSOME__PERICENTRIC_REGION | 18 | 0.599 | 2.067 | 0 | 0 | 0 |
| CMV_24HRS_UP | 56 | 0.361 | 2.065 | 0 | 0 | 0 |
| DNA_DAMAGE_SIGNALING | 57 | 0.413 | 2.065 | 0 | 0 | 0 |
| NUCLEOPLASM | 140 | 0.454 | 2.064 | 0 | 0 | 0 |
| BRCA_BRCA1_POS | 50 | 0.567 | 2.054 | 0 | 0 | 0 |
| LEE_MYC_UP | 34 | 0.377 | 2.049 | 0 | 0 | 0 |
| RIBONUCLEOPROTEIN_COMPLEX | 85 | 0.472 | 2.027 | 0 | 0 | 0 |
| SPLICEOSOME | 27 | 0.554 | 2.022 | 0 | 0 | 0 |
| SERUM_FIBROBLAST_CELLCYCLE | 96 | 0.504 | 2.016 | 0 | 0 | 0 |
| POD1_KO_UP | 264 | 0.382 | 2.001 | 0 | 0 | 0 |
| HSA04110_CELL_CYCLE | 80 | 0.468 | 1.993 | 0 | 0 | 0 |
| LEE_MYC_TGFA_UP | 37 | -0.465 | -1.86 | 0 | 0 | 0 |
| INTERCELLULAR_JUNCTION | 24 | -0.532 | -1.884 | 0 | 0 | 0 |
| HSA00480_GLUTATHIONE_METABOLISM | 24 | -0.506 | -1.899 | 0 | 0 | 0 |
| GOLGI_APPARATUS | 110 | -0.413 | -1.915 | 0 | 0 | 0 |
| HSA00380_TRYPTOPHAN_METABOLISM | 29 | -0.542 | -1.918 | 0 | 0 | 0 |
| CELL_JUNCTION | 30 | -0.447 | -1.918 | 0 | 0 | 0 |
| PROTEASOME_DEGRADATION | 24 | -0.467 | -1.937 | 0 | 0 | 0 |
| HSA00510_N_GLYCAN_BIOSYNTHESIS | 20 | -0.529 | -1.959 | 0 | 0 | 0 |
| GLYCEROPHOSPHOLIPID_METABOLISM | 21 | -0.461 | -1.98 | 0 | 0 | 0 |
| HSA00565_ETHER_LIPID_METABOLISM | 16 | -0.585 | -2.02 | 0 | 0 | 0 |
| HSA04640_HEMATOPOIETIC_CELL_LINEAGE | 26 | -0.532 | -2.123 | 0 | 0 | 0 |
| HSA02010_ABC_TRANSPORTERS_GENERAL | 18 | -0.637 | -2.134 | 0 | 0 | 0 |
| BETA_ALANINE_METABOLISM | 16 | -0.537 | -2.245 | 0 | 0 | 0 |
| HSA00590_ARACHIDONIC_ACID_METABOLISM | 26 | -0.537 | -2.263 | 0 | 0 | 0 |
| GNATENKO_PLATELET_UP | 23 | -0.589 | -2.327 | 0 | 0 | 0 |
| GAMMA_UNIQUE_FIBRO_DN | 29 | -0.578 | -2.359 | 0 | 0 | 0 |
| HSA00071_FATTY_ACID_METABOLISM | 27 | -0.51 | -2.386 | 0 | 0 | 0 |
| PLATELET_EXPRESSED | 20 | -0.613 | -3.342 | 0 | 0 | 0 |
| CELL_CYCLE | 52 | 0.58 | 2.786 | 0 | 0 | 0 |
| MOREAUX_TACI_HI_VS_LOW_DN | 92 | 0.519 | 2.654 | 0 | 0 | 0 |
| HESS_HOXAANMEIS1_DN | 52 | 0.638 | 2.504 | 0 | 0 | 0 |
| LI_FETAL_VS_WT_KIDNEY_DN | 104 | 0.466 | 2.397 | 0 | 0 | 0 |
| SANSOM_APC_LOSS5_UP | 57 | 0.63 | 2.107 | 0 | 0 | 0 |
| ABBUD_LIF_UP | 30 | -0.541 | -1.935 | 0 | 0 | 0 |
| STEMCELL_COMMON_UP | 120 | 0.44 | 1.998 | 0 | 0 | 0 |
| GOLGI_MEMBRANE | 24 | -0.557 | -1.841 | 0 | 0 | 0 |
| CYTOPLASMIC_VESICLE_MEMBRANE | 20 | -0.583 | -1.953 | 0 | 0 | 0 |
| COATED_VESICLE | 29 | -0.693 | -2.3 | 0 | 0 | 0 |
| MEMBRANE_BOUND_VESICLE | 63 | -0.524 | -2.439 | 0 | 0 | 0 |
| ORGANELLE_LUMEN | 238 | 0.468 | 2.53 | 0 | 0 | 0 |
| NUCLEAR_PART | 315 | 0.49 | 2.188 | 0 | 0 | 0 |
| VESICLE | 64 | -0.522 | -2.357 | 0 | 0 | 0 |
| PORE_COMPLEX | 29 | 0.682 | 2.037 | 0 | 0 | 0 |
| CYTOPLASMIC_VESICLE | 62 | -0.533 | -2.659 | 0 | 0 | 0 |
| CYTOPLASMIC_MEMBRANE_BOUND_VESICLE | 61 | -0.535 | -1.942 | 0 | 0 | 0 |
| APICOLATERAL_PLASMA_MEMBRANE | 15 | -0.619 | -2.114 | 0 | 0 | 0 |
| VESICLE_MEMBRANE | 22 | -0.564 | -2.544 | 0 | 0 | 0 |
| CHROMOSOMAL_PART | 61 | 0.592 | 2.223 | 0 | 0 | 0 |
| MORF_ESPL1 | 47 | 0.514 | 3.402 | 0 | 0 | 0 |
| MORF_HDAC2 | 175 | 0.516 | 3.314 | 0 | 0 | 0 |
| RNA_SPLICING | 53 | 0.605 | 2.764 | 0 | 0 | 0 |
| DNA_REPLICATION | 60 | 0.634 | 2.708 | 0 | 0 | 0 |
| MODULE_61 | 37 | 0.708 | 2.654 | 0 | 0 | 0 |
| GNF2_TDG | 25 | 0.746 | 2.529 | 0 | 0 | 0 |
| MODULE_57 | 42 | 0.673 | 2.475 | 0 | 0 | 0 |
| MORF_UNG | 57 | 0.564 | 2.455 | 0 | 0 | 0 |
| MORF_BUB1 | 38 | 0.61 | 2.421 | 0 | 0 | 0 |
| GNF2_PA2G4 | 60 | 0.614 | 2.413 | 0 | 0 | 0 |
| MORF_XRCC5 | 153 | 0.442 | 2.399 | 0 | 0 | 0 |
| MORF_PCNA | 64 | 0.614 | 2.385 | 0 | 0 | 0 |
| MORF_PRKDC | 117 | 0.492 | 2.384 | 0 | 0 | 0 |
| GNF2_ELAC2 | 31 | 0.673 | 2.38 | 0 | 0 | 0 |
| GNF2_MLH1 | 30 | 0.636 | 2.38 | 0 | 0 | 0 |
| GNF2_APEX1 | 66 | 0.638 | 2.342 | 0 | 0 | 0 |
| MODULE_252 | 160 | 0.449 | 2.338 | 0 | 0 | 0 |
| GNF2_RRM1 | 72 | 0.61 | 2.337 | 0 | 0 | 0 |
| NUCLEASE_ACTIVITY | 27 | 0.553 | 2.337 | 0 | 0 | 0 |
| MORF_BUB3 | 190 | 0.468 | 2.321 | 0 | 0 | 0 |
| MRNA_PROCESSING_GO_0006397 | 47 | 0.578 | 2.312 | 0 | 0 | 0 |
| MODULE_219 | 23 | 0.701 | 2.306 | 0 | 0 | 0 |
| RNA_PROCESSING | 102 | 0.54 | 2.298 | 0 | 0 | 0 |
| GCM_MSN | 17 | 0.539 | 2.293 | 0 | 0 | 0 |
| MORF_RAD23A | 226 | 0.407 | 2.248 | 0 | 0 | 0 |
| GNF2_NS | 33 | 0.57 | 2.217 | 0 | 0 | 0 |
| RNA_BINDING | 136 | 0.492 | 2.216 | 0 | 0 | 0 |
| HORMONE_RECEPTOR_BINDING | 15 | 0.543 | 2.198 | 0 | 0 | 0 |
| MORF_PTPN11 | 76 | 0.466 | 2.198 | 0 | 0 | 0 |
| MODULE_125 | 36 | 0.665 | 2.193 | 0 | 0 | 0 |
| MODULE_126 | 122 | 0.422 | 2.191 | 0 | 0 | 0 |
| MORF_PPP1CC | 110 | 0.485 | 2.184 | 0 | 0 | 0 |
| MORF_FBL | 103 | 0.485 | 2.184 | 0 | 0 | 0 |
| GNF2_G22P1 | 25 | 0.642 | 2.173 | 0 | 0 | 0 |
| MODULE_32 | 135 | 0.464 | 2.167 | 0 | 0 | 0 |
| MODULE_183 | 42 | 0.601 | 2.138 | 0 | 0 | 0 |
| MORF_MSH2 | 43 | 0.604 | 2.134 | 0 | 0 | 0 |
| MODULE_158 | 35 | 0.662 | 2.128 | 0 | 0 | 0 |
| MODULE_244 | 124 | 0.436 | 2.103 | 0 | 0 | 0 |
| GNF2_BUB3 | 17 | 0.693 | 2.082 | 0 | 0 | 0 |
| GNF2_CENPF | 51 | 0.492 | 2.075 | 0 | 0 | 0 |
| MORF_EIF3S2 | 166 | 0.411 | 2.061 | 0 | 0 | 0 |
| GNF2_RFC4 | 51 | 0.601 | 2.058 | 0 | 0 | 0 |
| MORF_FEN1 | 50 | 0.59 | 2.045 | 0 | 0 | 0 |
| MORF_SMC1L1 | 39 | 0.651 | 2.04 | 0 | 0 | 0 |
| MORF_CDC16 | 46 | 0.461 | 2.036 | 0 | 0 | 0 |
| DNA_DEPENDENT_DNA_REPLICATION | 33 | 0.668 | 2.032 | 0 | 0 | 0 |
| MODULE_403 | 34 | 0.558 | 2.02 | 0 | 0 | 0 |
| MORF_RAD54L | 68 | 0.545 | 2.019 | 0 | 0 | 0 |
| MORF_RRM1 | 71 | 0.574 | 2.002 | 0 | 0 | 0 |
| MORF_RPA1 | 44 | 0.495 | 1.999 | 0 | 0 | 0 |
| MODULE_77 | 20 | -0.47 | -1.866 | 0 | 0 | 0 |
| GNF2_HPX | 37 | -0.434 | -1.887 | 0 | 0 | 0 |
| SECRETORY_PATHWAY | 40 | -0.495 | -1.902 | 0 | 0 | 0 |
| MORF_PRKCA | 63 | -0.349 | -1.903 | 0 | 0 | 0 |
| MODULE_247 | 15 | -0.671 | -1.927 | 0 | 0 | 0 |
| LIPID_BINDING | 41 | -0.457 | -2.021 | 0 | 0 | 0 |
| GNF2_MAPT | 23 | -0.54 | -2.029 | 0 | 0 | 0 |
| MODULE_19 | 189 | -0.357 | -2.082 | 0 | 0 | 0 |
| MODULE_484 | 18 | -0.606 | -2.106 | 0 | 0 | 0 |
| MODULE_568 | 33 | -0.41 | -2.11 | 0 | 0 | 0 |
| GOLGI_VESICLE_TRANSPORT | 25 | -0.611 | -2.197 | 0 | 0 | 0 |
| GNF2_DNM1 | 32 | -0.695 | -2.203 | 0 | 0 | 0 |
| VESICLE_MEDIATED_TRANSPORT | 97 | -0.456 | -2.229 | 0 | 0 | 0 |
| SECRETION | 73 | -0.492 | -2.234 | 0 | 0 | 0 |
| RNA_SPLICING__VIA_TRANSESTERIFICATION_ REACTIONS | 22 | 0.627 | 2.176 | 0 | 0 | 0 |
| MORF_RFC4 | 101 | 0.552 | 2.239 | 0 | 0 | 0 |
| GNF2_FEN1 | 45 | 0.593 | 2.219 | 0 | 0 | 0 |
| GNF2_DEK | 40 | 0.69 | 2.663 | 0 | 0 | 0 |
| GNF2_SMC4L1 | 68 | 0.629 | 2.389 | 0 | 0 | 0 |
| GNF2_MCM5 | 45 | 0.696 | 2.599 | 0 | 0 | 0 |
| GNF2_MCM4 | 45 | 0.551 | 2.102 | 0 | 0 | 0 |
| MODULE_91 | 31 | -0.493 | -1.858 | 0 | 0 | 0 |
| MODULE_198 | 191 | 0.451 | 2.451 | 0 | 0 | 0 |
| GNF2_NPM1 | 41 | 0.63 | 1.83 | 0 | 0 | 0.2 |
| TRANSCRIPTION | 345 | 0.307 | 1.79 | 0 | 0 | 0.2 |
| NUCLEOBASE__NUCLEOSIDE_AND_NUCLEOTIDE_ METABOLIC_PROCESS | 27 | 0.529 | 1.79 | 0 | 0 | 0.2 |
| MORF_PPP2CA | 75 | 0.444 | 1.797 | 0 | 0 | 0.2 |
| TRANSCRIPTION__DNA_DEPENDENT | 291 | 0.322 | 1.797 | 0 | 0 | 0.2 |
| STRUCTURE_SPECIFIC_DNA_BINDING | 32 | 0.59 | 1.798 | 0 | 0 | 0.2 |
| MODULE_278 | 28 | 0.528 | 1.801 | 0 | 0 | 0.2 |
| CELL_CYCLE_PHASE | 100 | 0.391 | 1.802 | 0 | 0 | 0.2 |
| SPLICEOSOME_ASSEMBLY | 15 | 0.578 | 1.804 | 0 | 0 | 0.2 |
| MITOTIC_CELL_CYCLE | 97 | 0.358 | 1.815 | 0 | 0 | 0.2 |
| MODULE_16 | 272 | 0.37 | 1.816 | 0 | 0 | 0.2 |
| MODULE_389 | 68 | 0.37 | 1.818 | 0 | 0 | 0.2 |
| GNF2_CDC20 | 51 | 0.494 | 1.83 | 0 | 0 | 0.2 |
| REGULATION_OF_DNA_METABOLIC_PROCESS | 28 | 0.522 | 1.831 | 0 | 0 | 0.2 |
| MODULE_102 | 16 | 0.777 | 1.838 | 0 | 0 | 0.2 |
| GCM_PPP1CC | 43 | 0.459 | 1.841 | 0 | 0 | 0.2 |
| NUCLEOCYTOPLASMIC_TRANSPORT | 51 | 0.455 | 1.843 | 0 | 0 | 0.2 |
| GNF2_CKS1B | 36 | 0.572 | 1.843 | 0 | 0 | 0.2 |
| TRANSFERASE_ACTIVITY__TRANSFERRING_ONE_ CARBON_GROUPS | 21 | 0.594 | 1.847 | 0 | 0 | 0.2 |
| PEPTIDE_BINDING | 30 | 0.423 | 1.859 | 0 | 0 | 0.2 |
| MRNA_METABOLIC_PROCESS | 52 | 0.563 | 1.862 | 0 | 0 | 0.2 |
| MODULE_159 | 47 | 0.485 | 1.885 | 0 | 0 | 0.2 |
| MODULE_281 | 17 | 0.493 | 1.886 | 0 | 0 | 0.2 |
| MODULE_17 | 233 | 0.385 | 1.888 | 0 | 0 | 0.2 |
| GNF2_ANP32B | 29 | 0.631 | 1.896 | 0 | 0 | 0.2 |
| GNF2_PCNA | 57 | 0.588 | 1.897 | 0 | 0 | 0.2 |
| CELL_CYCLE_GO_0007049 | 180 | 0.373 | 1.902 | 0 | 0 | 0.2 |
| GNF2_RFC3 | 34 | 0.615 | 1.908 | 0 | 0 | 0.2 |
| GNF2_KPNB1 | 51 | 0.66 | 1.911 | 0 | 0 | 0.2 |
| MORF_CUL1 | 42 | 0.518 | 1.913 | 0 | 0 | 0.2 |
| GNF2_CKS2 | 43 | 0.491 | 1.913 | 0 | 0 | 0.2 |
| MORF_HAT1 | 117 | 0.51 | 1.916 | 0 | 0 | 0.2 |
| GNF2_SMC2L1 | 29 | 0.538 | 1.918 | 0 | 0 | 0.2 |
| POSITIVE_REGULATION_OF_PROTEIN_ MODIFICATION_PROCESS | 15 | 0.486 | 1.924 | 0 | 0 | 0.2 |
| GNF2_MSH6 | 26 | 0.599 | 1.929 | 0 | 0 | 0.2 |
| GNF2_CCNB2 | 52 | 0.494 | 1.931 | 0 | 0 | 0.2 |
| MODULE_98 | 237 | 0.418 | 1.933 | 0 | 0 | 0.2 |
| MODULE_331 | 33 | 0.512 | 1.937 | 0 | 0 | 0.2 |
| MODULE_277 | 22 | 0.574 | 1.938 | 0 | 0 | 0.2 |
| GNF2_FBL | 72 | 0.512 | 1.945 | 0 | 0 | 0.2 |
| GNF2_PPP6C | 21 | 0.525 | 1.954 | 0 | 0 | 0.2 |
| MORF_DEK | 163 | 0.397 | 1.956 | 0 | 0 | 0.2 |
| GNF2_DAP3 | 58 | 0.47 | 1.957 | 0 | 0 | 0.2 |
| GNF2_HAT1 | 38 | 0.669 | 1.96 | 0 | 0 | 0.2 |
| GNF2_H2AFX | 27 | 0.489 | 1.96 | 0 | 0 | 0.2 |
| GNF2_CENPE | 36 | 0.474 | 1.963 | 0 | 0 | 0.2 |
| MORF_GSPT1 | 35 | 0.581 | 1.973 | 0 | 0 | 0.2 |
| NUCLEOTIDYLTRANSFERASE_ACTIVITY | 31 | 0.543 | 1.973 | 0 | 0 | 0.2 |
| MORF_UBE2N | 64 | 0.445 | 1.985 | 0 | 0 | 0.2 |
| GCM_GSPT1 | 58 | -0.347 | -1.777 | 0 | 0 | 0.4 |
| GNF2_RAB3A | 19 | -0.685 | -1.803 | 0 | 0 | 0.4 |
| PHOSPHOPROTEIN_PHOSPHATASE_ACTIVITY | 48 | -0.37 | -1.756 | 0 | 0 | 0.4 |
| HYDROLASE_ACTIVITY__HYDROLYZING_O_ GLYCOSYL_COMPOUNDS | 17 | -0.567 | -1.803 | 0 | 0 | 0.4 |
| NUCLEAR_PORE | 27 | 0.703 | 1.842 | 0 | 0 | 0.2 |
| REPLICATION_FORK | 15 | 0.706 | 1.888 | 0 | 0 | 0.2 |
| ADIP_DIFF_CLUSTER3 | 23 | 0.463 | 1.907 | 0 | 0 | 0.2 |
| ADIP_DIFF_CLUSTER5 | 31 | 0.468 | 1.81 | 0 | 0 | 0.2 |
| HSC_EARLYPROGENITORS_FETAL | 250 | 0.373 | 1.859 | 0 | 0 | 0.2 |
| CANTHARIDIN_DN | 37 | 0.535 | 1.939 | 0 | 0 | 0.2 |
| LEE_TCELLS1_UP | 88 | 0.334 | 1.812 | 0 | 0 | 0.2 |
| BREASTCA_THREE_CLASSES | 26 | 0.44 | 1.855 | 0 | 0 | 0.2 |
| GNF2_ST13 | 26 | 0.392 | 1.714 | 0 | 0 | 0.6 |
| RESPONSE_TO_EXTRACELLULAR_STIMULUS | 18 | 0.416 | 1.747 | 0 | 0 | 0.4 |
| SEQUENCE_SPECIFIC_DNA_BINDING | 23 | 0.542 | 1.751 | 0 | 0 | 0.4 |
| GNF2_MSH2 | 22 | 0.675 | 1.752 | 0 | 0 | 0.4 |
| MORF_BUB1B | 46 | 0.529 | 1.752 | 0 | 0 | 0.4 |
| MODULE_525 | 30 | 0.502 | 1.763 | 0 | 0 | 0.4 |
| MODULE_337 | 32 | 0.558 | 1.763 | 0 | 0 | 0.4 |
| GNF2_DENR | 26 | 0.522 | 1.77 | 0 | 0 | 0.4 |
| GNF2_XRCC5 | 55 | 0.552 | 1.771 | 0 | 0 | 0.4 |
| DNA_INTEGRITY_CHECKPOINT | 15 | 0.677 | 1.776 | 0 | 0 | 0.4 |
| TRANSLATION_REGULATOR_ACTIVITY | 18 | 0.568 | 1.778 | 0 | 0 | 0.4 |
| GCM_PSME1 | 57 | 0.419 | 1.779 | 0 | 0 | 0.4 |
| GCM_RAD21 | 27 | 0.522 | 1.782 | 0 | 0 | 0.4 |
| CARBOXYLIC_ACID_TRANSPORT | 18 | 0.478 | 1.789 | 0 | 0 | 0.4 |
| MODULE_192 | 53 | -0.379 | -1.743 | 0 | 0 | 0.4 |
| GNF2_ICAM3 | 19 | -0.452 | -1.798 | 0 | 0 | 0.4 |
| MODULE_108 | 28 | -0.292 | -1.711 | 0 | 0 | 0.6 |
| MODULE_116 | 22 | -0.5 | -1.711 | 0 | 0 | 0.6 |
| MODULE_28 | 27 | -0.528 | -1.705 | 0 | 0 | 0.6 |
| AS3_FIBRO_C3 | 27 | -0.41 | -1.78 | 0 | 0 | 0.4 |
| BRG1_SW13_UP | 30 | -0.501 | -1.799 | 0 | 0 | 0.4 |
| DAC_PANC50_UP | 22 | -0.443 | -1.782 | 0 | 0 | 0.4 |
| FSH_GRANULOSA_UP | 50 | -0.397 | -1.789 | 0 | 0 | 0.4 |
| GCM_RAB10 | 79 | 0.342 | 1.71 | 0 | 0 | 0.6 |
| M_PHASE | 64 | 0.377 | 1.716 | 0 | 0 | 0.6 |
| DNA_REPAIR | 73 | 0.378 | 1.719 | 0 | 0 | 0.6 |
| MORF_DEAF1 | 34 | 0.417 | 1.72 | 0 | 0 | 0.6 |
| MORF_AATF | 125 | 0.453 | 1.728 | 0 | 0 | 0.6 |
| GNF2_RRM2 | 35 | 0.491 | 1.728 | 0 | 0 | 0.6 |
| GCM_HDAC1 | 28 | 0.539 | 1.73 | 0 | 0 | 0.6 |
| CELL_CYCLE_PROCESS | 114 | 0.375 | 1.732 | 0 | 0 | 0.6 |
| METHYLTRANSFERASE_ACTIVITY | 21 | 0.594 | 1.742 | 0 | 0 | 0.6 |
| MORF_RFC1 | 48 | 0.391 | 1.744 | 0 | 0 | 0.6 |
| MODULE_73 | 15 | -0.56 | -1.679 | 0 | 0 | 0.8 |
| AS3_FIBRO_C1 | 18 | -0.606 | -1.72 | 0 | 0.001 | 0.6 |
| DNA_RECOMBINATION | 26 | 0.526 | 1.689 | 0 | 0.001 | 0.6 |
| HYPOPHYSECTOMY_RAT_UP | 22 | -0.593 | -1.719 | 0 | 0.001 | 0.6 |
| GCM_CBFB | 53 | 0.482 | 1.705 | 0 | 0.001 | 0.6 |
| MORF_SOD1 | 192 | 0.34 | 1.706 | 0 | 0.001 | 0.6 |
| SERINE_TYPE_ENDOPEPTIDASE_ACTIVITY | 15 | 0.561 | 1.707 | 0 | 0.001 | 0.6 |
| MODULE_22 | 33 | -0.457 | -1.634 | 0 | 0.001 | 0.8 |
| MORF_CDK2 | 47 | 0.412 | 1.656 | 0 | 0.001 | 0.6 |
| MORF_ANP32B | 127 | 0.35 | 1.658 | 0 | 0.001 | 0.6 |
| MODULE_206 | 65 | 0.354 | 1.664 | 0 | 0.001 | 0.6 |
| ENDOPEPTIDASE_ACTIVITY | 54 | 0.454 | 1.666 | 0 | 0.001 | 0.6 |
| GNF2_SMC1L1 | 17 | 0.598 | 1.666 | 0 | 0.001 | 0.6 |
| PEPTIDASE_ACTIVITY | 77 | 0.368 | 1.667 | 0 | 0.001 | 0.6 |
| GCM_DDX5 | 50 | 0.441 | 1.674 | 0 | 0.001 | 0.6 |
| MODULE_567 | 42 | 0.396 | 1.684 | 0 | 0.001 | 0.6 |
| RNA_METABOLIC_PROCESS | 407 | 0.38 | 1.685 | 0 | 0.001 | 0.6 |
| GNF2_CDC2 | 51 | 0.473 | 1.696 | 0 | 0.001 | 0.6 |
| PHOSPHOLIPID_BINDING | 22 | -0.483 | -1.666 | 0 | 0.001 | 0.8 |
| PHOSPHOTRANSFERASE_ACTIVITY__ALCOHOL_ GROUP_AS_ACCEPTOR | 180 | -0.292 | -1.669 | 0 | 0.001 | 0.8 |
| GNF2_BUB1B | 43 | 0.492 | 1.62 | 0 | 0.001 | 0.8 |
| CLATHRIN_COATED_VESICLE | 21 | -0.677 | -1.666 | 0 | 0.001 | 0.8 |
| MODULE_80 | 18 | -0.517 | -1.621 | 0 | 0.001 | 0.8 |
| MODULE_54 | 180 | 0.434 | 1.648 | 0 | 0.001 | 0.8 |
| RIBONUCLEOPROTEIN_COMPLEX_BIOGENESIS _AND_ASSEMBLY | 44 | 0.488 | 1.65 | 0 | 0.001 | 0.8 |
| MODULE_430 | 26 | -0.427 | -1.651 | 0 | 0.001 | 0.8 |
| SECRETION_BY_CELL | 50 | -0.491 | -1.655 | 0 | 0.001 | 0.8 |
| MODULE_242 | 59 | -0.385 | -1.661 | 0 | 0.001 | 0.8 |
| MODULE_197 | 94 | 0.374 | 1.595 | 0 | 0.001 | 0.8 |
| ROS_MOUSE_AORTA_DN | 56 | -0.431 | -1.662 | 0 | 0.001 | 0.8 |
| MODULE_426 | 36 | -0.349 | -1.641 | 0 | 0.001 | 0.8 |
| MORF_GNB1 | 196 | 0.348 | 1.64 | 0 | 0.001 | 0.8 |
| DOUBLE_STRANDED_DNA_BINDING | 18 | 0.656 | 1.642 | 0 | 0.001 | 0.8 |
| GCM_PTPRU | 19 | -0.36 | -1.607 | 0 | 0.001 | 0.8 |
| MORF_CCNF | 44 | 0.481 | 1.626 | 0 | 0.001 | 0.8 |
| MODULE_18 | 265 | 0.326 | 1.628 | 0 | 0.001 | 0.8 |
| DNA_BINDING | 257 | 0.324 | 1.628 | 0 | 0.001 | 0.8 |
| MODULE_332 | 22 | 0.45 | 1.629 | 0 | 0.001 | 0.8 |
| GH_EXOGENOUS_ANY_DN | 35 | -0.436 | -1.632 | 0 | 0.002 | 0.8 |
| MODULE_137 | 283 | -0.319 | -1.594 | 0 | 0.002 | 0.8 |
| TRANSLATION_FACTOR_ACTIVITY__NUCLEIC_ ACID_BINDING | 18 | 0.568 | 1.617 | 0 | 0.002 | 0.8 |
| MORF_UBE2I | 146 | 0.298 | 1.61 | 0 | 0.002 | 0.8 |
| MODULE_124 | 60 | 0.426 | 1.611 | 0 | 0.002 | 0.8 |
| GCM_USP6 | 22 | -0.384 | -1.586 | 0 | 0.002 | 0.8 |
| VOLTAGE_GATED_CATION_CHANNEL_ACTIVITY | 24 | 0.296 | 1.605 | 0 | 0.002 | 0.8 |
| GNF2_SPTA1 | 28 | 0.299 | 1.559 | 0 | 0.002 | 1 |
| NITROGEN_COMPOUND_BIOSYNTHETIC_PROCESS | 20 | -0.359 | -1.617 | 0 | 0.002 | 0.8 |
| NON_MEMBRANE_BOUND_ORGANELLE | 330 | 0.339 | 1.602 | 0 | 0.002 | 0.8 |
| REGULATION_OF_NUCLEOBASE__NUCLEOSIDE__ NUCLEOTIDE_AND_NUCLEIC_ACID_METABOLIC_ PROCESS | 273 | 0.285 | 1.593 | 0 | 0.002 | 0.8 |
| GCM_PFN1 | 35 | 0.36 | 1.529 | 0 | 0.002 | 1 |
| TRANSMEMBRANE_RECEPTOR_PROTEIN_TYROSINE_ KINASE_SIGNALING_PATHWAY | 38 | -0.38 | -1.613 | 0 | 0.002 | 0.8 |
| MODULE_129 | 104 | -0.328 | -1.554 | 0 | 0.002 | 1 |
| GCM_DENR | 29 | 0.372 | 1.579 | 0 | 0.002 | 0.8 |
| GNF2_BUB1 | 24 | 0.593 | 1.586 | 0 | 0.002 | 0.8 |
| MORF_IFNA1 | 70 | -0.327 | -1.604 | 0 | 0.002 | 0.8 |
| MODULE_100 | 282 | -0.32 | -1.543 | 0 | 0.003 | 1 |
| MODULE_220 | 136 | -0.285 | -1.601 | 0 | 0.003 | 0.8 |
| P21_P53_MIDDLE_DN | 19 | 0.608 | 1.791 | 0 | 0.003 | 0.2 |
| GOLDRATH_CELLCYCLE | 28 | 0.518 | 1.8 | 0 | 0.003 | 0.2 |
| UVB_SCC_DN | 52 | 0.36 | 1.809 | 0 | 0.003 | 0.2 |
| MODULE_53 | 212 | 0.347 | 1.566 | 0 | 0.003 | 1 |
| MORF_EIF3S6 | 75 | 0.373 | 1.575 | 0 | 0.003 | 1 |
| GLYCINE_SERINE_AND_THREONINE_METABOLISM | 17 | 0.52 | 1.81 | 0 | 0.003 | 0.2 |
| ET743_SARCOMA_DN | 153 | 0.337 | 1.818 | 0 | 0.003 | 0.2 |
| SERUM_FIBROBLAST_CORE_UP | 122 | 0.467 | 1.834 | 0 | 0.003 | 0.2 |
| TRANSCRIPTION_FACTOR_BINDING | 158 | 0.29 | 1.565 | 0 | 0.003 | 1 |
| BRCA1_OVEREXP_PROSTATE_DN | 41 | 0.427 | 1.838 | 0 | 0.003 | 0.2 |
| MRNA_SPLICING | 30 | 0.617 | 1.853 | 0 | 0.003 | 0.2 |
| HDACI_COLON_CUR48HRS_UP | 40 | 0.438 | 1.857 | 0 | 0.003 | 0.2 |
| TRANSCRIPTION_FACTOR_COMPLEX | 38 | 0.392 | 1.859 | 0 | 0.003 | 0.2 |
| PARK_MSCS_DIFF | 24 | 0.395 | 1.867 | 0 | 0.003 | 0.2 |
| BAF57_BT549_DN | 173 | 0.382 | 1.884 | 0 | 0.003 | 0.2 |
| GCM_BMPR2 | 48 | 0.305 | 1.509 | 0 | 0.003 | 1 |
| CMV_HCMV_TIMECOURSE_6HRS_DN | 28 | 0.392 | 1.891 | 0 | 0.003 | 0.2 |
| UNFOLDED_PROTEIN_BINDING | 22 | 0.348 | 1.557 | 0 | 0.003 | 1 |
| UNDERHILL_PROLIFERATION | 15 | 0.691 | 1.905 | 0 | 0.003 | 0.2 |
| MODULE_284 | 19 | -0.432 | -1.584 | 0 | 0.003 | 0.8 |
| REGULATION_OF_ANATOMICAL_STRUCTURE_ MORPHOGENESIS | 15 | -0.432 | -1.585 | 0 | 0.003 | 0.8 |
| IDX_TSA_UP_CLUSTER5 | 68 | 0.391 | 1.906 | 0 | 0.003 | 0.2 |
| PROTEASE_INHIBITOR_ACTIVITY | 18 | -0.558 | -1.588 | 0 | 0.003 | 0.8 |
| GREENBAUM_E2A_UP | 28 | 0.473 | 1.579 | 0 | 0.003 | 0.8 |
| ET743_SARCOMA_24HRS_DN | 67 | 0.439 | 1.911 | 0 | 0.003 | 0.2 |
| STEROID_METABOLIC_PROCESS | 21 | 0.454 | 1.551 | 0 | 0.003 | 1 |
| ORGANIC_ACID_TRANSMEMBRANE_TRANSPORTER_ ACTIVITY | 19 | 0.492 | 1.552 | 0 | 0.003 | 1 |
| MORF_ACP1 | 133 | 0.366 | 1.553 | 0 | 0.003 | 1 |
| MYC_ONCOGENIC_SIGNATURE | 116 | 0.464 | 1.912 | 0 | 0.003 | 0.2 |
| PENG_RAPAMYCIN_DN | 139 | 0.421 | 1.92 | 0 | 0.003 | 0.2 |
| SINGLE_STRANDED_DNA_BINDING | 24 | 0.591 | 1.545 | 0 | 0.003 | 1 |
| VANTVEER_BREAST_OUTCOME_GOOD_VS_POOR_DN | 45 | 0.468 | 1.924 | 0 | 0.003 | 0.2 |
| MODULE_363 | 35 | -0.375 | -1.511 | 0 | 0.004 | 1 |
| HSC_EARLYPROGENITORS_SHARED | 250 | 0.373 | 1.94 | 0 | 0.004 | 0.2 |
| CELL_CELL_ADHESION | 42 | -0.374 | -1.578 | 0 | 0.004 | 0.8 |
| UVC_XPCS_ALL_DN | 276 | 0.315 | 1.568 | 0 | 0.004 | 1 |
| MODULE_85 | 27 | -0.362 | -1.509 | 0 | 0.004 | 1 |
| TRANSLATION_FACTORS | 33 | 0.549 | 1.952 | 0 | 0.004 | 0.2 |
| MODULE_261 | 50 | 0.376 | 1.541 | 0 | 0.004 | 1 |
| G1PATHWAY | 18 | 0.59 | 1.954 | 0 | 0.004 | 0.2 |
| WANG_MLL_CBP_VS_GMP_DN | 33 | 0.514 | 1.575 | 0 | 0.004 | 1 |
| NUCLEAR_CHROMOSOME_PART | 21 | 0.611 | 1.954 | 0 | 0.004 | 0.2 |
| ST_DIFFERENTIATION_PATHWAY_IN_PC12_CELLS | 24 | -0.456 | -1.605 | 0 | 0.004 | 0.8 |
| MODULE_221 | 19 | -0.462 | -1.495 | 0 | 0.004 | 1 |
| IGF1_NIH3T3_UP | 19 | 0.614 | 1.97 | 0 | 0.004 | 0.2 |
| MODULE_139 | 40 | -0.375 | -1.496 | 0 | 0.004 | 1 |
| NUCLEOPLASM_PART | 109 | 0.441 | 1.977 | 0 | 0.004 | 0.2 |
| MORF_EIF4E | 49 | 0.428 | 1.535 | 0 | 0.004 | 1 |
| IDX_TSA_UP_CLUSTER3 | 78 | 0.513 | 1.98 | 0 | 0.004 | 0.2 |
| SHEPARD_CRASH_AND_BURN_MUT_VS_WT_UP | 87 | 0.419 | 1.988 | 0 | 0.004 | 0.2 |
| UVC_TTD_4HR_DN | 180 | 0.315 | 1.556 | 0 | 0.004 | 1 |
| MORF_RAN | 178 | 0.286 | 1.522 | 0 | 0.004 | 1 |
| GNF2_TYK2 | 21 | 0.552 | 1.524 | 0 | 0.004 | 1 |
| MODULE_11 | 278 | -0.3 | -1.559 | 0 | 0.004 | 1 |
| GNF2_GLTSCR2 | 15 | -0.465 | -1.564 | 0.333 | 0.004 | 1 |
| RESPONSE_TO_OXIDATIVE_STRESS | 25 | -0.416 | -1.568 | 0 | 0.004 | 1 |
| MODULE_208 | 51 | -0.285 | -1.489 | 0 | 0.004 | 1 |
| NUCLEOBASE__NUCLEOSIDE__NUCLEOTIDE_AND_ NUCLEIC_ACID_TRANSPORT | 16 | 0.669 | 1.52 | 0 | 0.004 | 1 |
| MORF_JUND | 30 | -0.324 | -1.482 | 0 | 0.004 | 1 |
| PROTEIN_KINASE_ACTIVITY | 154 | -0.327 | -1.555 | 0 | 0.004 | 1 |
| ORGANELLE_ENVELOPE | 117 | 0.41 | 1.754 | 0 | 0.005 | 0.4 |
| MORF_SART1 | 36 | 0.462 | 1.52 | 0 | 0.005 | 1 |
| INTRACELLULAR_NON_MEMBRANE_BOUND_ ORGANELLE | 330 | 0.339 | 1.757 | 0 | 0.005 | 0.4 |
| G2PATHWAY | 17 | 0.565 | 1.762 | 0 | 0.005 | 0.4 |
| ZHAN_MULTIPLE_MYELOMA_SUBCLASSES_DIFF | 20 | 0.612 | 1.773 | 0 | 0.005 | 0.4 |
| MORF_ERH | 81 | 0.376 | 1.517 | 0 | 0.005 | 1 |
| GNF2_HMMR | 42 | 0.48 | 1.477 | 0 | 0.005 | 1 |
| IDX_TSA_UP_CLUSTER2 | 39 | 0.427 | 1.538 | 0 | 0.005 | 1 |
| HSA00230_PURINE_METABOLISM | 88 | 0.44 | 1.773 | 0 | 0.005 | 0.4 |
| RIBOSOME | 25 | 0.451 | 1.774 | 0 | 0.005 | 0.4 |
| MORF_RAD21 | 110 | 0.326 | 1.473 | 0 | 0.005 | 1 |
| HSC_INTERMEDIATEPROGENITORS_FETAL | 91 | 0.405 | 1.777 | 0 | 0.005 | 0.4 |
| HCC_SURVIVAL_GOOD_VS_POOR_DN | 86 | 0.4 | 1.78 | 0 | 0.005 | 0.4 |
| MODULE_15 | 211 | 0.309 | 1.512 | 0 | 0.005 | 1 |
| NUCLEAR_EXPORT | 19 | 0.582 | 1.513 | 0 | 0.005 | 1 |
| ENVELOPE | 117 | 0.41 | 1.783 | 0 | 0.005 | 0.4 |
| AGED_MOUSE_CORTEX_UP | 23 | 0.451 | 1.536 | 0 | 0.005 | 1 |
| REN_E2F1_TARGETS | 29 | 0.615 | 1.785 | 0 | 0.005 | 0.4 |
| MODULE_207 | 75 | 0.33 | 1.51 | 0 | 0.005 | 1 |
| MODULE_491 | 35 | -0.379 | -1.463 | 0 | 0.005 | 1 |
| GNF2_RPA1 | 20 | 0.722 | 1.508 | 0 | 0.005 | 1 |
| OXIDOREDUCTASE_ACTIVITY__ACTING_ON_NADH_ OR_NADPH | 16 | -0.583 | -1.469 | 0 | 0.005 | 1 |
| ENZYME_INHIBITOR_ACTIVITY | 57 | -0.313 | -1.528 | 0 | 0.006 | 1 |
| MORF_IL9 | 25 | -0.32 | -1.529 | 0 | 0.006 | 1 |
| MORF_CSNK2B | 197 | 0.32 | 1.507 | 0 | 0.006 | 1 |
| HYDROLASE_ACTIVITY__ACTING_ON_GLYCOSYL_ BONDS | 21 | -0.486 | -1.466 | 0 | 0.006 | 1 |
| GNF2_BNIP3L | 28 | -0.361 | -1.459 | 0 | 0.006 | 1 |
| MODULE_436 | 47 | -0.361 | -1.445 | 0 | 0.006 | 1 |
| MORF_RFC5 | 37 | 0.385 | 1.446 | 0 | 0.006 | 1 |
| GNF2_PTPN6 | 25 | 0.384 | 1.498 | 0 | 0.006 | 1 |
| PROTEIN_OLIGOMERIZATION | 17 | -0.469 | -1.515 | 0 | 0.006 | 1 |
| MORF_XPC | 41 | -0.372 | -1.515 | 0 | 0.006 | 1 |
| CMV_HCMV_TIMECOURSE_4HRS_DN | 17 | 0.434 | 1.713 | 0 | 0.006 | 0.6 |
| FERRANDO_MLL_T_ALL_DN | 55 | 0.429 | 1.719 | 0 | 0.006 | 0.6 |
| IDX_TSA_UP_CLUSTER4 | 29 | 0.391 | 1.727 | 0 | 0.007 | 0.6 |
| O6BG_RESIST_MEDULLOBLASTOMA_DN | 28 | 0.468 | 1.74 | 0 | 0.007 | 0.6 |
| CMV_HCMV_TIMECOURSE_20HRS_UP | 38 | 0.367 | 1.743 | 0 | 0.007 | 0.6 |
| CYTOKINE_BINDING | 22 | -0.369 | -1.509 | 0 | 0.007 | 1 |
| GNF2_MMP1 | 20 | -0.357 | -1.511 | 0 | 0.007 | 1 |
| MODULE_3 | 247 | 0.272 | 1.492 | 0 | 0.007 | 1 |
| YU_CMYC_DN | 38 | -0.383 | -1.529 | 0 | 0.007 | 1 |
| MEMBRANE_ORGANIZATION_AND_BIOGENESIS | 61 | -0.375 | -1.504 | 0 | 0.007 | 1 |
| MODULE_352 | 16 | 0.646 | 1.489 | 0 | 0.007 | 1 |
| TRANSPORT_VESICLE | 15 | -0.589 | -1.501 | 0 | 0.007 | 1 |
| CMV_HCMV_6HRS_UP | 15 | 0.438 | 1.5 | 0 | 0.007 | 1 |
| CMV_8HRS_DN | 28 | 0.344 | 1.502 | 0 | 0.008 | 1 |
| LIPID_BIOSYNTHETIC_PROCESS | 46 | 0.393 | 1.48 | 0 | 0.008 | 1 |
| MORF_GMPS | 41 | 0.359 | 1.48 | 0 | 0.008 | 1 |
| REGULATION_OF_GENE_EXPRESSION__EPIGENETIC | 18 | 0.471 | 1.427 | 0.25 | 0.008 | 1 |
| MACROMOLECULAR_COMPLEX_ASSEMBLY | 136 | 0.261 | 1.475 | 0 | 0.008 | 1 |
| CELL_CYCLE_CHECKPOINT_GO_0000075 | 32 | 0.376 | 1.476 | 0.2 | 0.008 | 1 |
| NUCLEOTIDE_METABOLIC_PROCESS | 20 | 0.603 | 1.477 | 0 | 0.008 | 1 |
| REGULATION_OF_TRANSPORT | 26 | -0.37 | -1.433 | 0 | 0.008 | 1 |
| G1_S_TRANSITION_OF_MITOTIC_CELL_CYCLE | 22 | 0.518 | 1.474 | 0 | 0.008 | 1 |
| MODULE_55 | 378 | -0.291 | -1.482 | 0 | 0.008 | 1 |
| ATPASE_ACTIVITY__COUPLED_TO_MOVEMENT_OF_ SUBSTANCES | 25 | -0.375 | -1.482 | 0 | 0.008 | 1 |
| RCC_NL_UP | 303 | 0.326 | 1.703 | 0 | 0.008 | 0.6 |
| GNF2_TTK | 33 | 0.479 | 1.464 | 0 | 0.009 | 1 |
| GCM_RAF1 | 28 | 0.426 | 1.462 | 0 | 0.009 | 1 |
| BRCA_BRCA1_NEG | 76 | -0.283 | -1.491 | 0 | 0.009 | 1 |
| RESPONSE_TO_NUTRIENT_LEVELS | 16 | 0.403 | 1.456 | 0 | 0.009 | 1 |
| MORF_PDPK1 | 30 | 0.363 | 1.452 | 0 | 0.01 | 1 |
| XU_CBP_UP | 18 | 0.525 | 1.485 | 0 | 0.01 | 1 |
| HSC_LATEPROGENITORS_FETAL | 261 | 0.281 | 1.48 | 0 | 0.01 | 1 |
| PHOSPHORIC_MONOESTER_HYDROLASE_ACTIVITY | 66 | -0.35 | -1.47 | 0 | 0.01 | 1 |
| GNATENKO_PLATELET | 23 | -0.589 | -1.832 | 0 | 0.01 | 0.2 |
| MORF_GPX4 | 39 | -0.457 | -1.467 | 0 | 0.01 | 1 |
| PURINE_METABOLISM | 68 | 0.382 | 1.489 | 0 | 0.01 | 1 |
| UVC_XPCS_8HR_DN | 235 | 0.306 | 1.474 | 0 | 0.01 | 1 |
| MITOCHONDRIAL_RESPIRATORY_CHAIN | 16 | -0.553 | -1.836 | 0 | 0.01 | 0.2 |
| GCM_ACTG1 | 83 | 0.35 | 1.39 | 0 | 0.01 | 1 |

**Table S8: Gene Set Enrichment Analysis for genes showing significant change in expression within 8 hours of MYC-ER^TAM^ activation in the suprabasal keratinocytes.**

Gene sets enriched in genes up-regulated (red) or down-regulated (blue) in the suprabasal keratinocytes within 8 hours following activation of MYC-ER^TAM^. Gene sets are taken from the Molecular Signature Database and from published datasets, and only those with FDR < 0.01 are shown. Size = The number of genes in the gene set that are also in the expression data set; ES = Enrichment score; NES = Normalized enrichment score; NOM p-value = Nominal p-value; FDR q-value = False discovery rate; FWER p-value = Family-wise error rate.

| **Gene Set** | **Size** | **ES** | **NES** | **NOM**  **p-val** | **FDR**  **q-val** | **FWER**  **p-val** |
| --- | --- | --- | --- | --- | --- | --- |
| IDX_TSA_UP_CLUSTER4 | 29 | 0.58 | 3.25 | 0 | 0 | 0 |
| GENOTOXINS_ALL_24HRS_REG | 18 | 0.709 | 2.927 | 0 | 0 | 0 |
| GNF2_APEX1 | 66 | 0.516 | 2.836 | 0 | 0 | 0 |
| GLYCOLYSIS_AND_GLUCONEOGENESIS | 26 | 0.58 | 2.642 | 0 | 0 | 0 |
| ADIP_VS_FIBRO_DN | 19 | 0.663 | 2.641 | 0 | 0 | 0 |
| NADLER_OBESITY_UP | 45 | 0.624 | 2.627 | 0 | 0 | 0 |
| H2O2_CSBRESCUED_UP | 34 | 0.596 | 2.58 | 0 | 0 | 0 |
| GNF2_NPM1 | 41 | 0.586 | 2.558 | 0 | 0 | 0 |
| MODULE_248 | 37 | 0.468 | 2.491 | 0 | 0 | 0 |
| GNF2_PTX3 | 26 | 0.495 | 2.488 | 0 | 0 | 0 |
| GCM_ANP32B | 23 | 0.497 | 2.472 | 0 | 0 | 0 |
| WANG_MLL_CBP_VS_GMP_DN | 33 | 0.573 | 2.462 | 0 | 0 | 0 |
| MORF_PPP6C | 54 | 0.521 | 2.452 | 0 | 0 | 0 |
| MODULE_86 | 26 | 0.533 | 2.452 | 0 | 0 | 0 |
| MODULE_62 | 64 | 0.54 | 2.448 | 0 | 0 | 0 |
| MYOD_BRG1_UP | 18 | 0.63 | 2.436 | 0 | 0 | 0 |
| MODULE_114 | 188 | 0.466 | 2.418 | 0 | 0 | 0 |
| TNFALPHA_4HRS_UP | 26 | 0.479 | 2.406 | 0 | 0 | 0 |
| MODULE_32 | 135 | 0.43 | 2.396 | 0 | 0 | 0 |
| PENG_GLUTAMINE_DN | 190 | 0.45 | 2.379 | 0 | 0 | 0 |
| GNF2_GLTSCR2 | 15 | 0.708 | 2.373 | 0 | 0 | 0 |
| ELECTRON_CARRIER_ACTIVITY | 40 | 0.505 | 2.362 | 0 | 0 | 0 |
| MITOCHONDRIA | 246 | 0.466 | 2.352 | 0 | 0 | 0 |
| MODULE_291 | 41 | 0.506 | 2.351 | 0 | 0 | 0 |
| HESS_HOXAANMEIS1_UP | 52 | 0.451 | 2.341 | 0 | 0 | 0 |
| MORF_EIF3S2 | 166 | 0.46 | 2.322 | 0 | 0 | 0 |
| MODULE_151 | 175 | 0.468 | 2.317 | 0 | 0 | 0 |
| MORF_FBL | 103 | 0.505 | 2.282 | 0 | 0 | 0 |
| ELECTRON_TRANSPORT_CHAIN | 63 | 0.55 | 2.276 | 0 | 0 | 0 |
| MORF_PSMC1 | 109 | 0.477 | 2.274 | 0 | 0 | 0 |
| PENG_RAPAMYCIN_DN | 139 | 0.482 | 2.272 | 0 | 0 | 0 |
| MORF_HDAC1 | 157 | 0.456 | 2.264 | 0 | 0 | 0 |
| VEGF_MMMEC_12HRS_UP | 24 | 0.473 | 2.258 | 0 | 0 | 0 |
| MORF_XRCC5 | 153 | 0.414 | 2.252 | 0 | 0 | 0 |
| MORF_HAT1 | 117 | 0.411 | 2.234 | 0 | 0 | 0 |
| ANTI_APOPTOSIS | 70 | 0.53 | 2.226 | 0 | 0 | 0 |
| MODULE_152 | 86 | 0.554 | 2.221 | 0 | 0 | 0 |
| MORF_ERH | 81 | 0.493 | 2.209 | 0 | 0 | 0 |
| MITOCHONDRIAL_MEMBRANE_PART | 36 | 0.548 | 2.203 | 0 | 0 | 0 |
| MORF_AP2M1 | 130 | 0.466 | 2.195 | 0 | 0 | 0 |
| GCM_NPM1 | 69 | 0.505 | 2.189 | 0 | 0 | 0 |
| MORF_NPM1 | 91 | 0.474 | 2.178 | 0 | 0 | 0 |
| NEGATIVE_REGULATION_OF_APOPTOSIS | 85 | 0.477 | 2.171 | 0 | 0 | 0 |
| GNF2_FBL | 72 | 0.528 | 2.17 | 0 | 0 | 0 |
| MORF_BUB3 | 190 | 0.439 | 2.17 | 0 | 0 | 0 |
| WERNER_FIBRO_UP | 33 | 0.566 | 2.169 | 0 | 0 | 0 |
| CELLULAR_CATABOLIC_PROCESS | 104 | 0.378 | 2.145 | 0 | 0 | 0 |
| BYSTRYKH_HSC_BRAIN_CIS_GLOCUS | 54 | 0.386 | 2.139 | 0 | 0 | 0 |
| NING_COPD_UP | 98 | 0.468 | 2.138 | 0 | 0 | 0 |
| MODULE_306 | 19 | 0.617 | 2.131 | 0 | 0 | 0 |
| TARTE_PC | 46 | 0.502 | 2.118 | 0 | 0 | 0 |
| GNF2_BUB3 | 17 | 0.495 | 2.114 | 0 | 0 | 0 |
| OXIDATIVE_PHOSPHORYLATION | 43 | 0.447 | 2.111 | 0 | 0 | 0 |
| GNF2_G22P1 | 25 | 0.63 | 2.107 | 0 | 0 | 0 |
| GNF2_MMP1 | 20 | 0.611 | 2.103 | 0 | 0 | 0 |
| SERUM_FIBROBLAST_CORE_UP | 122 | 0.394 | 2.099 | 0 | 0 | 0 |
| ADIP_VS_PREADIP_DN | 26 | 0.635 | 2.098 | 0 | 0 | 0 |
| SERINE_TYPE_PEPTIDASE_ACTIVITY | 18 | 0.559 | 2.096 | 0 | 0 | 0 |
| IGLESIAS_E2FMINUS_UP | 116 | 0.471 | 2.091 | 0 | 0 | 0 |
| GCM_ACTG1 | 83 | 0.445 | 2.09 | 0 | 0 | 0 |
| MODULE_219 | 23 | 0.529 | 2.088 | 0 | 0 | 0 |
| BREASTCA_THREE_CLASSES | 26 | 0.594 | 2.075 | 0 | 0 | 0 |
| MORF_HDAC2 | 175 | 0.375 | 2.074 | 0 | 0 | 0 |
| MORF_AP3D1 | 74 | 0.507 | 2.07 | 0 | 0 | 0 |
| MITOCHONDRIAL_ENVELOPE | 61 | 0.552 | 2.07 | 0 | 0 | 0 |
| PHOSPHOINOSITIDE_METABOLIC_PROCESS | 15 | 0.619 | 2.068 | 0 | 0 | 0 |
| MITOCHONDRIAL_INNER_MEMBRANE | 42 | 0.585 | 2.063 | 0 | 0 | 0 |
| MODULE_42 | 18 | 0.576 | 2.06 | 0 | 0 | 0 |
| SHIPP_FL_VS_DLBCL_DN | 28 | 0.477 | 2.057 | 0 | 0 | 0 |
| PARK_MSCS_DIFF | 24 | 0.475 | 2.057 | 0 | 0 | 0 |
| MODULE_233 | 16 | 0.573 | 2.056 | 0 | 0 | 0 |
| MORF_DAP3 | 125 | 0.442 | 2.052 | 0 | 0 | 0 |
| RESPONSE_TO_OXIDATIVE_STRESS | 25 | 0.515 | 2.051 | 0 | 0 | 0 |
| G_PROTEIN_COUPLED_RECEPTOR_BINDING | 22 | 0.527 | 2.044 | 0 | 0 | 0 |
| GNF2_ST13 | 26 | 0.592 | 2.04 | 0 | 0 | 0 |
| MORF_NME2 | 88 | 0.484 | 2.036 | 0 | 0 | 0 |
| HSA00190_OXIDATIVE_PHOSPHORYLATION | 75 | 0.44 | 2.034 | 0 | 0 | 0 |
| MITOCHONDRION | 190 | 0.429 | 2.03 | 0 | 0 | 0 |
| MORF_CTBP1 | 94 | 0.444 | 2.025 | 0 | 0 | 0 |
| MORF_ATOX1 | 50 | 0.51 | 2.025 | 0 | 0 | 0 |
| MORF_CSNK2B | 197 | 0.417 | 2.012 | 0 | 0 | 0 |
| MORF_UNG | 57 | 0.556 | 2.01 | 0 | 0 | 0 |
| MORF_MAP2K2 | 88 | 0.477 | 2.007 | 0 | 0 | 0 |
| UVB_NHEK2_UP | 47 | 0.467 | 1.996 | 0 | 0 | 0 |
| UVB_SCC_UP | 62 | 0.442 | 1.995 | 0 | 0 | 0 |
| GNF2_CASP1 | 54 | 0.345 | 1.993 | 0 | 0 | 0 |
| MORF_G22P1 | 118 | 0.462 | 1.991 | 0 | 0 | 0 |
| GNF2_CD53 | 31 | 0.504 | 1.988 | 0 | 0 | 0 |
| NEGATIVE_REGULATION_OF_DEVELOPMENTAL_PROCESS | 106 | 0.407 | 1.988 | 0 | 0 | 0 |
| CALRES_MOUSE_NEOCORTEX_DN | 43 | 0.439 | 1.975 | 0 | 0 | 0 |
| GNF2_TST | 29 | 0.373 | 1.974 | 0 | 0 | 0 |
| PENG_LEUCINE_DN | 104 | 0.393 | 1.971 | 0 | 0 | 0 |
| ZUCCHI_EPITHELIAL_DN | 23 | 0.428 | 1.97 | 0 | 0 | 0 |
| GO_ROS | 18 | 0.504 | 1.966 | 0 | 0 | 0 |
| NUCLEOTIDE_METABOLIC_PROCESS | 20 | 0.417 | 1.966 | 0 | 0 | 0 |
| YAGI_AML_PROGNOSIS | 26 | 0.461 | 1.964 | 0 | 0 | 0 |
| MORF_EI24 | 100 | 0.46 | 1.964 | 0 | 0 | 0 |
| GLYCEROPHOSPHOLIPID_METABOLIC_PROCESS | 21 | 0.543 | 1.955 | 0 | 0 | 0 |
| HSA00530_AMINOSUGARS_METABOLISM | 17 | 0.646 | 1.952 | 0 | 0 | 0 |
| GCM_CSNK2B | 58 | 0.465 | 1.951 | 0 | 0 | 0 |
| MODULE_278 | 28 | 0.512 | 1.949 | 0 | 0 | 0 |
| MORF_PPP1CA | 101 | 0.434 | 1.948 | 0 | 0 | 0 |
| ORGANELLE_MEMBRANE | 185 | 0.347 | 1.946 | 0 | 0 | 0 |
| APPEL_IMATINIB_UP | 26 | 0.465 | 1.944 | 0 | 0 | 0 |
| MODULE_22 | 33 | 0.617 | 1.943 | 0 | 0 | 0 |
| SANA_IFNG_ENDOTHELIAL_DN | 59 | 0.502 | 1.942 | 0 | 0 | 0 |
| VENTRICLES_UP | 121 | 0.378 | 1.94 | 0 | 0 | 0 |
| BASSO_REGULATORY_HUBS | 104 | 0.407 | 1.94 | 0 | 0 | 0 |
| MORF_ANP32B | 127 | 0.481 | 1.934 | 0 | 0 | 0 |
| COLLER_MYC_UP | 15 | 0.627 | 1.933 | 0 | 0 | 0 |
| MORF_EIF4A2 | 72 | 0.412 | 1.933 | 0 | 0 | 0 |
| GNF2_FGR | 20 | 0.6 | 1.93 | 0 | 0 | 0 |
| MORF_PSMC2 | 68 | 0.455 | 1.929 | 0 | 0 | 0 |
| RIBOSOME | 25 | 0.527 | 1.927 | 0 | 0 | 0 |
| LYASE_ACTIVITY | 30 | 0.442 | 1.926 | 0 | 0 | 0 |
| MODULE_79 | 60 | 0.448 | 1.925 | 0 | 0 | 0 |
| MODULE_174 | 53 | 0.519 | 1.925 | 0 | 0 | 0 |
| MYOD_NIH3T3_UP | 46 | 0.429 | 1.922 | 0 | 0 | 0 |
| MORF_DEAF1 | 34 | 0.536 | 1.919 | 0 | 0 | 0 |
| NEGATIVE_REGULATION_OF_PROGRAMMED_CELL_DEATH | 86 | 0.473 | 1.918 | 0 | 0 | 0 |
| MODULE_116 | 22 | 0.58 | 1.914 | 0 | 0 | 0 |
| MORF_DDB1 | 136 | 0.391 | 1.913 | 0 | 0 | 0 |
| STRUCTURAL_CONSTITUENT_OF_RIBOSOME | 36 | 0.457 | 1.912 | 0 | 0 | 0 |
| MORF_DEK | 163 | 0.408 | 1.908 | 0 | 0 | 0 |
| CALRES_MOUSE_UP | 19 | 0.507 | 1.902 | 0 | 0 | 0 |
| RIBONUCLEOPROTEIN_COMPLEX_BIOGENESIS_AND_ ASSEMBLY | 44 | 0.492 | 1.901 | 0 | 0 | 0 |
| BADPATHWAY | 16 | 0.629 | 1.901 | 0 | 0 | 0 |
| MORF_ACTG1 | 69 | 0.448 | 1.899 | 0 | 0 | 0 |
| CHEN_HOXA5_TARGETS_DN | 31 | 0.486 | 1.89 | 0 | 0 | 0 |
| MEMBRANE_LIPID_METABOLIC_PROCESS | 51 | 0.453 | 1.89 | 0 | 0 | 0 |
| RACCYCDPATHWAY | 17 | 0.507 | 1.889 | 0 | 0 | 0 |
| ROS_MOUSE_AORTA_UP | 17 | 0.484 | 1.888 | 0 | 0 | 0 |
| BRCA1_OVEREXP_DN | 74 | 0.48 | 1.885 | 0 | 0 | 0 |
| MODULE_159 | 47 | 0.493 | 1.885 | 0 | 0 | 0 |
| CANTHARIDIN_DN | 37 | 0.528 | 1.884 | 0 | 0 | 0 |
| GILDEA_BLADDER_UP | 18 | 0.573 | 1.882 | 0 | 0 | 0 |
| NUCLEOLUS | 64 | 0.499 | 1.879 | 0 | 0 | 0 |
| MODULE_83 | 167 | 0.483 | 1.877 | 0 | 0 | 0 |
| MORF_MBD4 | 46 | 0.481 | 1.875 | 0 | 0 | 0 |
| MITOCHONDRIAL_RESPIRATORY_CHAIN | 16 | 0.448 | 1.873 | 0 | 0 | 0 |
| MODULE_170 | 59 | 0.438 | 1.871 | 0 | 0 | 0 |
| HSA05040_HUNTINGTONS_DISEASE | 15 | 0.659 | 1.87 | 0 | 0 | 0 |
| GNF2_MCM5 | 45 | 0.443 | 1.866 | 0 | 0 | 0 |
| HDACI_COLON_SUL48HRS_DN | 40 | 0.397 | 1.866 | 0 | 0 | 0 |
| MODULE_77 | 20 | 0.56 | 1.863 | 0 | 0 | 0 |
| AGED_MOUSE_MUSCLE_DN | 19 | 0.548 | 1.863 | 0 | 0 | 0 |
| TAKEDA_NUP8_HOXA9_6H_DN | 18 | 0.45 | 1.859 | 0 | 0 | 0 |
| GNF2_NS | 33 | 0.502 | 1.857 | 0 | 0 | 0 |
| PENG_LEUCINE_UP | 57 | 0.448 | 1.857 | 0 | 0 | 0 |
| RUTELLA_HEPATGFSNDCS_UP | 93 | 0.406 | 1.855 | 0 | 0 | 0 |
| SERINE_HYDROLASE_ACTIVITY | 18 | 0.559 | 1.849 | 0 | 0 | 0 |
| PGC | 238 | 0.401 | 1.849 | 0 | 0 | 0 |
| MODULE_307 | 17 | 0.568 | 1.848 | 0 | 0 | 0 |
| MORF_CCNI | 50 | 0.43 | 1.843 | 0 | 0 | 0 |
| HSA00251_GLUTAMATE_METABOLISM | 17 | 0.498 | 1.842 | 0 | 0 | 0 |
| GNF2_PA2G4 | 60 | 0.466 | 1.841 | 0 | 0 | 0 |
| YANG_OSTECLASTS_SIG | 22 | 0.572 | 1.84 | 0 | 0 | 0 |
| HSA05010_ALZHEIMERS_DISEASE | 17 | 0.545 | 1.839 | 0 | 0 | 0 |
| MODULE_245 | 18 | 0.606 | 1.837 | 0 | 0 | 0 |
| NO1PATHWAY | 20 | 0.534 | 1.834 | 0 | 0 | 0 |
| COENZYME_METABOLIC_PROCESS | 25 | 0.402 | 1.834 | 0 | 0 | 0 |
| UBIQUITIN_MEDIATED_PROTEOLYSIS | 19 | 0.39 | 1.833 | 0 | 0 | 0 |
| CANCER_NEOPLASTIC_META_UP | 47 | 0.465 | 1.831 | 0 | 0 | 0 |
| HSA05223_NON_SMALL_CELL_LUNG_CANCER | 28 | 0.464 | 1.829 | 0 | 0 | 0 |
| NING_COPD_DN | 80 | 0.415 | 1.825 | 0 | 0 | 0 |
| HDACI_COLON_BUT30MIN_DN | 18 | 0.495 | 1.82 | 0 | 0 | 0 |
| MODULE_28 | 27 | 0.452 | 1.819 | 0 | 0 | 0 |
| MORF_PTPN9 | 22 | 0.534 | 1.818 | 0 | 0 | 0 |
| CITED1_KO_HET_UP | 15 | 0.545 | 1.816 | 0 | 0 | 0 |
| STRESS_ARSENIC_SPECIFIC_DN | 16 | 0.443 | 1.814 | 0 | 0 | 0 |
| GUANYL_NUCLEOTIDE_BINDING | 22 | 0.536 | 1.813 | 0 | 0 | 0 |
| TRANSLATION_FACTORS | 33 | 0.528 | 1.805 | 0 | 0 | 0 |
| GNF2_UBE2I | 22 | 0.532 | 1.805 | 0 | 0 | 0 |
| RNA_SPLICING | 53 | 0.414 | 1.802 | 0 | 0 | 0 |
| REGULATION_OF_PROGRAMMED_CELL_DEATH | 168 | 0.347 | 1.801 | 0 | 0 | 0 |
| MITOCHONDRIAL_MATRIX | 33 | 0.496 | 1.801 | 0 | 0 | 0 |
| MORF_AATF | 125 | 0.4 | 1.798 | 0 | 0 | 0 |
| HSA00051_FRUCTOSE_AND_MANNOSE_METABOLISM | 25 | 0.512 | 1.798 | 0 | 0 | 0 |
| GCM_TPT1 | 30 | 0.482 | 1.793 | 0 | 0 | 0 |
| HSC_INTERMEDIATEPROGENITORS_ADULT | 81 | 0.418 | 1.791 | 0 | 0 | 0 |
| GNF2_XRCC5 | 55 | 0.478 | 1.791 | 0 | 0 | 0 |
| MODULE_355 | 19 | 0.563 | 1.79 | 0 | 0 | 0 |
| MORF_BAG5 | 30 | 0.49 | 1.786 | 0 | 0 | 0 |
| MORF_GPX4 | 39 | 0.509 | 1.784 | 0 | 0 | 0 |
| MORF_PPP2R4 | 34 | 0.528 | 1.783 | 0 | 0 | 0 |
| GNF2_CD48 | 20 | 0.553 | 1.782 | 0 | 0 | 0 |
| AMINE_METABOLIC_PROCESS | 78 | 0.393 | 1.779 | 0 | 0 | 0 |
| MORF_ACP1 | 133 | 0.397 | 1.777 | 0 | 0 | 0 |
| MITOCHONDRIAL_RIBOSOME | 18 | 0.506 | 1.776 | 0 | 0 | 0 |
| MODULE_60 | 217 | 0.391 | 1.776 | 0 | 0 | 0 |
| MITOCHONDRIAL_LUMEN | 33 | 0.496 | 1.776 | 0 | 0 | 0 |
| MODULE_155 | 18 | 0.606 | 1.766 | 0 | 0 | 0 |
| HDACI_COLON_CUR48HRS_UP | 40 | 0.457 | 1.764 | 0 | 0 | 0 |
| MODULE_1 | 225 | 0.338 | 1.763 | 0 | 0 | 0 |
| MODULE_183 | 42 | 0.415 | 1.763 | 0 | 0 | 0 |
| MRNA_PROCESSING_GO_0006397 | 47 | 0.411 | 1.76 | 0 | 0 | 0 |
| HSA04910_INSULIN_SIGNALING_PATHWAY | 76 | 0.41 | 1.759 | 0 | 0 | 0 |
| AMINO_ACID_AND_DERIVATIVE_METABOLIC_PROCESS | 61 | 0.379 | 1.756 | 0 | 0 | 0 |
| ICHIBA_GVHD | 204 | 0.325 | 1.754 | 0 | 0 | 0 |
| HOHENKIRK_MONOCYTE_DEND_DN | 62 | 0.44 | 1.754 | 0 | 0 | 0 |
| MORF_XPC | 41 | 0.508 | 1.751 | 0 | 0 | 0 |
| BREASTCA_TWO_CLASSES | 90 | 0.358 | 1.751 | 0 | 0 | 0 |
| GCM_CSNK1D | 16 | 0.546 | 1.751 | 0 | 0 | 0 |
| MORF_PCNA | 64 | 0.446 | 1.746 | 0 | 0 | 0 |
| KANNAN_P53_UP | 19 | 0.439 | 1.745 | 0 | 0 | 0 |
| BLEO_MOUSE_LYMPH_LOW_24HRS_DN | 17 | 0.547 | 1.744 | 0 | 0 | 0 |
| CALCIUM_REGULATION_IN_CARDIAC_CELLS | 77 | 0.278 | 1.744 | 0 | 0 | 0 |
| MODULE_46 | 186 | 0.322 | 1.742 | 0 | 0 | 0 |
| UVC_TTD_4HR_UP | 38 | 0.379 | 1.742 | 0 | 0 | 0 |
| ET743_SARCOMA_6HRS_UP | 22 | 0.516 | 1.739 | 0 | 0 | 0 |
| MODULE_241 | 43 | 0.497 | 1.738 | 0 | 0 | 0 |
| MODULE_82 | 16 | 0.461 | 1.738 | 0 | 0 | 0 |
| NUCLEAR_ENVELOPE_ENDOPLASMIC_RETICULUM_ NETWORK | 54 | 0.364 | 1.737 | 0 | 0 | 0 |
| HIPPOCAMPUS_DEVELOPMENT_POSTNATAL | 31 | 0.532 | 1.735 | 0 | 0 | 0 |
| HADDAD_HSC_CD7_DN | 46 | -0.373 | -1.848 | 0 | 0 | 0 |
| GOLDRATH_CELLCYCLE | 28 | -0.547 | -1.855 | 0 | 0 | 0 |
| TOLLPATHWAY | 20 | -0.452 | -1.863 | 0 | 0 | 0 |
| CROONQUIST_IL6_STARVE_UP | 31 | -0.468 | -1.864 | 0 | 0 | 0 |
| SHEPARD_BMYB_MORPHOLINO_DN | 102 | -0.304 | -1.902 | 0 | 0 | 0 |
| METAL_ION_TRANSPORT | 36 | -0.431 | -1.939 | 0 | 0 | 0 |
| HSA00100_BIOSYNTHESIS_OF_STEROIDS | 16 | -0.47 | -1.939 | 0 | 0 | 0 |
| NELSON_ANDROGEN_UP | 41 | -0.375 | -1.948 | 0 | 0 | 0 |
| GENOTOXINS_ALL_4HRS_REG | 22 | -0.433 | -1.996 | 0 | 0 | 0 |
| CROONQUIST_IL6_RAS_DN | 18 | -0.503 | -2.032 | 0 | 0 | 0 |
| REGULATION_OF_ORGANELLE_ORGANIZATION_AND_ BIOGENESIS | 20 | -0.477 | -2.045 | 0 | 0 | 0 |
| HSA00590_ARACHIDONIC_ACID_METABOLISM | 26 | -0.378 | -2.062 | 0 | 0 | 0 |
| G2PATHWAY | 17 | -0.308 | -2.062 | 0 | 0 | 0 |
| NEGATIVE_REGULATION_OF_CELL_CYCLE | 46 | -0.439 | -2.091 | 0 | 0 | 0 |
| LEE_TCELLS3_UP | 61 | -0.487 | -2.258 | 0 | 0 | 0 |
| RECEPTOR_SIGNALING_PROTEIN_SERINE_THREONINE_ KINASE_ACTIVITY | 18 | -0.668 | -2.26 | 0 | 0 | 0 |
| ADIP_DIFF_CLUSTER5 | 31 | -0.452 | -2.29 | 0 | 0 | 0 |
| ALKPATHWAY | 17 | -0.552 | -2.343 | 0 | 0 | 0 |
| STOSSI_ER_UP | 22 | -0.499 | -2.35 | 0 | 0 | 0 |
| DOX_RESIST_GASTRIC_UP | 28 | -0.586 | -2.618 | 0 | 0 | 0 |
| CAMPTOTHECIN_PROBCELL_UP | 15 | 0.439 | 2.518 | 0 | 0 | 0 |
| ELECTRON_TRANSPORTER_ACTIVITY | 57 | 0.454 | 2.444 | 0 | 0 | 0 |
| IDX_TSA_UP_CLUSTER5 | 68 | 0.529 | 2.434 | 0 | 0 | 0 |
| HUMAN_MITODB_6_2002 | 240 | 0.484 | 2.358 | 0 | 0 | 0 |
| LVAD_HEARTFAILURE_UP | 51 | 0.407 | 2.24 | 0 | 0 | 0 |
| TNFALPHA_ALL_UP | 52 | 0.44 | 2.156 | 0 | 0 | 0 |
| NITROGEN_COMPOUND_METABOLIC_PROCESS | 87 | 0.381 | 1.754 | 0 | 0 | 0 |
| BRG1_ALAB_UP | 20 | 0.564 | 1.733 | 0 | 0 | 0 |
| CHANG_SERUM_RESPONSE_UP | 102 | 0.387 | 1.914 | 0 | 0 | 0 |
| ZELLER_MYC_UP | 17 | 0.6 | 1.811 | 0 | 0 | 0 |
| MOOTHA_VOXPHOS | 56 | 0.534 | 2.165 | 0 | 0 | 0 |
| FLECHNER_KIDNEY_TRANSPLANT_WELL_PBL_UP | 86 | 0.463 | 1.904 | 0 | 0 | 0 |
| LEE_TCELLS9_UP | 15 | -0.593 | -1.978 | 0 | 0 | 0 |
| GREENBAUM_E2A_UP | 28 | -0.599 | -2.198 | 0 | 0 | 0 |
| BRCA1_MES_UP | 25 | 0.451 | 1.918 | 0 | 0 | 0 |
| HYPOPHYSECTOMY_RAT_DN | 29 | 0.525 | 1.762 | 0 | 0 | 0 |
| MITOCHONDRIAL_MEMBRANE | 55 | 0.548 | 2.525 | 0 | 0 | 0 |
| ORGANELLE_INNER_MEMBRANE | 46 | 0.491 | 1.83 | 0 | 0 | 0 |
| RIBONUCLEOPROTEIN_COMPLEX | 85 | 0.385 | 1.812 | 0 | 0 | 0 |
| UBIQUITIN_LIGASE_COMPLEX | 15 | 0.49 | 1.754 | 0 | 0 | 0 |
| PROTEIN_FOLDING | 38 | 0.479 | 1.759 | 0 | 0 | 0 |
| RNA_SPLICING__VIA_TRANSESTERIFICATION_ REACTIONS | 22 | 0.583 | 1.843 | 0 | 0 | 0 |
| MITOSIS | 52 | -0.328 | -1.891 | 0 | 0 | 0 |
| NITROGEN_COMPOUND_BIOSYNTHETIC_PROCESS | 20 | 0.463 | 1.739 | 0 | 0 | 0 |
| RNA_BINDING | 136 | 0.436 | 1.802 | 0 | 0 | 0 |
| TRANSLATION_FACTOR_ACTIVITY__NUCLEIC_ACID_ BINDING | 18 | 0.49 | 1.877 | 0 | 0 | 0 |
| MORF_RPA2 | 98 | 0.423 | 2.286 | 0 | 0 | 0 |
| MORF_RAD23A | 226 | 0.403 | 2.125 | 0 | 0 | 0 |
| MORF_SOD1 | 192 | 0.451 | 2.08 | 0 | 0 | 0 |
| MORF_PRKDC | 117 | 0.403 | 1.959 | 0 | 0 | 0 |
| MORF_RAD21 | 110 | 0.436 | 1.927 | 0 | 0 | 0 |
| GCM_MYST2 | 90 | -0.304 | -1.851 | 0 | 0 | 0 |
| MORF_IL13 | 84 | -0.351 | -1.891 | 0 | 0 | 0 |
| MORF_FLT1 | 33 | -0.495 | -1.922 | 0 | 0 | 0 |
| MORF_ETV3 | 23 | -0.569 | -1.971 | 0 | 0 | 0 |
| MORF_JAG1 | 29 | -0.459 | -2.409 | 0 | 0 | 0 |
| MORF_PRKCA | 63 | -0.366 | -2.488 | 0 | 0 | 0 |
| MORF_CDK2 | 47 | 0.403 | 1.987 | 0 | 0 | 0 |
| MORF_CSNK1D | 34 | 0.422 | 1.758 | 0 | 0 | 0 |
| MORF_RAN | 178 | 0.48 | 2.416 | 0 | 0 | 0 |
| MORF_UBE2A | 28 | 0.439 | 2.224 | 0 | 0 | 0 |
| GCM_PSME1 | 57 | 0.464 | 2.127 | 0 | 0 | 0 |
| MORF_RAB6A | 32 | 0.398 | 2.087 | 0 | 0 | 0 |
| MORF_RAB1A | 119 | 0.437 | 2.072 | 0 | 0 | 0 |
| MORF_UBE2I | 146 | 0.443 | 1.866 | 0 | 0 | 0 |
| MORF_RAC1 | 128 | 0.393 | 1.832 | 0 | 0 | 0 |
| MORF_RAB5A | 56 | 0.454 | 1.798 | 0 | 0 | 0 |
| MORF_SART1 | 36 | 0.541 | 2.055 | 0 | 0 | 0 |
| MORF_SNRP70 | 29 | 0.518 | 1.805 | 0 | 0 | 0 |
| MORF_SKP1A | 117 | 0.436 | 1.733 | 0 | 0 | 0 |
| MORF_RUNX1 | 54 | -0.456 | -1.92 | 0 | 0 | 0 |
| MODULE_102 | 16 | 0.623 | 2.108 | 0 | 0 | 0 |
| MODULE_128 | 58 | 0.443 | 1.906 | 0 | 0 | 0 |
| GNF2_CCNA2 | 55 | -0.447 | -1.843 | 0 | 0 | 0 |
| MODULE_448 | 16 | -0.484 | -1.914 | 0 | 0 | 0 |
| GNF2_CDC20 | 51 | -0.478 | -2.033 | 0 | 0 | 0 |
| GNF2_CCNB2 | 52 | -0.515 | -2.073 | 0 | 0 | 0 |
| GNF2_ESPL1 | 31 | -0.471 | -2.154 | 0 | 0 | 0 |
| GNF2_HMMR | 42 | -0.487 | -2.199 | 0 | 0 | 0 |
| MODULE_433 | 31 | -0.558 | -2.48 | 0 | 0 | 0 |
| MODULE_357 | 34 | -0.588 | -2.518 | 0 | 0 | 0 |
| MODULE_154 | 33 | -0.595 | -2.537 | 0 | 0 | 0 |
| GNF2_TTK | 33 | -0.556 | -3.723 | 0 | 0 | 0 |
| MODULE_80 | 18 | -0.379 | -1.832 | 0 | 0 | 0.2 |
| GNF2_PCNA | 57 | -0.405 | -1.813 | 0 | 0 | 0.2 |
| MORF_NOS2A | 97 | -0.358 | -1.828 | 0 | 0 | 0.2 |
| CATION_TRANSPORT | 46 | -0.371 | -1.813 | 0 | 0 | 0.2 |
| ZHAN_MM_CD138_PR_VS_REST | 29 | -0.523 | -1.817 | 0 | 0 | 0.2 |
| LAL_KO_3MO_UP | 32 | 0.406 | 1.714 | 0 | 0 | 0.2 |
| IDX_TSA_UP_CLUSTER2 | 39 | 0.357 | 1.717 | 0 | 0 | 0.2 |
| CYTOKINE_PRODUCTION | 21 | -0.345 | -1.787 | 0 | 0 | 0.4 |
| MODULE_297 | 34 | -0.588 | -1.77 | 0 | 0 | 0.6 |
| MORF_SUPT3H | 121 | -0.3 | -1.734 | 0 | 0 | 0.6 |
| MORF_FOSL1 | 150 | -0.33 | -1.715 | 0 | 0 | 0.6 |
| MORF_JAK3 | 27 | -0.378 | -1.738 | 0 | 0 | 0.6 |
| MORF_RFC5 | 37 | -0.299 | -1.738 | 0 | 0 | 0.6 |
| GNF2_MKI67 | 21 | -0.536 | -1.76 | 0 | 0 | 0.6 |
| GNF2_RRM2 | 35 | -0.482 | -1.763 | 0 | 0 | 0.6 |
| GNF2_CDC2 | 51 | -0.389 | -1.668 | 0 | 0 | 0.8 |
| MODULE_403 | 34 | -0.41 | -1.697 | 0 | 0 | 0.8 |
| MORF_STK17A | 56 | -0.341 | -1.69 | 0 | 0 | 0.8 |
| NEGATIVE_REGULATION_OF_METABOLIC_PROCESS | 124 | -0.315 | -1.676 | 0 | 0 | 0.8 |
| CELL_CYCLE_GO_0007049 | 180 | -0.276 | -1.699 | 0 | 0 | 0.8 |
| HSC_INTERMEDIATEPROGENITORS_FETAL | 91 | 0.385 | 1.702 | 0 | 0 | 0.4 |
| GH_EXOGENOUS_ANY_DN | 35 | -0.396 | -1.646 | 0 | 0 | 0.8 |
| MORF_SS18 | 27 | 0.47 | 1.691 | 0 | 0 | 0.6 |
| GNF2_S100A4 | 28 | 0.513 | 1.688 | 0 | 0.001 | 0.8 |
| PEPTIDE_BINDING | 30 | 0.428 | 1.687 | 0 | 0.001 | 0.8 |
| P21_P53_ANY_DN | 33 | -0.44 | -1.621 | 0 | 0.001 | 1 |
| GNF2_CENPF | 51 | -0.387 | -1.604 | 0 | 0.001 | 1 |
| GH_EXOGENOUS_LATE_DN | 27 | -0.356 | -1.603 | 0 | 0.001 | 1 |
| MODULE_342 | 89 | -0.302 | -1.586 | 0 | 0.001 | 1 |
| MORF_RAP1A | 65 | -0.296 | -1.583 | 0 | 0.001 | 1 |
| HSC_STHSC_FETAL | 17 | -0.346 | -1.6 | 0 | 0.001 | 1 |
| HSA05214_GLIOMA | 35 | 0.497 | 1.675 | 0 | 0.001 | 0.8 |
| MODULE_100 | 282 | 0.23 | 1.669 | 0 | 0.001 | 0.8 |
| TGF_BETA_SIGNALING_PATHWAY | 28 | 0.273 | 1.712 | 0 | 0.001 | 0.2 |
| MORF_PRKAR1A | 83 | 0.421 | 1.712 | 0 | 0.001 | 0.2 |
| MODULE_273 | 37 | 0.476 | 1.714 | 0 | 0.001 | 0.2 |
| KUMAR_HOXA_DIFF | 251 | 0.322 | 1.714 | 0 | 0.001 | 0.2 |
| AGED_MOUSE_HYPOTH_UP | 27 | 0.46 | 1.715 | 0 | 0.001 | 0.2 |
| MYELOID_CELL_DIFFERENTIATION | 21 | 0.562 | 1.715 | 0 | 0.001 | 0.2 |
| PURINE_METABOLISM | 68 | 0.412 | 1.719 | 0 | 0.001 | 0.2 |
| MODULE_399 | 30 | 0.477 | 1.72 | 0 | 0.001 | 0.2 |
| MORF_PRKAG1 | 128 | 0.368 | 1.723 | 0 | 0.001 | 0.2 |
| MODULE_17 | 233 | 0.307 | 1.726 | 0 | 0.001 | 0.2 |
| MODULE_366 | 15 | -0.428 | -1.566 | 0 | 0.001 | 1 |
| LVAD_HEARTFAILURE_DN | 26 | 0.495 | 1.678 | 0 | 0.001 | 0.8 |
| MORF_RAD51L3 | 139 | -0.306 | -1.563 | 0 | 0.001 | 1 |
| GH_EXOGENOUS_MIDDLE_UP | 41 | -0.376 | -1.577 | 0 | 0.001 | 1 |
| GAMMA_ESR_WS_UNREG | 15 | -0.393 | -1.579 | 0 | 0.001 | 1 |
| GN_CAMP_GRANULOSA_UP | 35 | -0.382 | -1.564 | 0 | 0.001 | 1 |
| MODULE_138 | 37 | 0.379 | 1.643 | 0 | 0.001 | 1 |
| MORF_RAD54L | 68 | 0.392 | 1.638 | 0 | 0.002 | 1 |
| GCM_SUFU | 31 | -0.499 | -1.537 | 0 | 0.002 | 1 |
| MODULE_163 | 192 | -0.286 | -1.524 | 0 | 0.002 | 1 |
| UV-CMV_UNIQUE_HCMV_6HRS_UP | 61 | -0.327 | -1.547 | 0 | 0.002 | 1 |
| DNA_RECOMBINATION | 26 | -0.349 | -1.526 | 0 | 0.002 | 1 |
| MODULE_12 | 220 | 0.298 | 1.622 | 0 | 0.002 | 1 |
| NAKAJIMA_MCSMBP_MAST | 25 | 0.431 | 1.636 | 0 | 0.002 | 1 |
| BRG1_SW13_UP | 30 | 0.494 | 1.71 | 0 | 0.002 | 0.4 |
| HSA04012_ERBB_SIGNALING_PATHWAY | 50 | -0.329 | -1.522 | 0 | 0.002 | 1 |
| MICROTUBULE_BASED_PROCESS | 48 | -0.302 | -1.518 | 0 | 0.002 | 1 |
| BAF57_BT549_UP | 132 | -0.274 | -1.516 | 0 | 0.002 | 1 |
| SECONDARY_ACTIVE_TRANSMEMBRANE_TRANSPORTER_ ACTIVITY | 18 | -0.387 | -1.492 | 0 | 0.002 | 1 |
| HALMOS_CEBP_UP | 32 | -0.421 | -1.509 | 0 | 0.003 | 1 |
| ORGANELLE_LUMEN | 238 | 0.325 | 1.61 | 0 | 0.003 | 1 |
| MODULE_126 | 122 | 0.307 | 1.586 | 0 | 0.003 | 1 |
| CHEMOKINE_ACTIVITY | 19 | 0.517 | 1.588 | 0 | 0.003 | 1 |
| GNF2_PTPRC | 35 | 0.325 | 1.698 | 0 | 0.003 | 0.4 |
| MORF_MTA1 | 62 | 0.387 | 1.702 | 0 | 0.003 | 0.4 |
| GCM_CBFB | 53 | 0.435 | 1.703 | 0 | 0.003 | 0.4 |
| PHOSPHATIDYLINOSITOL_SIGNALING_SYSTEM | 41 | -0.329 | -1.489 | 0 | 0.003 | 1 |
| HSA03050_PROTEASOME | 21 | 0.456 | 1.704 | 0 | 0.003 | 0.4 |
| GNF2_ELAC2 | 31 | 0.447 | 1.705 | 0 | 0.003 | 0.4 |
| MORF_FEN1 | 50 | 0.471 | 1.705 | 0 | 0.003 | 0.4 |
| AGED_MOUSE_CORTEX_UP | 23 | 0.471 | 1.706 | 0 | 0.003 | 0.4 |
| MODULE_288 | 18 | 0.5 | 1.707 | 0 | 0.003 | 0.4 |
| UNDERHILL_PROLIFERATION | 15 | 0.469 | 1.709 | 0 | 0.003 | 0.4 |
| CATION_TRANSMEMBRANE_TRANSPORTER_ACTIVITY | 79 | -0.303 | -1.472 | 0 | 0.003 | 1 |
| HALMOS_CEBP_DN | 25 | -0.43 | -1.479 | 0 | 0.003 | 1 |
| AGEING_KIDNEY_UP | 198 | 0.325 | 1.592 | 0 | 0.003 | 1 |
| MORF_TFDP2 | 85 | -0.263 | -1.457 | 0 | 0.004 | 1 |
| REGULATION_OF_ANATOMICAL_STRUCTURE_ MORPHOGENESIS | 15 | 0.576 | 1.581 | 0 | 0.004 | 1 |
| SMITH_HCV_INDUCED_HCC_UP | 17 | -0.401 | -1.475 | 0 | 0.004 | 1 |
| CMV_ALL_UP | 68 | 0.319 | 1.584 | 0 | 0.004 | 1 |
| TRANSMEMBRANE_RECEPTOR_PROTEIN_TYROSINE_ KINASE_SIGNALING_PATHWAY | 38 | -0.342 | -1.456 | 0 | 0.004 | 1 |
| TAKEDA_NUP8_HOXA9_6H_UP | 30 | -0.296 | -1.473 | 0 | 0.004 | 1 |
| MODULE_93 | 102 | 0.367 | 1.688 | 0 | 0.004 | 0.6 |
| BCNU_GLIOMA_MGMT_48HRS_DN | 73 | 0.412 | 1.69 | 0 | 0.004 | 0.6 |
| H2O2_CSBRESCUED_C1_UP | 25 | 0.567 | 1.695 | 0 | 0.004 | 0.6 |
| MORF_IKBKG | 59 | 0.415 | 1.695 | 0 | 0.004 | 0.6 |
| MORF_RAD23B | 108 | 0.421 | 1.559 | 0 | 0.004 | 1 |
| STEMCELL_COMMON_UP | 120 | 0.295 | 1.565 | 0 | 0.005 | 1 |
| HSA04360_AXON_GUIDANCE | 55 | -0.328 | -1.42 | 0 | 0.005 | 1 |
| YU_CMYC_UP | 40 | -0.334 | -1.429 | 0.333 | 0.005 | 1 |
| MODULE_73 | 15 | -0.391 | -1.405 | 0.25 | 0.005 | 1 |
| RNA_TRANSCRIPTION_REACTOME | 22 | 0.409 | 1.549 | 0 | 0.006 | 1 |
| MODULE_284 | 19 | -0.437 | -1.402 | 0 | 0.006 | 1 |
| ROSS_CBF_LEUKEMIA | 30 | 0.394 | 1.543 | 0 | 0.006 | 1 |
| NEGATIVE_REGULATION_OF_CELLULAR_METABOLIC_ PROCESS | 123 | -0.322 | -1.807 | 0 | 0.006 | 0.2 |
| TPA_RESIST_MIDDLE_UP | 26 | 0.364 | 1.545 | 0 | 0.006 | 1 |
| RECEPTOR_SIGNALING_PROTEIN_ACTIVITY | 42 | -0.469 | -1.813 | 0 | 0.007 | 0.2 |
| CELL_CYCLE_CHECKPOINT | 19 | -0.487 | -1.831 | 0 | 0.007 | 0.2 |
| MEMBRANE_LIPID_BIOSYNTHETIC_PROCESS | 23 | 0.404 | 1.538 | 0 | 0.007 | 1 |
| IRITANI_ADPROX_VASC | 102 | 0.361 | 1.525 | 0 | 0.007 | 1 |
| SHEPARD_CRASH_AND_BURN_MUT_VS_WT_DN | 86 | -0.417 | -1.834 | 0 | 0.007 | 0.2 |
| MODULE_332 | 22 | -0.468 | -1.386 | 0 | 0.007 | 1 |
| UVB_NHEK1_UP | 83 | 0.425 | 1.515 | 0 | 0.007 | 1 |
| MORF_CDH4 | 47 | -0.341 | -1.383 | 0 | 0.007 | 1 |
| HADDAD_CD45CD7_PLUS_VS_MINUS_DN | 46 | -0.373 | -1.39 | 0 | 0.008 | 1 |
| ZHAN_TONSIL_BONEMARROW | 21 | 0.512 | 1.517 | 0 | 0.008 | 1 |
| BLEO_HUMAN_LYMPH_HIGH_24HRS_UP | 61 | 0.419 | 1.671 | 0 | 0.008 | 0.8 |
| PROGRAMMED_CELL_DEATH | 210 | 0.306 | 1.671 | 0 | 0.008 | 0.8 |
| GNF2_INPP5D | 25 | 0.526 | 1.671 | 0 | 0.008 | 0.8 |
| VEGF_MMMEC_3HRS_UP | 42 | 0.356 | 1.672 | 0 | 0.008 | 0.8 |
| UNFOLDED_PROTEIN_BINDING | 22 | 0.571 | 1.674 | 0 | 0.008 | 0.8 |
| ELECTRON_TRANSPORT_GO_0006118 | 25 | 0.473 | 1.674 | 0 | 0.009 | 0.8 |
| WONG_IFNA_HCC_RESISTANT_VS_SENSITIVE_DN | 20 | 0.54 | 1.679 | 0 | 0.009 | 0.8 |
| HOHENKIRK_MONOCYTE_DEND_UP | 62 | 0.41 | 1.505 | 0 | 0.009 | 1 |
| HSA05215_PROSTATE_CANCER | 52 | 0.452 | 1.487 | 0 | 0.009 | 1 |
| MODULE_316 | 15 | -0.433 | -1.359 | 0 | 0.009 | 1 |
| REPRODUCTIVE_PROCESS | 56 | -0.283 | -1.364 | 0 | 0.01 | 1 |
| GCM_APEX1 | 76 | 0.463 | 1.664 | 0 | 0.01 | 0.8 |
| PASSERINI_APOPTOSIS | 24 | 0.413 | 1.665 | 0 | 0.01 | 0.8 |
| MODULE_299 | 23 | 0.572 | 1.665 | 0 | 0.01 | 0.8 |
| MODULE_6 | 225 | 0.296 | 1.667 | 0 | 0.01 | 0.8 |
